# Diastereoselective synthesis of tetrahydroisoquinolines through the Pd-catalyzed post-Ugi transformations

**DOI:** 10.1039/d6ra06856a

**Published:** 2026-10-01

**Authors:** Manzoor Zaman, Ainara Bekbolatova, Sofia D. Martynova, Azamat Begenov, Niyaz Amire, Muhammad Hasan, Azat M. Makhmet, Rakhmetulla Yerkassov, Andrey Belyaev, Adilet Bekmagambetov, Davit Hayrapetyan, Olga P. Pereshivko, Vsevolod A. Peshkov, Anatoly A. Peshkov

**Affiliations:** a Soochow University, College of Chemistry, Chemical Engineering and Materials Science, Dushu Lake Campus Suzhou 215123 P. R. China; b Guangzhou Medical University, Guangzhou Municipal and Guangdong Provincial Key Laboratory of Molecular Target & Clinical Pharmacology, The NMPA and State Key Laboratory of Respiratory Disease, School of Pharmaceutical Sciences Guangzhou Guangdong 511436 China; c Nazarbayev University, Department of Chemistry, School of Sciences and Humanities Astana 010000 Kazakhstan vsevolod.peshkov@nu.edu.kz anatolii.peshkov@nu.edu.kz; d National Laboratory Astana, Nazarbayev University Astana 010000 Kazakhstan; e L. N. Gumilyov Eurasian National University, Department of Chemistry, Faculty of Natural Sciences Astana 010000 Kazakhstan; f Department of Chemistry, University of Eastern Finland FI-80101 Joensuu Finland

## Abstract

We present a diastereoselective approach to sp^3^-enriched tetrahydroisoquinolines from Ugi-derived propargylamides through a sequence combining Pd-catalyzed reductive Heck cyclization with heterogeneous double-bond hydrogenation. Although the initial reductive Heck annulation generally proceeded with weak stereocontrol over the exocyclic alkene geometry, the subsequent Pd-catalyzed hydrogenation proved highly diastereoselective, predominantly affording tetrahydroisoquinolines with a *cis* relative configuration. The hydrogenation was largely stereoconvergent, delivering the same major *cis* diastereomer irrespective of the *E*/*Z* geometry of the intermediate isoquinolines.

## Abstract 2

A diastereoselective approach to sp^3^-enriched tetrahydroisoquinolines from Ugi-derived propargylamides *via* a sequence of Pd-catalyzed reductive Heck cyclization and heterogeneous double-bond hydrogenation is reported.

## Graphical abstract

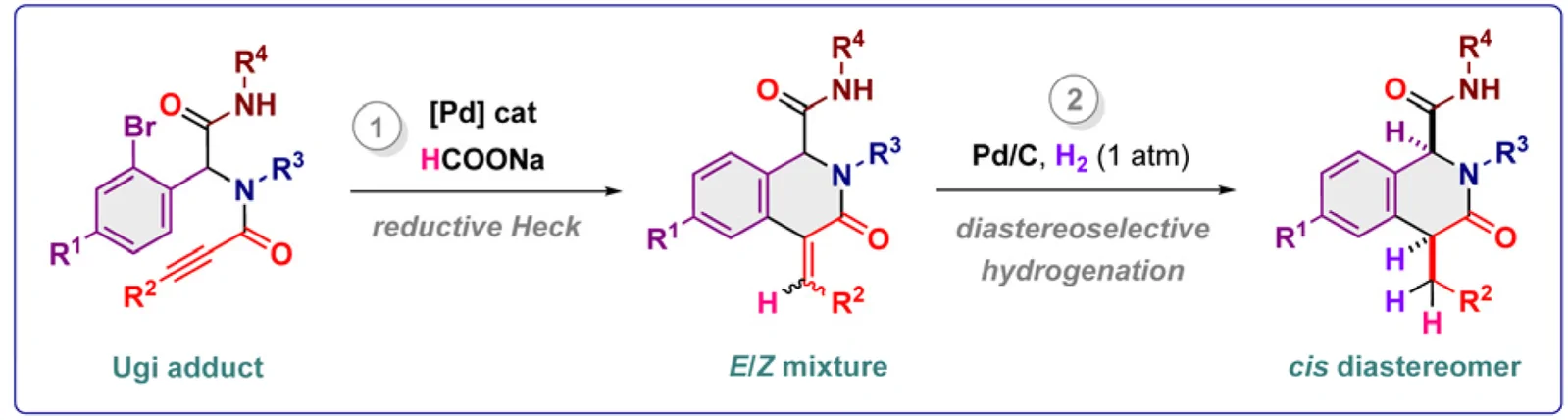

## Introduction

Isocyanide-based multicomponent reactions, notably the Ugi four-component reaction (U4CR), have proven highly effective in constructing complex organic frameworks from simple precursors.^1–5^ Further expansion of the chemical space accessible through Ugi chemistry can be achieved by combining it with post-MCR modifications^6–10^ within the framework of diversity-oriented synthesis (DOS).^11^ This enabled the generation of libraries of potentially bioactive heterocycles^12–17^ for medicinal chemistry applications.^18–20^

A typical Ugi reaction produces a dipeptide-like scaffold featuring a stereocenter at the peptidyl position. Thus, its stereochemical aspect contributes significantly to the overall structural diversity and complexity of the resulting products. Over the past decade, considerable progress has been achieved in controlling the stereochemistry of Ugi adducts, both during the multicomponent reaction itself^21–25^ and in subsequent post-MCR modifications.^26^

In terms of their effective utilization in post-MCR transformations, propiolic acid-derived Ugi adducts have attracted particular interest owing to the versatile reactivity of the alkyne moiety, which enables diverse modes of triple-bond activation.^27^ Notable examples include enolization-driven Michael additions,^28–31^ phosphine-catalyzed umpolung Michael additions,^32–37^ intramolecular Diels–Alder reactions^38–41^ and 1,3-dipolar cycloadditions,^42,43^ as well as coinage metals catalyzed hydroarylations,^44–48^ heterocyclizations^49–51^ and tandem processes.^52–58^

Triple-bond carbopalladation has emerged as a powerful tool for developing efficient annulation strategies^59–63^ that have also been extensively explored for propiolic acid-derived Ugi adducts. In this context, the Balalaie group investigated annulation pathways leading to five-membered ring systems, specifically targeting the synthesis of the 3-methyleneindolin-2-one core.^64–67^ In 2011, they employed Ugi adducts 5, generated from phenyl propiolic acid (2a) and *ortho*-iodoaniline (3), in a palladium-catalyzed Heck/Buchwald tandem reaction with anilines 6 to afford (*Z*)-3-(phenyl(phenylamino)methylene)indolin-2-ones 7 (Scheme 1A).^67^ The transformation proceeded with complete stereocontrol, delivering exclusively the *Z* configuration of the exocyclic double bond, which corresponds to the *anti*-mode of triple bond bis-functionalization.

**Scheme 1:**
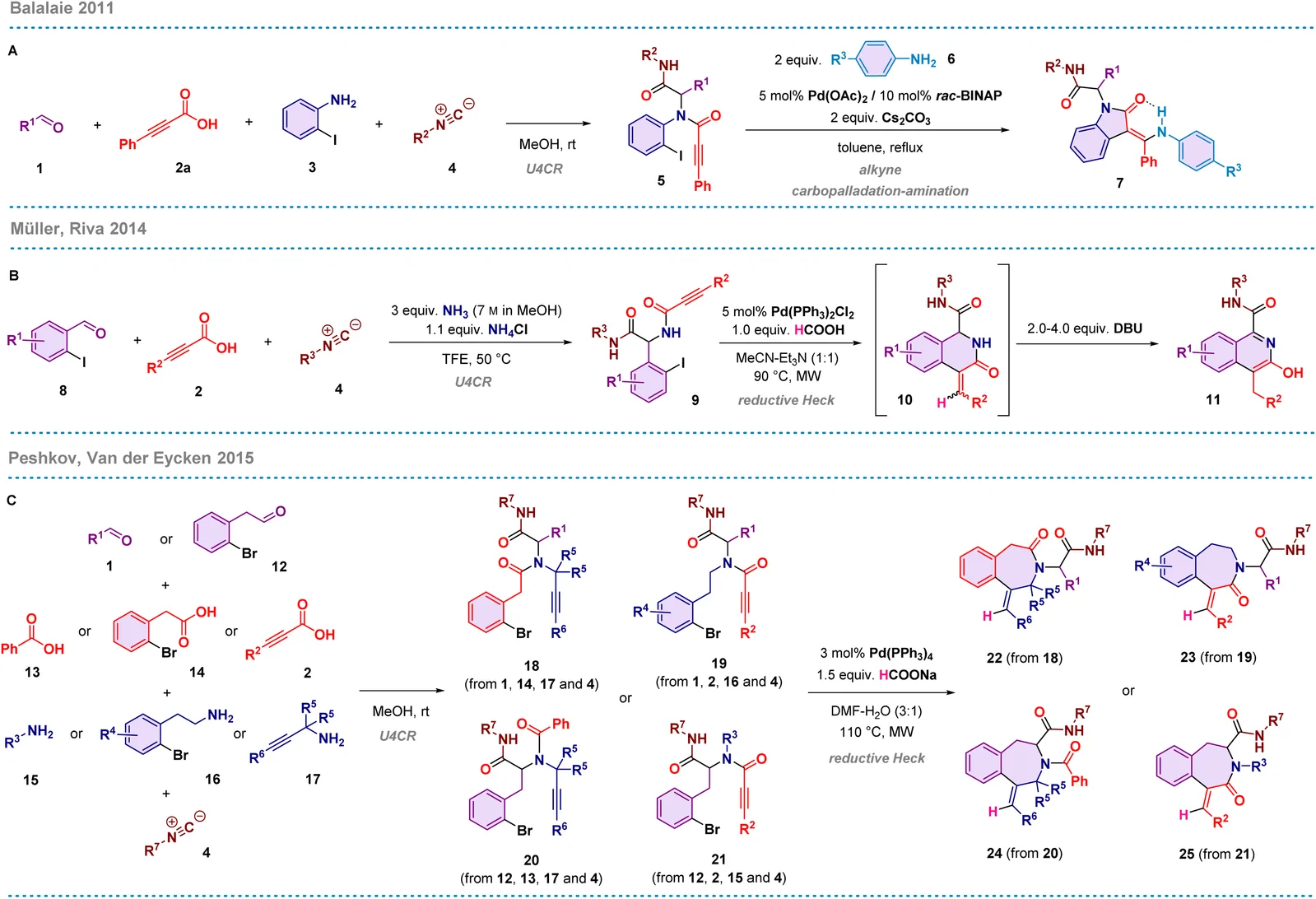
Post-Ugi transformation of propiolic acid-derived adducts involving triple-bond carbopalladation.

Müller, Riva, and co-workers studied the post-Ugi synthesis of intensely blue-emissive furo[2,3-*c*]isoquinolines^68,69^ and 3-hydroxyisoquinolines^70^*via* palladium-catalyzed processes involving alkyne carbopalladation. In the latter case, the sequence began with a Ugi reaction between *ortho*-iodobenzaldehydes 8, 3-substituted propiolic acids 2, and isocyanides 4, using ammonia as the amine component (Scheme 1B). The resulting propargylic amides 9 were subjected to a reductive Heck annulation to assemble 3-oxo-isoquinolines 10, which were subsequently transformed into 3-hydroxyisoquinolines 11*via* base-promoted aromatization. In addition to achieving a highly emissive profile, the aromatization step was beneficial because the initial annulation, proceeding *via* a 6-exo-dig cyclization, generated an exocyclic double bond in a non-stereoselective manner.

In 2015, Peshkov, Van der Eycken, and co-workers explored a related two-step sequence involving a Ugi reaction followed by reductive Heck cyclization to provide access to a 3-benzazepine framework in a diversity-oriented fashion (Scheme 1C).^71^ This tunable strategy enabled the systematic variation of substituents and the flexible distribution of the required functional groups among the Ugi reaction components. As a result, it provided access to four distinct types of 3-benzazepines 22–25 with different substitution patterns and full stereocontrol over the exocyclic double bond. Notably, the aryl bromide functionality could be incorporated into the aldehyde, acid, or amine component, while the alkyne moiety was embedded either in propiolic acid 2 or in propargylamine 17.

## Results and discussion

In the present work we attempt to merge post-Ugi reductive Heck cyclization with heterogeneous double bond hydrogenation for the diastereoselective synthesis of tetrahydroisoquinolines 29 (Scheme 2). The key hypothesis is that the insufficient stereocontrol over the configuration of the exocyclic double bond in the intermediate isoquinolines 28 could be compensated by high diastereocontrol in the formation of final tetrahydroisoquinolines 29. Considering that one face of 28 is sterically blocked by the isocyanide-derived amide moiety, hydrogenation of the double bond should predominantly afford the *cis* diastereomer of 29, with the configuration of the newly formed stereocenter being governed by the Ugi-derived stereocenter. In addition, the degree of saturation for the resulting heterocyclic core is increased, in line with our recently developed strategy for the construction of sp^3^-enriched Ugi-derived heterocycles through the incorporation of a post- or pre-Ugi Pd-catalyzed heterogeneous double-bond hydrogenation step.^32,72^

**Scheme 2:**
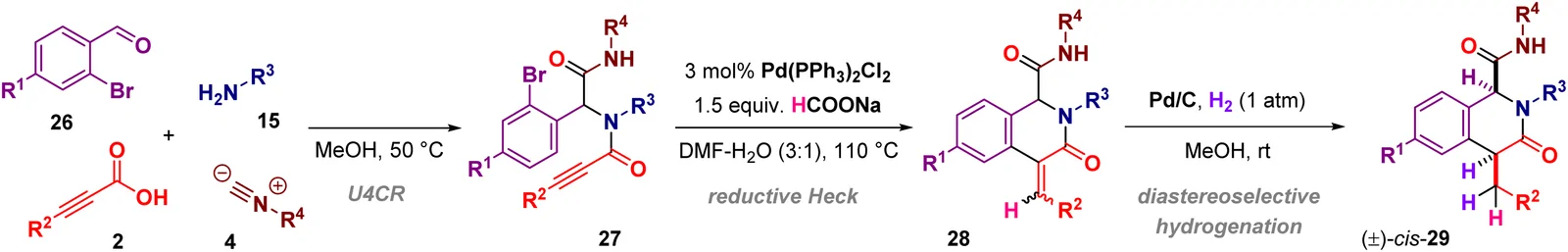
Diastereoselective synthesis of tetrahydroisoquinolines 29*via* post-Ugi reductive Heck cyclization and subsequent double bond hydrogenation.

First, we prepared a series of Ugi adducts 27 by reacting *ortho*-bromobenzaldehydes 26 with 3-substituted propiolic acids 2, amines 15, and isocyanides 4 (Table 1). All of the Ugi reactions proceeded smoothly, affording the desired propargylamides 27 in consistently good yields. The specific structures of the building blocks employed are provided in the SI (Table S1).

**Table 1:** Scope of the diastereoselective synthesis of tetrahydroisoquinolines 29

Entry	Ugi adduct 27^a^^b^	Isoquinolines 28^b^^c^	Tetrahydroisoquinolines 29^b^^d^
1	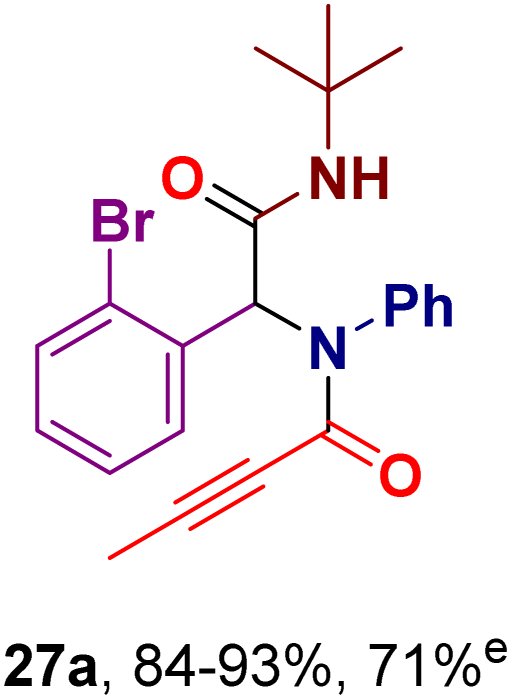	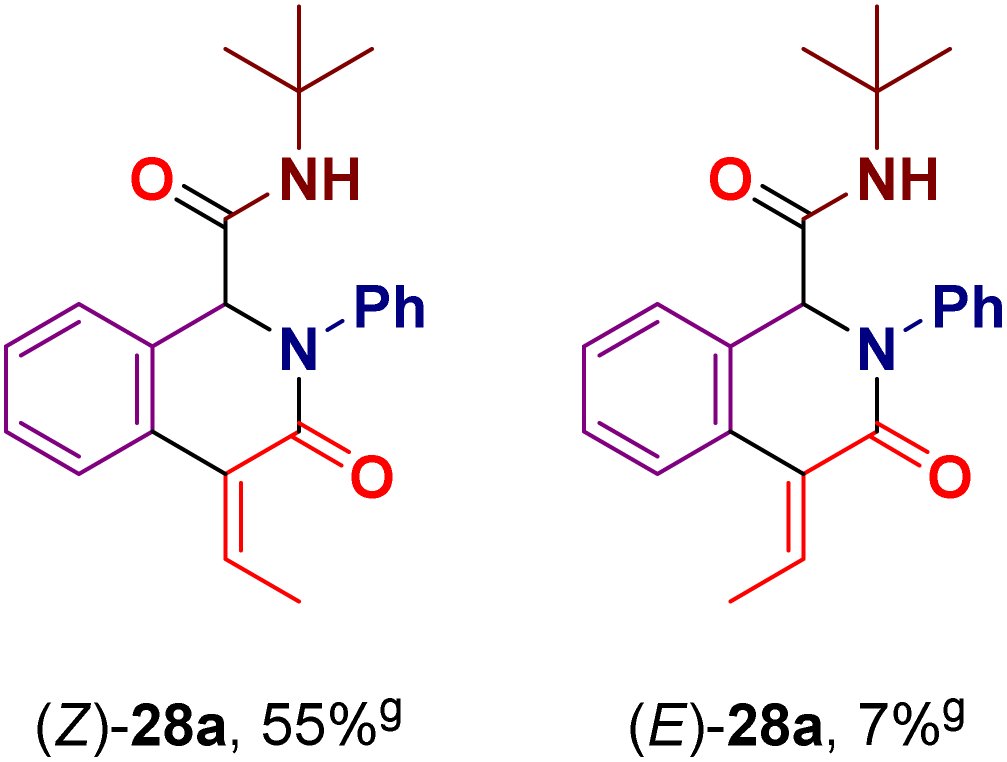	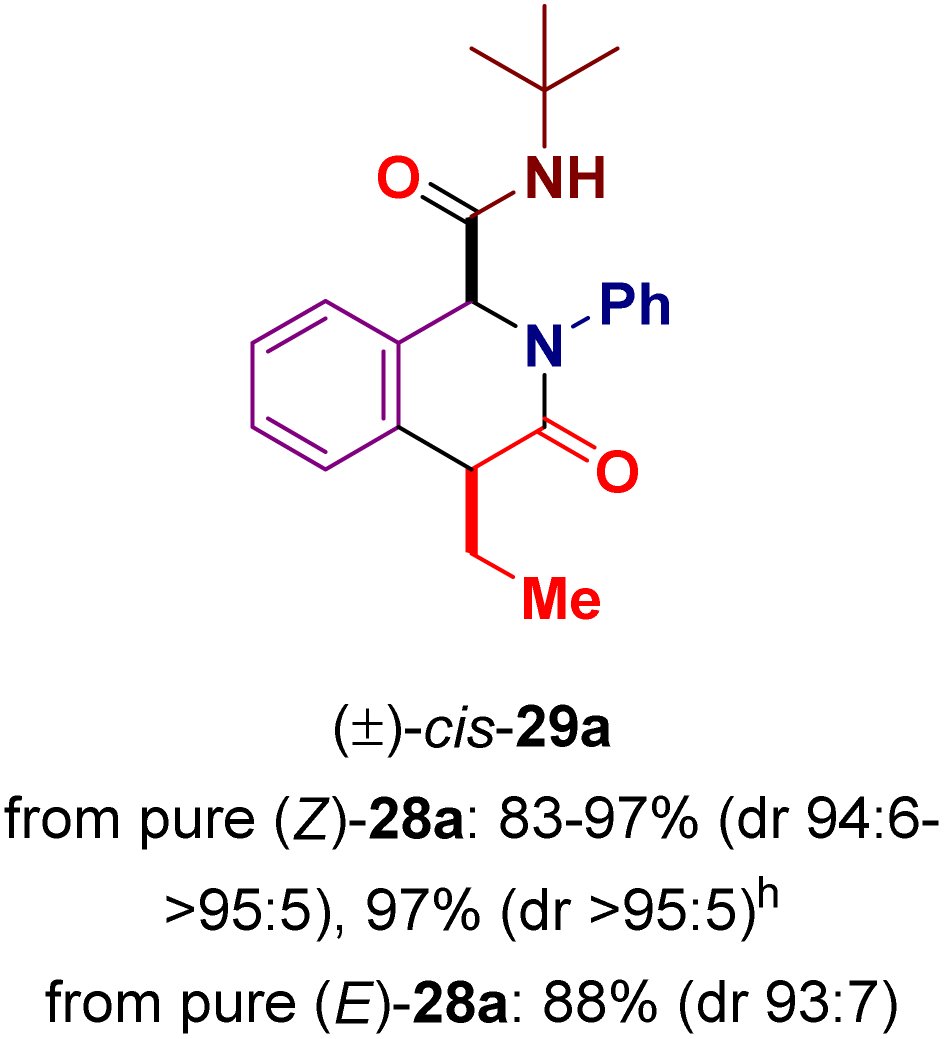
2	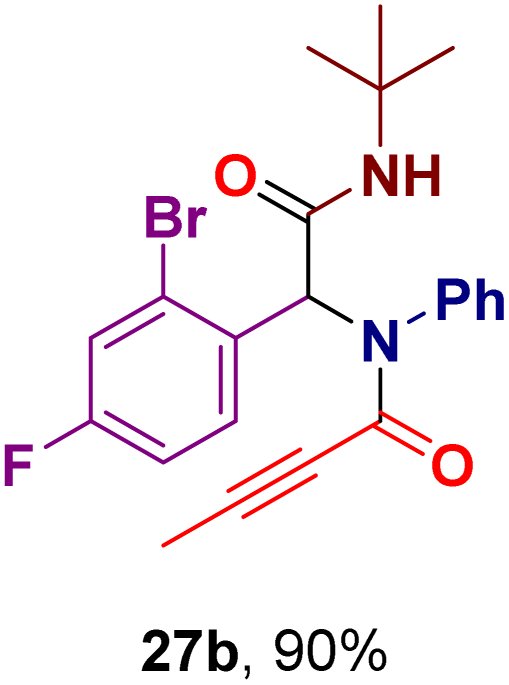	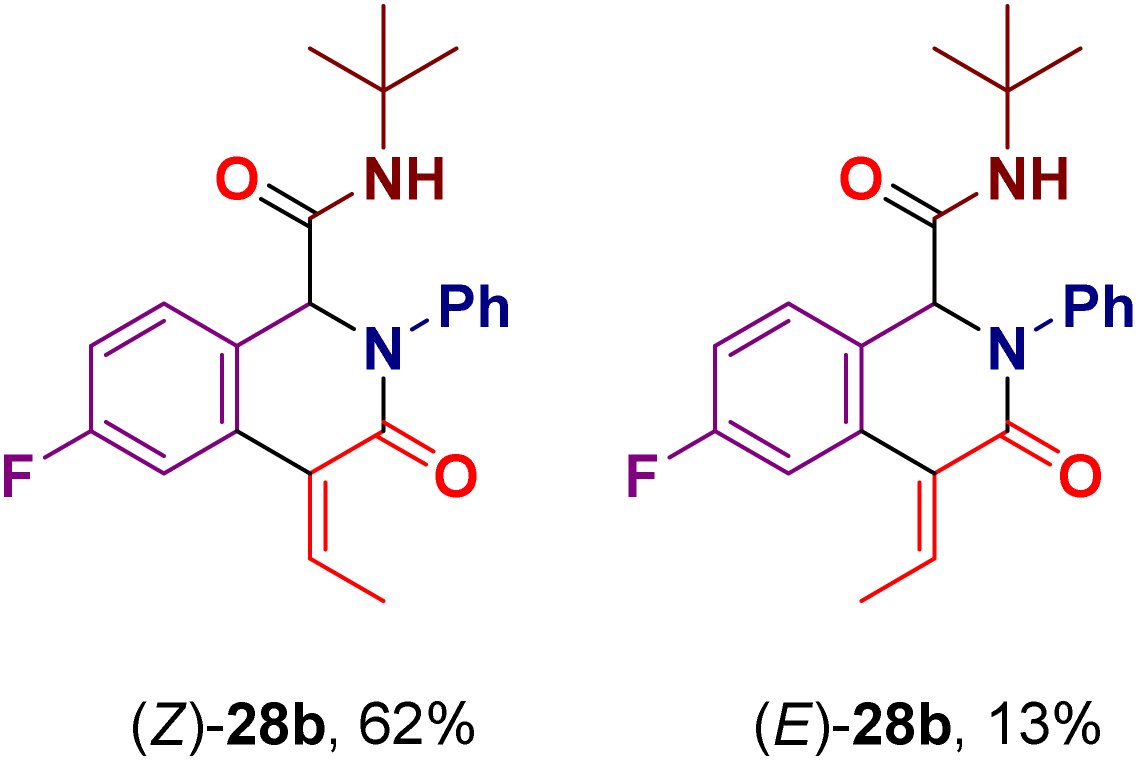	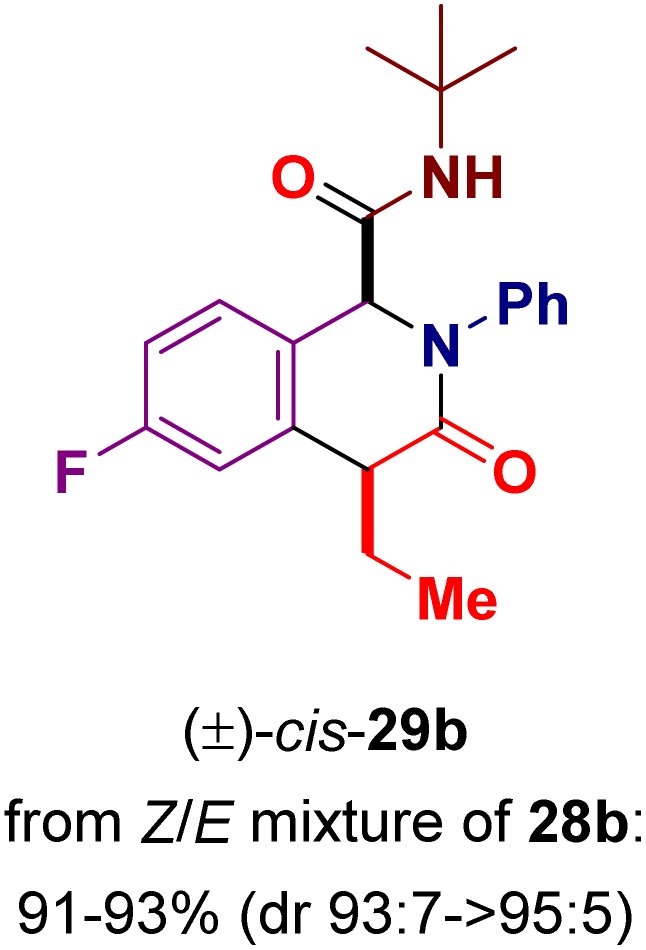
3	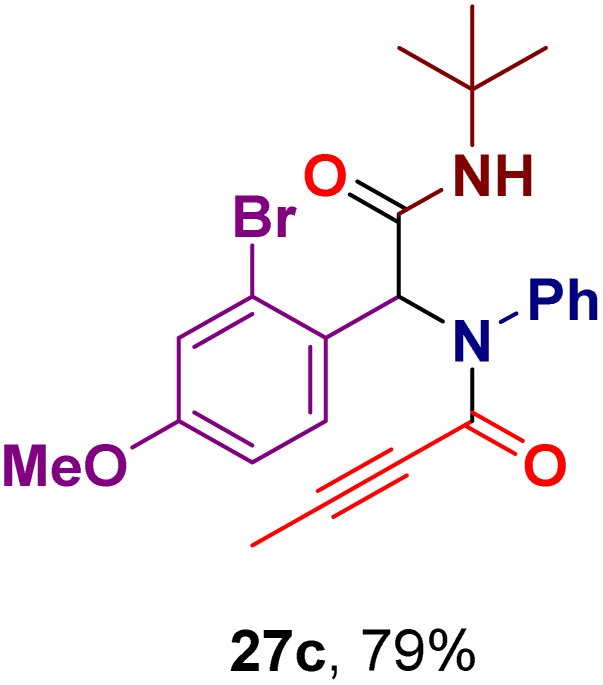	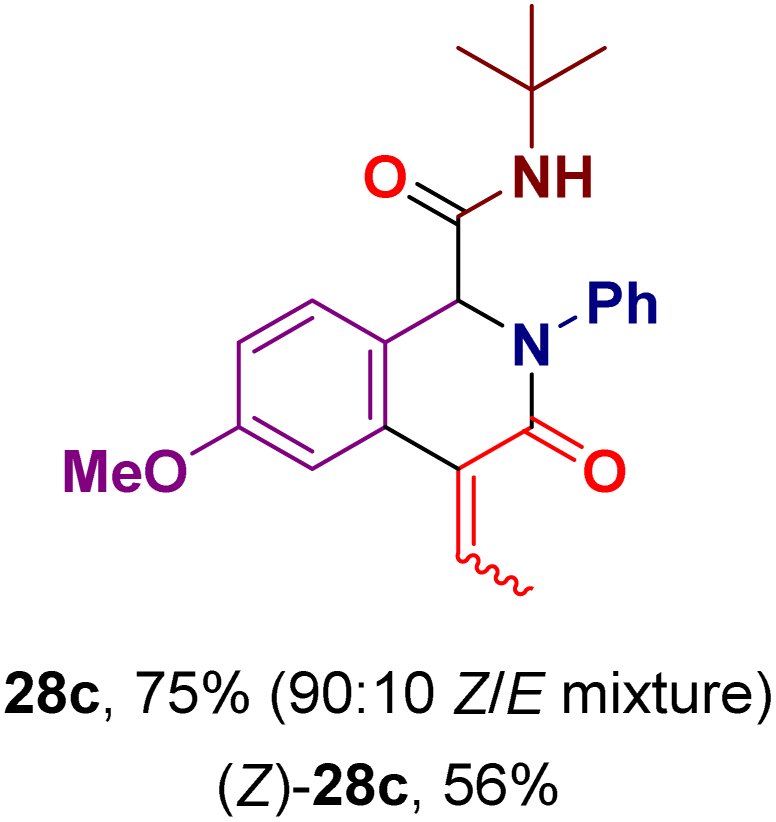	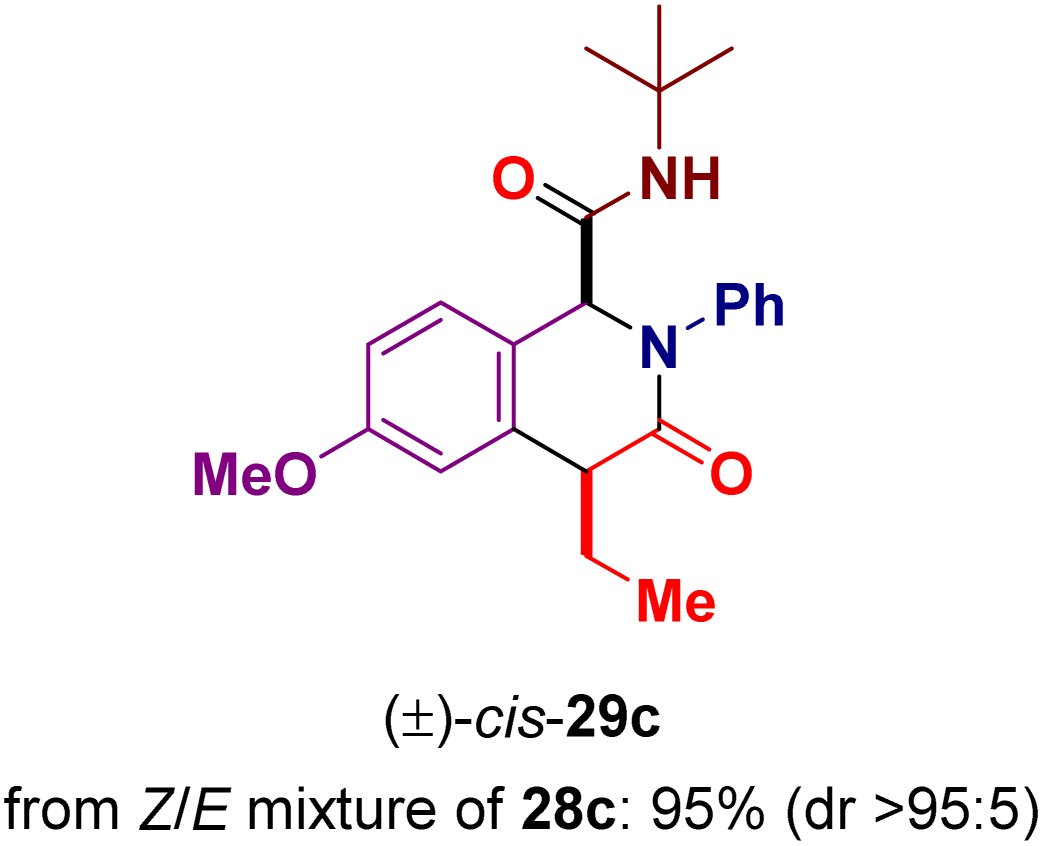
4	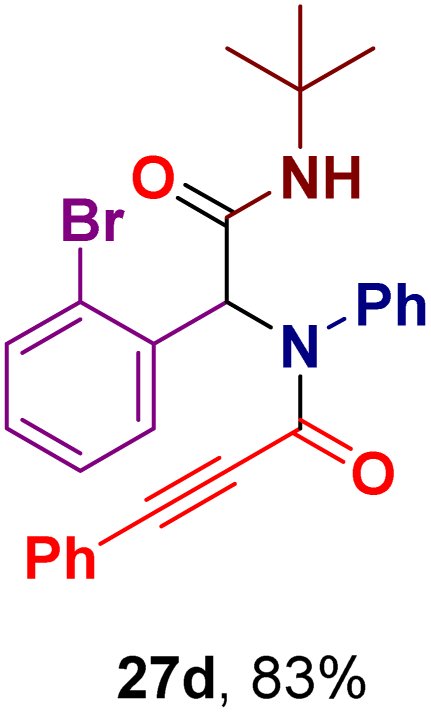	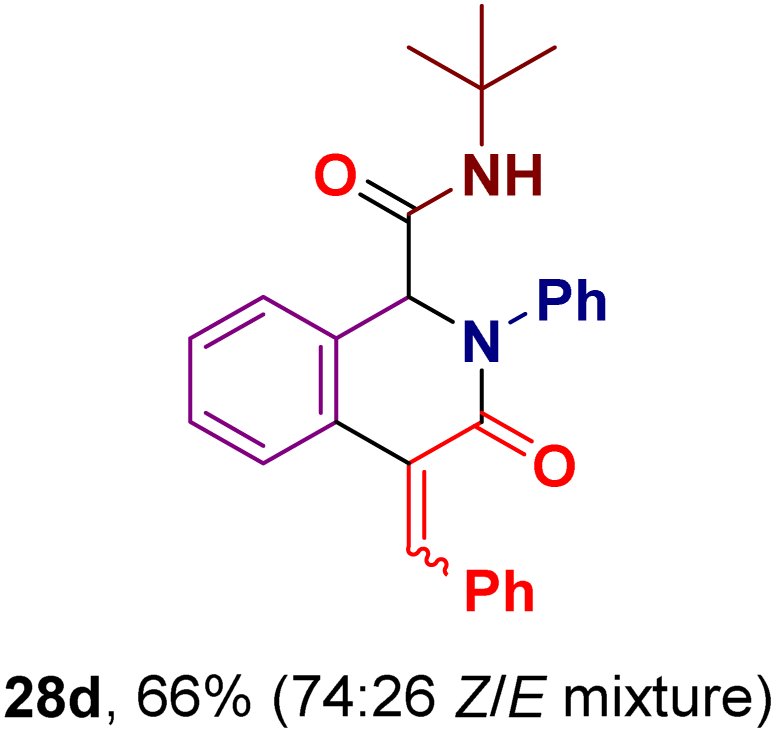	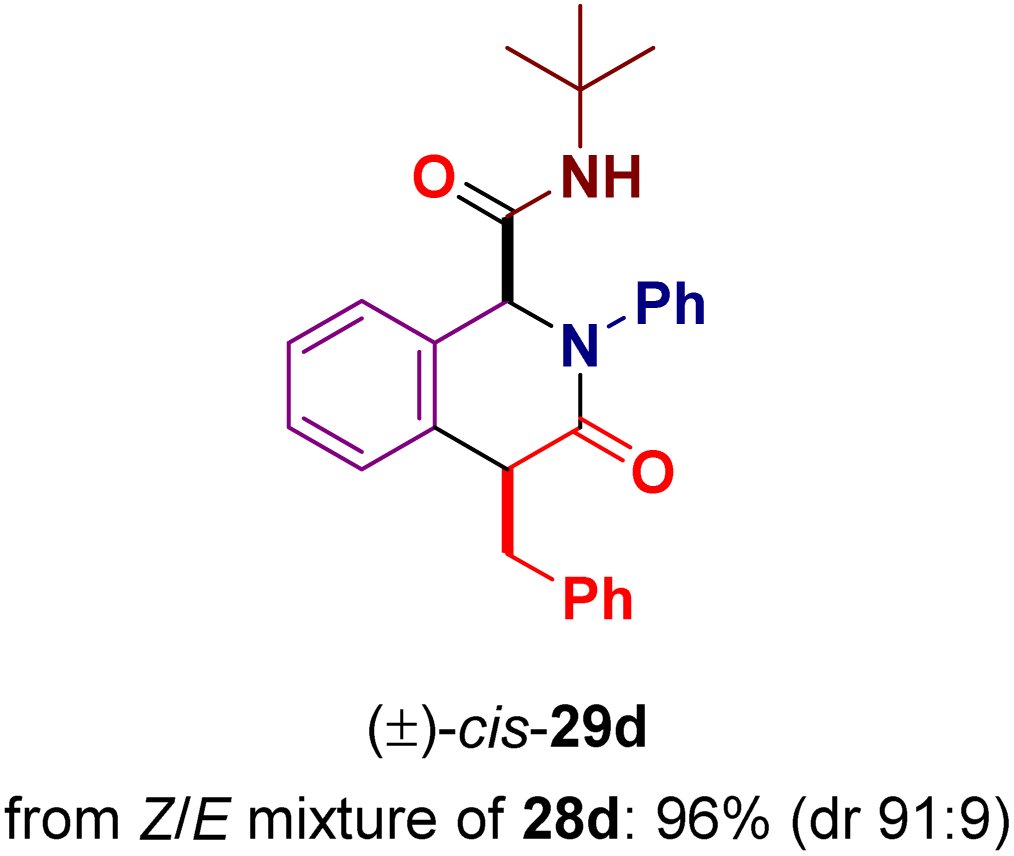
5	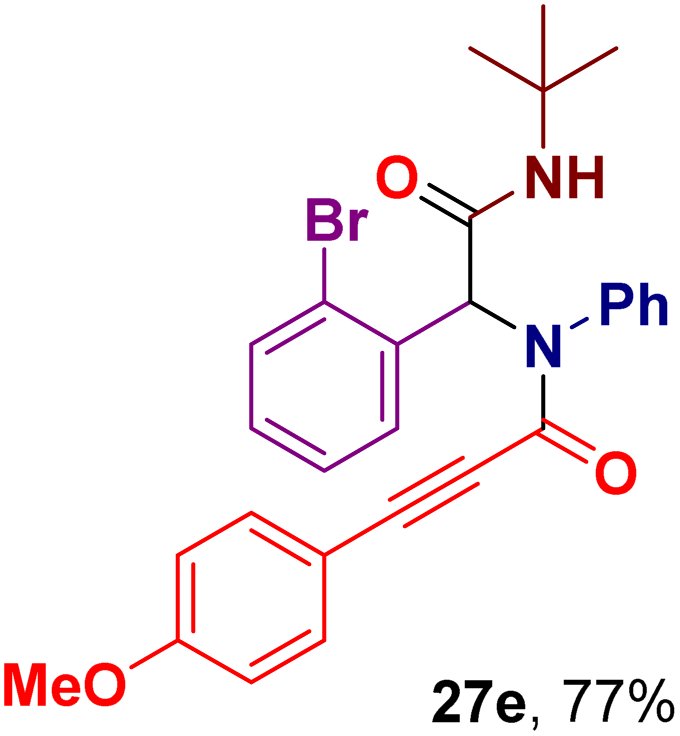	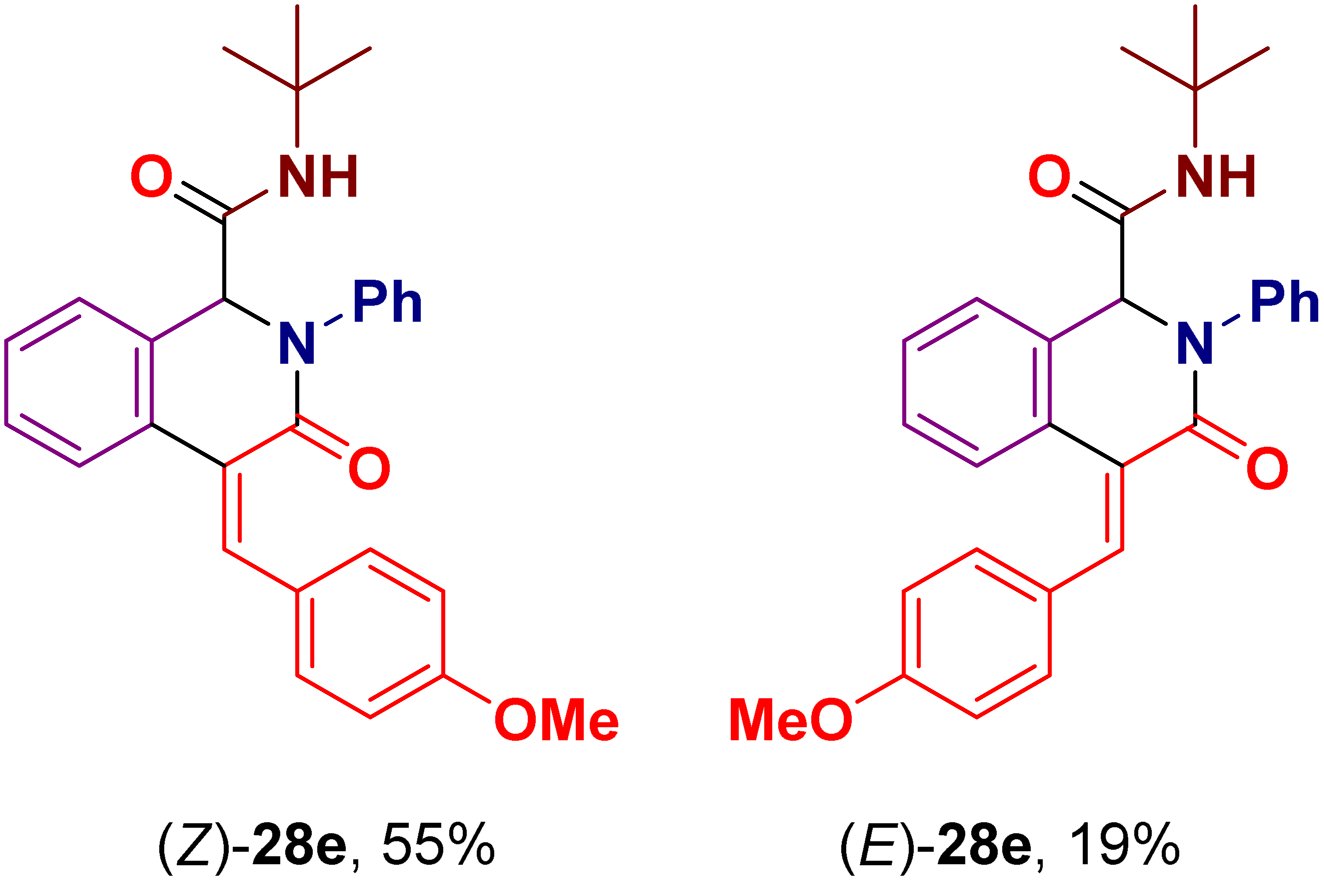	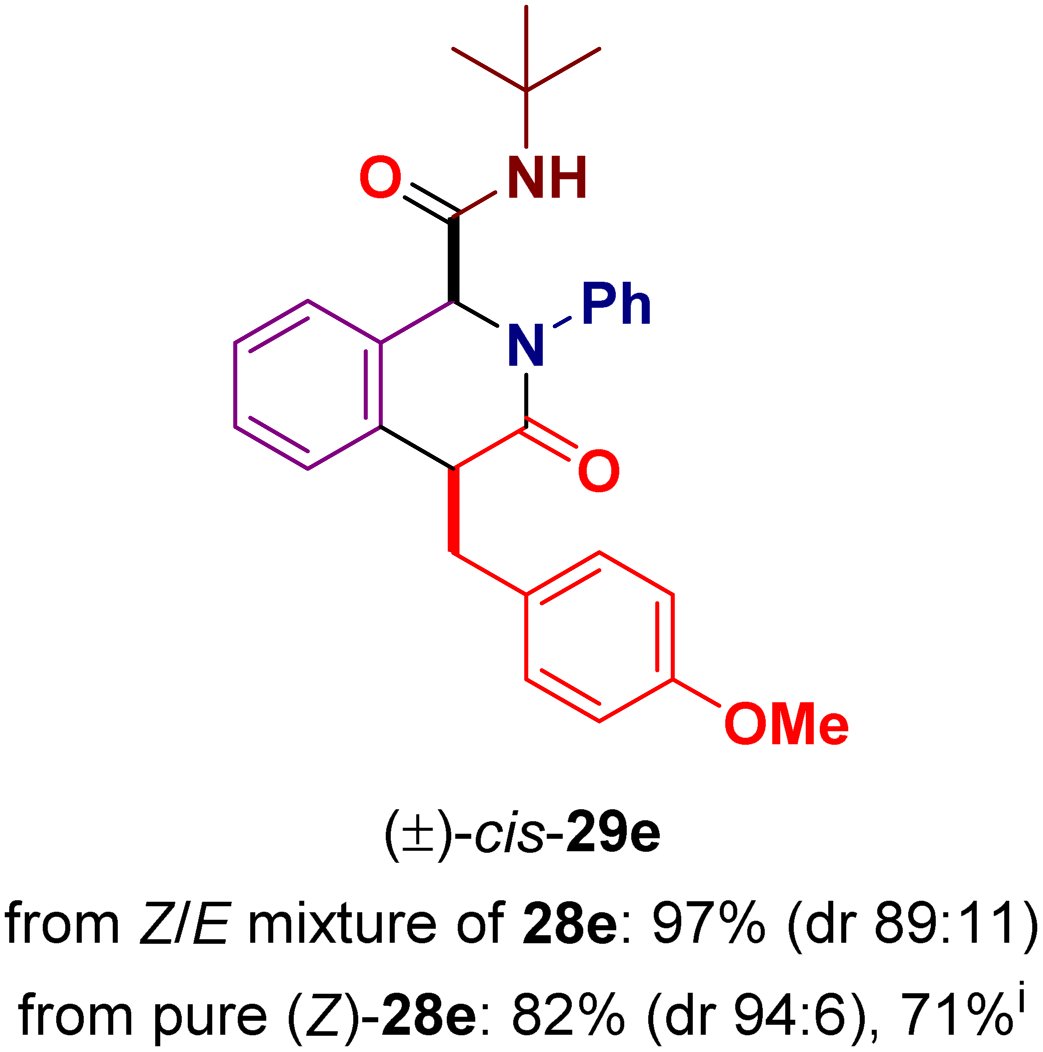
6	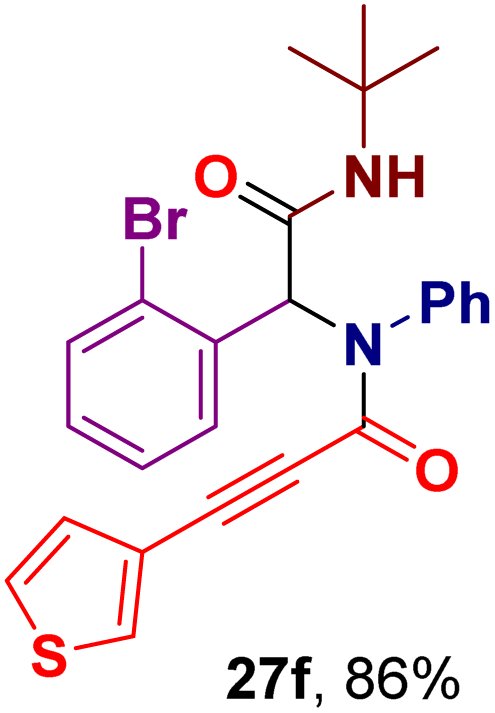	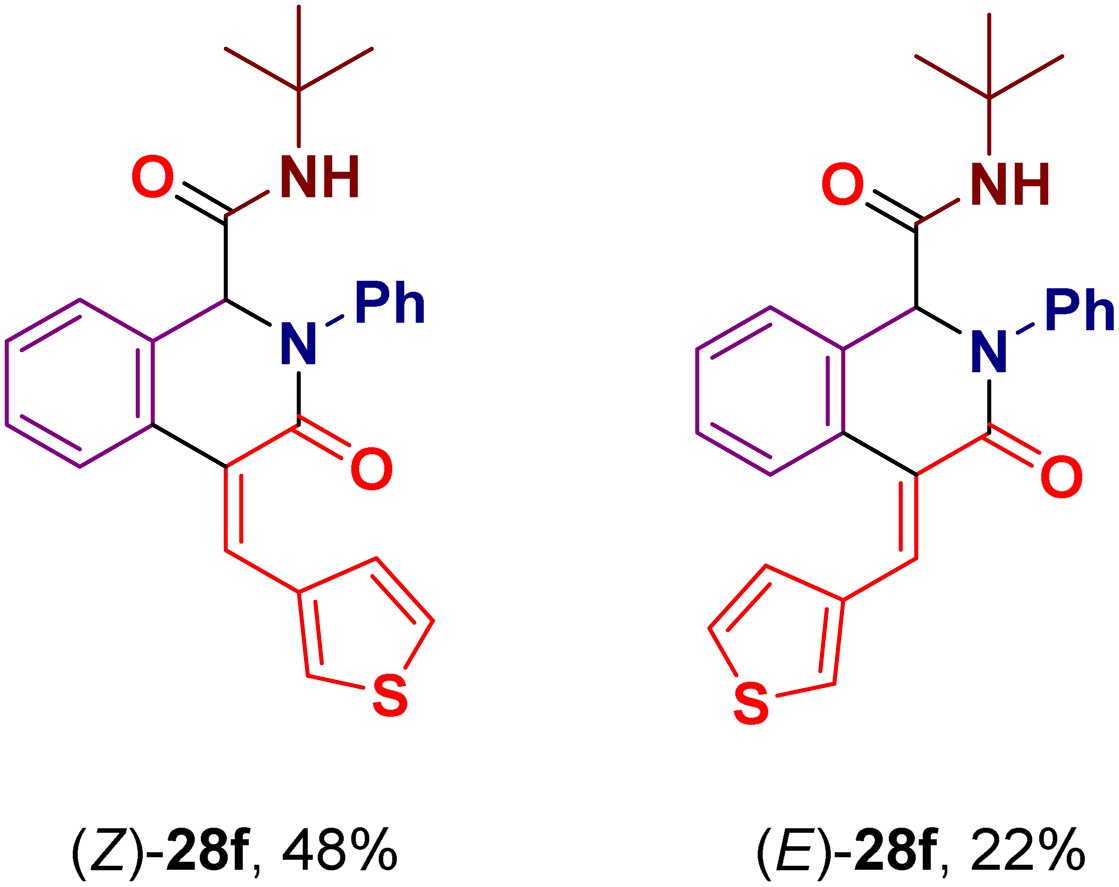	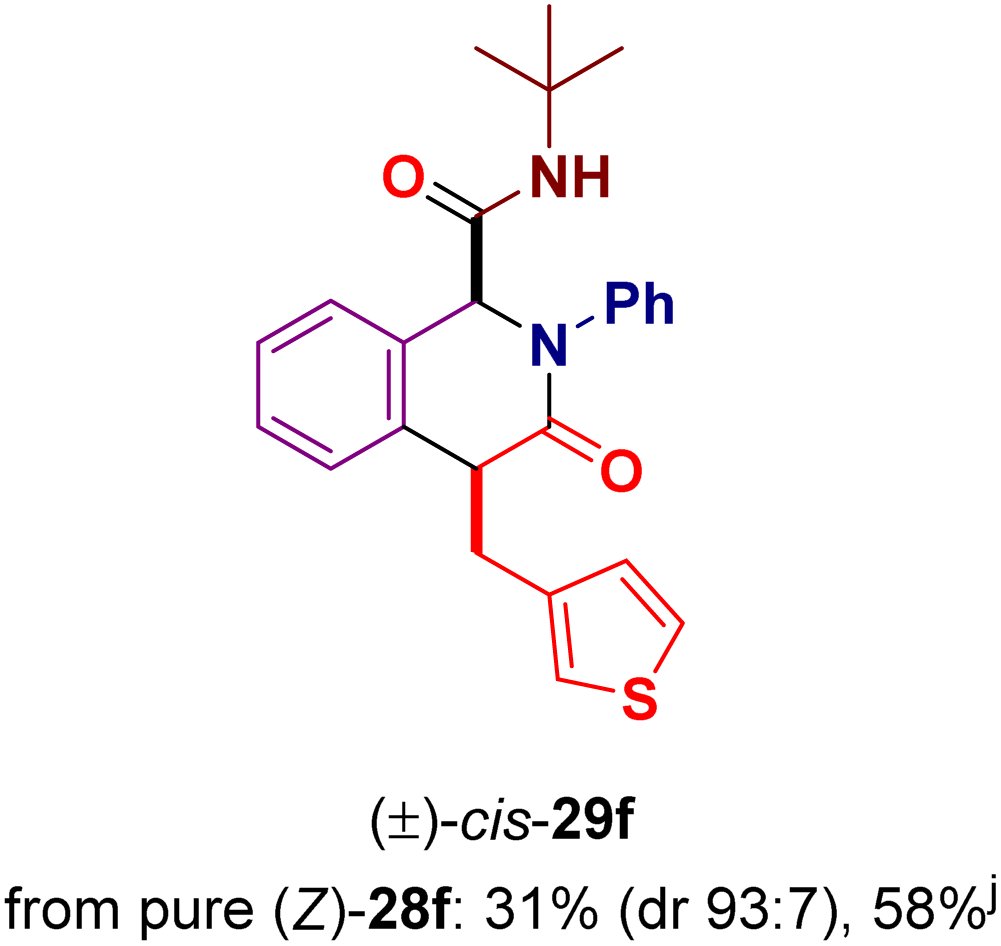
7	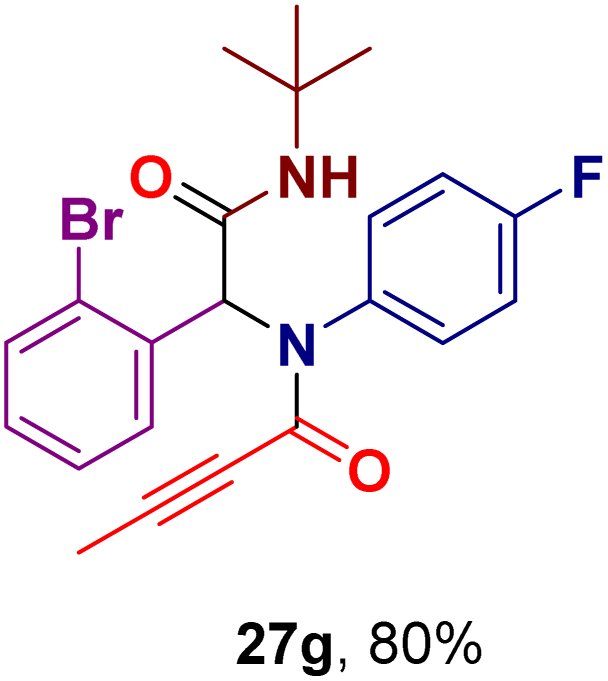	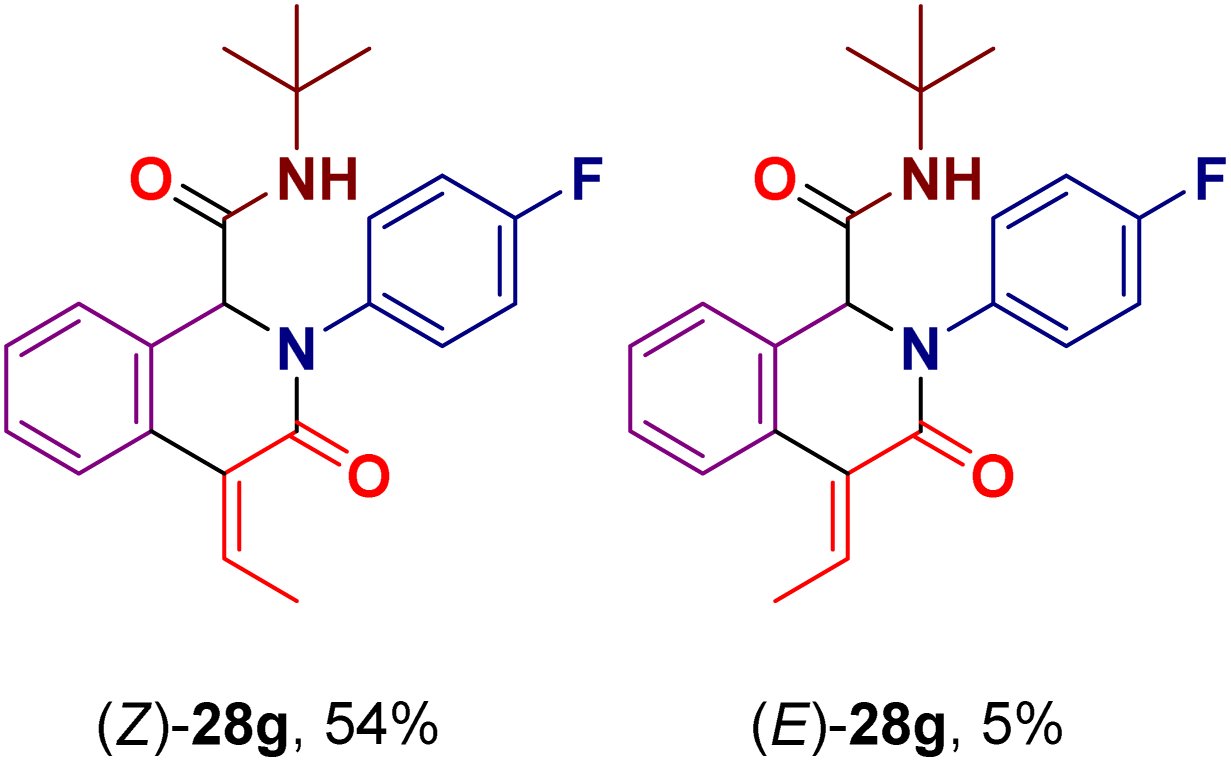	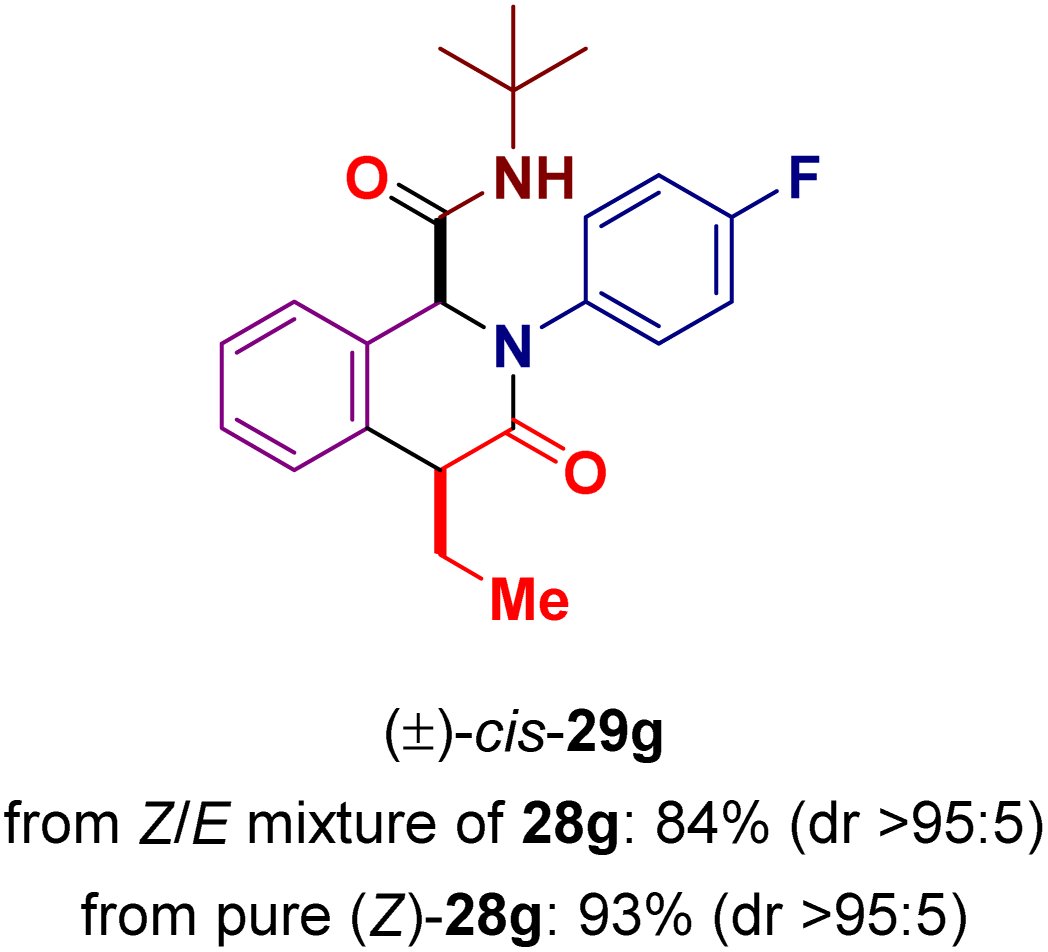
8	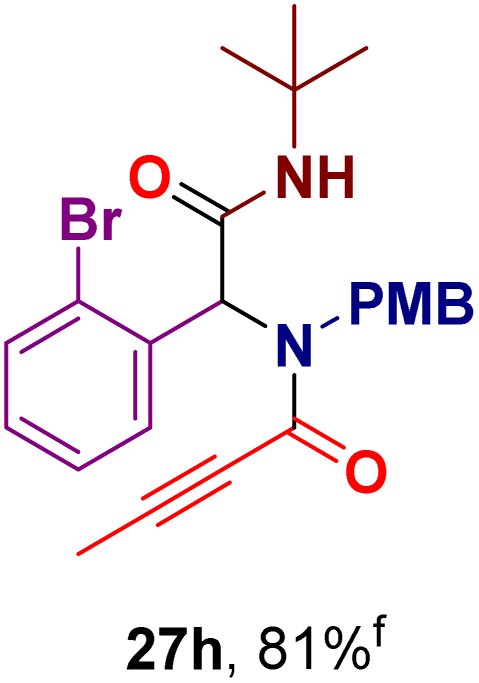	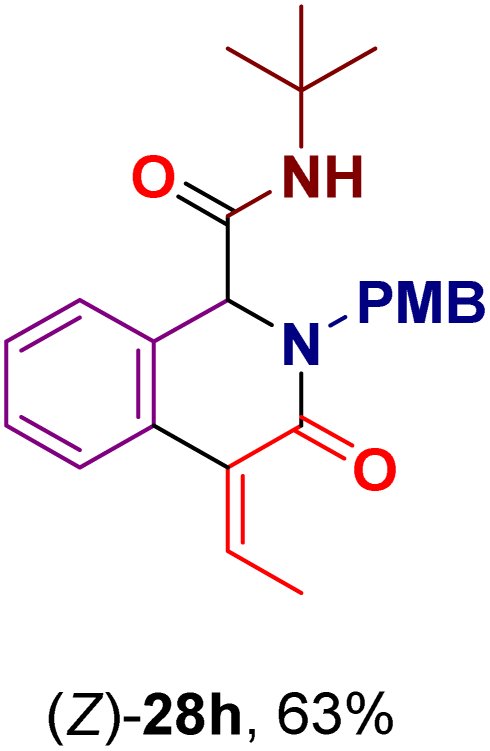	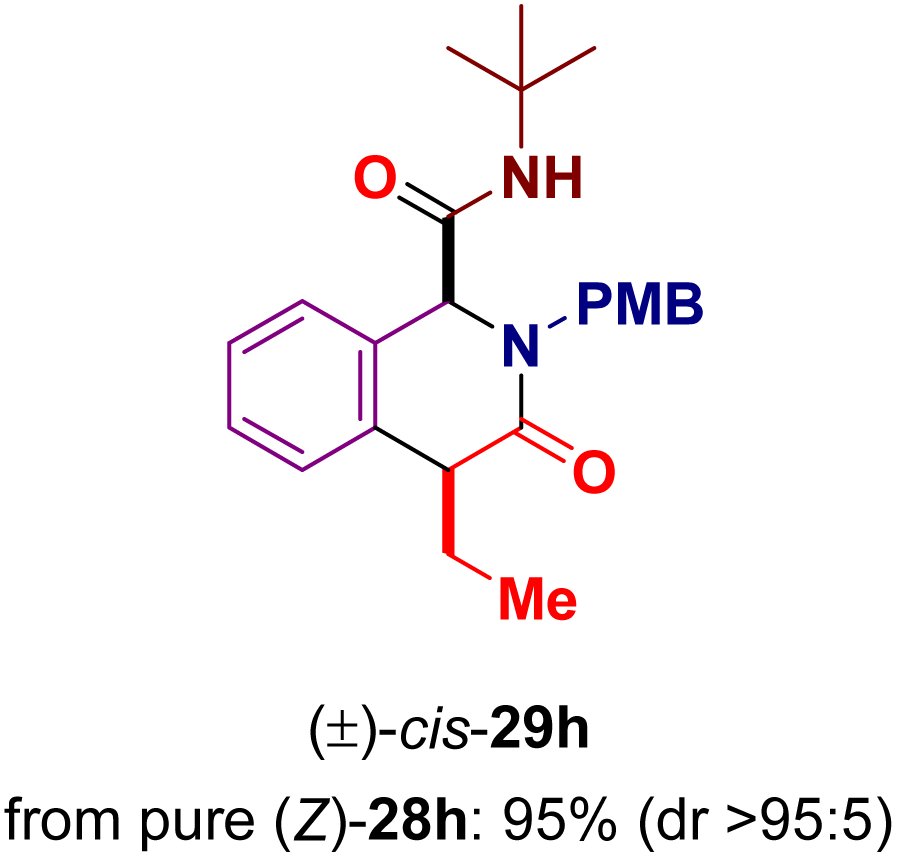
9	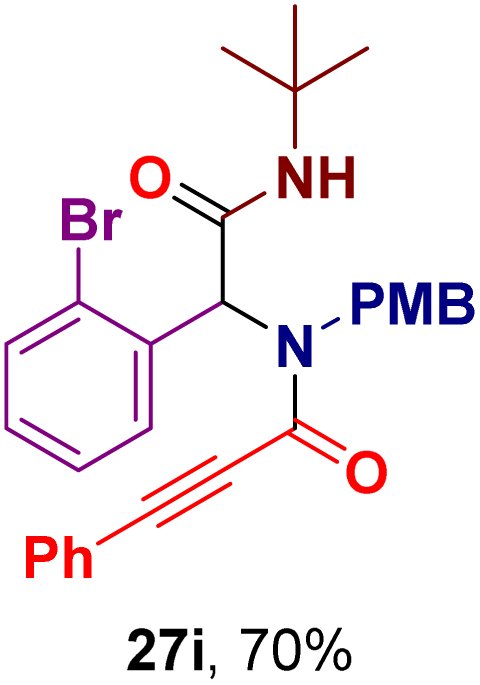	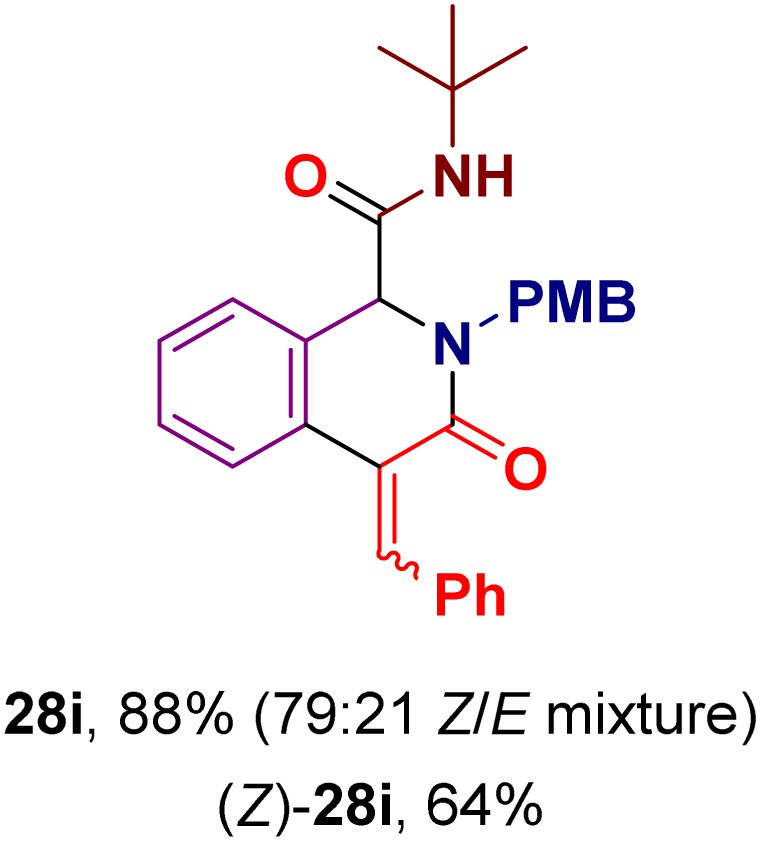	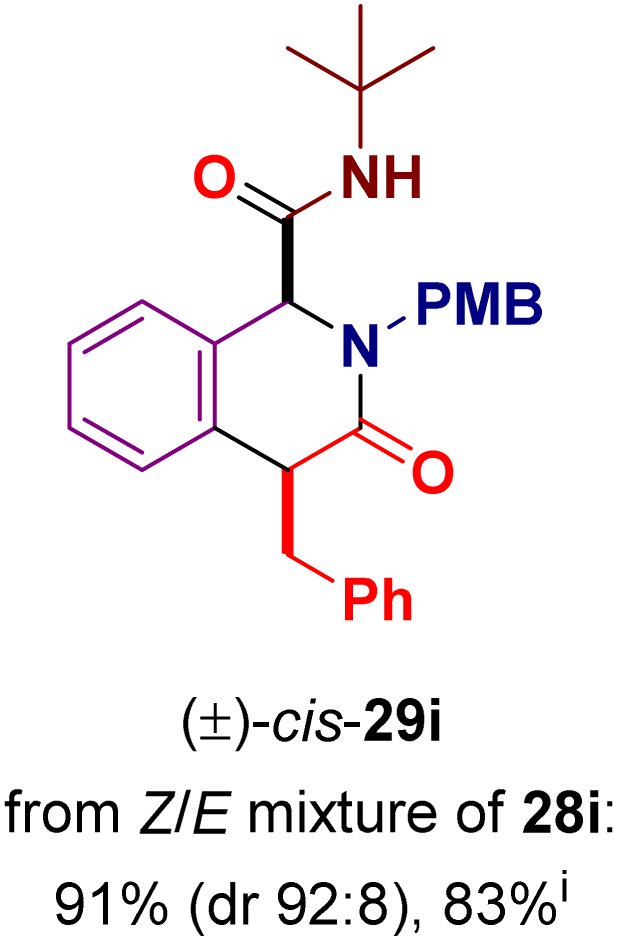
10	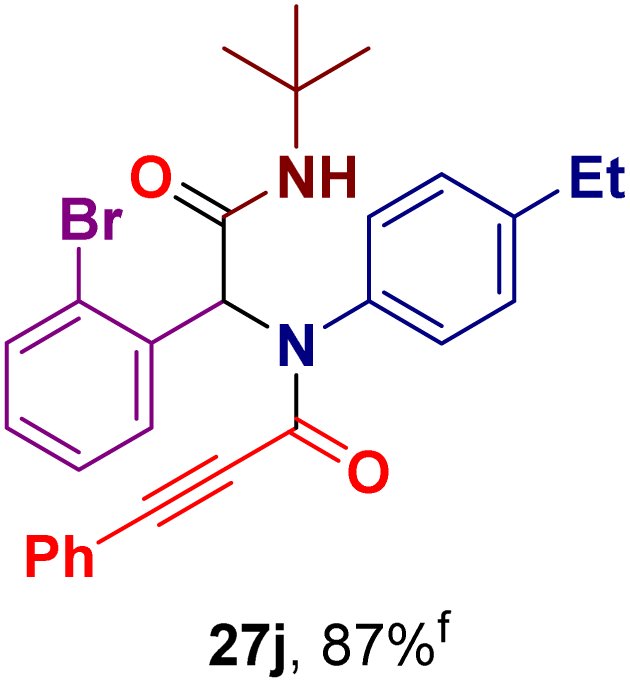	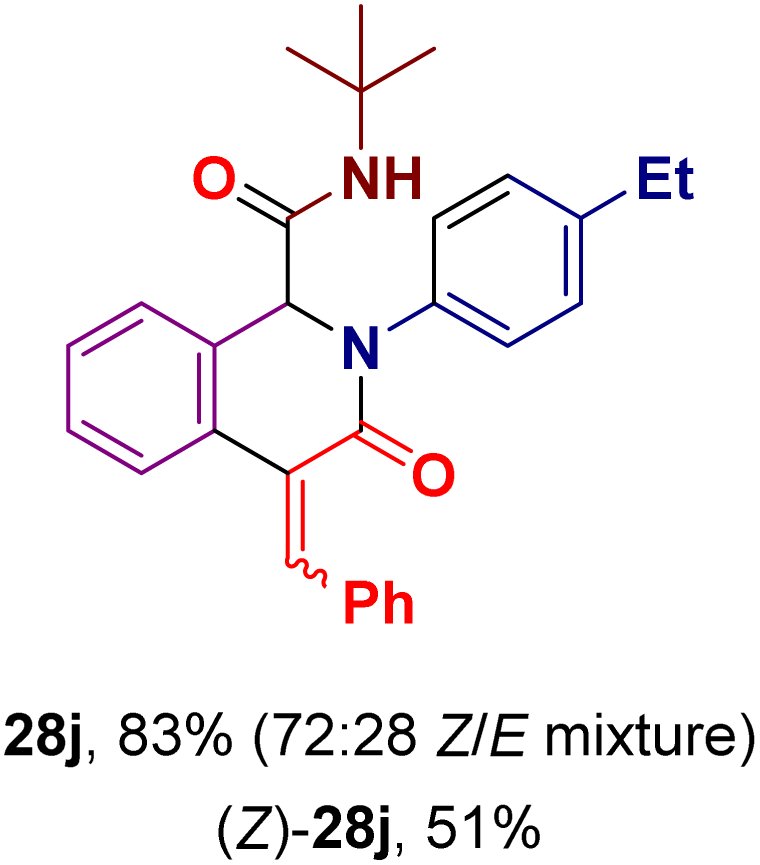	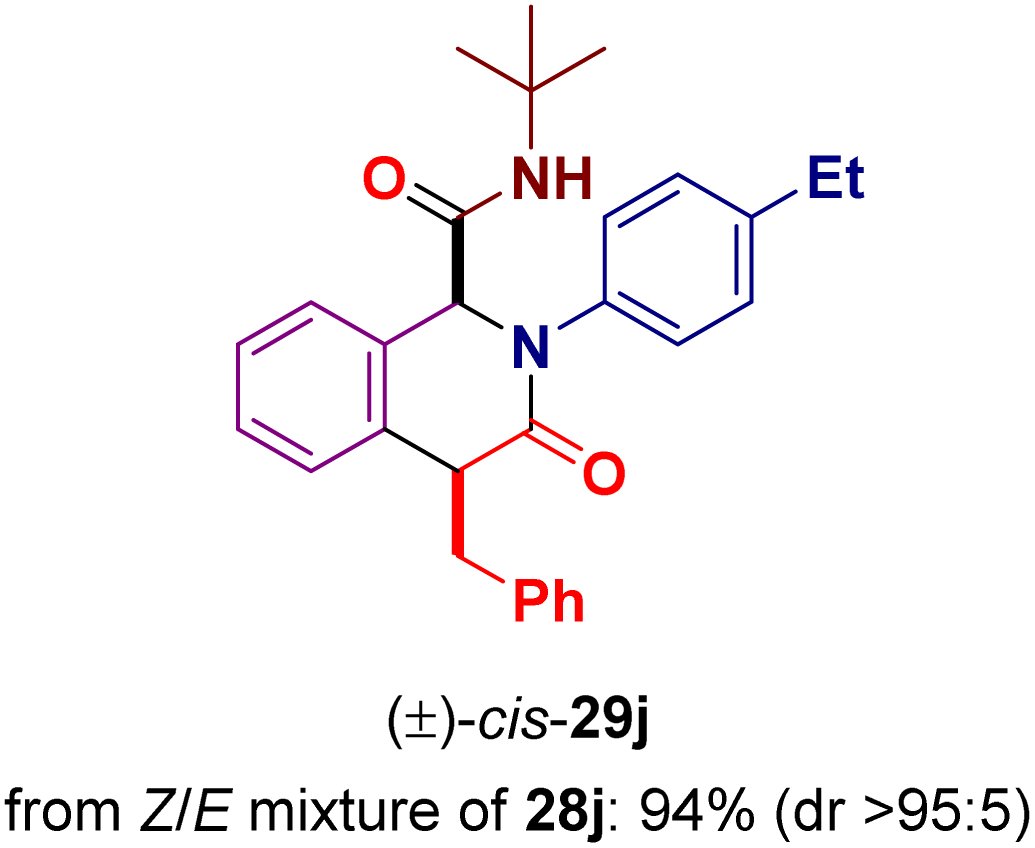
11	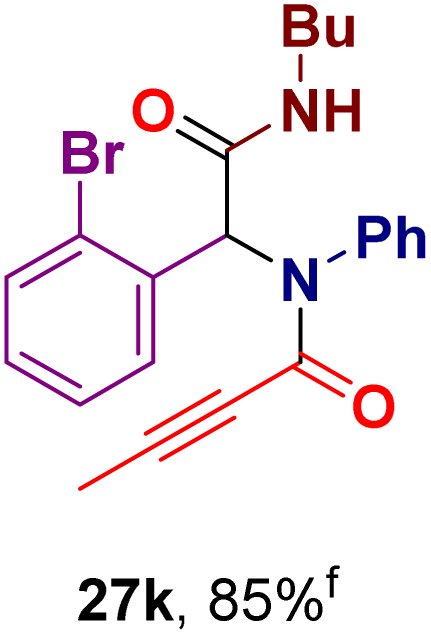	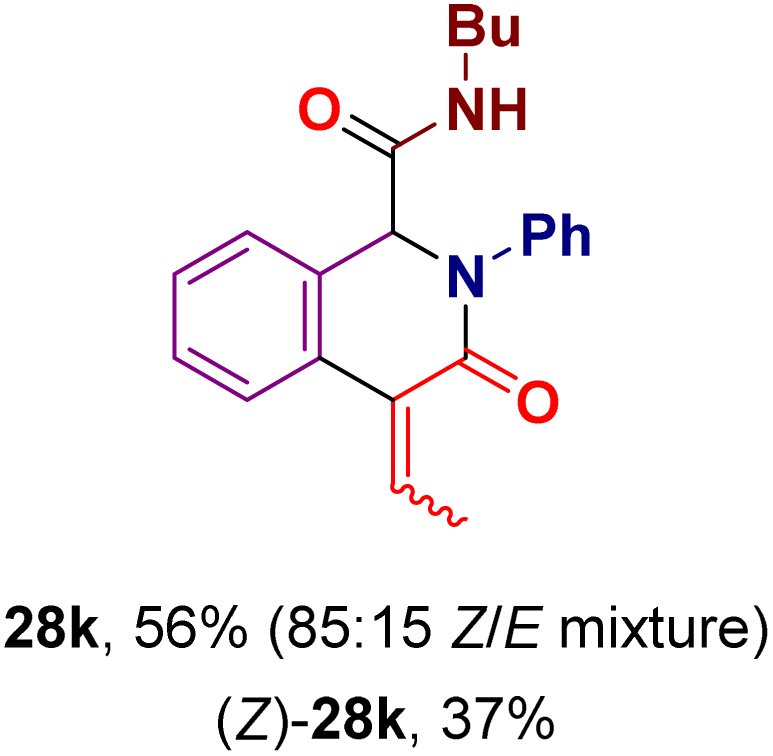	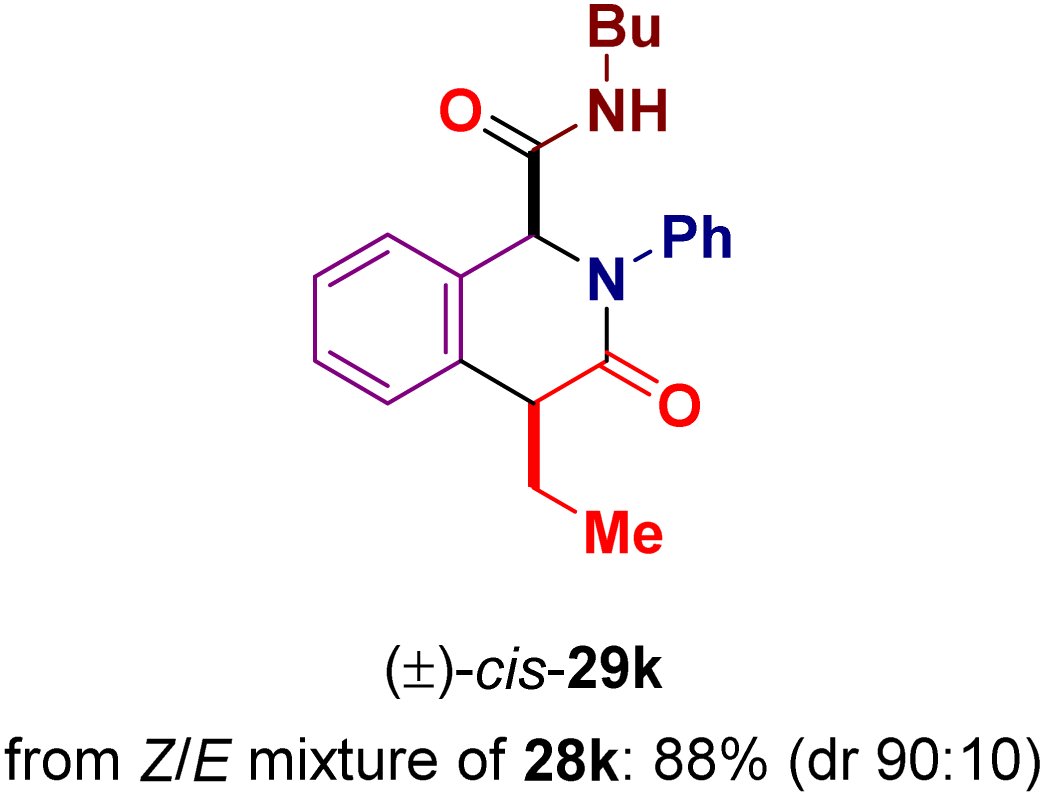
12	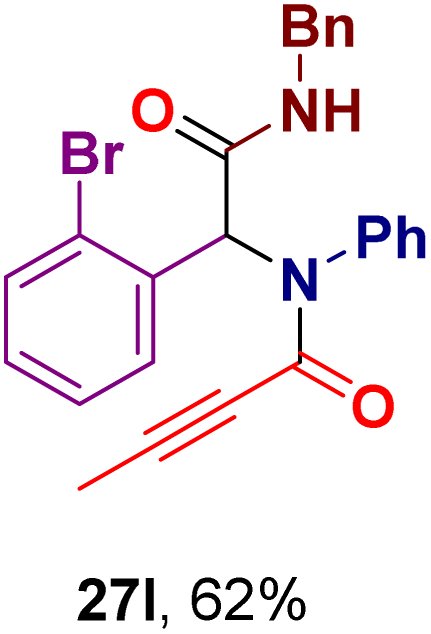	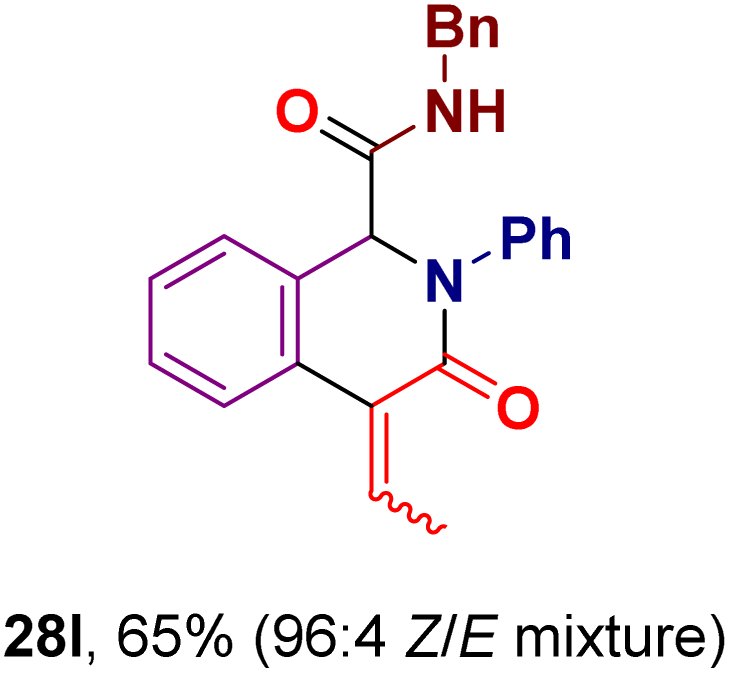	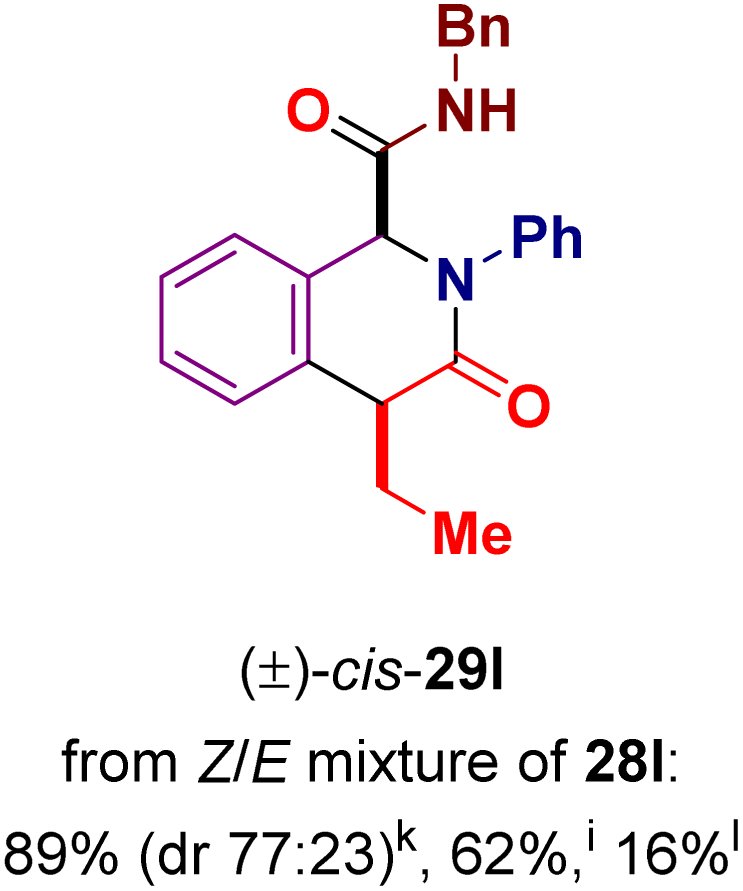

a Unless otherwise specified, the reactions were conducted on 1.5 mmol scale.

b Isolated yields are reported.

c Unless otherwise specified, the reactions were conducted on 0.6–1.0 mmol scale. The *Z*/*E* ratio of products obtained as mixtures of isomers was determined by ^1^H NMR spectroscopy.

d Unless otherwise specified, the reactions were conducted on 0.17–0.8 mmol scale. Unless otherwise specified, the reported *d*_r_ values were determined by ^1^H NMR analysis of the purified products.

e The reaction was conducted on 4.0 mmol scale.

f The reaction was conducted on 3.0 mmol scale.

g The reaction was conducted on 3.0 mmol scale.

h The reaction was conducted on 4.0 mmol scale.

i Isolated yield of the pure *cis* diastereomer.

j The isolated yield based on recovered starting material.

k Overall yield and diastereoselectivity were determined by ^1^H NMR analysis of the crude reaction mixture.

l Isolated yield of the pure *trans* diastereomer.

Before exploring the scope of the reductive Heck reaction, we conducted a brief optimization study using propargylamide 27a as a model substrate. The results revealed only a minor dependence of the reaction outcome on the Pd precatalyst employed but highlighted the importance of using a 3 : 1 DMF/water mixture as the reaction medium (Table S2 in the SI). Subjecting propargylamides 27 to the optimized reductive Heck conditions afforded isoquinolines 28 bearing an exocyclic double bond. As expected from the work of Müller and Riva, this transformation exhibited only modest stereoselectivity with respect to the double-bond configuration, producing 28 predominantly as mixtures of the major *Z* and minor *E* isomers (Table 1). Nevertheless, the reaction proceeded smoothly, affording isoquinolines 28 either as *Z*/*E* mixtures or as single stereoisomers. The structures of several representative *Z* and *E* isomers of isoquinolines 28 were established by single-crystal X-ray diffraction (scXRD) analysis (Fig. 1).

**Fig. 1:**
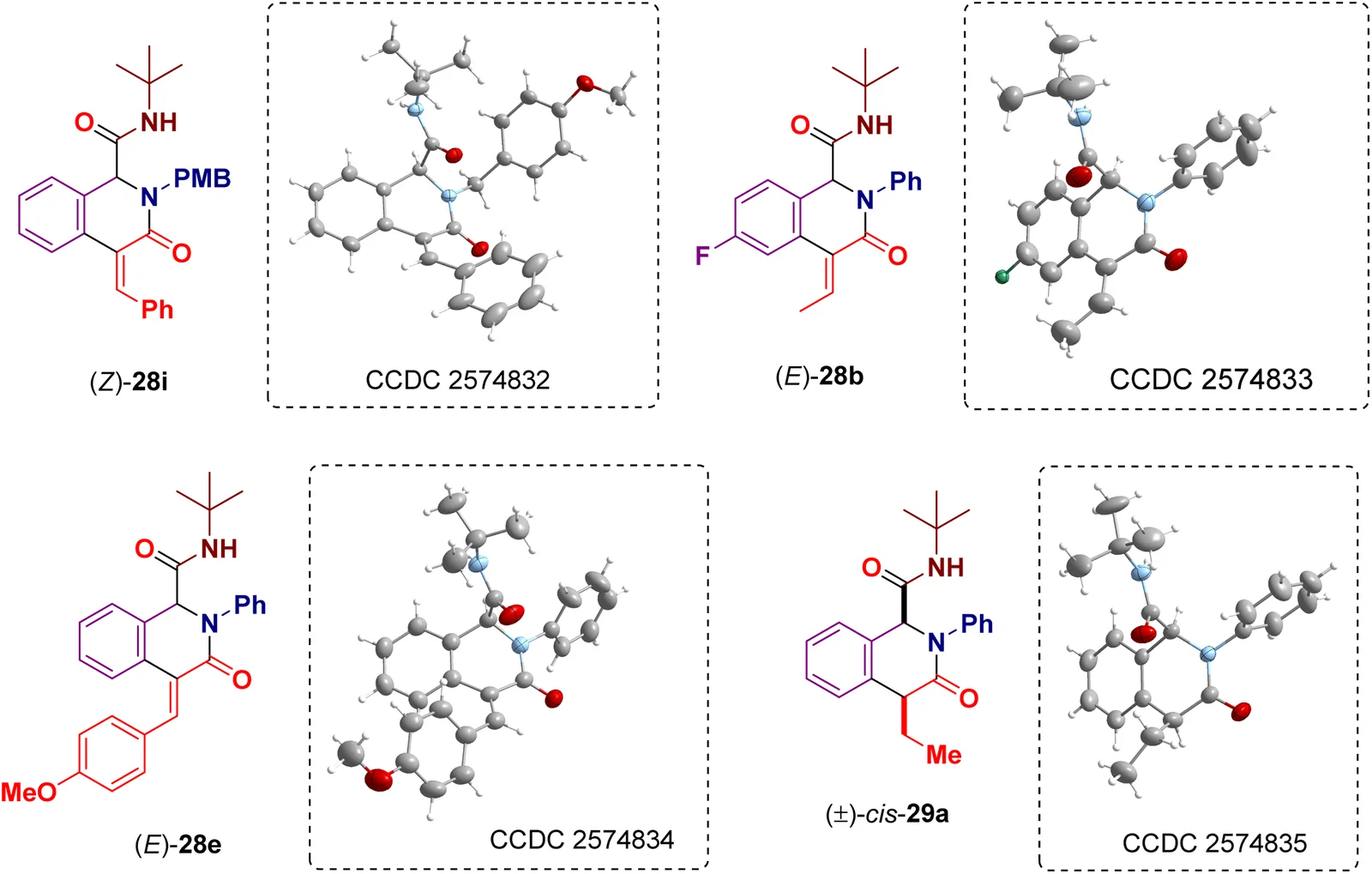
Crystal structures of (*Z*)-28i, (*E*)-28b, (*E*)-28e and *cis*-29a, showing thermal displacement ellipsoids at the 50% probability level.

Finally, Pd-catalyzed double-bond hydrogenation of 28 afforded the target tetrahydroisoquinolines 29 with high diastereoselectivity (Table 1). As expected based on the shielding effect of the isocyanide-derived amide moiety, the major diastereomer of 29 possessed a *cis* relative configuration of the substituents on the tetrahydroisoquinoline core. This assignment was further confirmed by scXRD analysis of representative tetrahydroisoquinoline 29a (Fig. 1). In the case of tetrahydroisoquinoline 29a, we also demonstrated that the *cis* diastereomer was favored regardless of whether the *Z* or *E* isomer of 28a was used as the starting material (Table 1, entry 1). In general, the diastereoselectivity of the hydrogenation was not significantly affected by whether the reaction was performed on *Z*/*E* mixtures or on individual stereoisomers of isoquinolines 28. The study revealed that in most instances the diastereoselectivity of the hydrogenation was slightly lower for substrates bearing (hetero)aryl substituents on the exocyclic double bond than for those bearing a methyl substituent (see *e.g.* entry 1 *versus* entry 4). The presence of a thiophen-3-yl substituent on the exocyclic double bond strongly inhibited the hydrogenation, requiring a higher catalyst loading and a longer reaction time (see Table S3 in SI for details). Nevertheless, the corresponding tetrahydroisoquinoline 29f could still be obtained with high diastereoselectivity, albeit in modest yield (Table 1, entry 6). This outcome is not surprising, considering the well-known poisoning effect of thiophene on Pd catalysts in heterogeneous alkene hydrogenation.^73^ With respect to the isocyanide-derived amide moiety, the presence of a branched *tert*-butyl group resulted in a slightly higher diastereomeric ratio for tetrahydroisoquinolines 29 than that observed for the linear *n*-butyl group (entry 1 *versus* entry 11). This effect is even more pronounced when compared with the substrate derived from benzyl isocyanide, as the corresponding tetrahydroisoquinoline 29m was formed with only moderate diastereocontrol towards the *cis* configuration (entry 12). This trend may reflect the greater steric shielding of one face of the exocyclic double bond by the *tert*-butyl-substituted amide moiety, which could enhance the facial selectivity of hydrogenation and thus favor formation of the *cis* diastereomer.

The scalability of the overall sequence was also assessed through the larger-scale synthesis of the model tetrahydroisoquinoline 29a, in which each step was performed on a 3.0 or 4.0 mmol scale (Table 1, entry 1).

Next, we turned our attention to the post-assembly modification of tetrahydroisoquinoline 29a (Scheme 3). Treatment of 29a with triflic acid in toluene at 100 °C resulted in the removal of the *tert*-butyl group from the exocyclic amide moiety, affording tetrahydroisoquinoline 30 in a good 68% yield. Treatment of 29a with LiAlH_4_ resulted in chemoselective reduction of the cyclic amide, affording tetrahydroisoquinoline 31 in an excellent 98% yield. This transformation could be scaled up to 3.0 mmol without a substantial decrease in yield. As expected, only the cyclic amide functionality was reduced, increasing the saturation of the heterocyclic core, whereas the more sterically hindered exocyclic amide moiety remained intact. These results demonstrate that both amide functionalities present in the parent tetrahydroisoquinoline scaffold are amenable to selective modification.

**Scheme 3:**
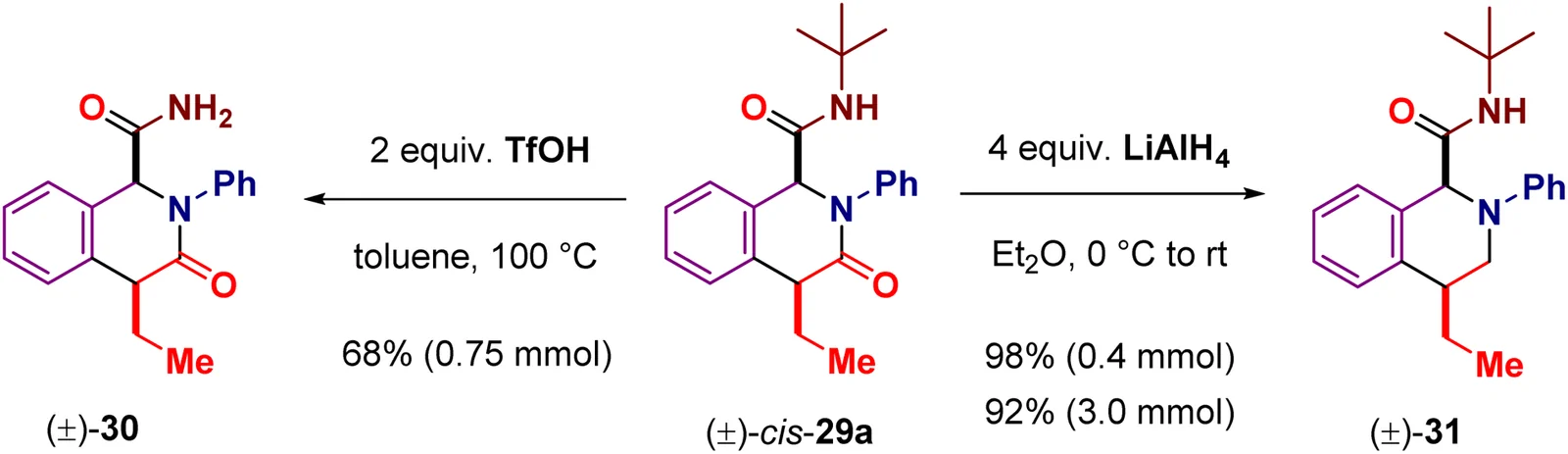
Post-assembly modifications of tetrahydroisoquinoline *cis*-29a.

Subjecting propargylamide 27d to a Heck–Suzuki tandem reaction yielded isoquinoline 33 bearing a fully substituted double bond (Scheme 4). However, its hydrogenation proved to be much less diastereoselective compared to isoquinolines 27 bearing trisubstituted double bond. Both the major *cis* and minor *trans* diastereomers of the resulting tetrahydroisoquinoline 34 were isolated in pure form. The relative configuration of the stereocenters in the minor hydrogenation product *trans*-34 was established by scXRD analysis.

**Scheme 4:**
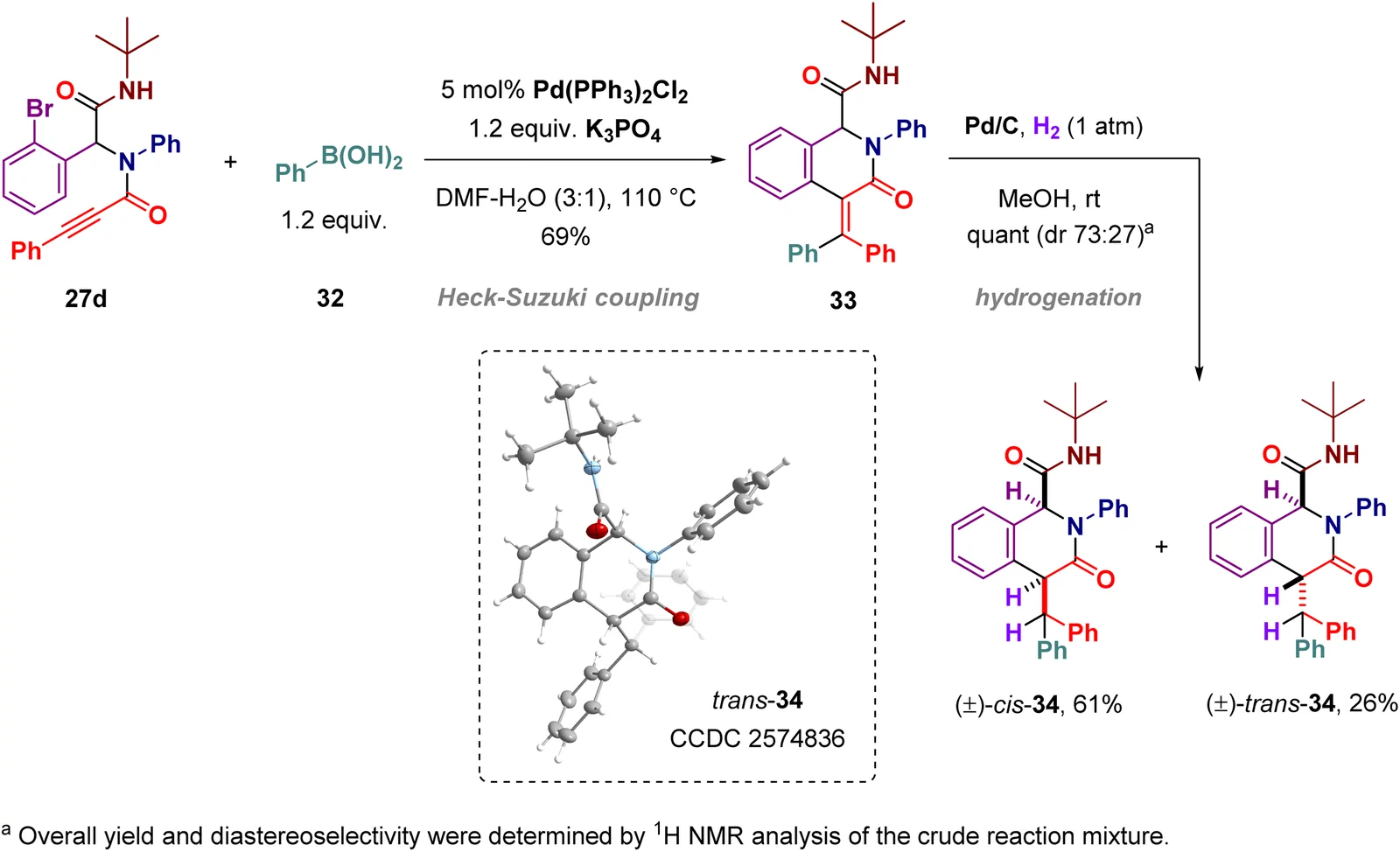
Synthesis of tetrahydroisoquinolines 34.

The use of 2-(2-bromophenyl)acetaldehyde 12 in place of *ortho*-bromobenzaldehydes 26 in the Ugi/reductive Heck sequence afforded 3-benzazepine 36 bearing an exocyclic double bond (Scheme 5). In this case as well, Pd-catalyzed heterogeneous double-bond hydrogenation proceeded with low diastereocontrol. The reduced selectivity may be attributed to the increased conformational flexibility of the seven-membered ring of 3-benzazepine core. Compared with the more conformationally constrained isoquinoline system, the 3-benzazepine can adopt multiple accessible conformations, thereby diminishing the stereochemical bias exerted by the pre-existing stereocenter. Both the *cis* and *trans* diastereomers of the resulting sp^3^-enriched 3-benzazepine 37 were isolated in pure form, and their structures were confirmed by scXRD analysis. The *cis* isomer of 37 crystallized only as a poorly resolved twinned crystal. Consequently, the crystallographic data were of insufficient quality for deposition in the CCDC. Nevertheless, they were sufficient to establish the atom connectivity and the relative configuration of the stereocenters (see Table S5 and Fig. S6 in SI for details).

**Scheme 5:**
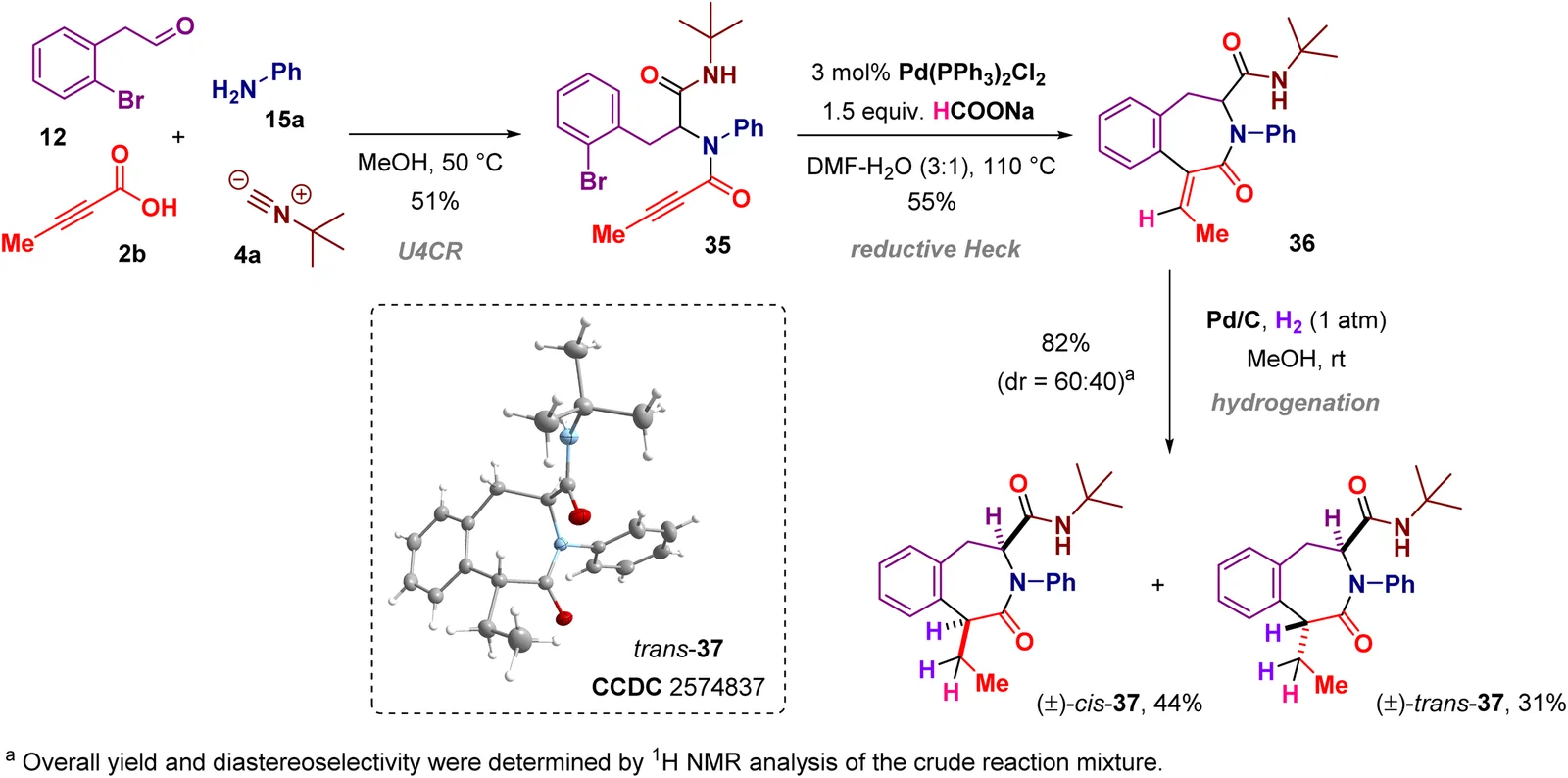
Synthesis of 3-benzazepines 37.

## Conclusions

Overall, we have explored a sequence of palladium-catalyzed post-Ugi transformations involving reductive Heck cyclization and alkene hydrogenation for the synthesis of densely functionalized, sp^3^-enriched tetrahydroisoquinolines. The key advantage of this approach is the efficient transfer of stereochemical information from the Ugi-derived stereocenter to a newly formed stereocenter, enabling predominantly *cis*-selective synthesis. Notably, the hydrogenation proved to be highly stereoconvergent, providing the same major *cis* diastereomer irrespective of the *E*/*Z* geometry of the precursor. The developed strategy complements existing post-Ugi protocols for isoquinoline synthesis^74,75^ while expanding the array of sp^3^-enriched heterocyclic scaffolds accessible through multicomponent and post-MCR chemistry.

## Author contributions

Manzoor Zaman: investigation, validation, methodology, writing – review & editing. Ainara Bekbolatova: investigation, validation, methodology, writing – review & editing. Sofia D. Martynova: investigation, validation, methodology, writing – review & editing. Azamat Begenov: investigation, validation, methodology, writing – review & editing. Niyaz Amire: investigation, validation, methodology, writing – review & editing. Muhammad Hasan: investigation, validation, methodology, writing – review & editing. Azat M. Makhmet: investigation, validation, methodology, writing – review & editing. Rakhmetulla Yerkassov: methodology, supervision, writing – review & editing. Andrey Belyaev: investigation, validation, writing – review & editing. Adilet Bekmagambetov: validation, methodology, writing – review & editing. Davit Hayrapetyan: methodology, supervision, writing – review & editing. Olga P. Pereshivko: conceptualization, methodology, supervision, writing – review & editing. Vsevolod A. Peshkov: conceptualization, methodology, funding acquisition, supervision, writing – original draft. Anatoly A. Peshkov: conceptualization, investigation, validation, methodology, funding acquisition, supervision, writing – original draft.

## Conflicts of interest

There are no conflicts to declare.

## Supplementary Material

RA-016-D6RA06856A-s001

## Data Availability

CCDC 2574832 (for (*Z*)-28i), 2574833 (for (*E*)-28b), 2574834 (for (*E*)-28e), 2574835 (for *cis*-29a), 2574836 (for *trans*-34) and 2574837 (for *trans*-37) contain the supplementary crystallographic data for this paper.^76*a*–*f*^ The data that support the findings of this study are available in the supplementary information (SI) of this article. Supplementary information: descriptions of the experimental procedures, product characterization data and the copies of NMR spectra. See DOI: https://doi.org/10.1039/d6ra06856a.
