# Supplementary material for: Diastereoselective synthesis of tetrahydroisoquinolines through the Pd-catalyzed post-Ugi transformations
Source: RSC Adv. 2026 Oct 1;16(46):59691–9. doi: 10.1039/d6ra06856a (PMC13629751; doi:10.1039/d6ra06856a)

Supplementary Information (SI) for RSC Advances.
This journal is © The Royal Society of Chemistry

Diastereoselective synthesis of tetrahydroisoquinolines through the Pd-catalyzed post-Ugi transformations

Manzoor Zaman,[1,2‡] Ainara Bekbolatova,[3,4‡] Sofia D. Martynova,[3] Azamat Begenov,[3,4] Niyaz Amire,[3,4] Muhammad Hasan,[1,3] Azat M. Makhmet,[3,4,5] Rakhmetulla Yerkassov,[5] Andrey Belyaev,[6] Adilet Bekmagambetov,[3,4] Davit Hayrapetyan,[3] Olga P. Pereshivko,[1,3] Vsevolod A. Peshkov,[1,3,4]* Anatoly A. Peshkov[3,4]*

[1] *Soochow University, College of Chemistry, Chemical Engineering and Materials Science, Dushu Lake Campus, Suzhou, 215123, P. R. China*

[2] *Guangzhou Medical University, Guangzhou Municipal and Guangdong Provincial Key Laboratory of Molecular Target & Clinical Pharmacology, the NMPA and State Key Laboratory of Respiratory Disease, School of Pharmaceutical Sciences, Guangzhou, Guangdong 511436, China*

[3] *Nazarbayev University, Department of Chemistry, School of Sciences and Humanities, Astana, 010000, Kazakhstan*

[4] *National Laboratory Astana, Nazarbayev University, Astana 010000, Kazakhstan*

[5] *L. N. Gumilyov Eurasian National University, Department of Chemistry, Faculty of Natural Sciences, Astana, 010000, Kazakhstan*

[6] *Department of Chemistry, University of Eastern Finland, FI-80101 Joensuu, Finland*

[‡] *Contributed equally*

Table of content

General Remarks	S3
Starting materials used for Ugi reaction	S4
General procedures for the synthesis of Ugi adducts **27**	S6
Screening parameters for the reductive Heck cyclization of Ugi adduct **27a**	S7
General procedures for the synthesis of isoquinoline derivatives **28**	S8
General procedures for the heterogeneous hydrogenation of isoquinoline derivatives **28**	S9
Heterogeneous hydrogenation of isoquinoline derivative **28f**	S11
Procedure for the synthesis of isoquinoline derivative **30**	S12
Procedure for the synthesis of isoquinoline derivative **31**	S13
Procedure for the synthesis of isoquinoline derivative **33**	S13
Procedure for the heterogeneous hydrogenation of isoquinoline derivative **33**	S14
Procedure for the synthesis of Ugi adduct **35**	S15
Procedure for the synthesis of 3-benzazepine derivative **36**	S15
Procedure for the heterogeneous hydrogenation of 3-benzazepine derivative **36**	S16
Characterization of synthesized compounds **27**	S17
Characterization of synthesized compounds **28**	S23
Characterization of synthesized compounds **29**	S32
Characterization of synthesized compounds **30**, **31** and **33**-**37**	S39
X-ray diffraction determination	S44
Copies of ^{1}H and $^{13}C\{^{1}H\}$ spectra	S51

General Remarks

Materials and solvents: All reagents and solvents were purchased from commercial sources and used without further purification.

2-Bromobenzaldehyde (Purity: 97%, CAS Number 6630-33-7) Apollo Scientific
2-Bromo-4-fluorobenzaldehyde (Purity: 98%, CAS Number 59142-68-6) AmBeed
2-Bromo-4-methoxybenzaldehyde (Purity: 98%, CAS Number 43192-31-0) AmBeed
Phenylpropiolic acid (Purity: 99%, CAS Number 637-44-5) Sigma-Aldrich
2-Butynoic acid (Purity: 99%, CAS Number 590-93-2) Apollo Scientific
3-(4-Methoxyphenyl)propiolic acid (Purity: 95%, CAS Number 2227-57-8) AmBeed
3-(Thiophen-3-yl)prop-2-ynoic acid (Purity: 95%, CAS Number 1041178-28-2) AmBeed
Aniline (Purity: 99.8%, CAS Number 62-53-3) Thermo Scientific Chemicals
4-Fluoroaniline (Purity: 99%, CAS Number 371-40-4) Sigma-Aldrich
4-Methoxybenzylamine (Purity: 98%, CAS Number 2393-23-9) Thermo Scientific Chemicals
4-Ethylaniline (Purity: 99%, CAS Number 589-16-2) Macklin
tert-Butyl isocyanide (Purity: 98%, CAS Number 7188-38-7) Sigma-Aldrich
n-Butyl isocyanide (Purity: 98%, CAS Number 2769-64-4) Macklin
Benzyl isocyanide (Purity: 98%, CAS Number 10340-91-7) Sigma-Aldrich
Bis(triphenylphosphine)palladium(II) dichloride (Purity: 98%, CAS Number 13965-03-2) Santa Cruz Biotechnology
Palladium on carbon (10 wt. % loading, Catalog Number 205699) Sigma-Aldrich
Palladium on carbon (5 wt. % loading, Catalog Number 205680) Sigma-Aldrich
Lithium aluminum hydride (Purity: 95%, CAS Number 16853-85-3) Sigma-Aldrich
Sodium formate (Purity: 99%, CAS Number 141-53-7) Sigma-Aldrich
Potassium phosphate (Purity: 90+%, CAS Number 7778-53-2) Thermo Scientific Chemicals
Phenylboronic acid (Purity: 98%, CAS Number 98-80-6) AmBeed
Trifluoromethanesulfonic acid (Purity: 99%, CAS Number 1493-13-6) Thermo Scientific Chemicals
Methanol (Purity: 99.9%, HPLC grade) Sigma-Aldrich
Hexane (Purity: 97.0%, HPLC grade) Sigma-Aldrich
Ethyl Acetate (Purity: 99.7%, HPLC grade) Sigma-Aldrich
Dichloromethane (Purity: 99.9%, ACS Reagent grade) Sigma-Aldrich
Pentane (Purity: 99.0%, Spectrophotometric grade) Sigma-Aldrich
Diethyl Ether (Purity: 99.8%, ACS Reagent grade) Sigma-Aldrich
Anhydrous Diethyl Ether (Purity: 99.7%, Catalog Number 296082) Sigma-Aldrich
Dimethyl Formamide (Purity: 99.8%, ACS Reagent grade) Sigma-Aldrich

Toluene (Purity: 99.7%, ACS Reagent grade) Sigma-Aldrich
Anhydrous toluene was obtained by drying over 3Å molecular sieves

Silicone oil bath was used for all of the reactions that required heating. Column chromatography was carried out with silica gel grade 60 (0.040–0.063 mm) 230–400 mesh. TLC was performed with Macherey-Nagel ≪Alugram Sil G/UV254≫ plates.

NMR spectroscopy: NMR spectroscopic data were recorded with a JEOL JNM-ECA 500 MHz spectrometer (500.16 MHz for ^{1}H and 125.78 MHz for ^{13}C{^{1}H}), a Bruker Avance III 400 MHz spectrometer (400.13 MHz for ^{1}H and 100.61 MHz for ^{13}C{^{1}H}) and with a Bruker Avance 600 MHz spectrometer (150.84 MHz for ^{13}C{^{1}H}), in DMSO-*D*6 or $CDCl_3$ and were referenced to solvent residual proton signal (δH = 2.50 and 7.26 ppm, respectively) or to TMS signal (δH = 0 ppm) and solvent carbon signal (δC = 39.52 and 77.16 ppm, respectively).

Mass spectrometry: Mass spectra were recorded on Agilent 6530 ESI-QTOF mass spectrometer equipped with UHPLC inlet and UV detector or by using BRUKER microTOF-Q III instrument with ESI source or by using BRUKER liquid chromatography mass spectrometry system - Impact II VIP.

Melting points: Melting points were determined in open capillary tubes using a Stuart Scientific SMP3 or an INESA WRR melting point apparatus.

Table S1. Starting materials used for Ugi reaction

Aldehydes	Alkynoic acids	Amines	Isocyanides
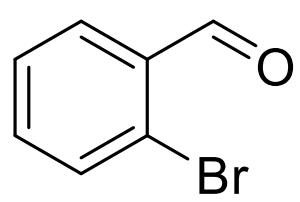 **26a**	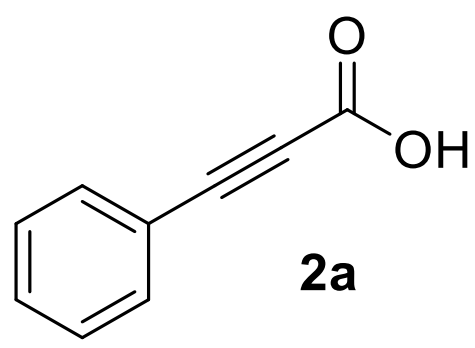 **2a**	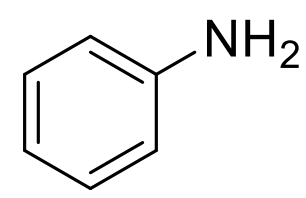 **15a**	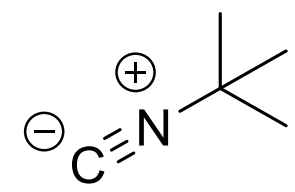 **4a**
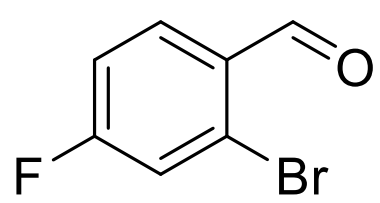 **26b**	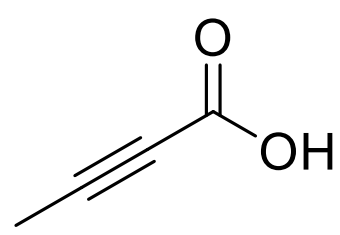 **2b**	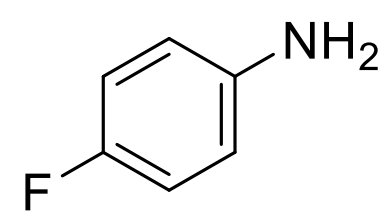 **15b**	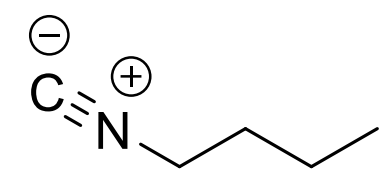 **4b**
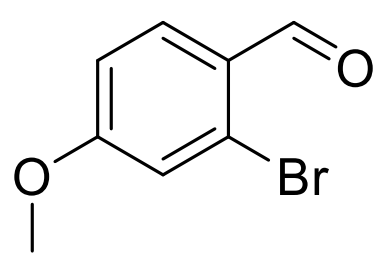 **26c**	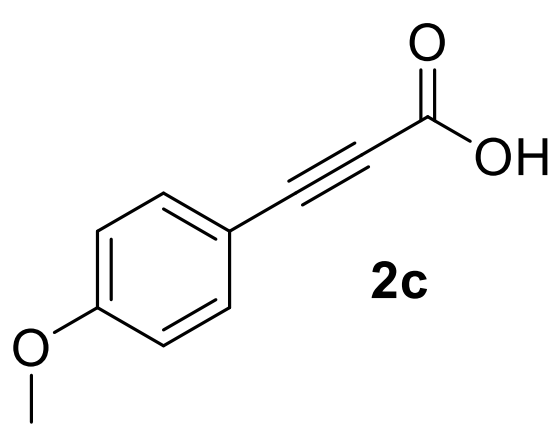 **2c**	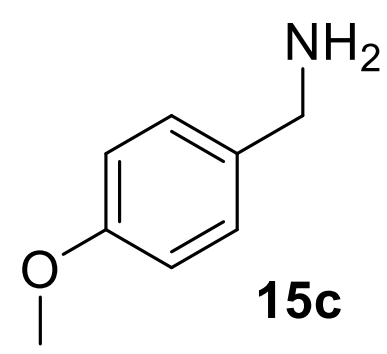 **15c**	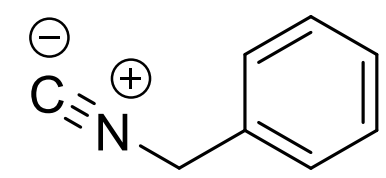 **4c**
	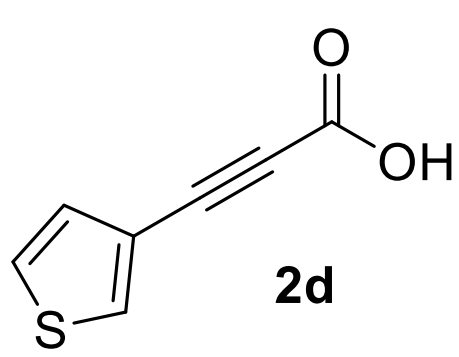 **2d**	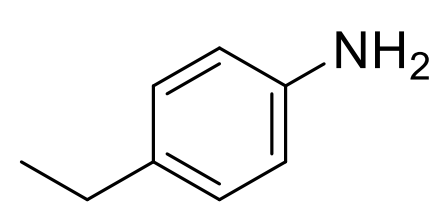 **15d**	

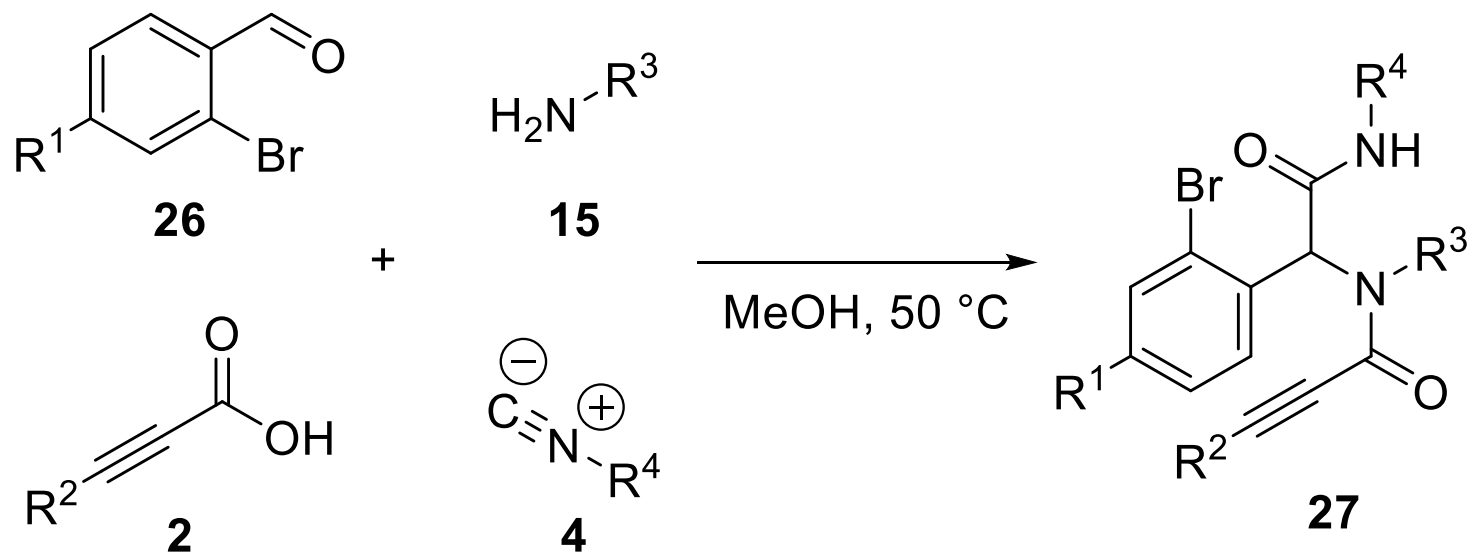

General procedure for the synthesis of Ugi adducts 27a-27g, 27i and 27l

3-Substituted propiolic acid (1.1 equiv., 1.65 mmol) was placed in a screw-cap vial equipped with a magnetic stir bar, followed by the addition of methanol (7.5 mL), the appropriate 2-bromobenzaldehyde (1.0 equiv., 1.5 mmol), amine (1.1 equiv., 1.65 mmol), and isocyanide (1.1 equiv., 1.65 mmol). The vial was sealed and the reaction mixture was stirred at 50 °C for 48 h. Upon completion of the reaction, the magnetic stir bar was removed and, unless otherwise stated, the reaction mixture was diluted with ethyl acetate, and silica gel was added. The solvents were removed under reduced pressure using a rotary evaporator (water bath temperature 40 °C; pressure gradually reduced from 200 mbar to 1–5 mbar). The residue was purified by column chromatography on silica gel using hexane/ethyl acetate as the eluent, followed by trituration with pentane, to afford the corresponding Ugi adducts **27** in pure form.

General procedure for the synthesis of Ugi adducts 27h, 27j and 27k

3-Substituted propiolic acid (1.1 equiv., 3.3 mmol) was placed in a screw-cap vial equipped with a magnetic stir bar, followed by the addition of methanol (15 mL), 2-bromobenzaldehyde (1.0 equiv., 3.0 mmol, 555 mg), the appropriate amine (1.1 equiv., 3.3 mmol), and isocyanide (1.1 equiv., 3.3 mmol). The vial was sealed and the reaction mixture was stirred at 50 °C for 48 h. Upon completion of the reaction, the magnetic stir bar was removed, the mixture was diluted with ethyl acetate, and silica gel was added. The solvents were removed under reduced pressure using a rotary evaporator (water bath temperature 40 °C; pressure gradually reduced from 200 mbar to 1–5 mbar). The residue was purified by column chromatography on silica gel using hexane/ethyl acetate as the eluent to afford the corresponding Ugi adducts **27** in pure form.

Procedure for the larger-scale synthesis of Ugi adduct 27a

But-2-ynoic acid (1.1 equiv., 4.4 mmol, 370 mg) was placed in a screw-cap vial equipped with a magnetic stir bar, followed by the addition of methanol (20 mL), 2-bromobenzaldehyde (1.0 equiv., 4.0 mmol, 740 mg), aniline (1.1 equiv., 4.4 mmol, 410 mg), and *tert*-butyl isocyanate isocyanide (1.1 equiv., 4.4 mmol, 365 mg). The vial was sealed and the reaction mixture was stirred at 50 °C for 48

h. Upon completion of the reaction, the magnetic stir bar was removed, the mixture was diluted with ethyl acetate, and silica gel was added. The solvents were removed under reduced pressure using a rotary evaporator (water bath temperature 40 °C; pressure gradually reduced from 200 mbar to 1–5 mbar). The residue was purified by column chromatography on silica gel using hexane/ethyl acetate as the eluent, followed by trituration with pentane, to afford the corresponding Ugi adduct **27a** in pure form.

Table S2. Screening parameters for the reductive Heck cyclization of Ugi adduct **27a**[a]

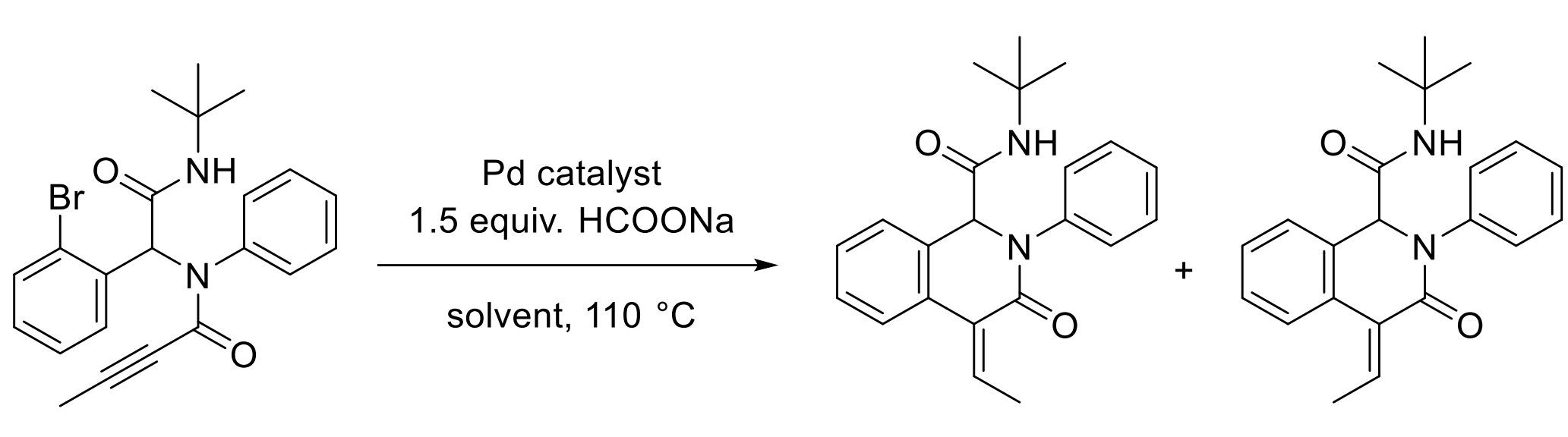

Entry	Pd catalyst	Solvent	Yield, %[b]	
			(*Z*)-**28a**	(*E*)-**28a**
1	3 mol% $Pd(PPh_3)_2Cl_2$	$DMF-H_2O$ (3:1)	52	6
2	3 mol% $Pd(OAc)_2$/6 mol% PPh_3	$DMF-H_2O$ (3:1)	48	6
3	3 mol% $Pd_2(dba)_3$/6 mol% PPh_3	$DMF-H_2O$ (3:1)	48	5
4	3 mol% $Pd(PPh_3)_4$	$DMF-H_2O$ (3:1)	51	6
5	3 mol% $Pd(PPh_3)_2Cl_2$	$DMF-H_2O$ (2:1)	42	4
6[c]	3 mol% $Pd(PPh_3)_2Cl_2$	$DMF-H_2O$ (1:1)	28	2
7[d]	3 mol% $Pd(PPh_3)_2Cl_2$	DMF	40	2

[a] All reactions were conducted on 0.1 mmol scale for 1 hour.
[b] Determined by ^1H NMR of crude reaction mixture after the aqueous workup using 3,4,5-trimethoxybenzaldehyde as internal standard.
[c] Only 78% conversion of **27a** is achieved.
[d] Only 85% conversion of **27a** is achieved.

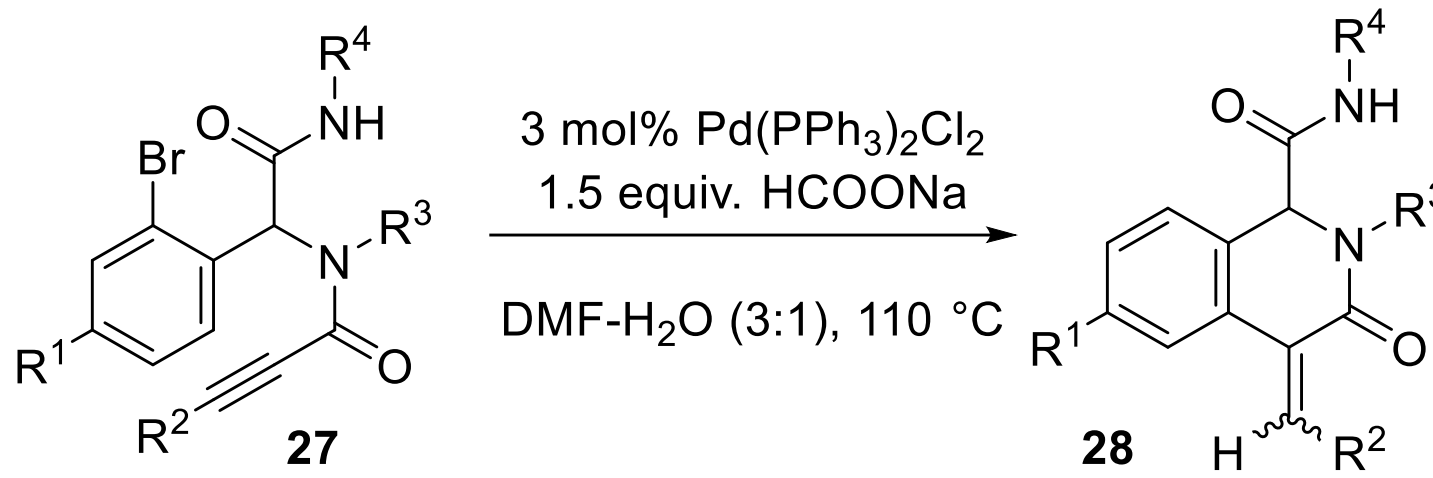

General procedure for the synthesis of isoquinoline derivatives 28a-28g, 28i and 28l

Bis(triphenylphosphine)palladium(II) chloride (3 mol%) was placed in a screw-cap vial equipped with a magnetic stir bar. A 3:1 mixture of dimethylformamide and distilled water was added, followed by the appropriate propargylamide **27** (1.0 equiv.) to give a 0.15 M solution. Sodium formate (1.5 equiv.) was then added, and the reaction mixture was purged with argon. The vial was sealed, and the resulting mixture was stirred at 110 °C for 1 h. Upon completion of the reaction, the mixture was cooled to room temperature and the magnetic stir bar was removed. The reaction mixture was transferred to a separatory funnel, diluted with ethyl acetate (20 mL per 1 mL of used dimethylformamide), and washed with distilled water (3 × 20 mL per 1 mL of used dimethylformamide). The organic layer was separated, dried over anhydrous sodium sulfate, and filtered. Silica gel was then added, and the solvents were removed under reduced pressure using a rotary evaporator (water bath temperature 40 °C; pressure gradually reduced from 200 mbar to 1–5 mbar). The residue was purified by column chromatography on silica gel using hexane/ethyl acetate as the eluent, followed by trituration with diethyl ether, to afford corresponding isoquinoline derivatives **28**.

General procedure for the synthesis of isoquinoline derivatives 28h, 28j and 28k

Propargylamide **27** (1.0 equiv.) and sodium formate (1.5 equiv.) were placed in a screw-cap vial equipped with a magnetic stir bar. A 3:1 mixture of dimethylformamide and distilled water was added to give a 0.2 M solution. Bis(triphenylphosphine)palladium(II) chloride (3 mol%) was then added, and the reaction mixture was purged with argon. The vial was sealed, and the resulting mixture was stirred at 110 °C for 1 h. Upon completion of the reaction, the mixture was cooled to room temperature and the magnetic stir bar was removed. The reaction mixture was transferred to a separatory funnel, diluted with ethyl acetate (20 mL per 1 mL of used dimethylformamide), and washed with distilled water (3 × 20 mL per 1 mL of used dimethylformamide). The organic layer was separated, dried over anhydrous sodium sulfate, and filtered. Silica gel was then added, and the solvents were removed under reduced pressure using a rotary evaporator (water bath temperature 40 °C; pressure gradually reduced from 200 mbar to 1–5 mbar). The residue was purified by column

chromatography on silica gel using hexane/ethyl acetate as the eluent to afford the corresponding isoquinoline derivatives **28**.

Procedure for the larger-scale synthesis of isoquinoline derivative 28a

Bis(triphenylphosphine)palladium(II) chloride (3 mol%, 64 mg) was placed in a screw-cap vial equipped with a magnetic stir bar. Dimethylformamide (15 mL) and distilled water (5 mL) were added, followed by *N*-(1-(2-bromophenyl)-2-(*tert*-butylamino)-2-oxoethyl)-*N*-phenylbut-2-ynamide (**27a**, 1.0 equiv., 3.05 mmol, 1303 mg). Sodium formate (1.5 equiv., 4.58 mmol, 311 mg) was then added, and the reaction mixture was purged with argon. The vial was sealed, and the resulting mixture was stirred at 110 °C for 1 h. Upon completion of the reaction, the mixture was cooled to room temperature and the magnetic stir bar was removed. The reaction mixture was transferred to a separatory funnel, diluted with ethyl acetate (200 mL), and washed with distilled water (3 × 200 mL). The organic layer was separated, dried over anhydrous sodium sulfate, and filtered. Silica gel was then added, and the solvents were removed under reduced pressure using a rotary evaporator (water bath temperature 40 °C; pressure gradually reduced from 200 mbar to 1–5 mbar). The residue was purified by column chromatography on silica gel using hexane/ethyl acetate as the eluent to afford the corresponding isoquinoline derivative **28a**.

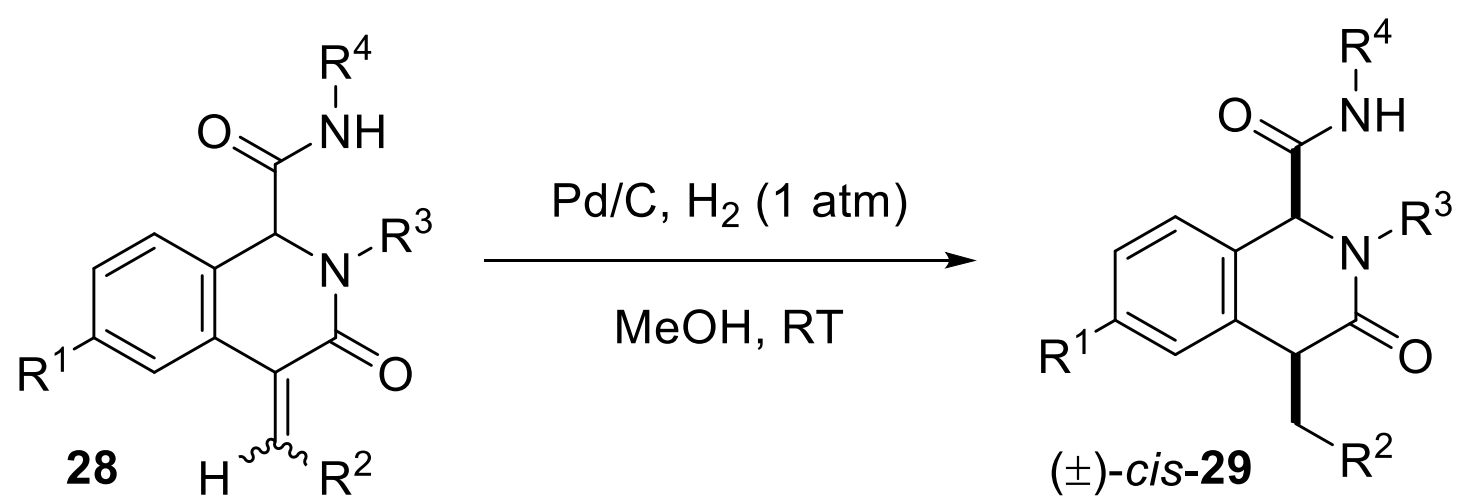

General procedure for the heterogeneous hydrogenation of isoquinoline derivatives 28a, 28b and 28d (Procedure A)

The screw-cap vial containing **28** was equipped with a magnetic stir bar and charged with methanol to give a 0.135 M solution. The vial was sealed with a septum and the solution was purged with argon, after which Pd/C (5 mol%, 5 wt % Pd) was added. The resulting suspension was sequentially flushed with argon and hydrogen, then stirred under a hydrogen atmosphere (1 atm) at room temperature for 24 hours. After completion, the reaction mixture was diluted with dichloromethane and filtered through a glass filter with extra-fine porosity to remove the Pd catalyst. The filtrate was concentrated and dried under reduced pressure using a rotary evaporator (water bath temperature 40 °C; pressure gradually reduced from 700 mbar to 1–5 mbar) to obtain crude product, which was subsequently triturated with diethyl ether to give pure **29**.

General procedure for the heterogeneous hydrogenation of isoquinoline derivatives 28c, 28e, 29g, 28i and 28l (Procedure B)

The screw-cap vial containing **28** was equipped with a magnetic stir bar and charged with methanol to give a 0.02 M solution. The vial was sealed with a septum and the solution was purged with argon, after which Pd/C (10 mol%, 10 wt % Pd) was added. The resulting suspension was sequentially flushed with argon and hydrogen, then stirred under a hydrogen atmosphere (1 atm) at room temperature for 24 hours. After completion, the reaction mixture was diluted with dichloromethane and filtered through a glass filter with extra-fine porosity to remove the Pd catalyst. The filtrate was concentrated and dried under reduced pressure using a rotary evaporator (water bath temperature 40 °C; pressure gradually reduced from 700 mbar to 1–5 mbar) to obtain crude product, which was subsequently triturated with diethyl ether or purified by column chromatography on silica gel using dichloromethane/ethyl acetate as the eluent to give pure **29**.

General procedure for the heterogeneous hydrogenation of isoquinoline derivatives 28b, 28e, 28g, 28h, 28i, 28j and 28k (Procedure C)

The screw-cap vial containing **28** was equipped with a magnetic stir bar and charged with methanol to give a 0.02 M solution. The vial was sealed with a septum and the solution was purged with argon, after which Pd/C (5 mol%, 10 wt % Pd) was added. The resulting suspension was sequentially flushed with argon and hydrogen, then stirred under a hydrogen atmosphere (1 atm) at room temperature for 24 hours. After completion, the resulting mixture was passed through a short silica gel column using ethyl acetate as the eluent to remove the Pd catalyst. The filtrate was concentrated and dried under reduced pressure using a rotary evaporator (water bath temperature 40 °C; pressure gradually reduced from 700 mbar to 1–5 mbar) to obtain pure **29**.

Procedure for the larger-scale heterogeneous hydrogenation of isoquinoline derivative 28a (Procedure D)

The screw-cap vial containing **28a** (4.0 mmol, 1394 mg) was equipped with a magnetic stir bar and charged with methanol (40 mL). The vial was sealed with a septum and the solution was purged with argon, after which Pd/C (10 mol%, 0.4 mmol, 424 mg of 10 wt % Pd) was added. The resulting suspension was sequentially flushed with argon and hydrogen, then stirred under a hydrogen atmosphere (1 atm) at room temperature for 24 hours. After completion, the reaction mixture was diluted with dichloromethane and filtered through a glass filter with extra-fine porosity to remove the Pd catalyst. The filtrate was concentrated and dried under reduced pressure using a rotary

evaporator (water bath temperature 40 °C; pressure gradually reduced from 700 mbar to 1–5 mbar) to obtain crude product, which was subsequently triturated with diethyl ether to give pure **29a**.

Table S3. Screening parameters for the heterogeneous hydrogenation of isoquinoline derivative **28f**[a]

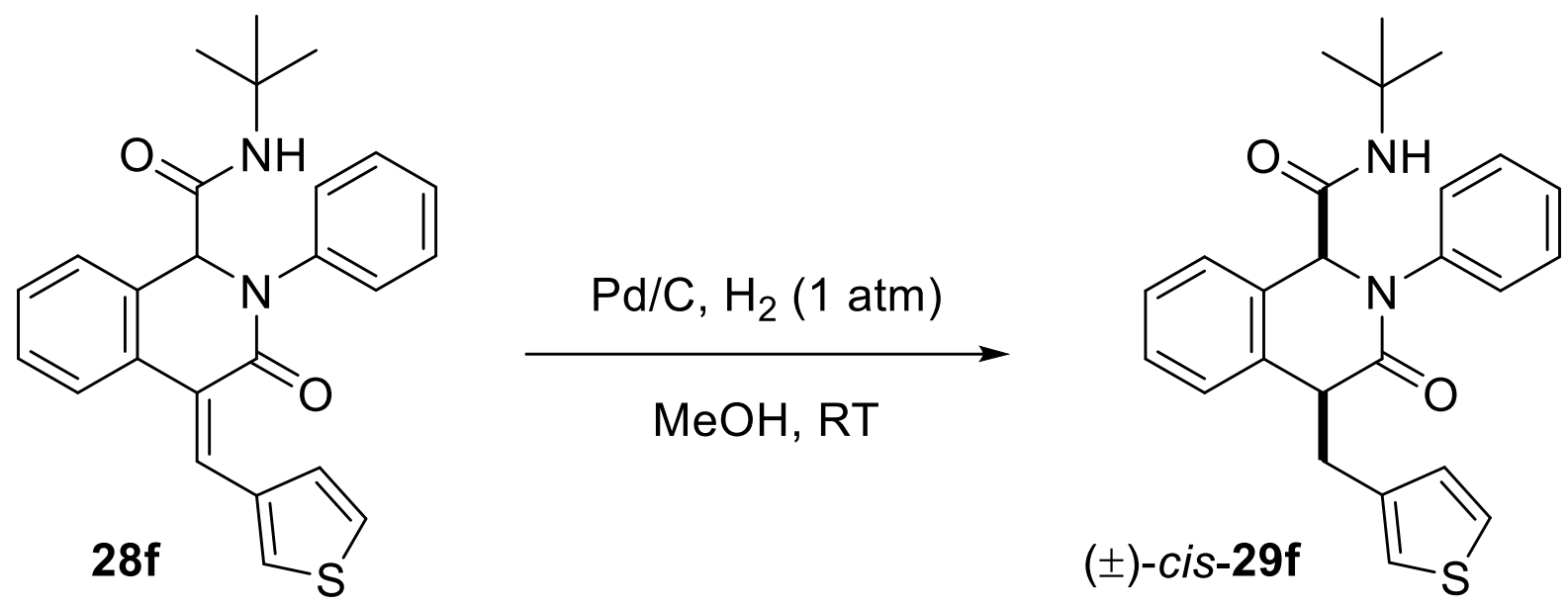

Starting material	Catalyst	Conditions	Yield, %[b]		
			(*E*)-**28f**	(*Z*)-**28f**	*cis*-**29f**
(*E*)-**28**	Pd/C (30 mol%, 10 wt % Pd)	MeOH (0.02 M), 7 days	93	traces	7
(*Z*)-**28f**	Pd/C (30 mol%, 10 wt % Pd)	MeOH (0.02 M), 7 days	9	59	19
(*Z*)-**28f**	Pd/C (30 mol%, 10 wt % Pd)	MeOH (0.02 M), 7 days	9	41	21
(*Z*)-**28f**[c]	Pd/C (5 mol%, 5 wt % Pd), then Pd/C (20 mol%, 5 wt % Pd)	MeOH (0.135 M), 1 day, then MeOH (0.045 M), 6 days	7	46[d]	31 (dr 93:7)[e]

[a] Unless otherwise specified, all reactions were conducted on 0.1 mmol scale.
[b] Unless otherwise specified, determined by ^{1}H NMR of crude reaction mixture after removal of Pd catalyst by filtration using 3,4,5-trimethoxybenzaldehyde as internal standard.
[c] Conducted on 0.36 mmol scale.
[d] Amount recovered after column chromatography.
[e] Isolated yield and dr of the isolated sample.

Highlights of the screening:

- The presence of a thiophen-3-yl substituent on the exocyclic double bond strongly inhibited the heterogeneous hydrogenation.

- No full conversion of starting isoquinoline derivative **28f** can be achieved even using a higher catalyst loading and a longer reaction time.
- Conducting the reaction with the *Z* isomer of **28f** led to higher conversion than with the *E* isomer. However, it was also accompanied by substantial double-bond isomerization and substrate degradation.

Procedure for the heterogeneous hydrogenation of isoquinoline derivative 28f (Procedure E)

The screw-cap vial containing **28f** (0.36 mmol, 150 mg) was equipped with a magnetic stir bar and charged with methanol (2.7 mL). The vial was sealed with a septum, and the solution was purged with argon before Pd/C (5 mol%, 5 wt% Pd, 38 mg) was added. The resulting suspension was sequentially flushed with argon and hydrogen and then stirred under a hydrogen atmosphere (1 atm) at room temperature for 24 h. Subsequently, a second portion of Pd/C (15 mol%, 5 wt% Pd, 115 mg) and methanol (5.4 mL) was added, and the reaction mixture was stirred under a hydrogen atmosphere at room temperature for an additional 144 h. The reaction mixture was then diluted with dichloromethane and filtered through an extra-fine porosity glass frit to remove the catalyst. The filtrate was concentrated under reduced pressure using a rotary evaporator (water bath temperature 40 °C; pressure gradually reduced from 700 mbar to 1–5 mbar) to afford the crude product. Purification by column chromatography on silica gel using hexane/ethyl acetate as the eluent afforded the corresponding hydrogenated product **29f** and recovered starting material **28f**.

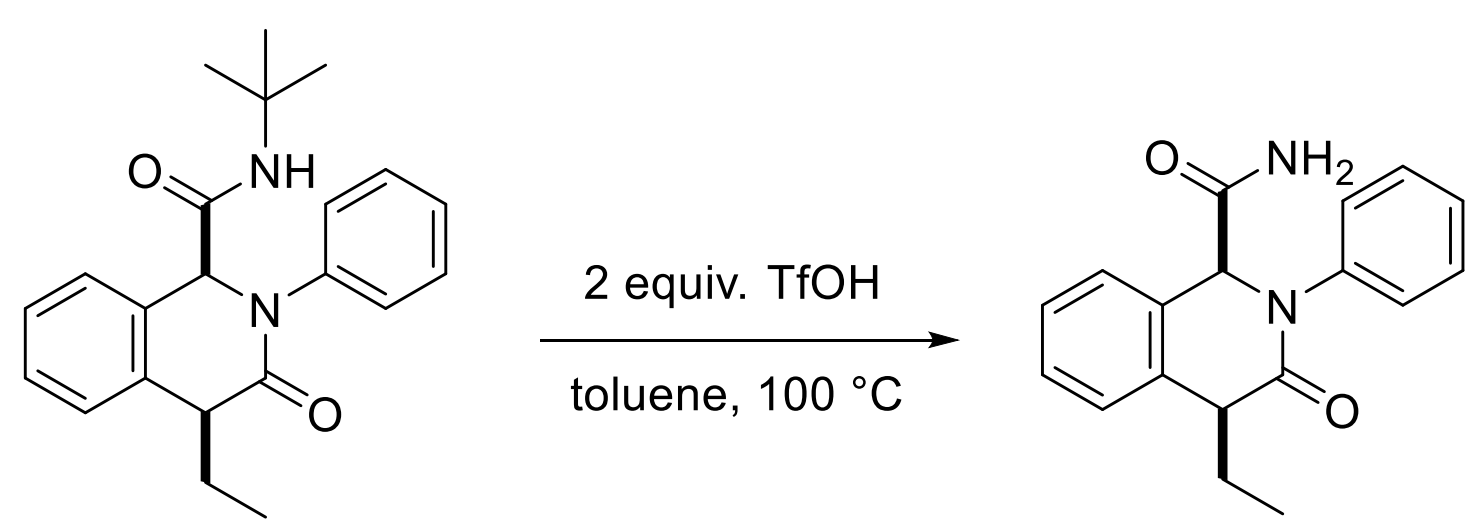

(±)-*cis*-**29a** (±)-**30**

Procedure for the synthesis of isoquinoline derivative 30

(±)-(1*S*,4*S*)-*N*-(*tert*-Butyl)-4-ethyl-3-oxo-2-phenyl-1,2,3,4-tetrahydroisoquinoline-1-carboxamide (**29a**, 1.0 equiv., 0.75 mmol, 263 mg) was placed in a screw-cap vial equipped with a magnetic stir bar, followed by the addition of anhydrous toluene (6 mL). The resulting suspension was flushed with argon, and trifluoromethanesulfonic acid (2.0 equiv., 1.5 mmol, 133 µL) was added. The reaction mixture was flushed with argon once more, the vial was sealed, and the mixture was stirred at 100 °C for 17 h. After completion of the reaction, the mixture was cooled to room temperature

and filtered through a glass filter. The collected solids were washed with diethyl ether to afford **30** as a pure product.

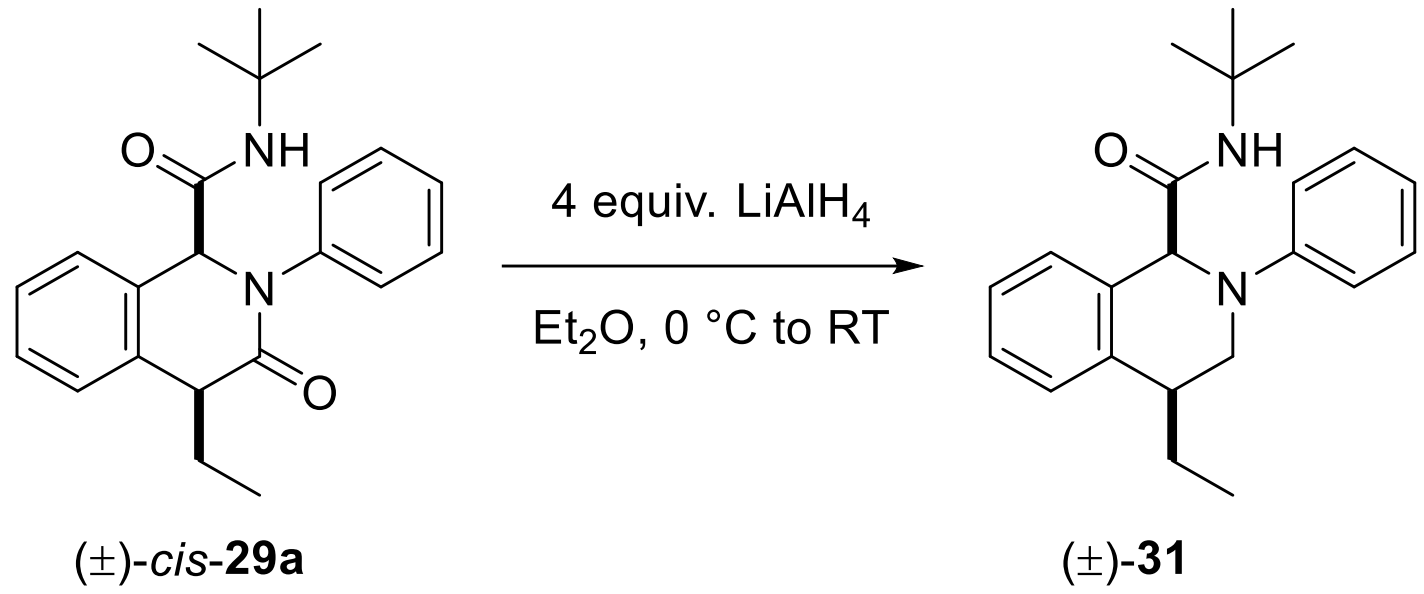

Procedure for the synthesis of isoquinoline derivative 31

(±)-(1*S*,4*S*)-*N*-(*tert*-Butyl)-4-ethyl-3-oxo-2-phenyl-1,2,3,4-tetrahydroisoquinoline-1-carboxamide (**29a**, 1 equiv., 3 mmol, 1051 mg) was placed in a screw cap vial and suspended in anhydrous diethyl ether (20 mL) followed by addition of lithium aluminum hydride (4.0 equiv., 12 mmol, 455 mg,) in one portion at 0 °C. The reaction vial was purged with argon, sealed, and removed from the ice bath. The reaction mixture was stirred at room temperature for 24 hours and then subjected to a Fieser workup. The reaction mixture was cooled down to 0 °C. Then water (455 µL) was carefully added followed by slow addition of 15% aqueous sodium hydroxide solution (455 µL). Then, the second portion of water (1365 µL) was added and the mixture was allowed to warm up to room temperature under stirring for approximately 15 minutes. The resulting mixture was dried from water with anhydrous sodium sulfate. Solids were filtered off and washed with diethyl ether. The filtrate was concentrated under reduced pressure and subsequently triturated with diethyl ether to deliver pure **31**.

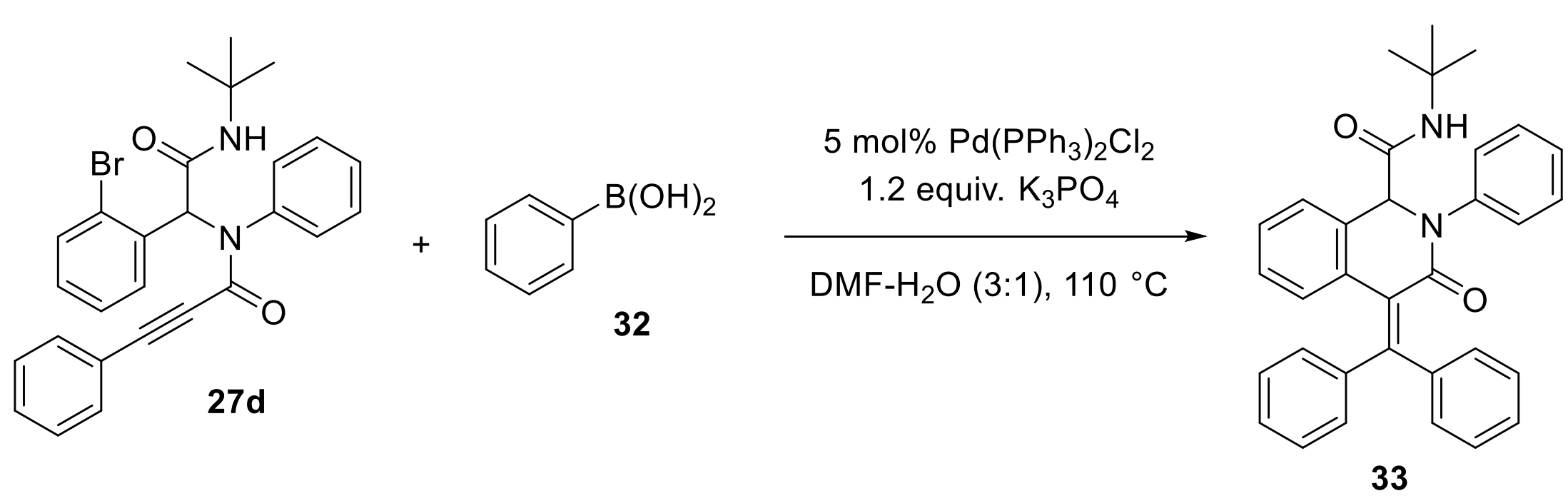

Procedure for the synthesis of isoquinoline derivative 33

Bis(triphenylphosphine)palladium(II) chloride (3 mol%, 0.027 mmol, 19 mg) was placed in a screw-cap vial equipped with a magnetic stir bar, followed by the addition of dimethylformamide (DMF,

6.75 mL) and distilled water (2.25 mL). *N*-(1-(2-Bromophenyl)-2-(*tert*-butylamino)-2-oxoethyl)-*N*,3-diphenylpropiolamide (**9d**, 1.0 equiv., 0.9 mmol, 440 mg), phenylboronic acid (1.2 equiv., 1.08 mmol, 132 mg) and tripotassium phosphate (1.2 equiv., 1.08 mmol, 229 mg) were then added sequentially. The vial was sealed, and the reaction mixture was stirred at 110 °C for 2 h. Upon completion of the reaction, the mixture was cooled to room temperature, and the magnetic stir bar was removed. The reaction mixture was transferred to a separatory funnel, diluted with ethyl acetate (75 mL), and washed with distilled water (3 × 75 mL). The organic layer was separated, dried over anhydrous sodium sulfate, and filtered. Silica gel was then added, and the solvents were removed under reduced pressure using a rotary evaporator (water bath temperature 40 °C; pressure gradually reduced from 200 mbar to 1–5 mbar). The residue was triturated with diethyl ether to give pure **33**.

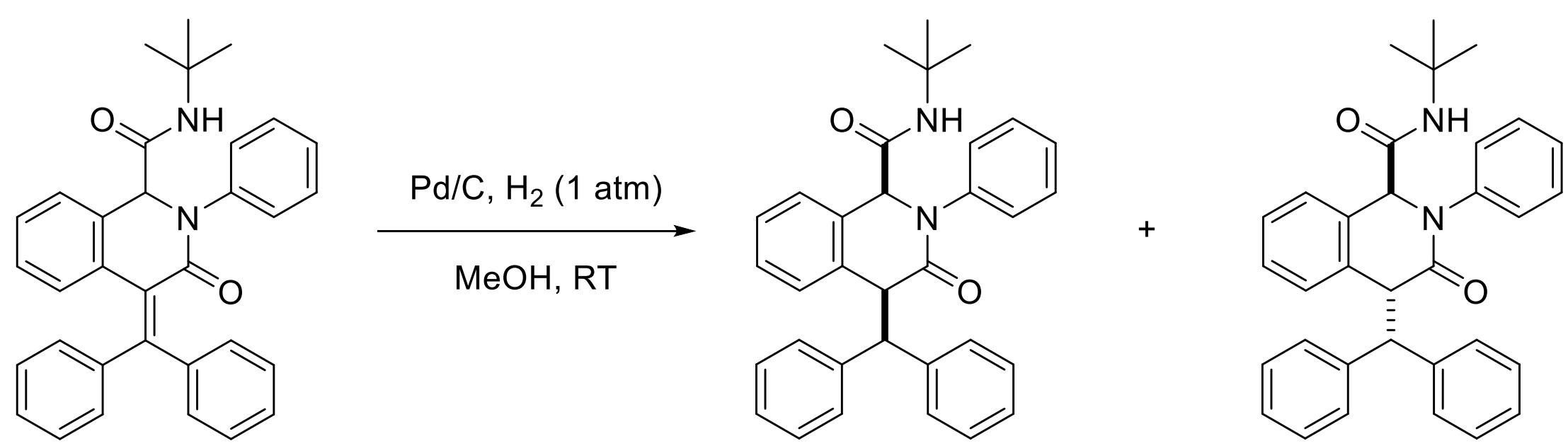

33 (±)-*cis*-**34** (±)-*trans*-**34**

Procedure for the heterogeneous hydrogenation of isoquinoline derivative 33

The screw-cap vial containing **33** (0.3 mmol, 146 mg) was equipped with a magnetic stir bar and charged with methanol (15 mL). The vial was sealed with a septum and the solution was purged with argon, after which Pd/C (20 mol%, 0.06 mmol, 64 mg of 10 wt % Pd) was added. The resulting suspension was sequentially flushed with argon and hydrogen, then stirred under a hydrogen atmosphere (1 atm) at room temperature for 72 hours. The reaction progress was monitored by thin-layer chromatography (TLC). After completion, the reaction mixture was diluted with dichloromethane and filtered through a glass filter with extra-fine porosity to remove the Pd catalyst. The filtrate was concentrated and dried under reduced pressure using a rotary evaporator (water bath temperature 40 °C; pressure gradually reduced from 700 mbar to 1–5 mbar) to obtain crude product, which was subsequently purified by column chromatography on silica gel using dichloromethane/ethyl acetate as the eluent to give pure *cis*-**34** and *trans*-**34**.

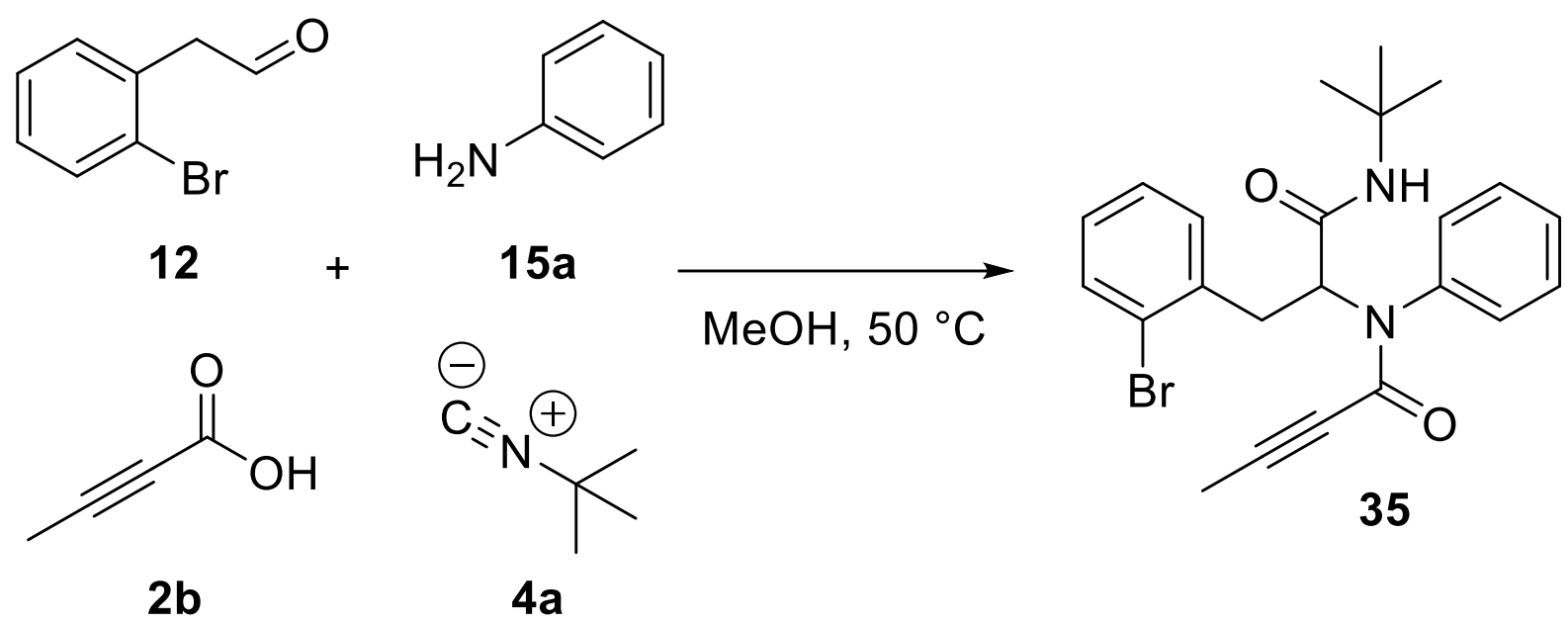

Procedure for the synthesis of Ugi adduct 35

But-2-ynoic acid (**2b**, 1.1 equiv., 1.65 mmol, 139 mg) was placed in a screw-cap vial equipped with a magnetic stir bar, followed by the addition of methanol (7.5 mL), 2-(2-bromophenyl)acetaldehyde (**12**, 1.0 equiv., 1.5 mmol, 299 mg), aniline (**15a**, 1.1 equiv., 1.65 mmol, 154 mg), tert-butyl isocyanide (**4a**, 1.1 equiv., 1.65 mmol, 137 mg). The vial was sealed and the reaction mixture was stirred at 50 °C for 48 h. Upon completion of the reaction, the magnetic stir bar was removed and, unless otherwise stated, the reaction mixture was diluted with ethyl acetate, and silica gel was added. The solvents were removed under reduced pressure using a rotary evaporator (water bath temperature 40 °C; pressure gradually reduced from 200 mbar to 1–5 mbar). The residue was purified by column chromatography on silica gel using hexane/ethyl acetate as the eluent, followed by trituration with pentane, to afford the corresponding Ugi adduct **35** in pure form.

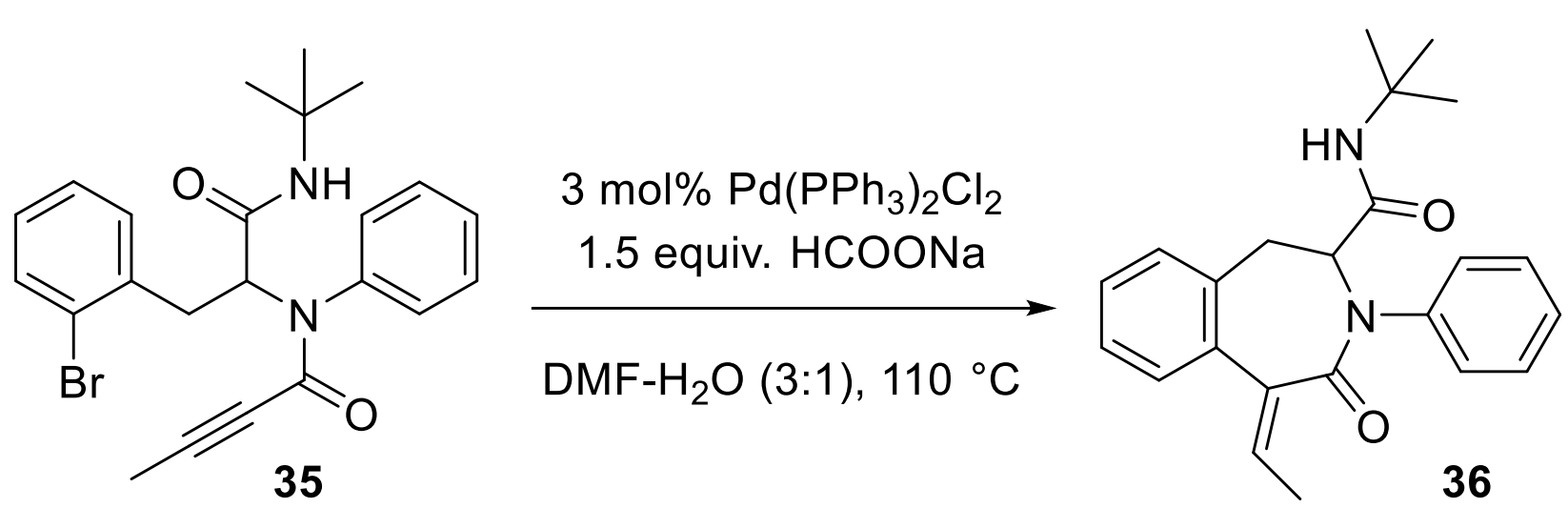

Procedure for the synthesis of 3-benzazepine derivative 36

Bis(triphenylphosphine)palladium(II) chloride (3 mol%, 0.0165 mmol, 12 mg) was placed in a screw-cap vial equipped with a magnetic stir bar. A 3:1 mixture of dimethylformamide (2.7 mL) and distilled water (0.9 mL) was added, followed by the *N*-(3-(2-bromophenyl)-1-(*tert*-butylamino)-1-oxopropan-2-yl)-*N*-phenylbut-2-ynamide (**35**, 1.0 equiv., 0.55 mmol, 243 mg). Sodium formate (1.5 equiv., 0.825 mmol, 56 mg) was then added, and the reaction mixture was purged with argon. The vial was sealed, and the resulting mixture was stirred at 110 °C for 1 h. Upon completion of the reaction, the mixture was cooled to room temperature and the magnetic stir bar was removed. The reaction mixture was transferred to a separatory funnel, diluted with ethyl acetate (50 mL), and washed with distilled

water (3 × 50 mL). The organic layer was separated, dried over anhydrous sodium sulfate, and filtered. Silica gel was then added, and the solvents were removed under reduced pressure using a rotary evaporator (water bath temperature 40 °C; pressure gradually reduced from 200 mbar to 1–5 mbar). The residue was purified by column chromatography on silica gel using hexane/ethyl acetate as the eluent, followed by trituration with diethyl ether, to afford corresponding 3-benzazepine derivative **36**.

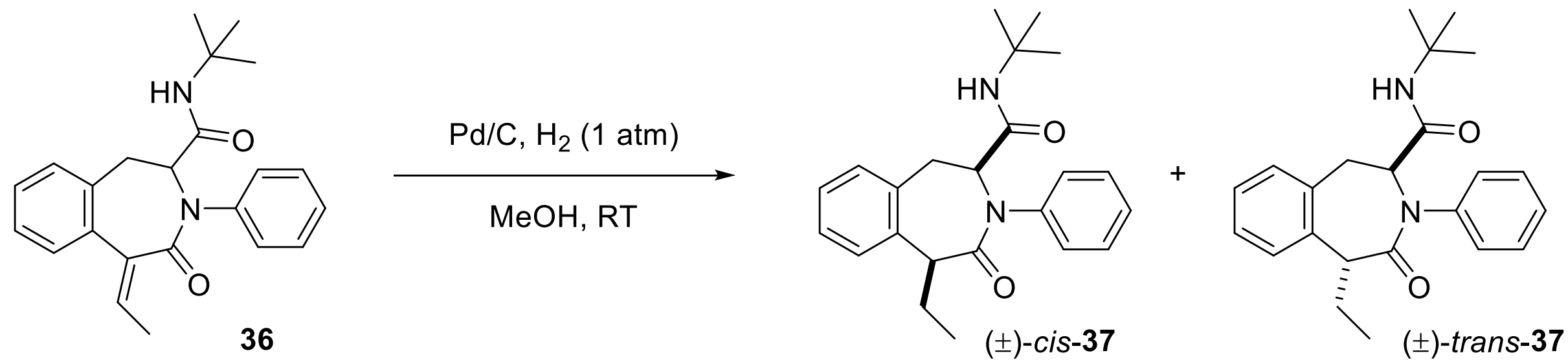

Procedure for the heterogeneous hydrogenation of 3-benzazepine derivative 36

The screw-cap vial containing (*Z*)-*N*-(*tert*-butyl)-5-ethylidene-4-oxo-3-phenyl-2,3,4,5-tetrahydro-1*H*-benzo[*d*]azepine-2-carboxamide (**36**, 0.35 mmol, 127 mg) was equipped with a magnetic stir bar and charged with methanol (17.5 mL). The vial was sealed with a septum and the solution was purged with argon, after which Pd/C (10 mol%, 0.035 mmol, 37 mg of 10 wt % Pd) was added. The resulting suspension was sequentially flushed with argon and hydrogen, then stirred under a hydrogen atmosphere (1 atm) at room temperature for 48 hours. The reaction progress was monitored by thin-layer chromatography (TLC). After completion, the reaction mixture was diluted with dichloromethane and filtered through a glass filter with extra-fine porosity to remove the Pd catalyst. The filtrate was concentrated and dried under reduced pressure using a rotary evaporator (water bath temperature 40 °C; pressure gradually reduced from 700 mbar to 1–5 mbar) to obtain crude product, which was subsequently purified by column chromatography on silica gel using hexane/ethyl acetate as the eluent to give pure *cis*-**37** and *trans*-**37**.

Characterization of synthesized compounds 27

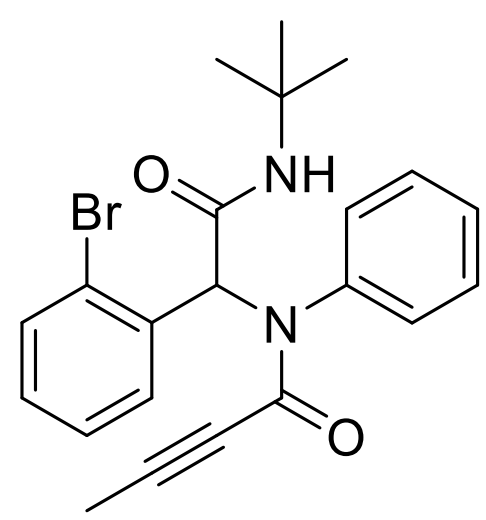	***N*-(1-(2-Bromophenyl)-2-(*tert*-butylamino)-2-oxoethyl)-*N*-phenylbut-2-ynamide 27a**<br>Purified by column chromatography on silica gel using hexane/ethyl acetate (20–40% ethyl acetate) as the eluent, followed by trituration with pentane. Yield: 538-596 mg (84-93%, 1.5 mmol scale), 1214 mg (71%, 4.0 mmol scale), white solid, melting point: 182-184 °C.<br>**^{1}H NMR** (500 MHz, $CDCl_3$) δ 7.53 – 7.46 (m, 1H), 7.31 (br s, 2H), 7.18 – 7.11 (m, 4H), 7.05 – 6.98 (m, 2H), 6.26 (s, 1H), 5.57 (s, 1H), 1.68 (s, 3H), 1.37 (s, 9H).<br>**^{13}C{^{1}H} NMR** (101 MHz, $CDCl_3$) δ 167.8, 154.7, 139.0, 133.8, 132.7, 131.7, 130.2, 129.9, 128.02, 127.98, 127.1, 126.1, 90.9, 73.9, 63.9, 51.7, 28.5, 3.8.<br>**HRMS** (ESI): m/z $[M+Na]^+$ for $C_{22}H_{23}BrN_2O_2$, calcd 449.0835, found 449.0829.
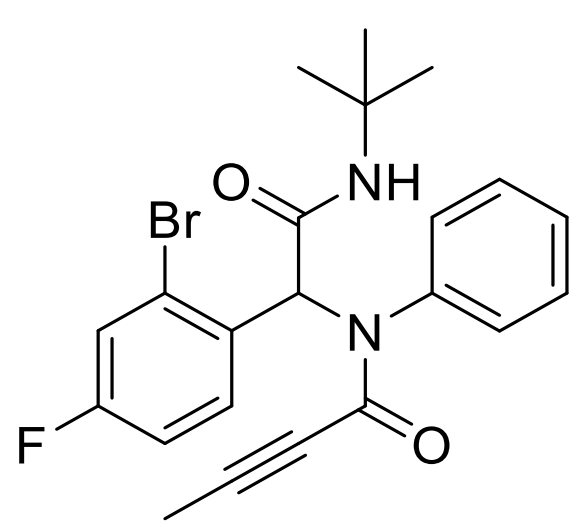	***N*-(1-(2-Bromo-4-fluorophenyl)-2-(*tert*-butylamino)-2-oxoethyl)-*N*-phenylbut-2-ynamide 27b**<br>Purified by column chromatography on silica gel using hexane/ethyl acetate (10–40% ethyl acetate) as the eluent, followed by trituration with pentane. Yield: 601 mg (90%, 1.5 mmol scale), white solid, melting point: 205-206°C.<br>**^{1}H NMR** (500 MHz, $CDCl_3$) δ 7.27 – 7.23 (m, 3H), 7.21 – 7.13 (m, 4H), 6.75 – 6.68 (m, 1H), 6.25 (s, 1H), 5.68 (s, 1H), 1.68 (s, 3H), 1.37 (s, 9H).<br>**^{13}C{^{1}H} NMR** (126 MHz, $CDCl_3$) δ 168.0, 162.1 (d, *J* = 253.4 Hz), 155.0, 138.9, 133.2 (d, *J* = 8.8 Hz), 130.4, 129.9 (d, *J* = 3.8 Hz), 128.44, 128.42, 126.5 (d, *J* = 9.4 Hz), 120.1 (d, *J* = 24.3 Hz), 114.5 (d, *J* = 20.9 Hz), 91.5, 73.9, 63.0, 52.0, 28.7, 4.0.<br>**HRMS** (ESI): m/z $[M+Na]^+$ for $C_{22}H_{22}BrFN_2O_2$, calcd 467.0741, found 467.0729.

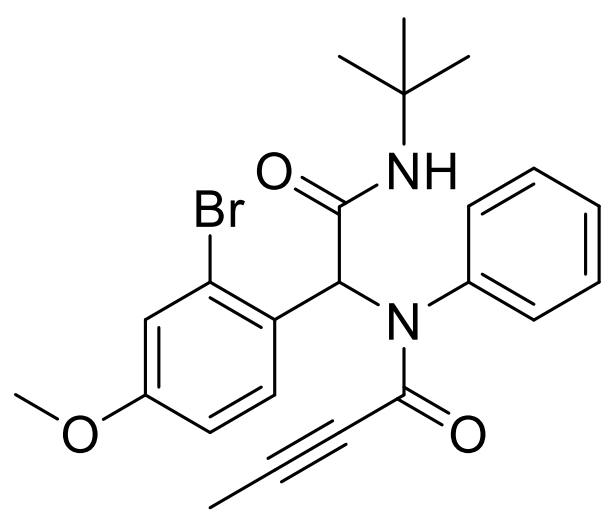

***N*-(1-(2-Bromo-4-methoxyphenyl)-2-(*tert*-butylamino)-2-oxoethyl)-*N*-phenylbut-2-ynamide 27c**

Purified by column chromatography on silica gel using hexane/ethyl acetate (20–40% ethyl acetate) as the eluent, followed by trituration with pentane. Yield: 542 mg (79%, 1.5 mmol scale), white solid, melting point: 184-185 °C.

^{1}H NMR (400 MHz, $CDCl_3$) δ 7.32 – 7.22 (m, 2H), 7.16 – 7.11 (m, 3H), 7.04 (d, *J* = 8.9 Hz, 1H), 7.02 (d, *J* = 2.7 Hz, 1H), 6.51 (dd, *J* = 8.8, 2.6 Hz, 1H), 6.21 (s, 1H), 5.59 (s, 1H), 3.69 (s, 3H), 1.65 (s, 3H), 1.35 (s, 9H).

^{13}C{^{1}H} NMR (151 MHz, $CDCl_3$) δ 168.2, 159.9, 154.8, 139.1, 132.4, 130.4, 128.2, 128.1, 126.6, 125.6, 117.9, 113.1, 90.9, 74.0, 63.3, 55.5, 51.7, 28.6, 3.8.

HRMS (ESI): m/z $[M+H]^+$ for $C_{23}H_{25}BrN_2O_3$, calcd 457.1121, found 457.1108.

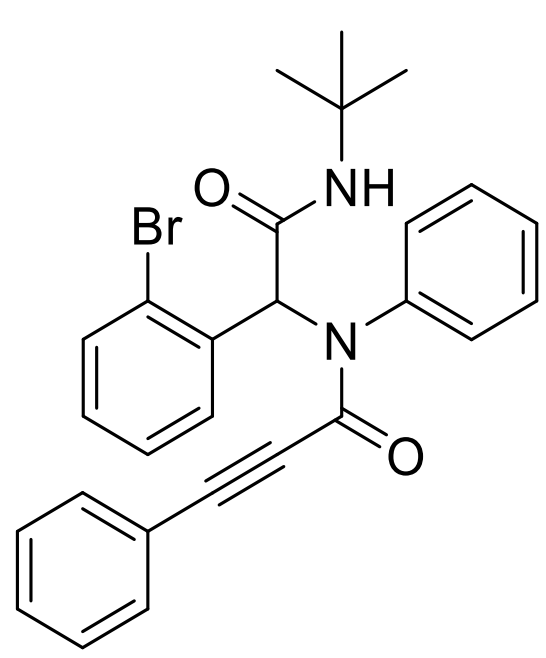

***N*-(1-(2-Bromophenyl)-2-(*tert*-butylamino)-2-oxoethyl)-*N*,3-diphenylpropiolamide 27d**

Purified by column chromatography on silica gel using hexane/ethyl acetate (10–40% ethyl acetate) as the eluent, followed by trituration with pentane. Yield: 609 mg (83%, 1.5 mmol scale), white solid, melting point: 154-155 °C.

^{1}H NMR (500 MHz, $CDCl_3$) δ 7.54 – 7.50 (m, 1H), 7.40 (br s, 2H), 7.32 – 7.27 (m, 1H), 7.23 – 7.16 (m, 6H), 7.07 – 7.01 (m, 4H), 6.34 (s, 1H), 5.60 (s, 1H), 1.39 (s, 9H).

^{13}C{^{1}H} NMR (126 MHz, $CDCl_3$) δ 167.9, 155.0, 139.2, 133.8, 132.9, 132.6, 132.0, 130.8, 130.2, 130.0, 128.41, 128.37, 128.3, 127.4, 126.4, 120.4, 92.1, 82.5, 64.1, 52.1, 28.7.

HRMS (ESI): m/z $[M+Na]^+$ for $C_{27}H_{25}BrN_2O_2$, calcd 511.0992, found 511.0969.

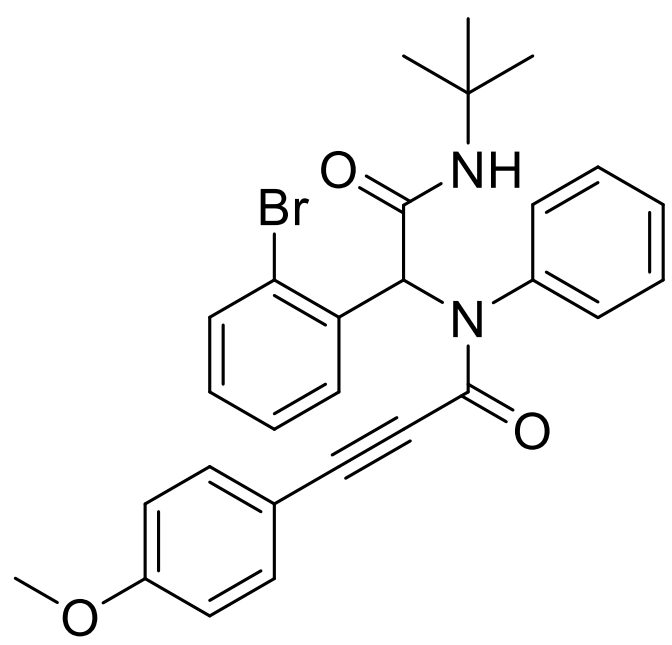

_N_-(1-(2-Bromophenyl)-2-(_tert_-butylamino)-2-oxoethyl)-3-(4-methoxyphenyl)-_N_-phenylpropiolamide 27e

No column chromatography was performed. Upon completion of the reaction, the reaction vial was kept at 4 °C for 24 h to allow precipitation of the product. The solvent was decanted, and the resulting precipitate was washed with diethyl ether to afford the pure product. Yield: 600 mg (77%, 1.5 mmol scale), white solid, melting point: 162-164 °C.

^{1}H NMR (500 MHz, $CDCl_3$) δ 7.54 – 7.49 (m, 1H), 7.39 (br s, 2H), 7.24 – 7.15 (m, 4H), 7.08 – 7.00 (m, 2H), 6.96 (d, *J* = 8.7 Hz, 2H), 6.71 (d, *J* = 8.4 Hz, 2H), 6.34 (s, 1H), 5.63 (s, 1H), 3.75 (s, 3H), 1.39 (s, 9H).

^{13}C{^{1}H} NMR (101 MHz, $CDCl_3$) δ 167.9, 160.9, 155.1, 139.3, 134.3, 133.9, 132.8, 131.8, 130.7, 130.0, 128.2, 127.2, 126.2, 113.9, 112.1, 92.7, 81.9, 64.0, 55.2, 51.8, 28.6.

HRMS (ESI): m/z $[M+Na]^+$ for $C_{28}H_{27}BrN_2O_3$, calcd 541.1097, found 541.1076.

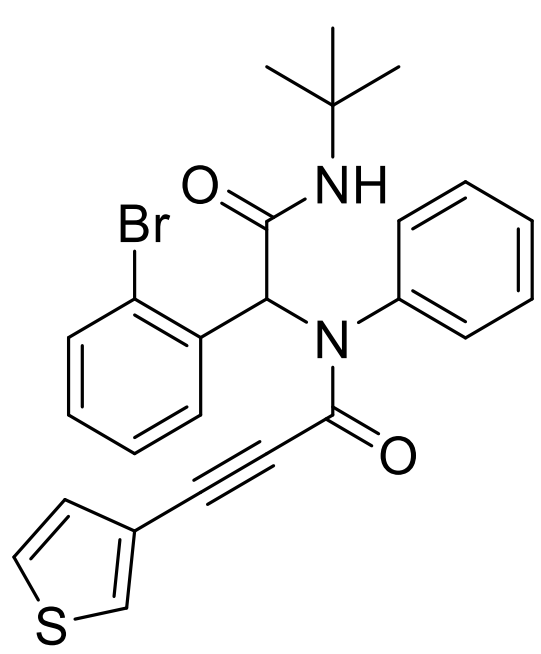

_N_-(1-(2-Bromophenyl)-2-(_tert_-butylamino)-2-oxoethyl)-_N_-phenyl-3-(thiophen-3-yl)propiolamide 27f

Purified by column chromatography on silica gel using hexane/ethyl acetate (10–30% ethyl acetate) as the eluent, followed by trituration with pentane. Yield: 639 mg (86%, 1.5 mmol scale), white solid, melting point: 166-168 °C.

^{1}H NMR (500 MHz, $CDCl_3$) δ 7.54 – 7.49 (m, 1H), 7.39 (br s, 2H), 7.23 – 7.12 (m, 6H), 7.07 – 7.00 (m, 2H), 6.71 (d, *J* = 5.2 Hz, 1H), 6.33 (s, 1H), 5.59 (s, 1H), 1.39 (s, 9H).

^{13}C{^{1}H} NMR (126 MHz, $CDCl_3$) δ 167.9, 155.1, 139.2, 133.8, 133.0, 132.6, 132.0, 130.8, 130.2, 130.1, 128.4, 128.3, 127.4, 126.4, 125.7, 119.7, 87.5, 82.5, 64.1, 52.1, 28.7.

HRMS (ESI): m/z $[M+H]^+$ for $C_{25}H_{23}BrN_2O_2S$, calcd 495.0736, found 495.0724.

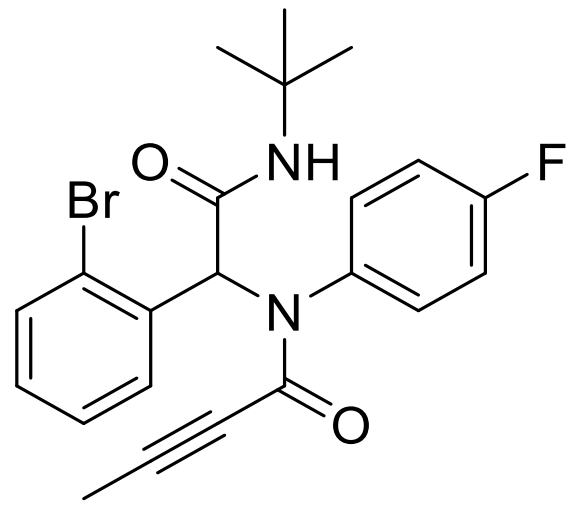	***N*-(1-(2-Bromophenyl)-2-(*tert*-butylamino)-2-oxoethyl)-*N*-(4-fluorophenyl)but-2-ynamide 27g**<br>Purified by column chromatography on silica gel using hexane/ethyl acetate (10–30% ethyl acetate) as the eluent, followed by trituration with diethyl ether. Yield: 534 mg (80%, 1.5 mmol scale), white solid, melting point: 199-200 °C.<br>**^{1}H NMR** (500 MHz, $CDCl_3$) δ 7.54 – 7.47 (m, 1H), 7.32 (br s, 2H), 7.15 – 7.11 (m, 1H), 7.08 – 7.00 (m, 2H), 6.85 – 6.75 (m, 2H), 6.24 (s, 1H), 5.51 (s, 1H), 1.70 (s, 3H), 1.36 (s, 9H).<br>**^{13}C{^{1}H} NMR** (101 MHz, $CDCl_3$) δ 168.1, 162.1 (d, *J* = 247.7 Hz), 155.0, 135.1 (d, *J* = 3.0 Hz), 133.8, 133.0, 132.4 (d, *J* = 8.7 Hz), 131.8, 130.3, 127.5, 126.3, 115.1 (d, *J* = 22.5 Hz), 91.5, 74.0, 63.9, 52.1, 28.7, 4.0.
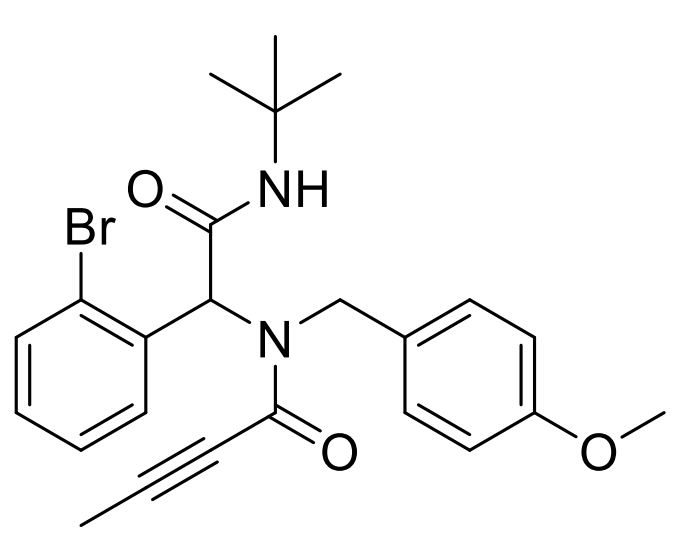	***N*-(1-(2-Bromophenyl)-2-(*tert*-butylamino)-2-oxoethyl)-*N*-(4-methoxybenzyl)but-2-ynamide 27h**<br>Purified by column chromatography on silica gel using hexane/ethyl acetate (10–30% ethyl acetate) as the eluent. Yield: 1145 mg (81%, 3.0 mmol scale), white solid, melting point: 182-184 °C, observed as a mixture of rotamers in the NMR spectra.<br>**^{1}H NMR** (400 MHz, $CDCl_3$) δ 7.57 – 7.50 (m, 1H), 7.42 – 7.36 (m, 0.35H), 7.33 – 7.26 (m, 1.35H), 7.26 – 7.19 (m, 0.65H), 7.08 – 7.03 (m, 0.65H), 7.00 (d, *J* = 8.7 Hz, 0.7H), 6.94 (d, *J* = 8.6 Hz, 1.3H), 6.72 (d, *J* = 8.7 Hz, 0.7H), 6.65 (d, *J* = 8.7 Hz, 1.3H), 6.17 (s, 0.35H), 5.91 (s, 0.65H), 5.48 (s, 0.35H), 5.46 (s, 0.65H), 4.95 (d, *J* = 16.2 Hz, 0.65H), 4.73 (d, *J* = 14.9 Hz, 0.35H), 4.57 (d, *J* = 16.2 Hz, 0.65H), 3.84 (d, *J* = 14.9 Hz, 0.35H), 3.75 (s, 1.05H), 3.74 (s, 1.95H), 2.02 (s, 1.05H), 1.98 (s, 1.95H), 1.27 (s, 5.85H), 1.17 (s, 3.15H).<br>**^{13}C{^{1}H} NMR** (101 MHz, $CDCl_3$) δ 168.1, 167.7, 159.2, 158.8, 156.9, 155.9, 134.32, 134.27, 133.5, 133.1, 131.3, 131.2, 130.5, 130.3, 130.2, 130.0, 129.1, 128.8, 127.8, 127.6, 127.1, 126.7, 114.1, 113.6, 91.9, 90.4, 73.9, 73.3, 67.8, 62.3, 55.42, 55.39, 51.8, 51.7, 51.2, 45.3, 28.6, 28.4, 4.3.<br>**HRMS** (ESI): m/z $[M+H]^+$ for $C_{24}H_{27}BrN_2O_3$, calcd 471.1278, found 471.1277.

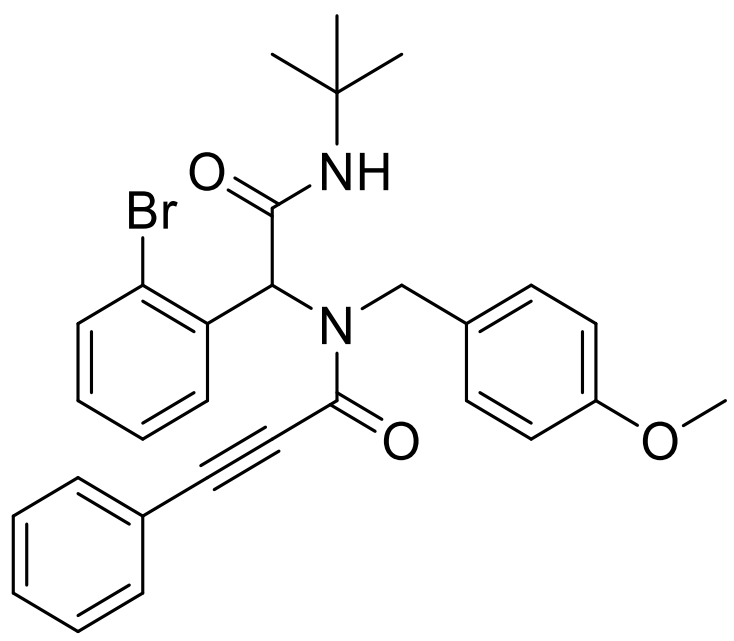

N-(1-(2-Bromophenyl)-2-(*tert*-butylamino)-2-oxoethyl)-N-(4-methoxybenzyl)-3-phenylpropiolamide 27i

No column chromatography was performed. Upon completion of the reaction, the reaction vial was kept at 4 °C for 24 h to allow precipitation of the product. The solvent was decanted, and the resulting precipitate was washed with diethyl ether to afford the pure product. Yield: 560 mg (70%, 1.5 mmol scale), white solid, melting point: 183-185 °C, observed as a mixture of rotamers in the NMR spectra.

^{1}H NMR (500 MHz, CDCl$_3$) δ 7.61 – 7.57 (m, 1.38H), 7.57 – 7.54 (m, 0.38H), 7.47 – 7.28 (m, 6.24H), 7.25 – 7.22 (m, 0.38H), 7.11 – 7.07 (m, 0.62H), 7.05 (d, *J* = 8.6 Hz, 0.76H), 7.00 (d, *J* = 8.7 Hz, 1.24H), 6.74 (d, *J* = 8.7 Hz, 0.76H), 6.66 (d, *J* = 8.6 Hz, 1.24H), 6.29 (s, 0.38H), 6.01 (s, 0.62H), 5.53 (s, 0.38H), 5.47 (s, 0.62H), 5.07 (d, *J* = 16.3 Hz, 0.62H), 4.82 (d, *J* = 14.9 Hz, 0.38H), 4.66 (d, *J* = 16.3 Hz, 0.62H), 3.91 (d, *J* = 14.9 Hz, 0.38H), 3.76 (s, 1.14H), 3.73 (s, 1.86H), 1.30 (s, 5.58H), 1.14 (s, 3.42H).

^{13}C{^{1}H} NMR (101 MHz, CDCl$_3$) δ 168.1, 167.7, 159.2, 158.8, 156.9, 155.9, 134.21, 134.15, 133.5, 133.1, 132.9, 132.6, 131.17, 131.15, 130.6, 130.5, 130.29, 130.27, 129.9, 129.0, 128.7, 128.63, 128.57, 128.3, 127.8, 127.6, 127.2, 126.8, 120.4, 120.1, 114.1, 113.7, 92.6, 91.2, 82.2, 81.5, 67.9, 62.4, 55.4, 55.3, 51.9, 51.8, 51.2, 45.5, 28.6, 28.4.

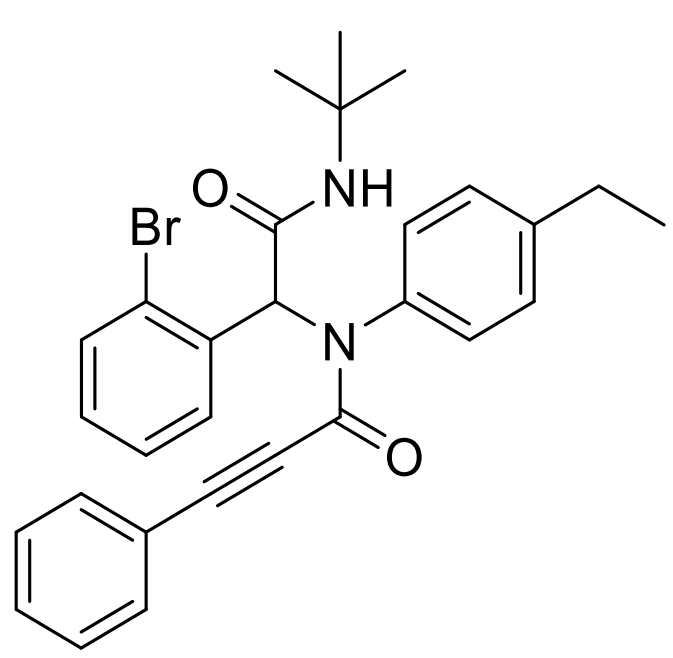

N-(1-(2-Bromophenyl)-2-(*tert*-butylamino)-2-oxoethyl)-N-(4-ethylphenyl)-3-phenylpropiolamide 27j

Purified by column chromatography on silica gel using hexane/ethyl acetate (10–30% ethyl acetate) as the eluent. Yield: 1351 mg (87%, 3.0 mmol scale), white solid, melting point: 191-193 °C.

^{1}H NMR (400 MHz, CDCl$_3$) δ 7.55 – 7.51 (m, 1H), 7.32 – 7.26 (m, 2H), 7.26 – 7.15 (m, 4H), 7.07 – 6.97 (m, 6H), 6.33 (s, 1H), 5.61 (s, 1H), 2.56 (q, *J* = 7.6 Hz, 2H), 1.38 (s, 9H), 1.16 (t, *J* = 7.6 Hz, 3H).

^{13}C{^{1}H} NMR (101 MHz, CDCl$_3$) δ 167.9, 155.2, 144.6, 136.9, 134.0, 132.9, 132.7, 132.0, 130.6, 130.1, 130.0, 128.3, 127.8, 127.4, 126.3, 120.6, 92.0, 82.7, 64.1, 52.0, 28.7, 28.6, 15.8.

	**HRMS** (ESI): m/z [M+Na]$^+$ for $C_{29}H_{29}BrN_2O_2$, calcd 539.1305, found 539.1305.
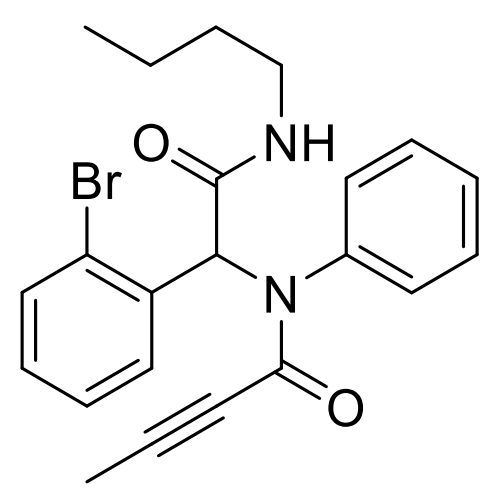	***N*-(1-(2-Bromophenyl)-2-(butylamino)-2-oxoethyl)-*N*-phenylbut-2-ynamide 27k**<br>Purified by column chromatography on silica gel using hexane/ethyl acetate (10–30% ethyl acetate) as the eluent. Yield: 1090 mg (85%, 3.0 mmol scale), white solid, melting point: 129-131 °C.<br>**^{1}H NMR** (400 MHz, $CDCl_3$) δ 7.53 – 7.47 (m, 1H), 7.34 – 7.26 (m, 2H), 7.17 – 7.10 (m, 4H), 7.06 – 6.96 (m, 2H), 6.39 (s, 1H), 5.76 (br s, 1H), 3.37 – 3.27 (m, 2H), 1.68 (s, 3H), 1.54 – 1.44 (m, 2H), 1.40 – 1.22 (m, 2H), 0.90 (t, *J* = 7.3 Hz, 3H).<br>**^{13}C{^{1}H} NMR** (101 MHz, $CDCl_3$) δ 168.8, 154.9, 139.1, 133.8, 132.8, 132.1, 130.4, 130.1, 128.24, 128.20, 127.2, 126.2, 91.2, 74.0, 63.6, 39.8, 31.5, 20.1, 13.8, 3.9.<br>**HRMS** (ESI): m/z [M+Na]$^+$ for $C_{22}H_{23}BrN_2O_2$, calcd 449.0835, found 449.0828.
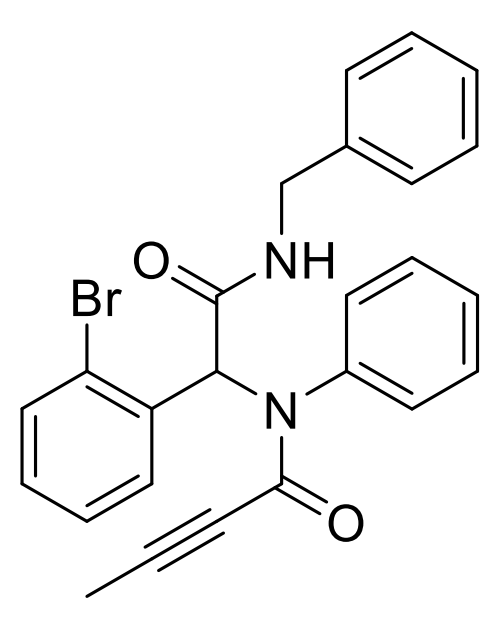	***N*-(2-(Benzylamino)-1-(2-bromophenyl)-2-oxoethyl)-*N*-phenylbut-2-ynamide 27l**<br>Purified by column chromatography on silica gel using hexane/ethyl acetate (20–30% ethyl acetate) as the eluent. Yield: 429 mg (62%, 1.5 mmol scale), white solid, melting point: 139-140 °C.<br>**^{1}H NMR** (500 MHz, $CDCl_3$) δ 7.52 – 7.46 (m, 1H), 7.33 – 7.23 (m, 7H), 7.17 – 7.11 (m, 4H), 7.06 – 6.96 (m, 2H), 6.44 (s, 1H), 6.06 (t, *J* = 6.1 Hz, 1H), 4.56 (dd, *J* = 14.9, 5.7 Hz, 1H), 4.50 (dd, *J* = 14.9, 5.8 Hz, 1H), 1.69 (s, 3H).<br>**^{13}C{^{1}H} NMR** (126 MHz, $CDCl_3$) δ 168.9, 155.0, 138.9, 137.9, 133.4, 132.8, 132.1, 130.4, 130.2, 128.6, 128.25, 128.22, 127.7, 127.4, 127.2, 126.2, 91.3, 73.9, 63.7, 43.8, 3.9.<br>**HRMS** (ESI): m/z [M+H]$^+$ for $C_{25}H_{21}BrN_2O_2$, calcd 461.0859, found 461.0869.

Characterization of synthesized compounds 28

Purification by column chromatography on silica gel using hexane/ethyl acetate (40% ethyl acetate) as the eluent afforded the individual *Z* and *E* isomers, which were further purified by trituration with diethyl ether.

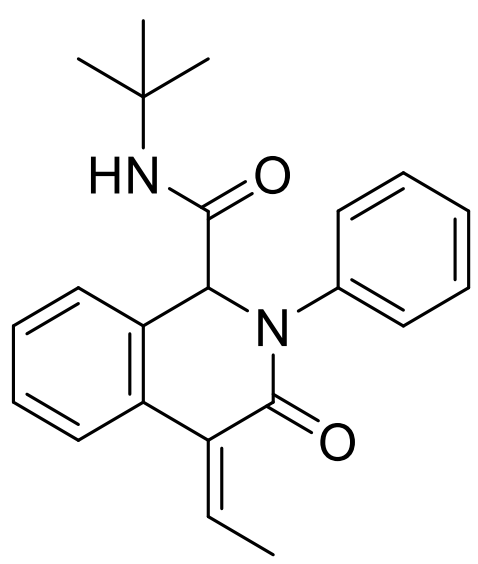

(*Z*)-*N*-(*tert*-Butyl)-4-ethylidene-3-oxo-2-phenyl-1,2,3,4-tetrahydroisoquinoline-1-carboxamide (*Z*)-28a

Yield: 575 mg (55%, 3.0 mmol scale), white solid, melting point: 215-217 °C.

^{1}H NMR (500 MHz, $CDCl_3$) δ 7.51 – 7.46 (m, 1H), 7.44 – 7.27 (m, 8H), 6.65 (q, *J* = 7.3 Hz, 1H), 5.74 (s, 1H), 5.05 (s, 1H), 2.33 (d, *J* = 7.4 Hz, 3H), 1.25 (s, 9H).

^{13}C{^{1}H} NMR (126 MHz, $CDCl_3$) δ 168.4, 165.1, 141.8, 137.9, 134.2, 130.4, 130.0, 129.4, 128.9, 127.6, 127.3, 126.22, 126.18, 124.0, 69.5, 51.9, 28.6, 16.1.

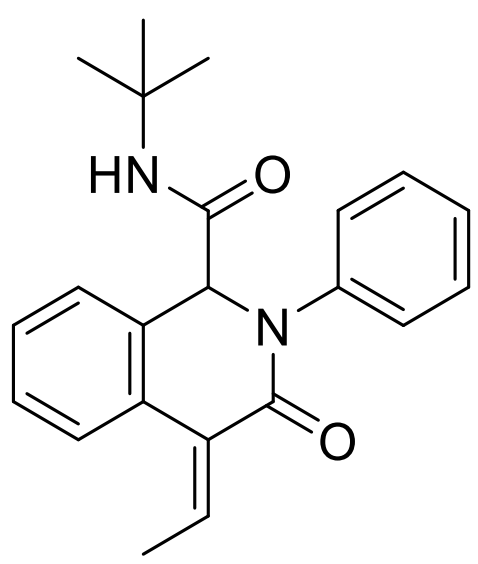

(*E*)-*N*-(*tert*-Butyl)-4-ethylidene-3-oxo-2-phenyl-1,2,3,4-tetrahydroisoquinoline-1-carboxamide (*E*)-28a

Yield: 73 mg (7%, 3.0 mmol scale). Yellowish solid, melting point (decomposition): 193-194 °C.

^{1}H NMR (500 MHz, $CDCl_3$) δ 7.54 – 7.50 (m, 1H), 7.45 – 7.39 (m, 4H), 7.37 – 7.32 (m, 3H), 7.31 – 7.27 (m, 1H), 7.13 (q, *J* = 7.5 Hz, 1H), 5.71 (s, 1H), 5.07 (s, 1H), 2.17 (d, *J* = 7.6 Hz, 3H), 1.24 (s, 9H).

^{13}C{^{1}H} NMR (126 MHz, $CDCl_3$) δ 168.3, 166.8, 142.0, 135.4, 132.1, 131.1, 130.7, 129.4, 128.2, 127.83, 127.77, 127.2, 126.8, 126.0, 69.3, 51.9, 28.6, 15.6.

HRMS (ESI): m/z $[M+Na]^+$ for $C_{22}H_{24}N_2O_2$, calcd 371.1730, found 371.1730.

Purification by column chromatography on silica gel using hexane/ethyl acetate (30-40% ethyl acetate) as the eluent afforded the individual *Z* and *E* isomers, which were further purified by trituration with diethyl ether.

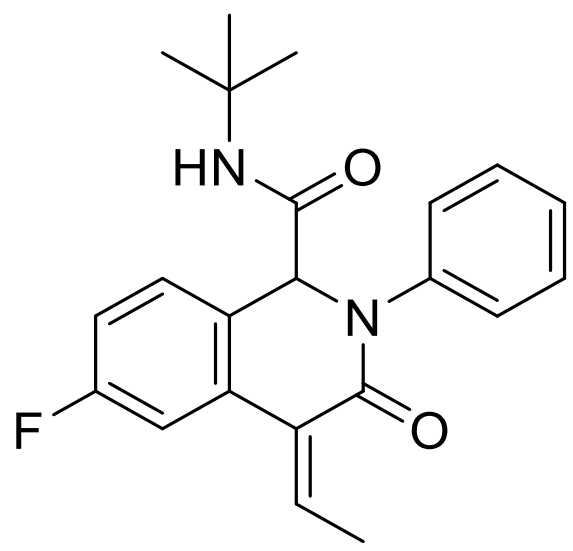

(*Z*)-*N*-(*tert*-Butyl)-4-ethylidene-6-fluoro-3-oxo-2-phenyl-1,2,3,4-tetrahydroisoquinoline-1-carboxamide (*Z*)-28b

Yield: 159 mg (62%, 0.7 mmol scale), white solid, melting point: 222-223 °C.

^{1}H NMR (500 MHz, $CDCl_3$) δ 7.45 – 7.40 (m, 2H), 7.34 – 7.28 (m, 4H), 7.20 – 7.13 (m, 1H), 7.05 – 6.95 (m, 1H), 6.65 (q, *J* = 7.3 Hz, 1H), 5.73 (s, 1H), 5.02 (s, 1H), 2.34 (d, *J* = 7.3 Hz, 3H), 1.25 (s, 9H).

^{13}C{^{1}H} NMR (126 MHz, $CDCl_3$) δ 168.2, 164.6, 163.2 (d, *J* = 247.1 Hz), 141.7, 139.2, 136.4 (d, *J* = 8.3 Hz), 129.5, 129.3 (d, *J* = 2.2 Hz), 128.1 (d, *J* = 8.7 Hz), 127.5, 126.4 (d, *J* = 2.9 Hz), 126.2, 114.7 (d, *J* = 22.4 Hz), 110.7 (d, *J* = 23.1 Hz), 68.9, 52.0, 28.6, 16.1.

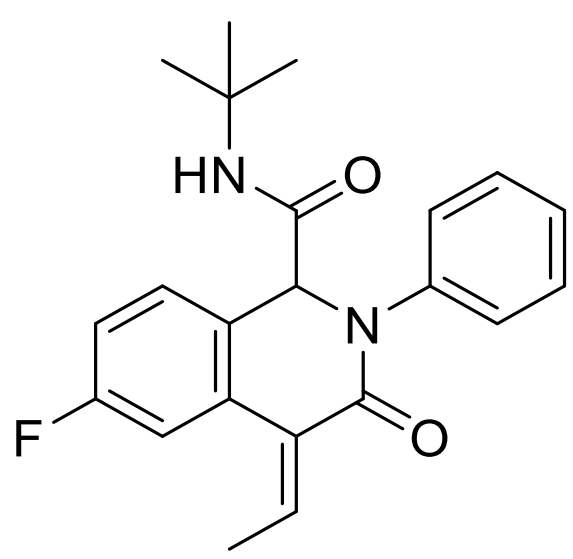

(*E*)-*N*-(*tert*-Butyl)-4-ethylidene-6-fluoro-3-oxo-2-phenyl-1,2,3,4-tetrahydroisoquinoline-1-carboxamide (*E*)-28b

Yield: 33 mg (13%, 0.7 mmol scale), white solid.

^{1}H NMR (500 MHz, $CDCl_3$) δ 7.44 – 7.36 (m, 3H), 7.33 – 7.27 (m, 3H), 7.21 (m, 1H), 7.17 (q, *J* = 7.4 Hz, 1H), 7.07 – 7.01 (m, 1H), 5.72 (s, 1H), 5.04 (s, 1H), 2.18 (d, *J* = 7.6 Hz, 3H), 1.24 (s, 9H).

^{13}C{^{1}H} NMR (126 MHz, $CDCl_3$) δ 168.1, 166.2, 162.4 (d, *J* = 247.0 Hz), 141.8, 136.8, 132.7 (d, *J* = 8.4 Hz), 130.3, 129.5, 128.6 (d, *J* = 8.7 Hz), 128.1 (d, *J* = 3.0 Hz), 127.4, 126.0, 114.9 (d, *J* = 22.2 Hz), 114.5 (d, *J* = 23.2 Hz), 68.7, 52.1, 28.7, 15.6.

HRMS (ESI): m/z $[M+H]^+$ for $C_{22}H_{23}FN_2O_2$, calcd 367.1816, found 367.1819.

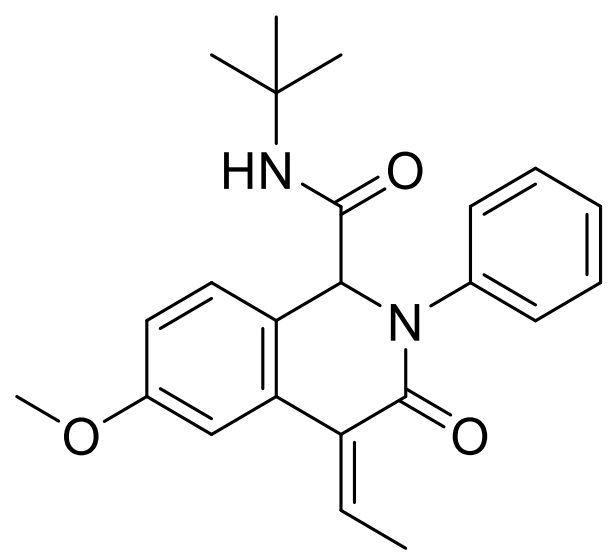

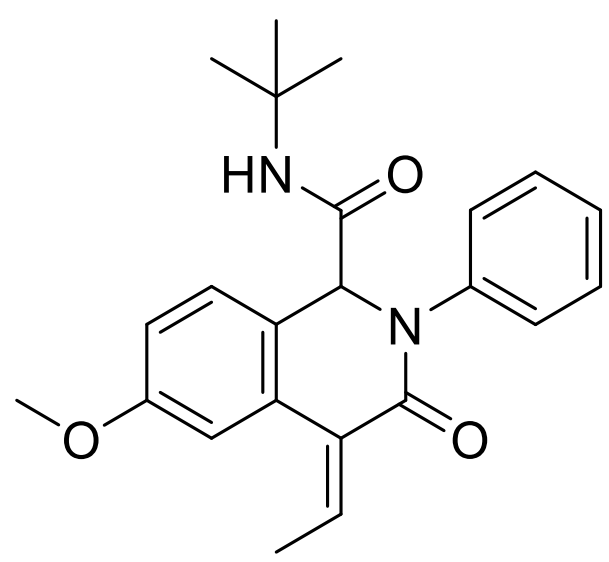

Purification by column chromatography on silica gel using hexane/ethyl acetate (40% ethyl acetate) as the eluent afforded a mixture of *Z* and *E* isomers. Yield for the mixture: 213 mg (75%, 0.75 mmol scale). Subsequent trituration with diethyl ether and methanol resulted in the removal of the *E* isomer, affording the pure *Z* isomer.

(*Z*)-*N*-(*tert*-Butyl)-4-ethylidene-6-methoxy-3-oxo-2-phenyl-1,2,3,4-tetrahydroisoquinoline-1-carboxamide (*Z*)-28c

Yield: 159 mg (56%, 0.75 mmol scale), white solid, melting point: 238-240 °C.

^{1}H NMR (500 MHz, $CDCl_3$) δ 7.98 (s, 1H), 7.45 – 7.38 (m, 3H), 7.34 – 7.28 (m, 1H), 7.22 – 7.15 (m, 2H), 7.10 – 7.05 (m, 1H), 6.92 – 6.86 (m, 1H), 6.71 (q, *J* = 7.3 Hz, 1H), 5.21 (s, 1H), 3.80 (s, 3H), 2.23 (d, *J* = 7.2 Hz, 3H), 1.16 (s, 9H).

^{13}C{^{1}H} NMR (126 MHz, DMSO-D_6) δ 168.7, 164.7, 159.3, 142.4, 135.8, 135.3, 129.1, 128.9, 126.83, 126.79, 126.72, 123.3, 113.7, 107.7, 66.2, 55.3, 50.5, 28.3, 16.0.

HRMS (ESI): m/z $[M+Na]^+$ for $C_{23}H_{26}N_2O_3$, calcd 401.1836, found 401.1835.

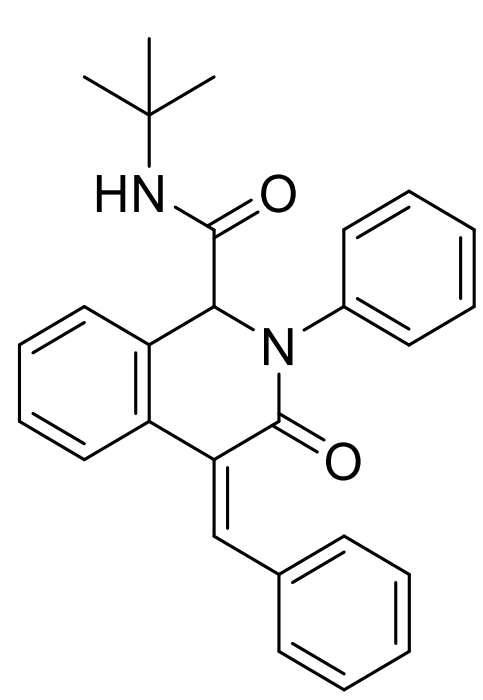

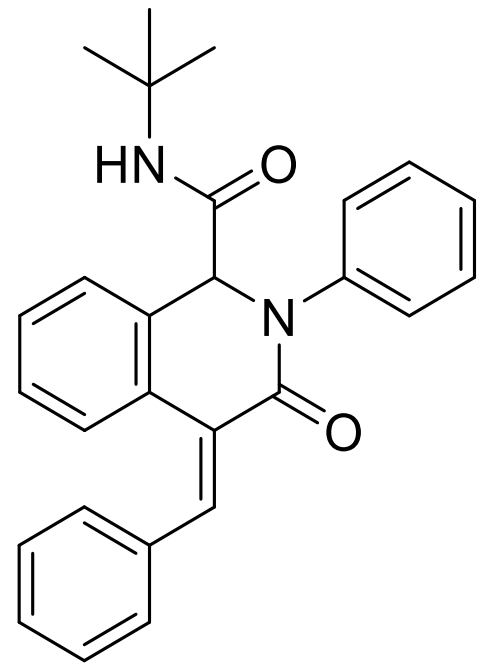

Purified by column chromatography on silica gel using hexane/ethyl acetate (30-40% ethyl acetate) as the eluent, followed by trituration with diethyl ether. Obtained as a mixture of *Z* and *E* isomers.

(*Z*)-4-Benzylidene-*N*-(*tert*-butyl)-3-oxo-2-phenyl-1,2,3,4-tetrahydroisoquinoline-1-carboxamide (*Z*)-28d + (*E*)-4-Benzylidene-*N*-(*tert*-butyl)-3-oxo-2-phenyl-1,2,3,4-tetrahydroisoquinoline-1-carboxamide (*E*)-28d

Yield: 217 mg (66%, 0.8 mmol scale), white solid.

^{1}H NMR (500 MHz, $CDCl_3$) δ 7.83 (s, 0.26H, minor), 7.77 – 7.73 (m, 1.48H, major), 7.68 – 7.65 (m, 0.74H, major), 7.48 – 7.26 (m, 11.52H, major+minor6), 7.20 (s, 0.74H, major), 7.15 – 7.12 (m, 0.26H, minor), 5.92 (s, 0.26H, minor), 5.83 (s, 0.74H, major), 5.14 (s, 0.74H, major), 5.12 (s, 0.26H, minor), 1.27 (s, 2.34H, minor), 1.20 (s, 6.66H, major).

^{13}C{^{1}H} NMR (126 MHz, $CDCl_3$) δ 168.1, 168.0, 166.5, 164.8, 141.94, 141.92, 137.9, 136.1, 135.29, 135.26, 135.0, 132.8, 131.7, 130.4,

	130.2, 130.0, 129.8, 129.40, 129.37, 129.35, 129.2, 128.9, 128.7, 128.4, 128.3, 128.2, 128.0, 127.8, 127.3, 127.2, 127.1, 126.4, 126.0, 125.9, 124.3, 69.5, 52.0, 51.9, 28.7, 28.5.
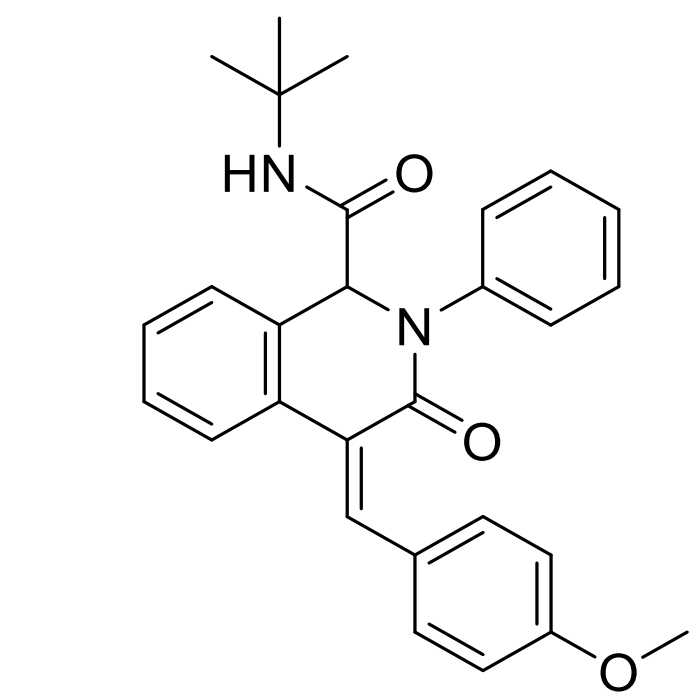<br><br>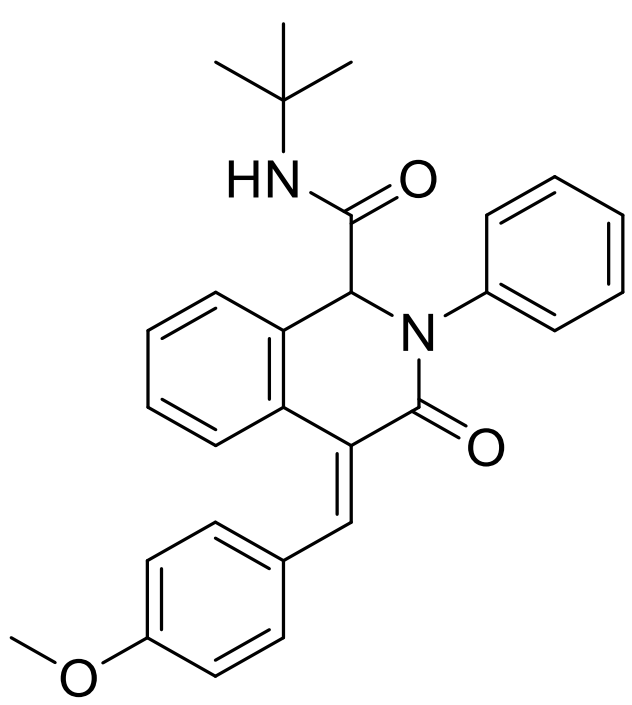 	Purification by column chromatography on silica gel using hexane/ethyl acetate (30-40% ethyl acetate) as the eluent afforded the individual *Z* and *E* isomers, which were further purified by trituration with diethyl ether.<br>**(*Z*)-*N*-(*tert*-Butyl)-4-(4-methoxybenzylidene)-3-oxo-2-phenyl-1,2,3,4-tetrahydroisoquinoline-1-carboxamide (*Z*)-28e**<br>Yield: 230 mg (55%, 0.7 mmol scale), white solid, melting point: 264-265 °C.<br>**^{1}H NMR** (500 MHz, CDCl$_3$) δ 7.76 (s, 1H), 7.49 – 7.33 (m, 8H), 7.33 – 7.27 (m, 2H), 7.20 – 7.14 (m, 1H), 6.81 (d, *J* = 8.8 Hz, 2H), 5.95 (s, 1H), 5.12 (s, 1H), 3.82 (s, 3H), 1.25 (s, 9H).<br>**^{13}C{^{1}H} NMR** (126 MHz, CDCl$_3$) δ 168.4, 166.7, 160.3, 142.0, 136.1, 132.8, 131.2, 130.7, 129.4, 128.3, 128.0, 127.91, 127.87, 127.4, 127.3, 127.2, 125.7, 114.1, 69.6, 55.4, 52.0, 28.7.<br>**(*E*)-*N*-(*tert*-Butyl)-4-(4-methoxybenzylidene)-3-oxo-2-phenyl-1,2,3,4-tetrahydroisoquinoline-1-carboxamide (*E*)-28e**<br>Yield: 80 mg (19%, 0.7 mmol scale), white solid, melting point: 130-131 °C.<br>**^{1}H NMR** (500 MHz, CDCl$_3$) δ 7.80 (d, *J* = 8.8 Hz, 2H), 7.66 – 7.60 (m, 1H), 7.47 – 7.32 (m, 7H), 7.31 – 7.26 (m, 1H), 7.11 (s, 1H), 6.87 (d, *J* = 8.9 Hz, 2H), 5.87 (s, 1H), 5.12 (s, 1H), 3.81 (s, 3H), 1.17 (s, 9H).<br>**^{13}C{^{1}H} NMR** (126 MHz, CDCl$_3$) δ 168.1, 165.2, 160.4, 142.0, 138.2, 135.7, 132.5, 131.7, 129.4, 129.2, 127.72, 127.69, 127.4, 127.2, 126.4, 125.9, 124.1, 113.7, 69.6, 55.4, 51.9, 28.5.<br>**HRMS** (ESI): m/z [M+Na]$^+$ for $C_{28}H_{28}N_2O_3$, calcd 463.1992, found 463.1991.

Purification by column chromatography on silica gel using hexane/ethyl acetate (30-40% ethyl acetate) as the eluent afforded the individual *Z* and *E* isomers, which were further purified by trituration with diethyl ether.

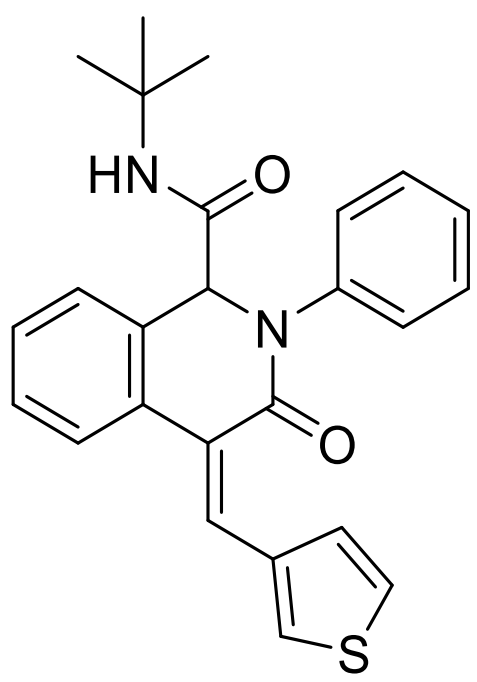

(*Z*)-*N*-(*tert*-Butyl)-3-oxo-2-phenyl-4-(thiophen-3-ylmethylene)-1,2,3,4-tetrahydroisoquinoline-1-carboxamide (*Z*)-28f

Yield: 200 mg (48%, 0.7 mmol scale), white solid, melting point: 214-215 °C.

^{1}H NMR (500 MHz, $CDCl_3$) δ 8.17 – 8.12 (m, 1H), 7.62 – 7.58 (m, 1H), 7.57 – 7.53 (m, 1H), 7.47 – 7.29 (m, 8H), 7.27 – 7.25 (m, 1H), 7.15 (s, 1H), 5.82 (s, 1H), 5.11 (s, 1H), 1.13 (s, 9H).

^{13}C{^{1}H} NMR (126 MHz, $CDCl_3$) δ 168.0, 165.0, 141.9, 136.2, 135.4, 131.8, 131.6, 130.4, 130.2, 129.5, 129.2, 127.9, 127.4, 126.5, 125.9, 125.2, 124.2, 69.8, 51.9, 28.5.

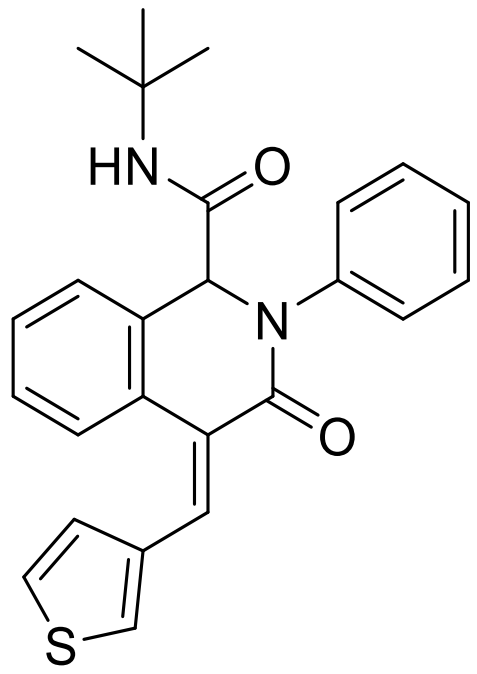

(*E*)-*N*-(*tert*-Butyl)-3-oxo-2-phenyl-4-(thiophen-3-ylmethylene)-1,2,3,4-tetrahydroisoquinoline-1-carboxamide (*E*)-28f

Yield: 92 mg (22%, 0.7 mmol scale), white solid, melting point: 253-255 °C.

^{1}H NMR (500 MHz, $CDCl_3$) δ 7.75 (s, 1H), 7.58 – 7.54 (m, 1H), 7.53 – 7.49 (m, 1H), 7.46 – 7.40 (m, 3H), 7.37 – 7.28 (m, 4H), 7.26 – 7.20 (m, 2H), 7.10 – 7.05 (m, 1H), 5.88 (s, 1H), 5.11 (s, 1H), 1.22 (s, 9H).

^{13}C{^{1}H} NMR (126 MHz, $CDCl_3$) δ 168.2, 166.7, 142.0, 136.5, 132.9, 130.8, 130.0, 129.4, 128.7, 128.6, 128.3, 128.2, 128.0, 127.4, 127.2, 127.1, 126.0, 125.8, 69.6, 52.0, 28.7.

HRMS (ESI): m/z $[M+H]^+$ for $C_{25}H_{24}N_2O_2S$, calcd 417.1631, found 417.1630.

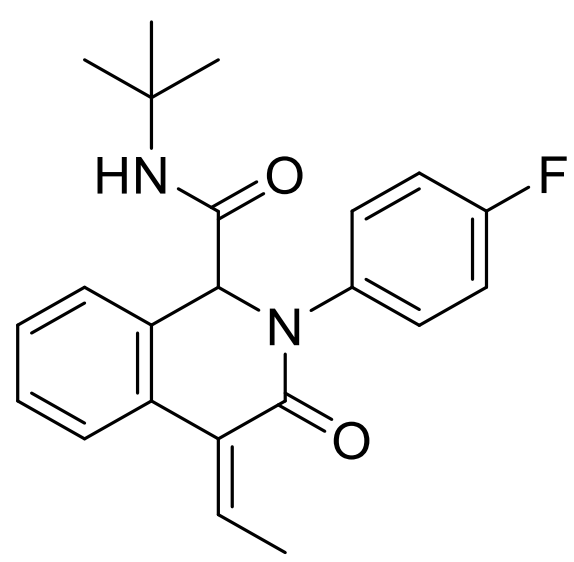<br>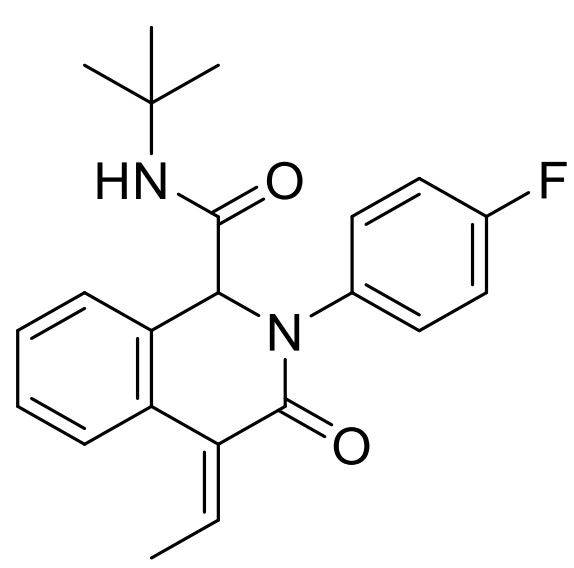	Purification by column chromatography on silica gel using hexane/ethyl acetate (30-40% ethyl acetate) as the eluent afforded the individual *Z* and *E* isomers, which were further purified by trituration with diethyl ether.<br>**(*Z*)-*N*-(*tert*-Butyl)-4-ethylidene-2-(4-fluorophenyl)-3-oxo-1,2,3,4-tetrahydroisoquinoline-1-carboxamide (*Z*)-28g**<br>Yield: 198 mg (54%, 0.7 mmol scale), white solid, melting point: 223-224 °C.<br>**^{1}H NMR** (500 MHz, $CDCl_3$) δ 7.52 – 7.47 (m, 1H), 7.42 – 7.36 (m, 1H), 7.34 – 7.28 (m, 4H), 7.13 – 7.06 (m, 2H), 6.66 (q, *J* = 7.4 Hz, 1H), 5.62 (s, 1H), 4.99 (s, 1H), 2.33 (d, *J* = 7.4 Hz, 3H), 1.24 (s, 9H).<br>**^{13}C{^{1}H} NMR** (126 MHz, $CDCl_3$) δ 168.2, 165.2, 161.4 (d, *J* = 246.8 Hz), 138.2, 137.8 (d, *J* = 3.4 Hz), 134.3, 130.1, 129.8, 129.1, 128.4 (d, *J* = 8.6 Hz), 127.6, 126.0, 124.1, 116.3 (d, *J* = 22.5 Hz), 69.7, 52.0, 28.6, 16.1.<br>**(*E*)-*N*-(*tert*-Butyl)-4-ethylidene-2-(4-fluorophenyl)-3-oxo-1,2,3,4-tetrahydroisoquinoline-1-carboxamide (*E*)-28g**<br>Yield: 18 mg (5%, 0.7 mmol scale), white solid, melting point: 270-272 °C.<br>**^{1}H NMR** (500 MHz, $CDCl_3$) δ 7.55 – 7.50 (m, 1H), 7.46 – 7.38 (m, 2H), 7.38 – 7.29 (m, 3H), 7.12 (q, *J* = 8.2 Hz, 1H), 7.11 – 7.06 (m, 2H), 5.61 (s, 1H), 5.00 (s, 1H), 2.17 (d, *J* = 7.6 Hz, 3H), 1.23 (s, 9H).<br>**^{13}C{^{1}H} NMR** (126 MHz, $CDCl_3$) δ 168.1, 166.9, 161.4 (d, *J* = 247.0 Hz), 138.0 (d, *J* = 3.1 Hz), 135.7, 132.0, 130.9, 130.8, 128.3, 128.2 (d, *J* = 8.3 Hz), 127.93, 127.90, 126.6, 116.2 (d, *J* = 22.7 Hz), 69.5, 52.0, 28.6, 15.6.<br>**HRMS** (ESI): m/z $[M+Na]^+$ for $C_{22}H_{23}FN_2O_2$ calcd 389.1636, found 389.1636.
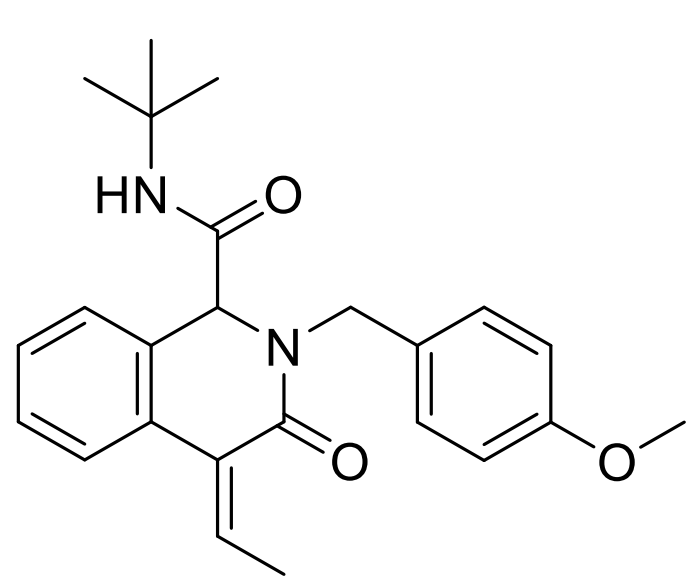	Purified by column chromatography on silica gel using hexane/ethyl acetate (5-15% ethyl acetate) as the eluent. Isolated as the pure *Z* isomer.<br>**(*Z*)-*N*-(*tert*-Butyl)-4-ethylidene-2-(4-methoxybenzyl)-3-oxo-1,2,3,4-tetrahydroisoquinoline-1-carboxamide (*Z*)-28h**<br>Yield: 148 mg (63%, 0.6 mmol scale), white solid, melting point: 135-137 °C.

	**^{1}H NMR** (400 MHz, $CDCl_3$) δ 7.43 – 7.39 (m, 1H), 7.34 – 7.21 (m, 5H), 6.83 (d, *J* = 8.7 Hz, 2H), 6.55 (q, *J* = 7.4 Hz, 1H), 5.37 (s, 1H), 5.07 (d, *J* = 14.6 Hz, 1H), 4.72 (s, 1H), 4.41 (d, *J* = 14.6 Hz, 1H), 3.77 (s, 3H), 2.34 (d, *J* = 7.4 Hz, 3H), 1.10 (s, 9H).<br>**^{13}C{^{1}H} NMR** (101 MHz, $CDCl_3$) δ 168.1, 165.0, 159.4, 136.7, 134.0, 130.11, 130.09, 129.7, 128.8, 128.7, 127.5, 126.5, 123.7, 114.4, 65.8, 55.4, 51.5, 49.3, 28.5, 16.0.<br>**HRMS** (ESI): m/z $[M+Na]^+$ for $C_{24}H_{28}N_2O_3$, calcd 415.1992, found 415.1998.
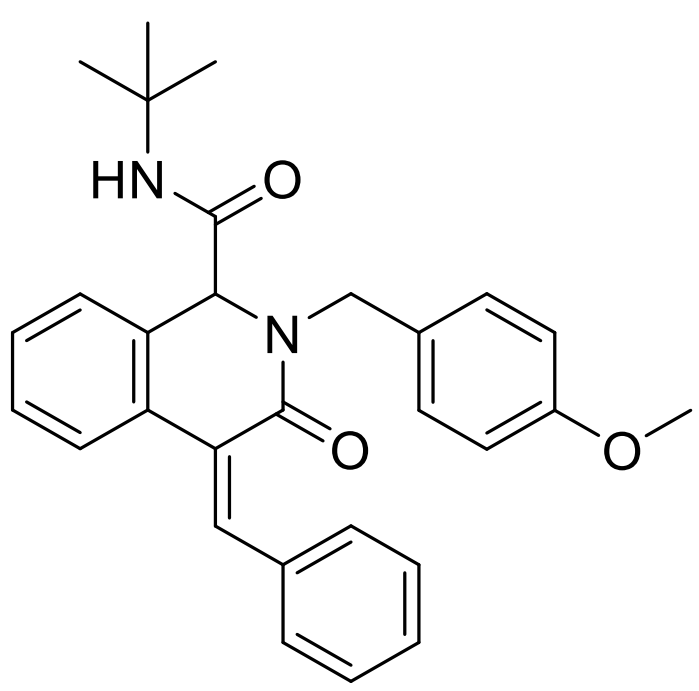<br>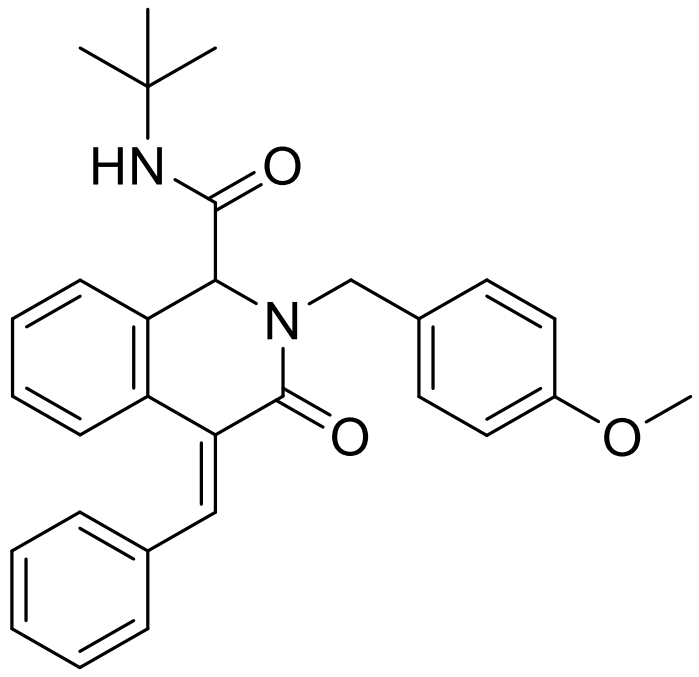	Purification by column chromatography on silica gel using hexane/ethyl acetate (40% ethyl acetate) as the eluent afforded a mixture of *Z* and *E* isomers. Yield for the mixture: 360 mg (88%, 0.9 mmol scale). Subsequent trituration with diethyl ether and methanol resulted in the removal of the *E* isomer, affording the pure *Z* isomer.<br>**(*Z*)-4-Benzylidene-*N*-(*tert*-butyl)-2-(4-methoxybenzyl)-3-oxo-1,2,3,4-tetrahydroisoquinoline-1-carboxamide (*Z*)-28i**<br>Yield: 261 mg (64%, 0.9 mmol scale), white solid, melting point: 181-184 °C.<br>**^{1}H NMR** (500 MHz, $CDCl_3$) δ 7.68 (d, *J* = 7.5 Hz, 2H), 7.62 – 7.57 (m, 1H), 7.42 – 7.36 (m, 3H), 7.36 – 7.26 (m, 5H), 7.13 (s, 1H), 6.84 (d, *J* = 8.2 Hz, 2H), 5.49 (s, 1H), 5.00 (d, *J* = 14.5 Hz, 1H), 4.80 (s, 1H), 4.58 (d, *J* = 14.5 Hz, 1H), 3.78 (s, 3H), 1.05 (s, 9H).<br>**^{13}C{^{1}H} NMR** (126 MHz, $CDCl_3$) δ 167.8, 164.9, 159.4, 136.9, 135.2, 134.9, 131.3, 130.2, 129.9, 129.5, 129.0, 128.8, 128.6, 128.2, 128.0, 126.7, 123.9, 114.4, 65.8, 55.4, 51.6, 49.8, 28.3.<br>**HRMS** (ESI): m/z $[M+H]^+$ for $C_{29}H_{30}N_2O_3$, calcd 455.2329, found 455.2327.

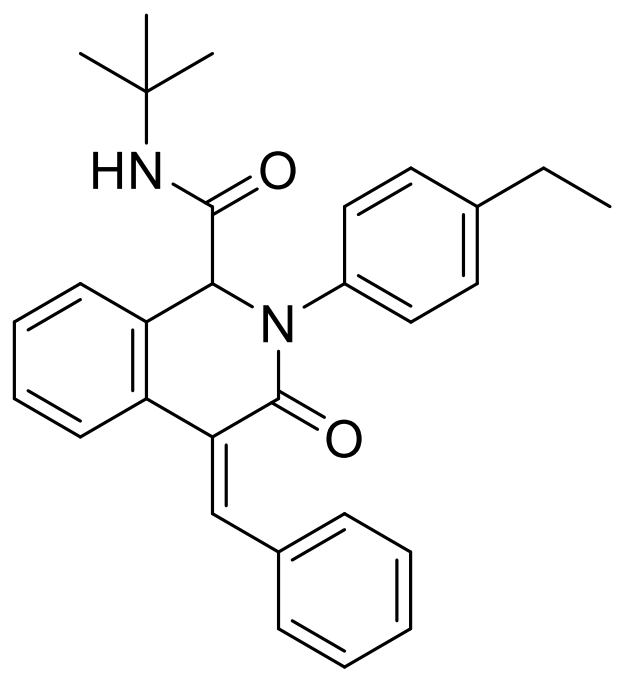<br>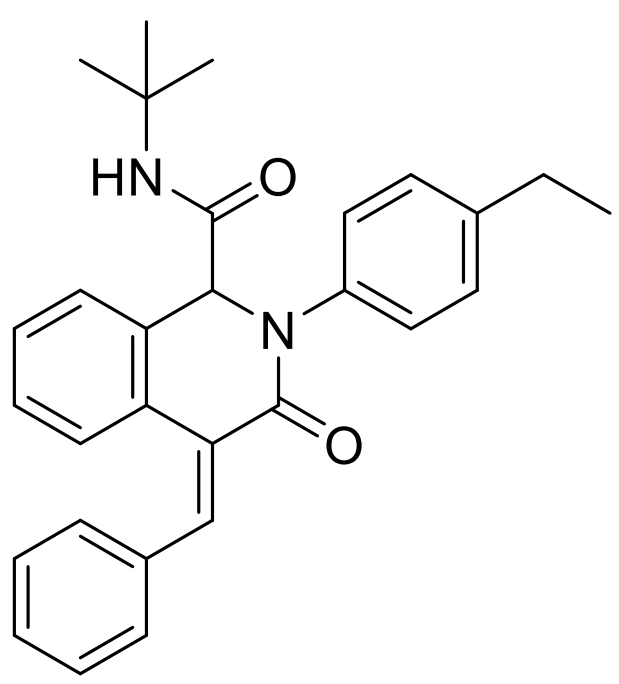	Purified by column chromatography on silica gel using hexane/ethyl acetate (5-15% ethyl acetate) as the eluent. Overall yield for the *Z*/*E* mixture of isomers: 364 mg (83%, 1.0 mmol scale).<br>**(*Z*)-4-Benzylidene-*N*-(*tert*-butyl)-2-(4-ethylphenyl)-3-oxo-1,2,3,4-tetrahydroisoquinoline-1-carboxamide (*Z*)-28j**<br>Yield: 224 mg (51%, 1.0 mmol scale), white solid, melting point: 192-194 °C.<br>**^{1}H NMR** (400 MHz, $CDCl_3$) δ 7.76 (d, *J* = 7.2 Hz, 2H), 7.68 – 7.63 (m, 1H), 7.48 – 7.42 (m, 1H), 7.41 – 7.21 (m, 9H), 7.18 (s, 1H), 5.87 (s, 1H), 5.11 (s, 1H), 2.65 (q, *J* = 7.6 Hz, 2H), 1.23 (t, *J* = 7.6 Hz, 3H), 1.19 (s, 9H).<br>**^{13}C{^{1}H} NMR** (151 MHz, $CDCl_3$) δ 168.3, 168.1, 166.5, 164.9, 143.40, 143.37, 139.5, 137.8, 136.0, 135.33, 135.30, 135.0, 132.9, 131.8, 130.5, 130.3, 130.0, 129.9, 129.4, 129.1, 128.88, 128.86, 128.84, 128.7, 128.4, 128.3, 128.2, 128.0, 127.8, 127.1, 126.4, 125.8, 125.7, 124.2, 69.69, 69.66, 52.0, 51.9, 28.7, 28.5, 15.6, 15.5.<br>**HRMS** (ESI): m/z $[M+H]^+$ for $C_{29}H_{30}N_2O_2$, calcd 439.2380, found 439.2371.
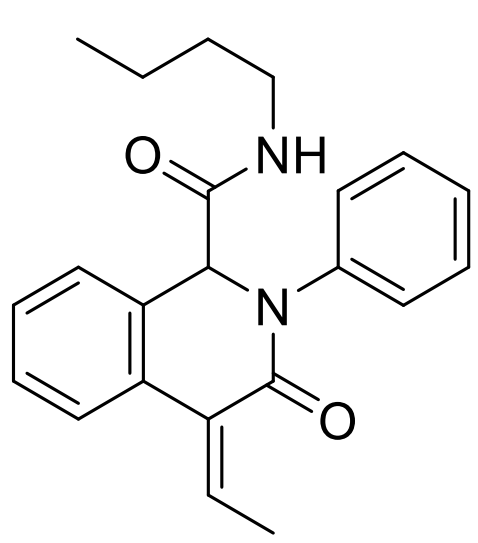<br>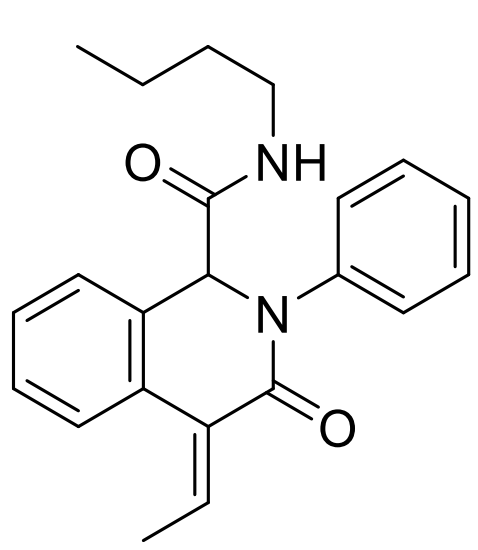	Purified by column chromatography on silica gel using hexane/ethyl acetate (5-20% ethyl acetate) as the eluent. Overall yield for the *Z*/*E* mixture of isomers: 195 mg (56%, 1.0 mmol scale).<br>**(*Z*)-*N*-Butyl-4-ethylidene-3-oxo-2-phenyl-1,2,3,4-tetrahydroisoquinoline-1-carboxamide (*Z*)-28k**<br>Yield: 129 mg (37%, 1.0 mmol scale), white solid, melting point: 203-205 °C.<br>**^{1}H NMR** (400 MHz, $CDCl_3$) δ 7.51 – 7.46 (m, 1H), 7.44 – 7.27 (m, 8H), 6.65 (q, *J* = 7.4 Hz, 1H), 5.89 (br s, 1H), 5.16 (s, 1H), 3.30 – 3.13 (m, 2H), 2.34 (d, *J* = 7.4 Hz, 3H), 1.41 – 1.34 (m, 2H), 1.25 – 1.16 (m, 2H), 0.85 (t, *J* = 7.3 Hz, 3H).<br>**^{13}C{^{1}H} NMR** (101 MHz, $CDCl_3$) δ 169.2, 165.3, 141.9, 138.2, 134.4, 130.2, 129.9, 129.4, 129.0, 127.5, 127.3, 126.3, 124.0, 68.8, 39.7, 31.4, 19.9, 16.1, 13.7.<br>**HRMS** (ESI): m/z $[M+Na]^+$ for $C_{22}H_{24}N_2O_2$, calcd 371.1730, found 371.1731.

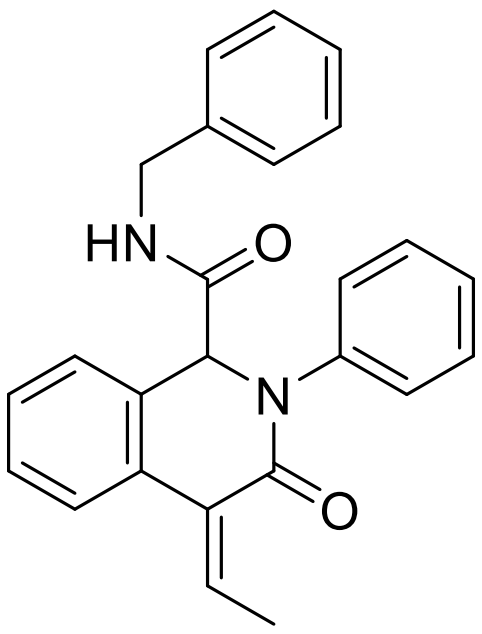<br>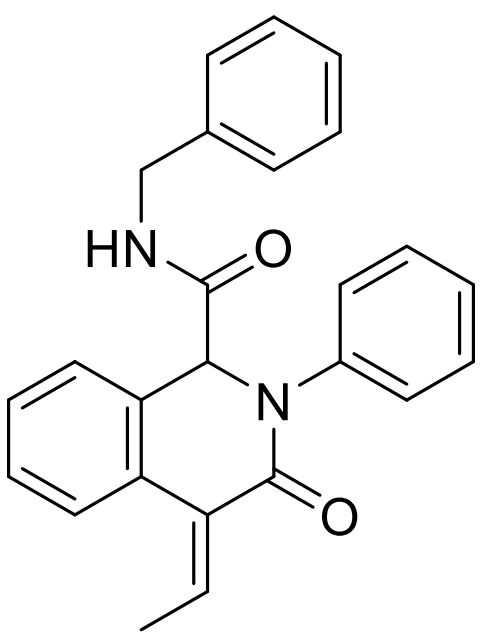	The reaction was run for 48 hours. Purified by column chromatography on silica gel using hexane/ethyl acetate (20-40% ethyl acetate) as the eluent. Obtained as a mixture of *Z* and *E* isomers, but only the signals from the major *Z* isomer are listed below. Yield: 224 mg (65%, 0.9 mmol scale), white solid, melting point: 161-162 °C.<br>**(*Z*)-*N*-Benzyl-4-ethylidene-3-oxo-2-phenyl-1,2,3,4-tetrahydroisoquinoline-1-carboxamide (*Z*)-28l**<br>**^{1}H NMR** (500 MHz, $CDCl_3$) δ 7.50 – 7.47 (m, 1H), 7.42 – 7.35 (m, 4H), 7.33 – 7.24 (m, 7H), 7.09 – 7.05 (m, 2H), 6.61 (q, *J* = 7.3 Hz, 1H), 6.23 (t, *J* = 5.8 Hz, 1H), 5.22 (s, 1H), 4.45 (dd, *J* = 14.8, 6.1 Hz, 1H), 4.32 (dd, *J* = 14.8, 5.3 Hz, 1H), 2.21 (d, *J* = 7.4 Hz, 3H).<br>**^{13}C{^{1}H} NMR** (126 MHz, $CDCl_3$) δ 169.3, 165.8, 141.7, 137.8, 137.7, 134.4, 129.9, 129.5, 129.2, 128.8, 128.5, 127.33, 127.32, 127.30, 127.29, 126.6, 126.1, 123.8, 68.2, 43.5, 16.0.<br>**HRMS** (ESI): m/z $[M+H]^+$ for $C_{25}H_{22}N_2O_2$, calcd 383.1754, found 383.1761.

Characterization of synthesized compounds 29

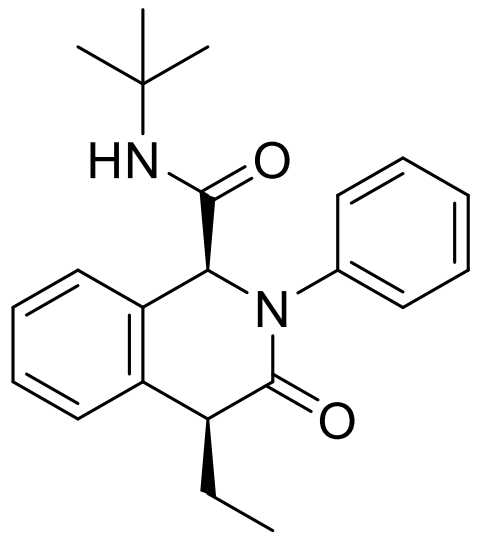	**(±)-(1*S*,4*S*)-*N*-(*tert*-Butyl)-4-ethyl-3-oxo-2-phenyl-1,2,3,4-tetrahydroisoquinoline-1-carboxamide *cis*-29a**<br>Conducted according to general procedure **A** using the pure *E* isomer of **28a** and purified by trituration with diethyl ether. Yield: 52 mg (88%, dr 93:7, 0.17 mmol scale).<br>Conducted according to general procedure **A** using the pure *Z* isomer of **28a** and purified by trituration with diethyl ether. Yield: 272 mg (97%, dr 94:6, 0.8 mmol scale).<br>Conducted according to procedure **D** using the pure *Z* isomer of **28a** and purified by trituration with diethyl ether. Yield: 1360 mg (97%, dr >95:5, 4.0 mmol scale), white solid, melting point: 248-249 °C.<br>**^{1}H NMR** (500 MHz, $CDCl_3$) δ 7.42 – 7.34 (m, 4H), 7.33 – 7.27 (m, 4H), 7.22 – 7.17 (m, 1H), 5.82 (s, 1H), 5.20 (s, 1H), 3.56 (dd, *J* = 8.5, 6.9 Hz, 1H), 2.25 – 2.12 (m, 1H), 2.01 – 1.88 (m, 1H), 1.23 (s, 9H), 1.12 (t, *J* = 7.4 Hz, 3H).<br>**^{13}C{^{1}H} NMR** (126 MHz, $CDCl_3$) δ 172.1, 168.4, 142.2, 136.4, 130.3, 129.5, 128.6, 128.3, 127.6, 127.2, 127.0, 126.9, 69.3, 51.9, 49.8, 30.0, 28.5, 12.7.<br>**HRMS** (ESI): m/z $[M+Na]^+$ for $C_{22}H_{26}N_2O_2$, calcd 373.1886, found 373.1882.
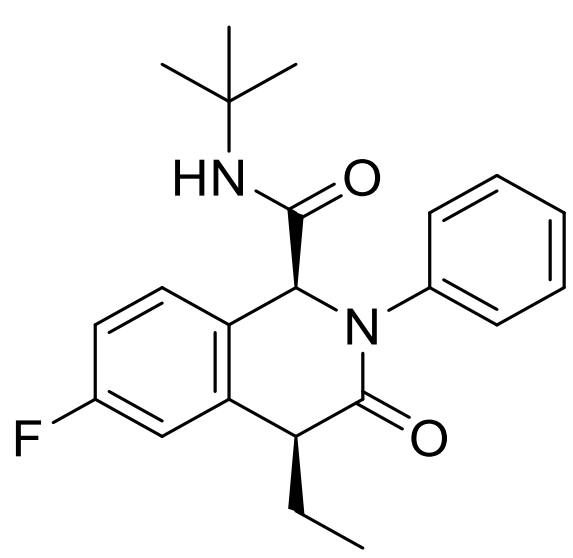	**(±)-(1*S*,4*S*)-*N*-(*tert*-Butyl)-4-ethyl-6-fluoro-3-oxo-2-phenyl-1,2,3,4-tetrahydroisoquinoline-1-carboxamide *cis*-29b**<br>Conducted according to general procedure **C** using the mixture of *Z* and *E* isomers of **28b**. Yield: 103 mg (93%, dr 93:7, 0.3 mmol scale).<br>Conducted according to general procedure **A** using the mixture of *Z* and *E* isomers of **28b** and purified by trituration with diethyl ether. Yield: 101 mg (91%, dr >95:5, 0.3 mmol scale), white solid, melting point: 275-277 °C.<br>**^{1}H NMR** (500 MHz, $CDCl_3$) δ 7.46 – 7.40 (m, 2H), 7.40 – 7.35 (m, 1H), 7.34 – 7.29 (m, 3H), 7.04 – 6.99 (m, 1H), 6.94 – 6.88 (m, 1H), 5.75 (s, 1H), 5.18 (s, 1H), 3.55 (t, *J* = 7.7 Hz, 1H), 2.23 – 2.13 (m, 1H), 2.01 – 1.91 (m, 1H), 1.25 (s, 9H), 1.14 (t, *J* = 7.5 Hz, 3H).

	**$^{13}C\{^1H\}$ NMR** (126 MHz, $CDCl_3$) δ 171.5, 168.2, 162.5 (d, *J* = 247.7 Hz), 141.9, 138.8 (d, *J* = 7.7 Hz), 129.6, 128.8 (d, *J* = 8.4 Hz), 127.7, 127.1, 126.1 (d, *J* = 2.9 Hz), 114.9 (d, *J* = 21.7 Hz), 114.3 (d, *J* = 21.9 Hz), 68.7, 52.0, 49.6, 30.1, 28.5, 12.6.<br>**HRMS** (ESI): m/z $[M+H]^+$ for $C_{22}H_{25}FN_2O_2$, calcd 369.1973, found 369.1971.
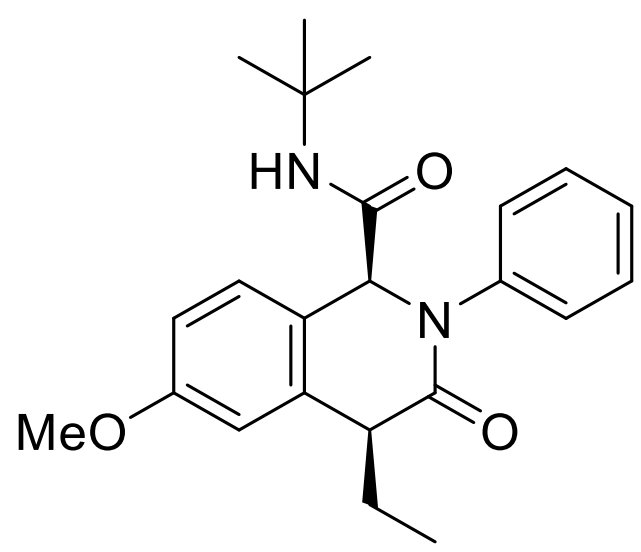	**(±)-(1*S*,4*S*)-*N*-(*tert*-Butyl)-4-ethyl-6-methoxy-3-oxo-2-phenyl-1,2,3,4-tetrahydroisoquinoline-1-carboxamide *cis*-29c**<br>Conducted according to general procedure **B** using the mixture of *Z* and *E* isomers of **28c** and purified by trituration with diethyl ether. Yield: 90 mg (95%, dr >95:5, 0.25 mmol scale), white solid, melting point: 254-255 °C.<br>**^1H NMR** (400 MHz, $CDCl_3$) δ 7.44 – 7.38 (m, 2H), 7.33 – 7.27 (m, 4H), 6.88 – 6.83 (m, 1H), 6.73 – 6.68 (m, 1H), 5.73 (s, 1H), 5.15 (s, 1H), 3.84 (s, 3H), 3.53 (dd, *J* = 8.3, 7.0 Hz, 1H), 2.24 – 2.11 (m, 1H), 2.02 – 1.89 (m, 1H), 1.24 (s, 9H), 1.14 (t, *J* = 7.5 Hz, 3H).<br>**$^{13}C\{^1H\}$ NMR** (101 MHz, $CDCl_3$) δ 172.0, 168.7, 159.6, 142.3, 138.0, 129.5, 128.0, 127.5, 127.2, 122.6, 113.3, 113.0, 68.8, 55.5, 51.9, 50.0, 29.9, 28.6, 12.7.<br>**HRMS** (ESI): m/z $[M+H]^+$ for $C_{23}H_{28}N_2O_3$, calcd 381.2173, found 381.2173.
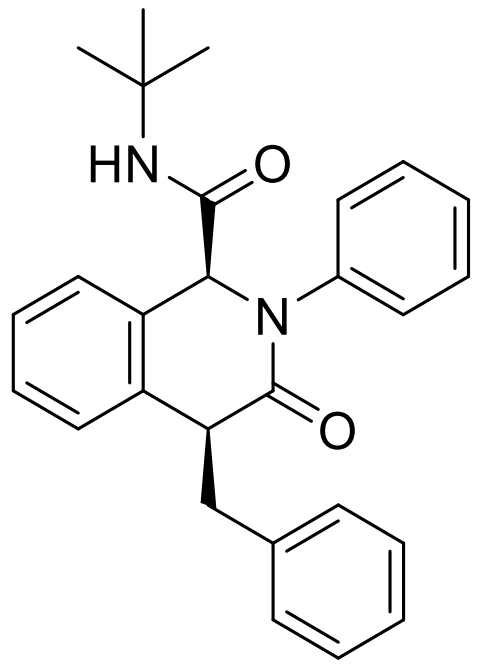	**(±)-(1*S*,4*S*)-4-Benzyl-*N*-(*tert*-butyl)-3-oxo-2-phenyl-1,2,3,4-tetrahydroisoquinoline-1-carboxamide *cis*-29d**<br>Conducted according to general procedure **A** using the mixture of *Z* and *E* isomers of **28d** and purified by column chromatography on silica gel using hexane/ethyl acetate (35% ethyl acetate) as the eluent, followed by trituration with diethyl ether. Yield: 139 mg (96%, dr 91:9, 0.35 mmol scale), white solid, melting point: 230-232 °C.<br>**^1H NMR** ^1H NMR (500 MHz, $CDCl_3$) δ 7.46 – 7.40 (m, 2H), 7.38 – 7.30 (m, 4H), 7.29 – 7.21 (m, 4H), 7.09 – 7.02 (m, 3H), 6.37 – 6.31 (m, 1H), 5.71 (s, 1H), 5.21 (s, 1H), 3.89 (dd, *J* = 11.0, 4.6 Hz, 1H), 3.62 (dd, *J* = 12.9, 4.6 Hz, 1H), 3.19 (dd, *J* = 13.1, 11.0 Hz, 1H), 1.29 (s, 9H).

$^{13}C\{^{1}H\}$ NMR ^{13}C NMR (126 MHz, $CDCl_3$) δ 171.9, 168.4, 142.2, 138.9, 134.9, 130.4, 130.0, 129.5, 129.2, 128.3, 127.6, 127.5, 127.3, 127.0, 126.6, 126.2, 69.3, 51.9, 50.5, 42.5, 28.5.

HRMS (ESI): m/z $[M+H]^+$ for $C_{27}H_{28}N_2O_2$, calcd 413.2224, found 413.2204.

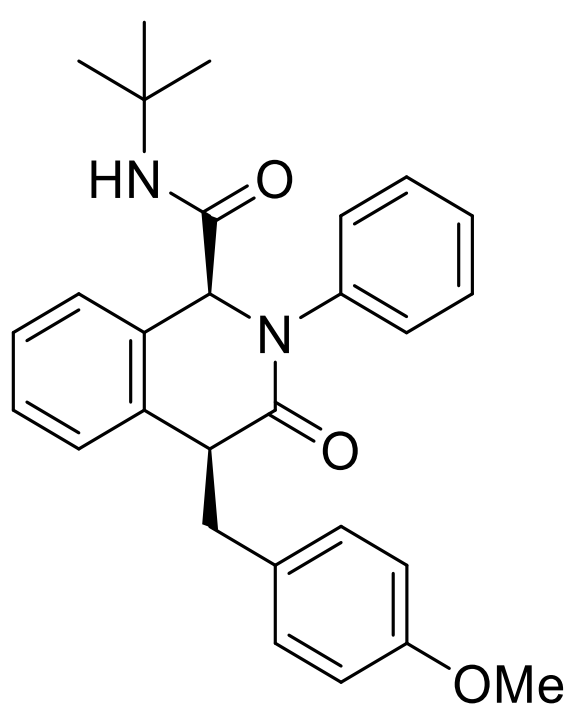

(±)-(1*S*,4*S*)-*N*-(*tert*-Butyl)-4-(4-methoxybenzyl)-3-oxo-2-phenyl-1,2,3,4-tetrahydroisoquinoline-1-carboxamide *cis*-29e

Conducted according to general procedure **C** using the mixture of *Z* and *E* isomers of **28e**. Yield: 86 mg (97%, dr 89:11, 0.2 mmol scale)

Conducted according to general procedure **C** using the pure *Z* isomer of **28e** and purified by column chromatography on silica gel using hexane/ethyl acetate (20-30% ethyl acetate). Yield: 109 mg (82%, dr 94:6, 0.3 mmol scale).

Conducted according to general procedure **B** using the pure *Z* isomer of **28e** and purified by trituration with diethyl ether. Yield of the pure *cis* diastereomer: 94 mg (71%, 0.3 mmol scale), white solid, melting point: 228-230 °C.

^{1}H NMR (500 MHz, $CDCl_3$) δ 7.46 – 7.40 (m, 2H), 7.38 – 7.30 (m, 4H), 7.29 – 7.24 (m, 1H), 7.14 – 7.06 (m, 1H), 6.96 (d, *J* = 8.6 Hz, 2H), 6.79 (d, *J* = 8.6 Hz, 2H), 6.43 – 6.37 (m, 1H), 5.67 (s, 1H), 5.21 (s, 1H), 3.85 (dd, *J* = 10.9, 4.7 Hz, 1H), 3.79 (s, 3H), 3.55 (dd, *J* = 13.2, 4.7 Hz, 1H), 3.13 (dd, *J* = 13.1, 10.9 Hz, 1H), 1.28 (s, 9H).

$^{13}C\{^{1}H\}$ NMR (126 MHz, $CDCl_3$) δ 171.8, 168.4, 158.3, 142.2, 135.0, 131.0, 130.8, 130.3, 129.5, 129.3, 127.7, 127.6, 127.2, 127.0, 126.3, 113.7, 69.4, 55.3, 52.0, 50.8, 41.5, 28.6.

HRMS (ESI): m/z $[M+H]^+$ for $C_{28}H_{30}N_2O_3$, calcd 443.2329, found 443.2347.

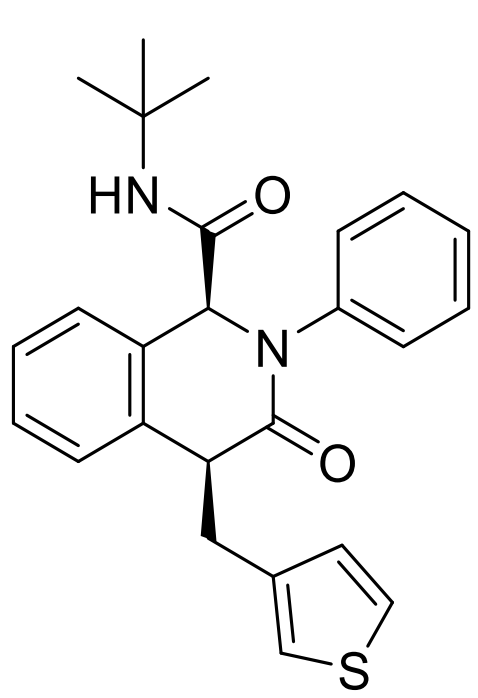

(±)-(1*S*,4*S*)-*N*-(*tert*-Butyl)-3-oxo-2-phenyl-4-(thiophen-3-ylmethyl)-1,2,3,4-tetrahydroisoquinoline-1-carboxamide *cis*-29f

Conducted according to procedure **E** using the pure *Z* isomer of **28f** and purified by column chromatography on silica gel using hexane/ethyl acetate (20-30% ethyl acetate). Yield: 47 mg (31%, 58% b.r.s.m., dr 93:7, 0.36 mmol scale), white solid.

^{1}H NMR (400 MHz, $CDCl_3$) δ 7.42 – 7.22 (m, 8H), 7.16 – 7.10 (m, 1H), 6.90 (dd, *J* = 4.9, 1.1 Hz, 1H), 6.83 – 6.77 (m, 1H), 6.46 (d, *J* = 7.6 Hz,

	1H), 5.79 (s, 1H), 5.17 (s, 1H), 3.87 (dd, *J* = 10.7, 4.7 Hz, 1H), 3.60 (dd, *J* = 13.5, 4.7 Hz, 1H), 3.35 (dd, *J* = 13.5, 10.7 Hz, 1H), 1.25 (s, 9H).<br>**^{13}C{^{1}H} NMR** (101 MHz, $CDCl_3$) δ 171.7, 168.4, 142.1, 139.2, 135.1, 130.3, 129.5, 129.02, 128.98, 127.8, 127.7, 127.3, 127.0, 126.1, 125.5, 123.0, 69.5, 52.0, 49.8, 36.9, 28.6.<br>**HRMS** (ESI): m/z $[M+Na]^+$ for $C_{25}H_{26}N_2O_2S$, calcd 441.1607, found 441.1597.
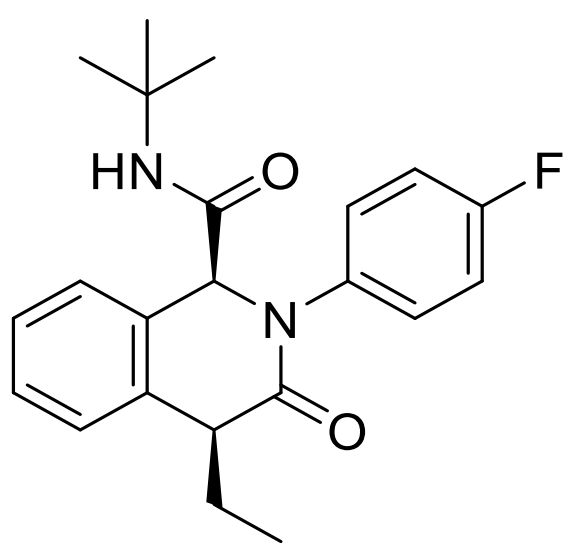	**(±)-(1*S*,4*S*)-*N*-(*tert*-Butyl)-4-ethyl-2-(4-fluorophenyl)-3-oxo-1,2,3,4-tetrahydroisoquinoline-1-carboxamide *cis*-29g**<br>Conducted according to general procedure **C** using the mixture of *Z* and *E* isomers of **28g**. Yield: 93 mg (84%, dr >95:5, 0.3 mmol scale).<br>Conducted according to general procedure **B** using the pure *Z* isomer of **28g** and purified by trituration with diethyl ether. Yield: 137 mg (93%, dr >95:5, 0.4 mmol scale), white solid, melting point: 219-221 °C.<br>**^{1}H NMR** (500 MHz, $CDCl_3$) δ 7.40 – 7.28 (m, 5H), 7.24 – 7.19 (m, 1H), 7.12 – 7.06 (m, 2H), 5.62 (s, 1H), 5.12 (s, 1H), 3.57 (dd, *J* = 8.6, 6.8 Hz, 1H), 2.24 – 2.12 (m, 1H), 1.99 – 1.86 (m, 1H), 1.24 (s, 9H), 1.11 (t, *J* = 7.4 Hz, 3H).<br>**^{13}C{^{1}H} NMR** (126 MHz, $CDCl_3$) δ 172.3, 168.2, 161.6 (d, *J* = 247.1 Hz), 138.2 (d, *J* = 3.3 Hz), 136.6, 130.2, 129.3 (d, *J* = 8.5 Hz), 128.8, 128.5, 127.0, 126.4, 116.3 (d, *J* = 22.6 Hz), 69.4, 52.0, 49.7, 30.0, 28.5, 12.7.<br>**HRMS** (ESI): m/z $[M+Na]^+$ for $C_{22}H_{25}FN_2O_2$, calcd 391.1792, found 391.1792.
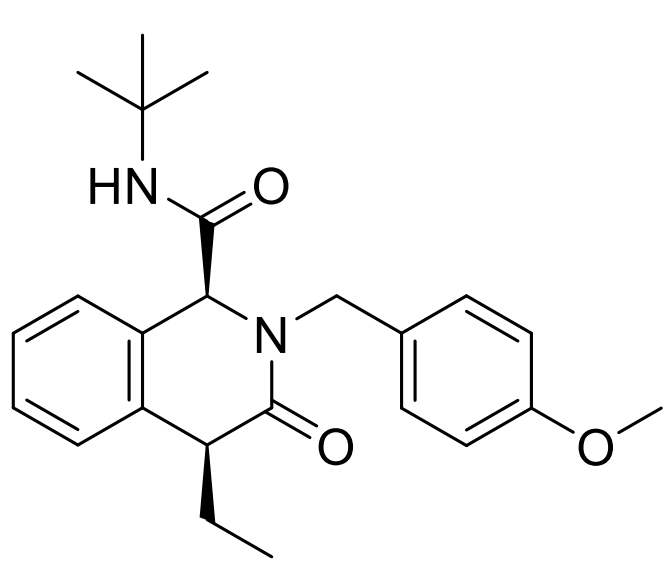	**(±)-(1*S*,4*S*)-*N*-(*tert*-Butyl)-4-ethyl-2-(4-methoxybenzyl)-3-oxo-1,2,3,4-tetrahydroisoquinoline-1-carboxamide *cis*-29h**<br>Conducted according to general procedure **C** using the pure *Z* isomer of **28h**. Yield: 75 mg (95%, dr >95:5, 0.2 mmol scale), white solid, melting point: 70-80 °C.<br>**^{1}H NMR** (400 MHz, $CDCl_3$) δ 7.35 – 7.27 (m, 2H), 7.26 – 7.23 (m, 1H), 7.20 (d, *J* = 8.4 Hz, 2H), 7.16 – 7.11 (m, 1H), 6.82 (d, *J* = 8.2 Hz, 2H), 5.51 (s, 1H), 5.29 (d, *J* = 14.7 Hz, 1H), 4.77 (s, 1H), 4.26 (d, *J* = 14.7 Hz, 1H), 3.77 (s, 3H), 3.53 (t, *J* = 7.7 Hz, 1H), 2.07 – 1.94 (m, 1H), 1.84 – 1.72 (m, 1H), 1.21 (s, 9H), 1.06 (t, *J* = 7.4 Hz, 3H).

$^{13}C\{^1H\}$ NMR (151 MHz, $CDCl_3$) δ 172.1, 168.5, 159.4, 136.2, 130.1, 130.0, 128.6, 128.3, 127.3, 127.0, 114.4, 64.9, 55.4, 51.8, 49.9, 49.3, 29.6, 28.5, 12.5.

HRMS (ESI): m/z $[M+Na]^+$ for $C_{24}H_{30}N_2O_3$, calcd 417.2149, found 417.2144.

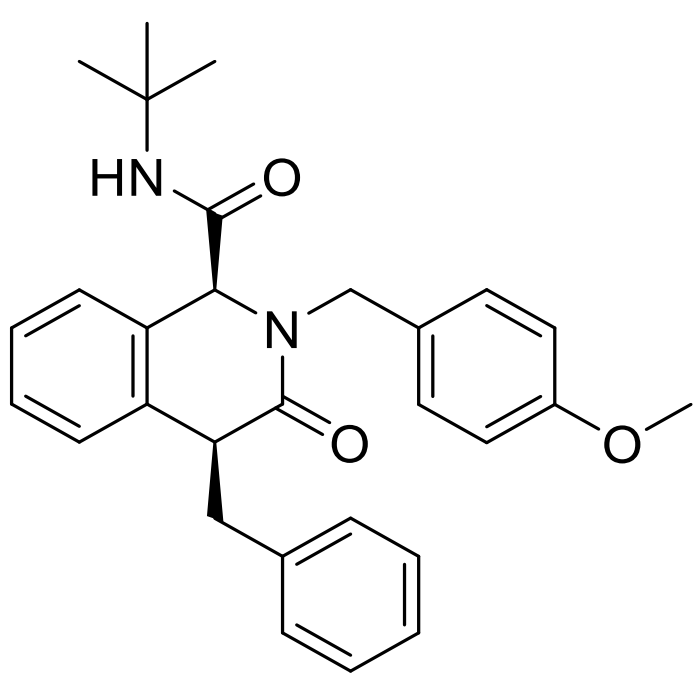

(±)-(1*S*,4*S*)-4-Benzyl-*N*-(*tert*-butyl)-2-(4-methoxybenzyl)-3-oxo-1,2,3,4-tetrahydroisoquinoline-1-carboxamide *cis*-29i

Conducted according to general procedure **C** using the mixture of *Z* and *E* isomers of **28i**. Yield: 125 mg (91%, dr 92:8, 0.3 mmol scale).

Conducted according to general procedure **B** using the mixture of *Z* and *E* isomers of **28i** and purified by column chromatography on silica gel using dichloromethane/ethyl acetate (5% ethyl acetate) as the eluent. Yield of the pure *cis* diastereomer: 152 mg (83%, 0.4 mmol scale), white solid, melting point: 179-180 °C.

^1H NMR (400 MHz, $CDCl_3$) δ 7.24 – 7.18 (m, 1H), 7.15 – 7.02 (m, 6H), 6.89 (d, *J* = 7.7 Hz, 2H), 6.89 – 6.85 (m, 1H), 6.68 (d, *J* = 8.6 Hz, 2H), 6.19 – 6.12 (m, 1H), 5.79 (s, 1H), 5.19 (d, *J* = 14.8 Hz, 1H), 4.76 (s, 1H), 4.19 (d, *J* = 14.9 Hz, 1H), 3.71 (dd, *J* = 11.0, 4.6 Hz, 1H), 3.60 (s, 3H), 3.43 (dd, *J* = 13.0, 4.6 Hz, 1H), 2.89 (dd, *J* = 13.0, 11.0 Hz, 1H), 1.18 (s, 9H).

$^{13}C\{^1H\}$ NMR (101 MHz, $CDCl_3$) δ 171.8, 168.3, 159.2, 138.6, 134.7, 130.2, 129.8, 129.7, 128.9, 128.3, 128.2, 127.4, 126.8, 126.5, 126.4, 114.2, 64.6, 55.2, 51.7, 50.2, 49.7, 42.1, 28.5.

HRMS (ESI): m/z $[M+H]^+$ for $C_{29}H_{32}N_2O_3$, calcd 457.2486, found 457.2491.

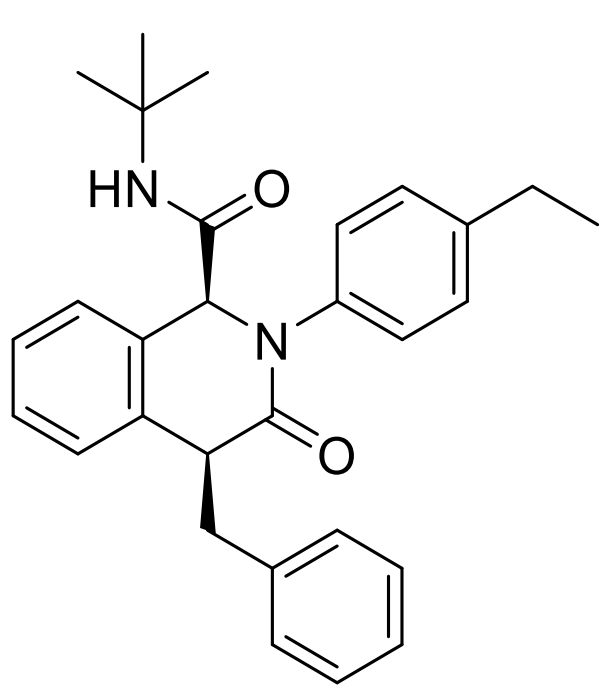

(±)-(1*S*,4*S*)-4-Benzyl-*N*-(*tert*-butyl)-2-(4-ethylphenyl)-3-oxo-1,2,3,4-tetrahydroisoquinoline-1-carboxamide *cis*-29j

Conducted according to general procedure **C** using the mixture of *Z* and *E* isomers of **28j**. Yield: 124 mg (94%, dr >95:5, 0.3 mmol scale), white solid, melting point: 139-141 °C.

^1H NMR (400 MHz, $CDCl_3$) δ 7.39 – 7.34 (m, 1H), 7.29 – 7.22 (m, 8H), 7.11 – 7.02 (m, 3H), 6.40 – 6.31 (m, 1H), 5.80 (s, 1H), 5.21 (s, 1H), 3.90 (dd, *J* = 10.9, 4.6 Hz, 1H), 3.63 (dd, *J* = 12.9, 4.6 Hz, 1H), 3.21 (dd, *J* = 12.8, 11.1 Hz, 1H), 2.68 (q, *J* = 7.6 Hz, 2H), 1.31 (s, 9H), 1.26 (t, *J* = 7.6 Hz, 3H).

	**$^{13}C\{^{1}H\}$ NMR** (101 MHz, $CDCl_3$) δ 171.8, 168.5, 143.7, 139.8, 138.8, 135.0, 130.5, 130.1, 129.2, 129.0, 128.4, 127.6, 126.99, 126.96, 126.7, 126.5, 69.6, 52.1, 50.7, 42.3, 28.7, 28.6, 15.5.<br>**HRMS** (ESI): m/z $[M+Na]^+$ for $C_{29}H_{32}N_2O_2$, calcd 463.2356, found 463.2352.
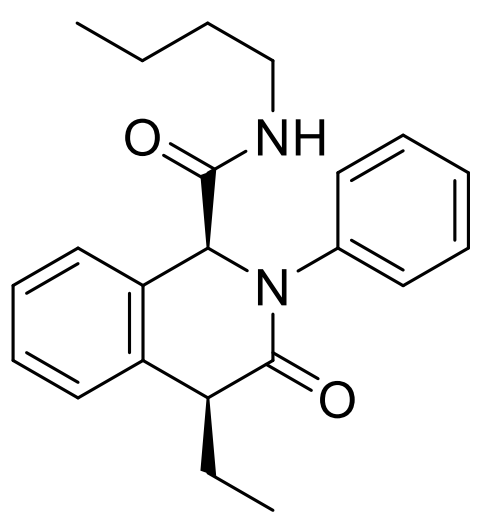	**(±)-(1*S*,4*S*)-*N*-Butyl-4-ethyl-3-oxo-2-phenyl-1,2,3,4-tetrahydroisoquinoline-1-carboxamide *cis*-29k**<br>Conducted according to general procedure **C** using the mixture of *Z* and *E* isomers of **28k**. Yield: 62 mg (88%, dr 90:10, 0.2 mmol scale), white solid, melting point: 161-163 °C.<br>**^{1}H NMR** (400 MHz, $CDCl_3$) δ 7.37 – 7.28 (m, 4H), 7.28 – 7.21 (m, 4H), 7.20 – 7.15 (m, 1H), 6.34 (t, *J* = 5.5 Hz, 1H), 5.24 (s, 1H), 3.49 (dd, *J* = 8.6, 6.8 Hz, 1H), 3.16 – 3.01 (m, 2H), 2.31 – 2.18 (m, 1H), 2.03 – 1.90 (m, 1H), 1.35 – 1.24 (m, 2H), 1.19 – 1.12 (m, 2H), 1.10 (t, *J* = 7.5 Hz, 3H), 0.81 (t, *J* = 7.3 Hz, 3H).<br>**$^{13}C\{^{1}H\}$ NMR** (101 MHz, $CDCl_3$) δ 172.4, 169.3, 158.9, 142.2, 136.6, 130.1, 129.4, 128.7, 128.3, 127.6, 127.4, 126.9, 126.6, 68.8, 49.7, 39.7, 31.3, 30.3, 19.9, 13.7, 12.8.<br>**HRMS** (ESI): m/z $[M+H]^+$ for $C_{22}H_{26}N_2O_2$, calcd 351.2067, found 351.2067.
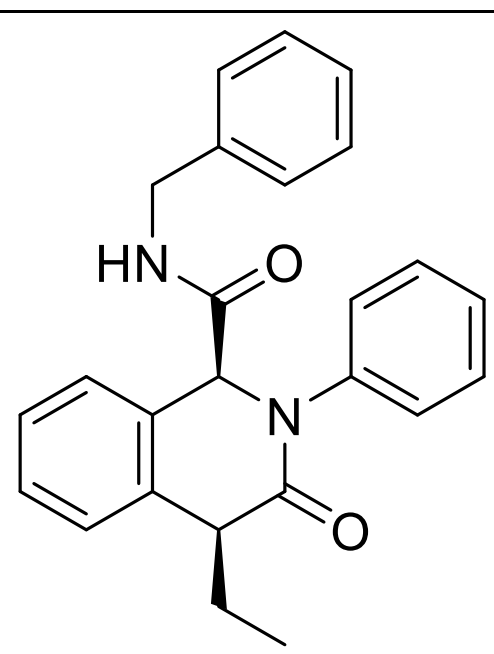<br>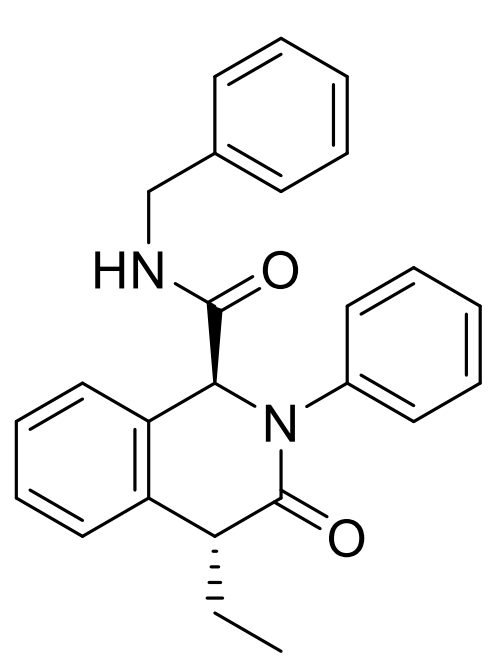	Conducted according to general procedure **B** using the mixture of *Z* and *E* isomers of **28l**. Purification by column chromatography on silica gel using hexane/ethyl acetate (20-40% ethyl acetate) as the eluent afforded the individual *cis* and *trans* diastereomers. Fractions containing both diastereomers were concentrated separately and re-subjected to column chromatography to obtain additional batches of pure *cis* and *trans* diastereomers.<br>**(±)-(1*S*,4*S*)-*N*-Benzyl-4-ethyl-3-oxo-2-phenyl-1,2,3,4-tetrahydroisoquinoline-1-carboxamide *cis*-29l**<br>Yield: 120 mg (62%, 0.5 mmol scale), white solid, melting point: 194-197 °C.<br>**^{1}H NMR** (500 MHz, $CDCl_3$) δ 7.38 – 7.30 (m, 4H), 7.29 – 7.22 (m, 7H), 7.20 – 7.14 (m, 1H), 7.04 – 6.97 (m, 2H), 6.47 (t, *J* = 5.8 Hz, 1H), 5.31 (s, 1H), 4.39 (dd, *J* = 14.8, 6.1 Hz, 1H), 4.26 (dd, *J* = 14.8, 5.4 Hz, 1H),

	3.46 (dd, *J* = 8.7, 6.7 Hz, 1H), 2.21 – 2.10 (m, 1H), 1.94 – 1.83 (m, 1H), 1.02 (t, *J* = 7.4 Hz, 3H).<br>**$^{13}C\{^1H\}$ NMR** (126 MHz, $CDCl_3$) δ 172.7, 169.3, 141.7, 137.7, 136.3, 129.6, 129.3, 128.5, 128.4, 128.0, 127.5, 127.3, 127.2, 126.8, 126.4, 68.4, 49.0, 43.4, 30.7, 12.7.<br>**HRMS** (ESI): m/z $[M+H]^+$ for $C_{25}H_{24}N_2O_2$, calcd 385.1911, found 385.1915.<br>**(±)-(1*S*,4*R*)-*N*-Benzyl-4-ethyl-3-oxo-2-phenyl-1,2,3,4-tetrahydroisoquinoline-1-carboxamide *trans*-29l**<br>Yield: 31 mg (16%, 0.5 mmol scale), white solid, melting point: 193-195 °C.<br>**^1H NMR** (500 MHz, $CDCl_3$) δ 7.43 – 7.21 (m, 12H), 7.05 – 6.96 (m, 2H), 6.34 (t, *J* = 5.9 Hz, 1H), 5.29 (s, 1H), 4.35 (dd, *J* = 14.9, 5.8 Hz, 1H), 4.29 (dd, *J* = 14.9, 5.6 Hz, 1H), 3.88 (t, *J* = 4.8 Hz, 1H), 2.31 (dqd, *J* = 14.5, 7.2, 4.7 Hz, 1H), 2.17 (dqd, *J* = 14.5, 7.3, 4.6 Hz, 1H), 0.93 (t, *J* = 7.3 Hz, 3H).<br>**$^{13}C\{^1H\}$ NMR** (126 MHz, $CDCl_3$) δ 171.8, 169.4, 142.1, 137.7, 135.2, 131.9, 129.4, 128.72, 128.70, 127.6, 127.5, 127.4, 127.0, 126.9, 126.4, 126.1, 68.6, 45.3, 43.8, 21.4, 10.3.<br>**HRMS** (ESI): m/z $[M+H]^+$ for $C_{25}H_{24}N_2O_2$, calcd 385.1911, found 385.1917.

Characterization of synthesized compounds 30, 31 and 33-37

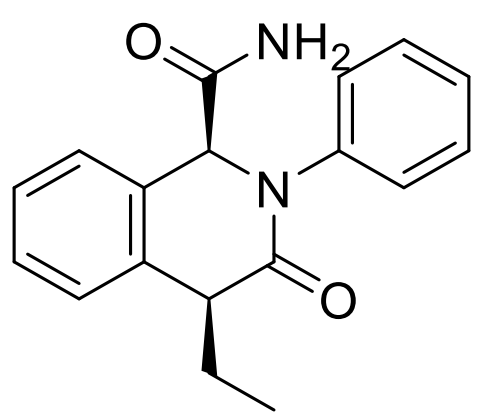	**(±)-(1*S*,4*S*)-4-Ethyl-3-oxo-2-phenyl-1,2,3,4-tetrahydroisoquinoline-1-carboxamide 30**<br>Yield: 150 mg (68%, 0.75 mmol scale), melting point: 279-281 °C.<br>**^{1}H NMR** (500 MHz, DMSO-*D*$_6$) δ 7.81 (s, 1H), 7.62 – 7.56 (m, 1H), 7.45 – 7.38 (m, 2H), 7.37 – 7.19 (m, 7H), 5.39 (s, 1H), 3.42 (t, *J* = 7.6 Hz, 1H), 2.23 (dp, *J* = 14.8, 7.5 Hz, 1H), 1.96 (dp, *J* = 15.1, 7.6 Hz, 1H), 1.04 (t, *J* = 7.5 Hz, 3H).<br>**^{13}C{^{1}H} NMR** (126 MHz, DMSO-*D*$_6$) δ 171.0, 170.9, 142.4, 136.4, 130.6, 129.0, 128.3, 127.7, 127.5, 127.1, 126.43, 126.35, 67.2, 48.1, 30.4, 12.8.<br>**HRMS** (ESI): m/z [M+H]$^+$ for $C_{18}H_{18}N_2O_2$, calcd 295.1441, found 295.1436.
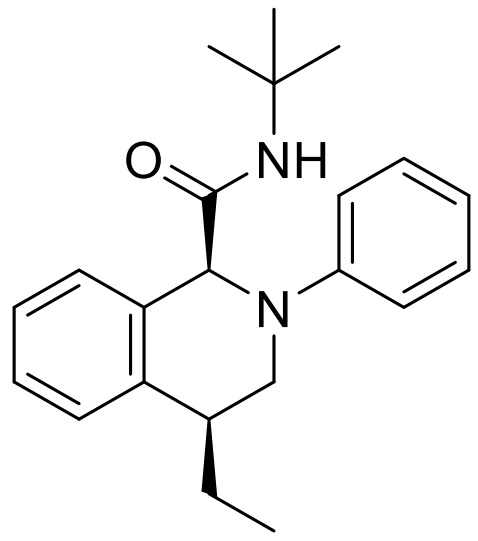	**(±)-(1*S*,4*S*)-*N*-(*tert*-Butyl)-4-ethyl-2-phenyl-1,2,3,4-tetrahydroisoquinoline-1-carboxamide 31**<br>Purified by trituration with diethyl ether. Yield: 929 mg (92%, 3 mmol scale), 132 mg (98%, 0.4 mmol scale), white solid, melting point: 102-105 °C.<br>**^{1}H NMR** (500 MHz, $CDCl_3$) δ 7.73 – 7.64 (m, 1H), 7.35 – 7.29 (m, 2H), 7.26 – 7.21 (m, 2H), 7.20 – 7.15 (m, 1H), 7.09 – 7.02 (m, 2H), 6.98 – 6.92 (m, 1H), 6.59 (s, 1H), 4.85 (s, 1H), 3.74 (dd, *J* = 12.2, 3.5 Hz, 1H), 3.35 (dd, *J* = 12.1, 3.8 Hz, 1H), 2.82 (tt, *J* = 7.1, 3.6 Hz, 1H), 1.92 – 1.79 (m, 2H), 1.15 (s, 9H), 1.12 (t, *J* = 7.4 Hz, 3H).<br>**^{13}C{^{1}H} NMR** (126 MHz, $CDCl_3$) δ 170.9, 150.4, 137.9, 132.1, 129.4, 128.4, 128.3, 127.1, 126.5, 120.8, 117.1, 65.9, 50.70, 50.68, 40.2, 28.41, 28.36, 12.4.<br>**HRMS** (ESI): m/z [M+Na]$^+$ for $C_{22}H_{28}N_2O$, calcd 359.2094, found 359.2070.

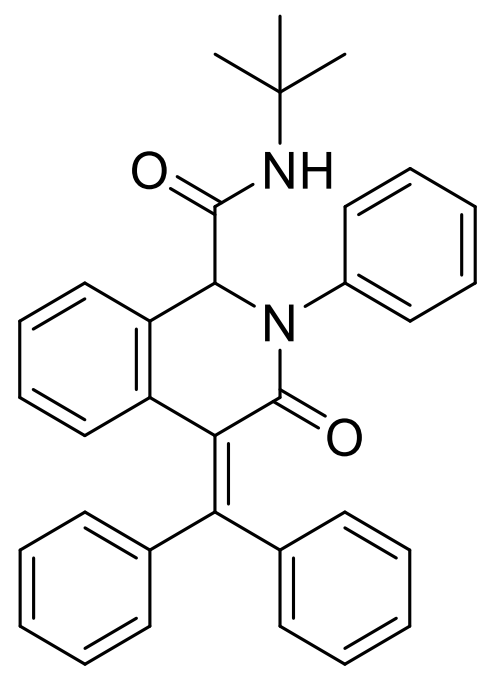	***N*-(*tert*-Butyl)-4-(diphenylmethylene)-3-oxo-2-phenyl-1,2,3,4-tetrahydroisoquinoline-1-carboxamide 33**<br>Purified by column chromatography on silica gel using hexane/ethyl acetate (15-35% ethyl acetate) as the eluent, followed by trituration with diethyl ether. Yield: 302 mg (69%, 0.9 mmol scale), white solid, melting point: 212-213 °C.<br>**^{1}H NMR** (500 MHz, $CDCl_3$) δ 7.49 – 7.44 (m, 2H), 7.41 – 7.37 (m, 1H), 7.36 – 7.29 (m, 4H), 7.28 – 7.24 (m, 3H), 7.23 – 7.19 (m, 2H), 7.18 – 7.13 (m, 3H), 7.08 – 7.03 (m, 2H), 7.03 – 6.97 (m, 1H), 6.92 – 6.87 (m, 1H), 6.10 (s, 1H), 5.12 (s, 1H), 1.42 (s, 9H).<br>**^{13}C{^{1}H} NMR** (126 MHz, $CDCl_3$) δ 168.3, 165.0, 150.0, 142.4, 141.8, 140.8, 133.8, 133.5, 130.2, 129.82, 129.79, 129.4, 128.7, 128.3, 127.9, 127.8, 127.7, 127.6, 127.5, 126.94, 126.91, 125.6, 69.6, 52.2, 28.9.<br>**HRMS** (ESI): m/z $[M+H]^+$ for $C_{33}H_{30}N_2O_2$, calcd 487.2380, found 487.2375.

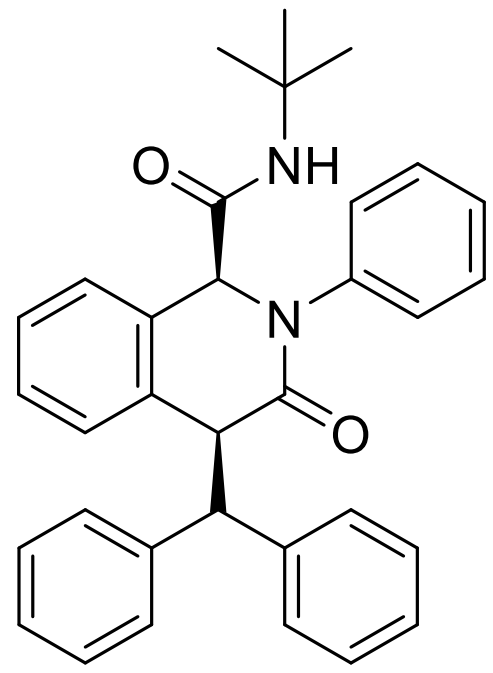

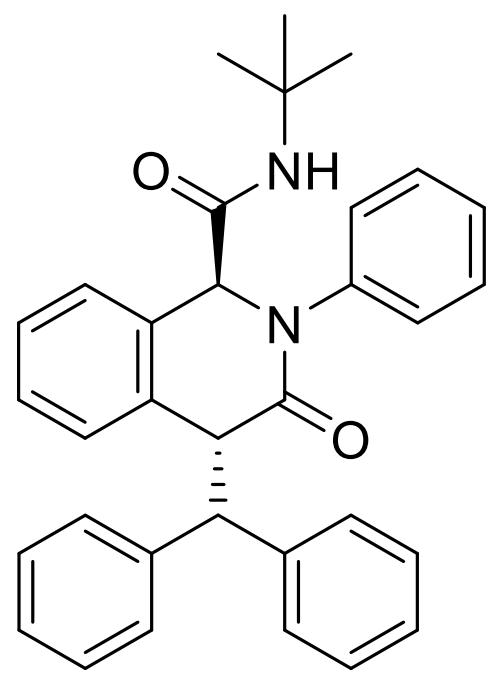

Purification by column chromatography on silica gel using hexane/ethyl acetate (5-15% ethyl acetate) as the eluent afforded the individual *cis* and *trans* diastereomers, which were further purified by trituration with pentane.

(±)-(1*S*,4*S*)-4-Benzhydryl-*N*-(*tert*-butyl)-3-oxo-2-phenyl-1,2,3,4-tetrahydroisoquinoline-1-carboxamide *cis*-34

Yield: 89 mg (61%, 0.3 mmol scale), white solid, melting point: 129-132 °C.

^{1}H NMR (500 MHz, $CDCl_3$) δ 7.73 – 7.64 (m, 2H), 7.43 – 7.31 (m, 5H), 7.27 – 7.22 (m, 3H), 7.17 – 7.05 (m, 7H), 7.00 – 6.93 (m, 1H), 6.45 – 6.37 (m, 1H), 5.82 (s, 1H), 5.38 (s, 1H), 4.53 (d, *J* = 11.9 Hz, 1H), 4.46 (d, *J* = 11.9 Hz, 1H), 1.37 (s, 9H).

^{13}C{^{1}H} NMR (126 MHz, $CDCl_3$) δ 170.5, 168.2, 142.6, 141.9, 140.6, 135.3, 130.9, 129.7, 129.6, 129.2, 128.8, 128.4, 128.3, 127.5, 127.4, 127.2, 127.1, 127.0, 126.8, 69.0, 56.9, 54.1, 52.2, 28.7.

(±)-(1*S*,4*R*)-4-Benzhydryl-*N*-(*tert*-butyl)-3-oxo-2-phenyl-1,2,3,4-tetrahydroisoquinoline-1-carboxamide *trans*-34

Yield: 38 mg (26%, 0.3 mmol scale), white solid, melting point: 226-228 °C.

^{1}H NMR (500 MHz, $CDCl_3$) δ 7.34 – 7.16 (m, 14H), 7.14 – 7.08 (m, 2H), 6.89 – 6.79 (m, 3H), 5.14 (s, 1H), 4.84 (d, *J* = 4.2 Hz, 1H), 4.74 (d, *J* = 4.2 Hz, 1H), 4.46 (s, 1H), 0.97 (s, 9H).

^{13}C{^{1}H} NMR ^{13}C NMR (126 MHz, $CDCl_3$) δ 170.4, 167.8, 140.7, 140.3, 139.5, 133.6, 131.4, 130.0, 129.1, 128.8, 128.5, 128.43, 128.35, 128.1, 127.6, 127.3, 127.2, 127.0, 126.9, 125.0, 68.1, 57.2, 52.3, 51.5, 28.0.

HRMS (ESI): m/z $[M+H]^+$ for $C_{33}H_{32}N_2O_2$, calcd 489.2537, found 489.2534.

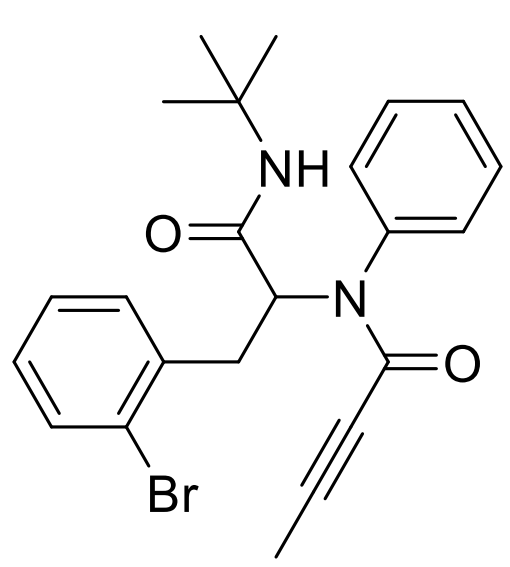

***N*-(3-(2-Bromophenyl)-1-(*tert*-butylamino)-1-oxopropan-2-yl)-*N*-phenylbut-2-ynamide 35**

Purified by column chromatography on silica gel using hexane/ethyl acetate (15-35% ethyl acetate) as the eluent, followed by trituration with diethyl ether. Yield: 338 mg (51%, 1.5 mmol scale), brownish oil.

^{1}H NMR (500 MHz, $CDCl_3$) δ 7.53 – 7.48 (m, 1H), 7.38 – 7.31 (m, 3H), 7.21 – 7.17 (m, 2H), 7.17 – 7.12 (m, 2H), 7.11 – 7.05 (m, 1H), 6.37 (s,

	1H), 5.19 (dd, *J* = 8.2, 7.0 Hz, 1H), 3.06 – 2.91 (m, 2H), 1.70 (s, 3H), 1.32 (s, 9H).<br>**$^{13}C\{^1H\}$ NMR** (126 MHz, $CDCl_3$) δ 168.6, 155.5, 138.8, 136.7, 132.9, 131.6, 129.6, 128.9, 128.7, 128.5, 127.5, 125.2, 92.1, 74.0, 59.1, 51.4, 34.9, 28.7, 4.0.<br>**HRMS** (ESI): m/z $[M+Na]^+$ for $C_{23}H_{25}BrN_2O_2$, calcd 463.0992, found 463.0988.
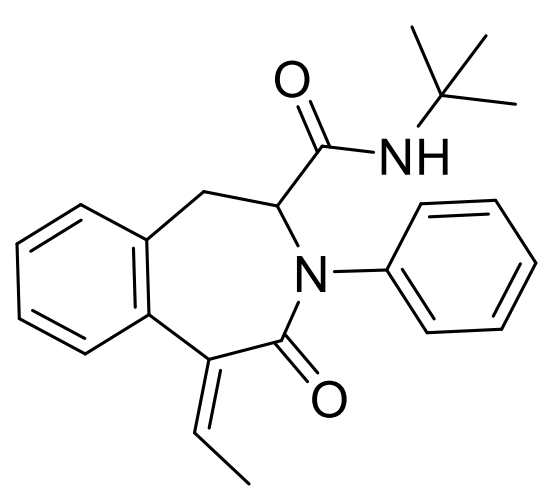	**(*Z*)-*N*-(*tert*-Butyl)-5-ethylidene-4-oxo-3-phenyl-2,3,4,5-tetrahydro-1*H*-benzo[*d*]azepine-2-carboxamide 36**<br>Purified by column chromatography on silica gel using hexane/ethyl acetate (30-40% ethyl acetate) as the eluent, followed by trituration with pentane. Yield: 110 mg (55%, 0.55 mmol scale), white solid, melting point: 113-115 °C.<br>**^1H NMR** (500 MHz, $CDCl_3$) δ 7.44 – 7.37 (m, 2H), 7.34 – 7.24 (m, 6H), 7.21 – 7.15 (m, 1H), 6.08 (q, *J* = 7.1 Hz, 1H), 5.47 (s, 1H), 4.54 (t, *J* = 4.8 Hz, 1H), 3.62 – 3.49 (m, 2H), 2.05 (d, *J* = 7.1 Hz, 3H), 1.15 (s, 9H).<br>**$^{13}C\{^1H\}$ NMR** (126 MHz, $CDCl_3$) δ 168.8, 167.9, 142.8, 138.6, 137.0, 136.2, 133.3, 129.5, 129.2, 128.2, 127.54, 127.46, 127.1, 65.1, 51.5, 34.7, 28.4, 16.8.<br>**HRMS** (ESI): m/z $[M+H]^+$ for $C_{23}H_{26}N_2O_2$, calcd 363.2067, found 363.2067.

Purification by column chromatography on silica gel using hexane/ethyl acetate (20-25% ethyl acetate) as the eluent afforded the individual *cis* and *trans* diastereomers, which were further purified by trituration with cold pentane.

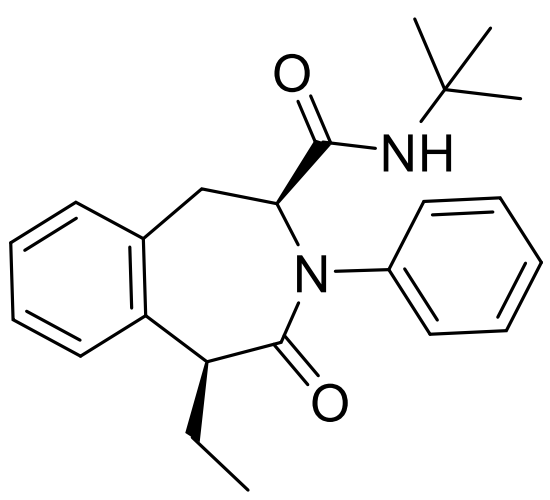

(±)-(2*S*,5*S*)-*N*-(*tert*-Butyl)-5-ethyl-4-oxo-3-phenyl-2,3,4,5-tetrahydro-1*H*-benzo[*d*]azepine-2-carboxamide *cis*-37

Yield: 56 mg (44%, 0.35 mmol scale), white solid, melting point: 152-153 °C.

^{1}H NMR (500 MHz, $CDCl_3$) δ 7.35 – 7.28 (m, 4H), 7.28 – 7.23 (m, 1H), 7.23 – 7.18 (m, 2H), 7.11 – 7.05 (m, 2H), 4.83 (s, 1H), 4.65 (t, *J* = 5.2 Hz, 1H), 4.10 (t, *J* = 7.2 Hz, 1H), 3.90 (dd, *J* = 15.4, 5.6 Hz, 1H), 3.43 (dd, *J* = 15.3, 4.9 Hz, 1H), 2.51 – 2.38 (m, 1H), 2.06 (s, 1H), 1.05 (t, *J* = 7.3 Hz, 3H), 0.95 (s, 9H).

^{13}C{^{1}H} NMR (126 MHz, $CDCl_3$) δ 172.1, 168.3, 142.7, 137.6, 135.7, 130.2, 129.0, 127.9, 127.8, 127.3, 127.2, 126.0, 67.8, 51.2, 50.2, 35.4, 28.2, 21.7, 13.0.

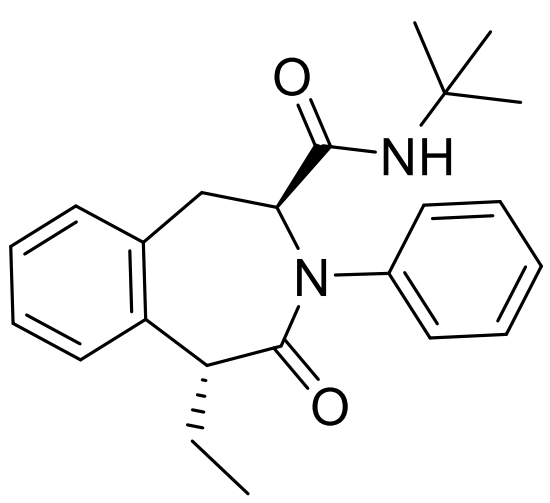

(±)-(2*S*,5*R*)-*N*-(*tert*-Butyl)-5-ethyl-4-oxo-3-phenyl-2,3,4,5-tetrahydro-1*H*-benzo[*d*]azepine-2-carboxamide *trans*-37

Yield: 40 mg (31%, 0.35 mmol scale), white solid, melting point: 190-192 °C.

^{1}H NMR ^{1}H NMR (500 MHz, $CDCl_3$) δ 7.32 – 7.16 (m, 7H), 6.95 – 6.87 (m, 2H), 5.81 (s, 1H), 4.29 (dd, *J* = 9.1, 7.8 Hz, 1H), 3.90 (dd, *J* = 8.2, 6.0 Hz, 1H), 3.80 (dd, *J* = 15.4, 7.9 Hz, 1H), 3.35 (dd, *J* = 15.3, 9.0 Hz, 1H), 2.46 – 2.34 (m, 1H), 2.00 – 1.90 (m, 1H), 1.32 (s, 9H), 1.07 (t, *J* = 7.3 Hz, 3H).

^{13}C{^{1}H} NMR (126 MHz, $CDCl_3$) δ 173.3, 170.5, 144.5, 138.5, 135.5, 129.4, 129.3, 127.7, 127.3, 127.2, 126.8, 125.3, 66.1, 51.8, 49.6, 35.0, 28.7, 21.0, 12.9.

HRMS (ESI): m/z $[M+H]^+$ for $C_{23}H_{28}N_2O_2$, calcd 365.2224, found 365.2228.

X-ray diffraction determination

The single crystals of (*Z*)-**28i**, (*E*)-**28b**, (*E*)-**28e**, *cis*-**29a**, *trans*-**34**, *cis*-**37**, and *trans*-**37** were immersed in a film of NVH mounted on a polyimide microloop (MiTeGen), and transferred to a stream of cold nitrogen (Cryostream 500/700 cooling system by Oxford Cryosystems), and measured at a temperature of 150–293 K. The X-ray diffraction data were collected on a Rigaku XtaLAB Synergy R diffractometer with a HyPix-Arc 100 detector using mirror-monochromated Cu Kα (λ = 1.54184 Å) radiation (INCOATEC microfocus sealed tube) or a four-circle diffractometer Xcalibur, Atlas, Gemini with a graphite monochromator and fine-focus sealed Mo Kα (λ = 0.71073 Å) X-ray tube. The frames were integrated with the CrysAlisPro software package using a narrow-frame algorithm. The CrysAlisPro program package was used for cell refinements and data reductions. The structure was solved using the intrinsic phasing method,[1, 2] refined and visualized with the OLEX2-1.5 program.[3] A semiempirical/empirical absorption correction using spherical harmonics, implemented in the SCALE3 ABSPACK scaling algorithm, and an absorption correction (SADABS) were applied to all data. All non-hydrogen atoms were refined anisotropically. Hydrogen atoms were included in structure factor calculations. All Hydrogen atoms were assigned to idealized geometric positions. The crystallographic details are summarized in Table S4 and S5. CCDC 2574832-2574837 contains supplementary crystallographic data for this paper.

References

1 Sheldrick, G. M. *SHELXT*– Integrated Space-Group and Crystal-Structure Determination. *Acta Crystallogr. Sect. Found. Adv.* **2015**, *71* (1), 3–8. https://doi.org/10.1107/s2053273314026370.

2 Sheldrick, G. M. Crystal Structure Refinement with*SHELXL*. *Acta Crystallogr. Sect. C Struct. Chem.* **2015**, *71* (1), 3–8. https://doi.org/10.1107/s2053229614024218.

3 Dolomanov, O. V.; Bourhis, L. J.; Gildea, R. J.; Howard, J. A. K.; Puschmann, H. *OLEX2*: A Complete Structure Solution, Refinement and Analysis Program. *J. Appl. Crystallogr.* **2009**, *42* (2), 339–341. https://doi.org/10.1107/s0021889808042726.

Table S4. Crystal data and structure refinement for (*Z*)-**28i**, (*E*)-**28b**, (*E*)-**28e** and *cis*-**29a**

Identification code	(*Z*)-**28i**	(*E*)-**28b**	(*E*)-**28e**	*cis*-**29a**
CCDC number	2574832	2574833	2574834	2574835
Empirical formula	$C_{33}H_{38}N_2O_5$	$C_{22}H_{23}FN_2O_2$	$C_{28}H_{28}N_2O_3$	$C_{22}H_{26}N_2O_2$
Formula weight	542.65	366.42	440.52	350.45
Temperature [K]	150.00(10)	223.00(10)	223.00(10)	293(10)
Crystal system		orthorhombic		monoclinic
Space group	$P2_12_12_1$	Pbca	Pbca	$P2_1/n$
a [Å]<br>b [Å]<br>c [Å]<br>α [°]<br>β [°]<br>γ [°]	12.26850(10)<br>13.83410(10)<br>17.50960(10)<br>90<br>90<br>90	15.1829(12)<br>13.0884(9)<br>20.1730(15)<br>90<br>90<br>90	11.8477(6)<br>13.9501(8)<br>28.9718(18)<br>90<br>90<br>90	9.5013(14)<br>13.0160(19)<br>16.763(3)<br>90<br>105.100(15)<br>90
Volume [Å^3]	2971.79(4)	4008.8(5)	4788.4(5)	2001.4(5)
Z	4	8	8	4
ρ_{calc} [g/cm^3]	1.213	1.214	1.222	1.163
μ [mm^{-1}]	0.654	0.084	0.080	0.075
F(000)	1160.0	1552.0	1872.0	752.0
Crystal size [mm^3]	0.091 × 0.08 × 0.055	0.3 × 0.2 × 0.15	0.45 × 0.4 × 0.2	0.4 × 0.3 × 0.08
Radiation type	Cu Kα (λ = 1.54184)	Mo Kα (λ = 0.71073)	Mo Kα (λ = 0.71073)	Mo Kα (λ = 0.71073)
2Θ range for data collection [°]	8.146 to 159.878	4.038 to 60.858	4.442 to 60.956	5.928 to 52.742
Index ranges	-15 ≤ h ≤ 15, -17 ≤ k ≤ 17, -22 ≤ l ≤ 22	-20 ≤ h ≤ 19, -17 ≤ k ≤ 17, -26 ≤ l ≤ 21	-16 ≤ h ≤ 16, -19 ≤ k ≤ 19, -38 ≤ l ≤ 37	-11 ≤ h ≤ 10, -16 ≤ k ≤ 16, -20 ≤ l ≤ 20
Reflections collected	130043	16555	19528	12599
Independent reflections	6399 [R_{int} = 0.0485, R_{sigma} = 0.0147]	5294 [R_{int} = 0.0581, R_{sigma} = 0.0712]	6376 [R_{int} = 0.0472, R_{sigma} = 0.0534]	4082 [R_{int} = 0.0935, R_{sigma} = 0.1074]
Data/restraints/parameters	6399/122/399	5294/135/279	6376/0/302	4082/15/260
Goodness-of-fit on F^2 [(a)]	1.186	1.016	1.018	0.979
Final R indexes [I>=2σ (I)] [(b)]	R_1 = 0.0395, wR_2 = 0.1091	R_1 = 0.0671, wR_2 = 0.1460	R_1 = 0.0599, wR_2 = 0.1315	R_1 = 0.0685, wR_2 = 0.1711
Final R indexes [all data] [(b)]	R_1 = 0.0405, wR_2 = 0.1102	R_1 = 0.1559, wR_2 = 0.1897	R_1 = 0.1216, wR_2 = 0.1655	R_1 = 0.1589, wR_2 = 0.2257
Largest diff. peak/hole [e/Å^{-3}]	0.44/-0.33	0.28/-0.20	0.17/-0.18	0.18/-0.18
Flack parameter	0.04(4)	–	–	–

[(a)] GooF = S = $[[\sum w(F_o^2-F_c^2)^2]/(m-n)]^{1/2}$, where m = number of reflexes and n = number of parameters; [(b)] $R_1 = \sum||F_o|-|F_c||/\sum|F_o|$; $wR_2 = [\sum[w(F_o^2-F_c^2)^2/\sum[(wF_o^2)^2]]^{1/2}$; $w = 1/[\sigma^2(F_o^2)+(aP)^2+bP]$, where $P = (F_o^2+2F_c^2)/3$

Table S5. Crystal data and structure refinement for *trans*-**34**, *cis*-**37**, and *trans*-**37**.

Identification code	*trans*-**34**	*cis*-**37**	*trans*-**37**
CCDC number	2574836	– [c]	2574837
Empirical formula	$C_{66}H_{64}N_4O_4$	$C_{138}H_{166}N_{12}O_{12}$	$C_{23}H_{28}N_2O_2$
Formula weight	977.21	2184.82	364.47
Temperature [K]	150.00(10)	150.00(10)	150.00(10)
Crystal system	triclinic	monoclinic	monoclinic
Space group	P-1	Ia	$P2_1/n$
a [Å]<br>b [Å]<br>c [Å]<br>α [°]<br>β [°]<br>γ [°]	9.47930(6)<br>12.13238(8)<br>24.86824(11)<br>99.9145(5)<br>95.1526(5)<br>108.9256(6)	29.7369(8)<br>17.8568(3)<br>27.6589(8)<br>90<br>115.378(3)<br>90	10.08336(12)<br>19.4656(2)<br>10.27415(11)<br>90<br>100.5717(11)<br>90
Volume [$Å^3$]	2632.23(3)	13269.7(6)	1982.37(4)
Z	2	4	4
ρ_{calc} [g/cm^3]	1.233	1.094	1.221
μ [mm^{-1}]	0.599	0.550	0.613
F(000)	1040.0	4696.0	784.0
Crystal size [mm^3]	0.098 × 0.045 × 0.033	0.202 × 0.099 × 0.045	0.98 × 0.77 × 0.12
Radiation type	Cu Kα (λ = 1.54184)	Cu Kα (λ = 1.54184)	Cu Kα (λ = 1.54184)
2Θ range for data collection [°]	7.308 to 159.646	5.942 to 160.112	9.086 to 160.074
Index ranges	-11 ≤ h ≤ 11, -15 ≤ k ≤ 14, -31 ≤ l ≤ 31	-37 ≤ h ≤ 37, -22 ≤ k ≤ 19, -35 ≤ l ≤ 34	-12 ≤ h ≤ 12, -24 ≤ k ≤ 24, -12 ≤ l ≤ 13
Reflections collected	107104	55539	29428
Independent reflections	11081 [R_{int} = 0.0516, R_{sigma} = 0.0227]	22699 [R_{int} = 0.0981, R_{sigma} = 0.0753]	4276 [R_{int} = 0.0447, R_{sigma} = 0.0263]
Data/restraints/parameters	11081/0/674	22699/2/1484	4276/0/254
Goodness-of-fit on F^2 [b]	1.065	2.288	1.064
Final R indexes [I>=2σ (I)] [c]	R_1 = 0.0363, wR_2 = 0.0941	R_1 = 0.2096, wR_2 = 0.5116	R_1 = 0.0372, wR_2 = 0.0988
Final R indexes [all data] [c]	R_1 = 0.0387, wR_2 = 0.0961	R_1 = 0.2313, wR_2 = 0.5323	R_1 = 0.0407, wR_2 = 0.1020
Largest diff. peak/hole [e/$Å^{-3}$]	0.26/-0.18	1.39/-0.89	0.28/-0.20

[a] GooF = S = $[[\sum w(F_o^2-F_c^2)^2]/(m-n)]^{1/2}$, where m = number of reflexes and n = number of parameters; [b] $R_1 = \sum||F_o|-|F_c||/\sum|F_o|$; $wR_2 = [\sum[w(F_o^2-F_c^2)^2/\sum[(wF_o^2)^2]]^{1/2}$; $w = 1/[\sigma^2(F_o^2)+(aP)^2+bP]$, where $P = (F_o^2+2F_c^2)/3$; [c] crystallizes only as twinned crystal of poor quality, hence, obtained data cannot be stored in CCDC database; only atom connectivity and the relative configuration of the stereocenters can be reliably extracted from the data.

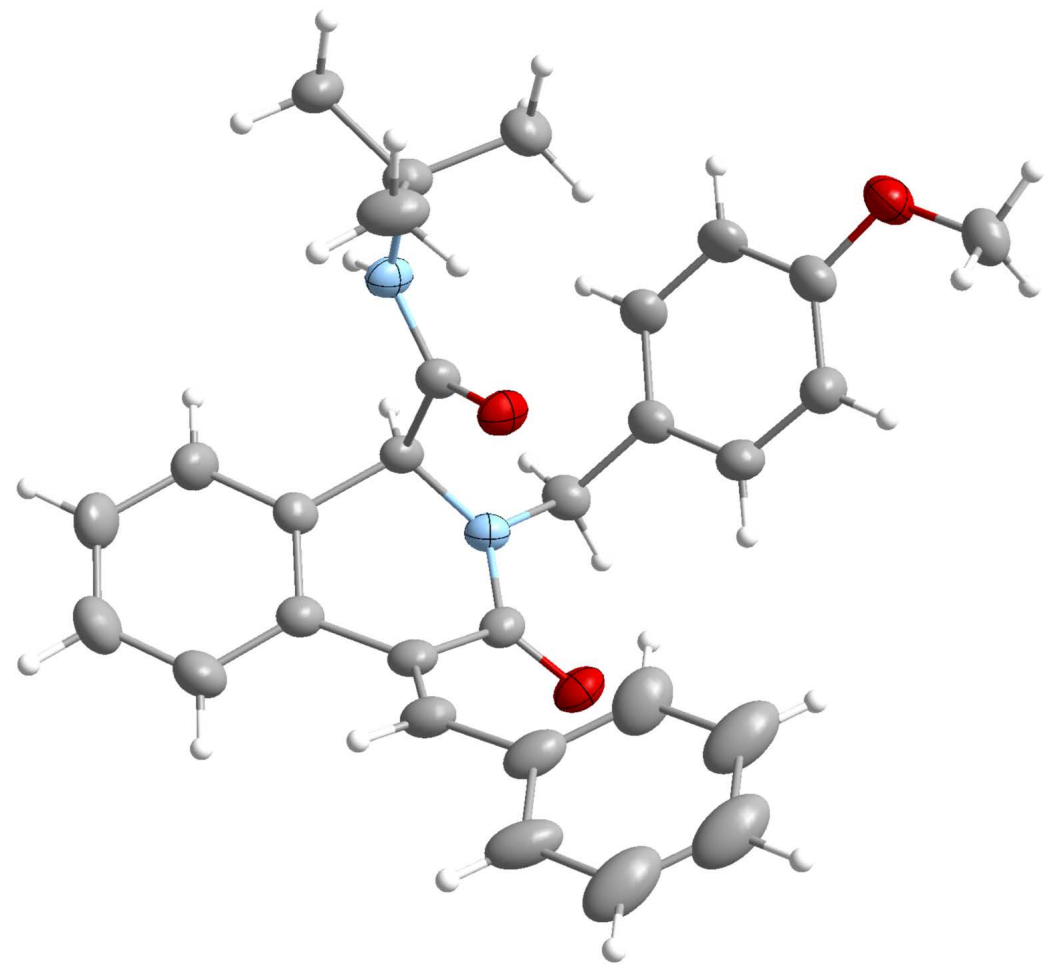

Figure S1. Molecular view of (*Z*)-**28i** (thermal ellipsoids are shown at the 50% probability level, color code: grey – carbon, blue – nitrogen, red – oxygen, white – hydrogen).

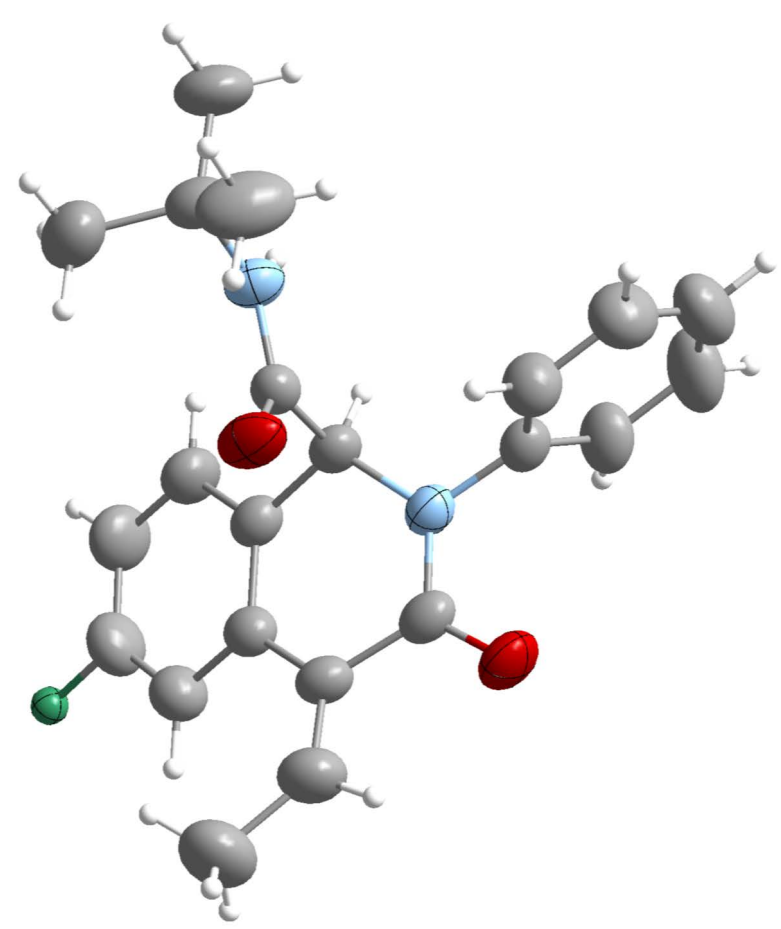

Figure S2. Molecular view of (*E*)-**28b** (thermal ellipsoids are shown at the 50% probability level, color code: grey – carbon, blue – nitrogen, red – oxygen, green – fluorine, white – hydrogen).

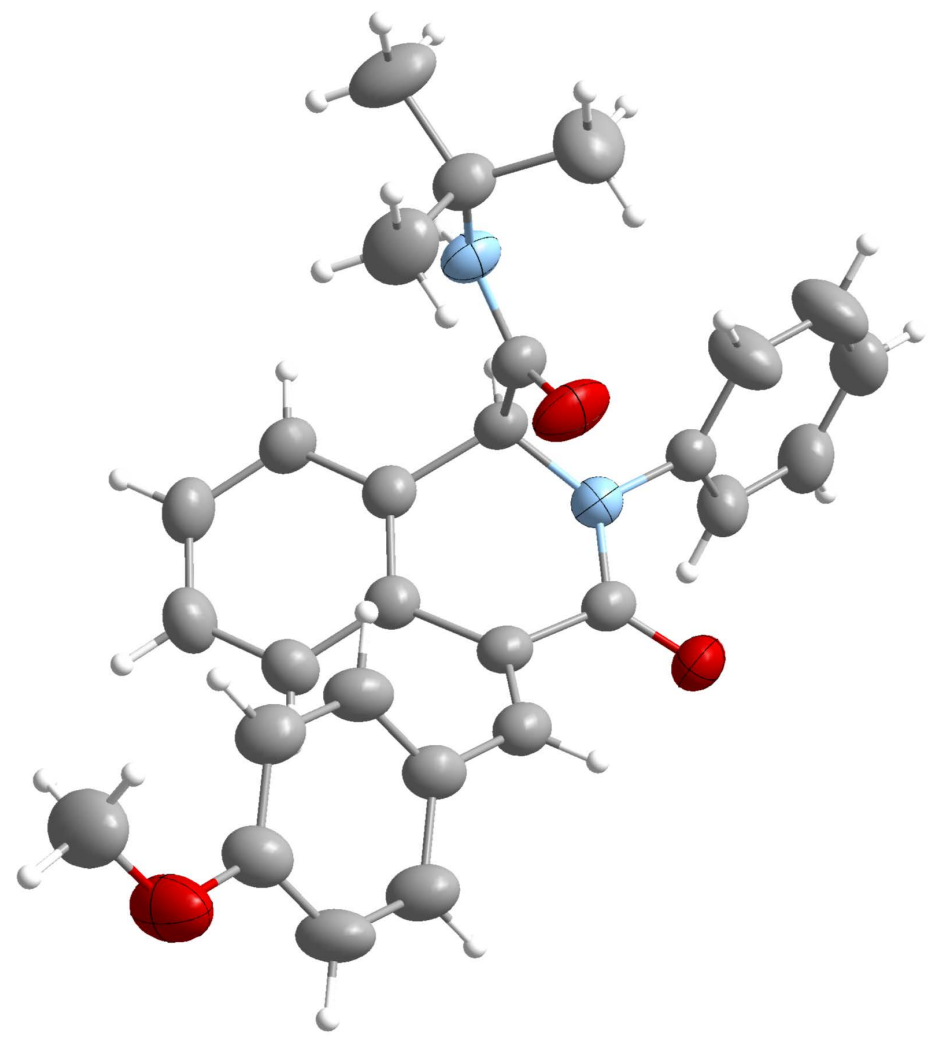

Figure S3. Molecular view of (*E*)-**28e** (thermal ellipsoids are shown at the 30% probability level, color code: grey – carbon, blue – nitrogen, red – oxygen, white – hydrogen).

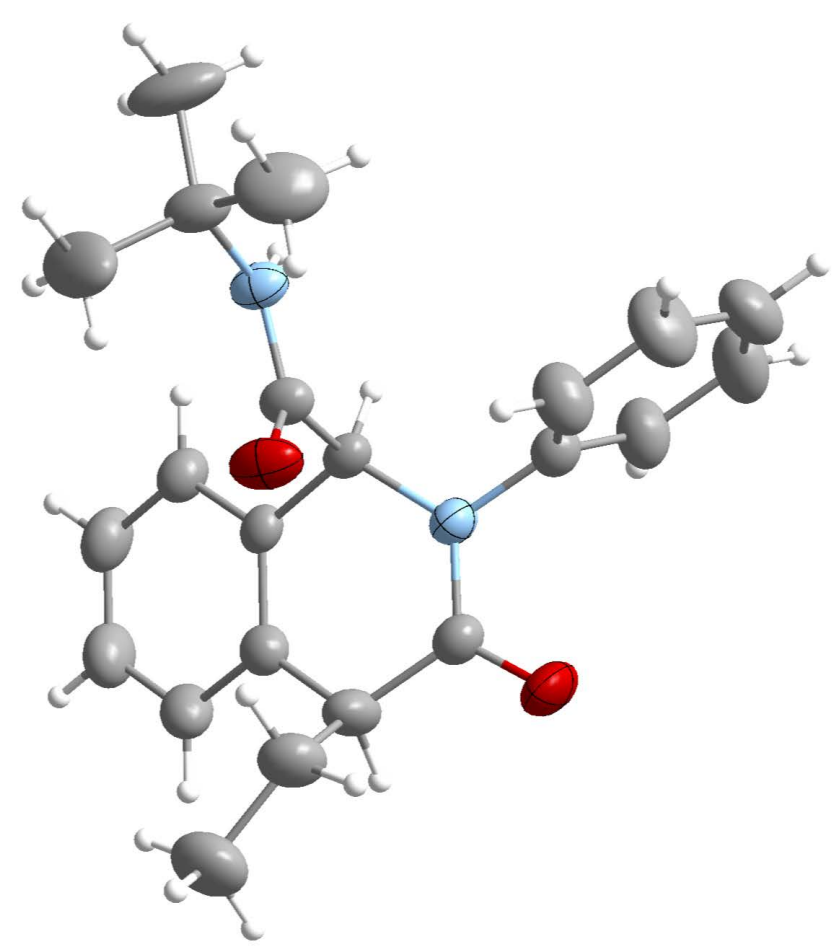

Figure S4. Molecular view of *cis*-**29a** (thermal ellipsoids are shown at the 30% probability level, color code: grey – carbon, blue – nitrogen, red – oxygen, white – hydrogen).

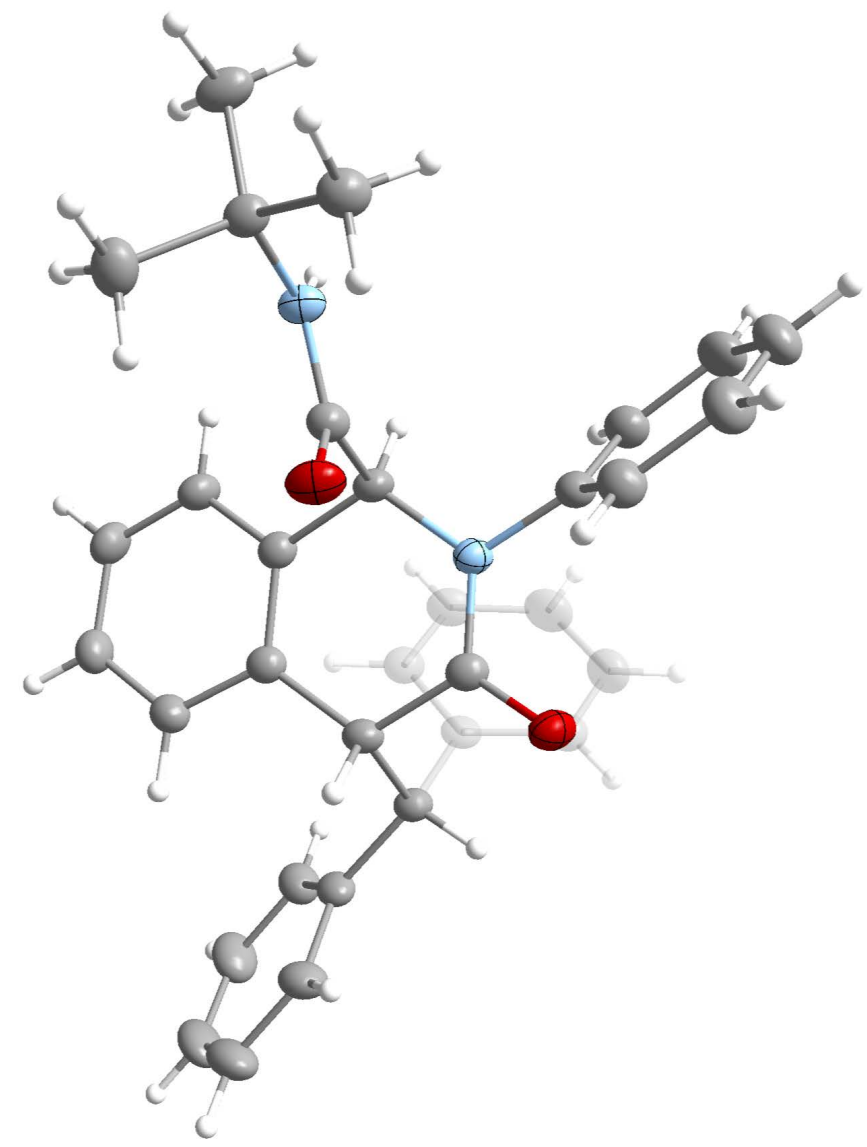

Figure S5. Molecular view of *trans*-**34** (thermal ellipsoids are shown at the 50% probability level, color code: grey – carbon, blue – nitrogen, red – oxygen, white – hydrogen).

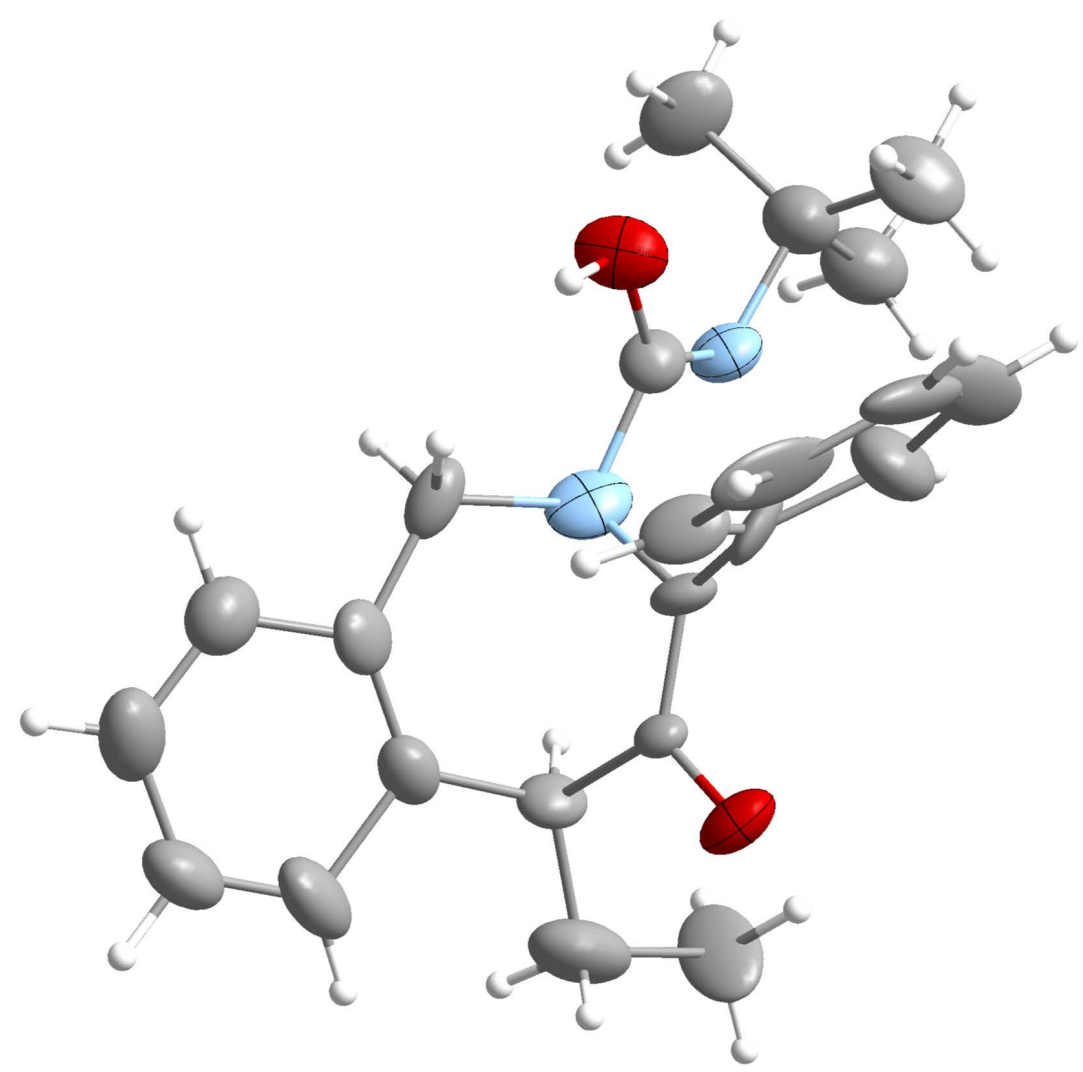

Figure S6. Molecular view of *cis*-**37** (thermal ellipsoids are shown at the 50% probability level, color code: grey – carbon, purple – bromine, blue – nitrogen, red – oxygen, white – hydrogen).

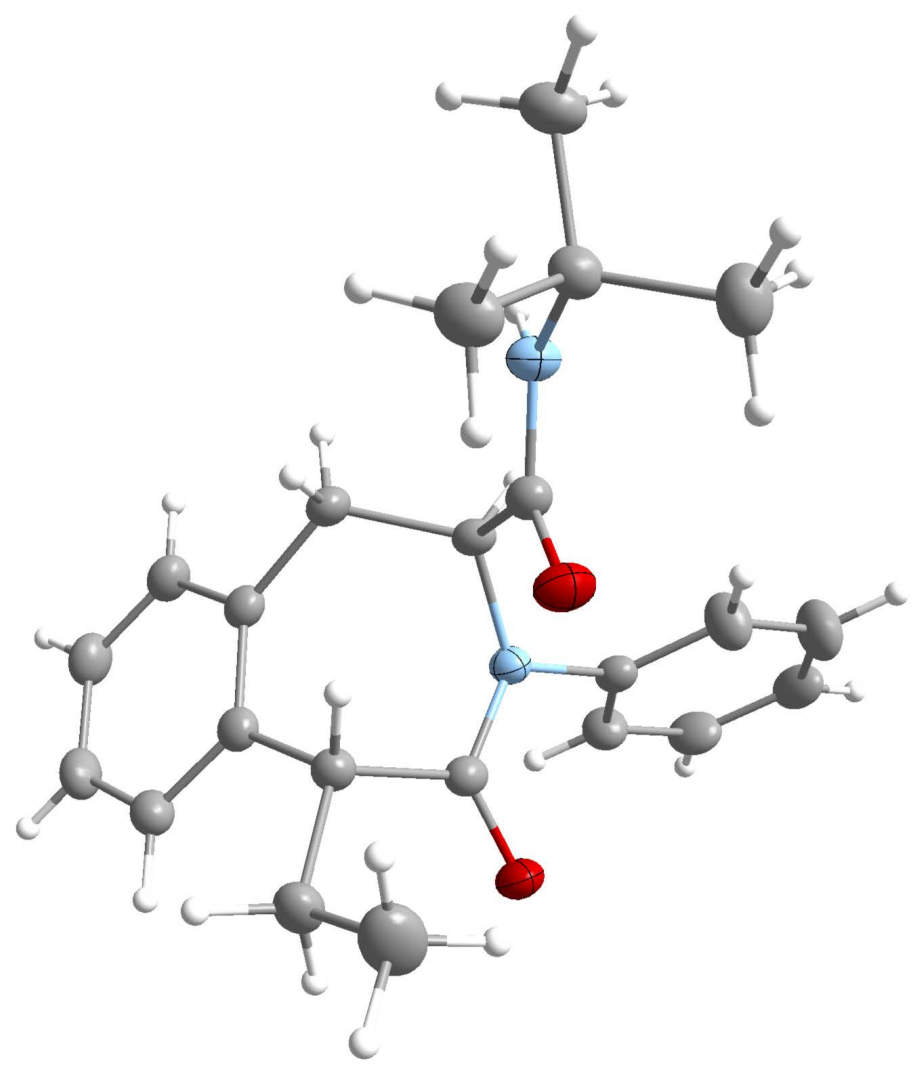

Figure S7. Molecular view of *trans*-**37** (thermal ellipsoids are shown at the 50% probability level, color code: grey – carbon, purple – bromine, blue – nitrogen, red – oxygen, white – hydrogen).

Copies of ^{1}H and $^{13}C\{^{1}H\}$ spectra

Copies of ^{1}H (500 MHz) and $^{13}C\{^{1}H\}$ (101 MHz) spectra of compound **27a**

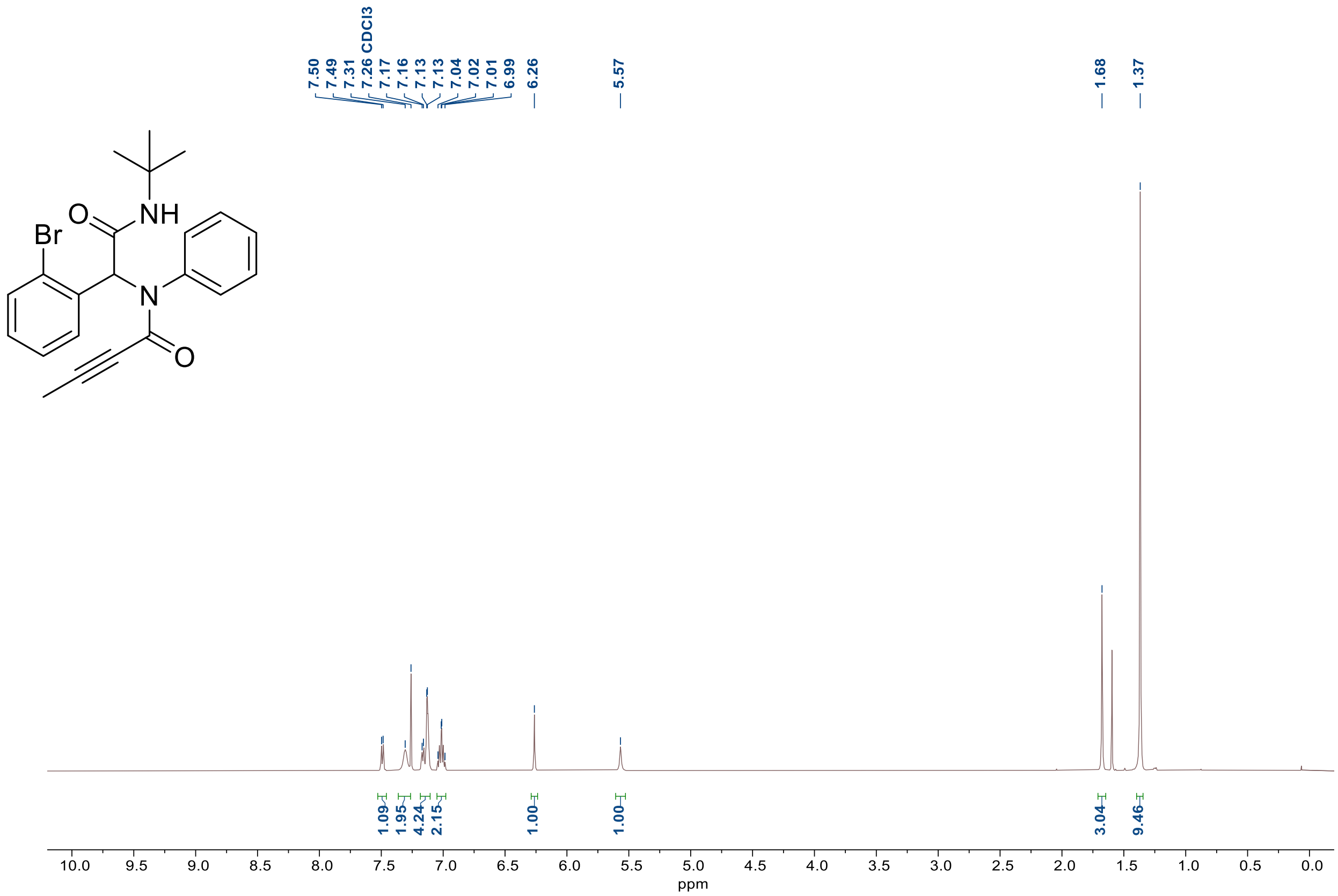

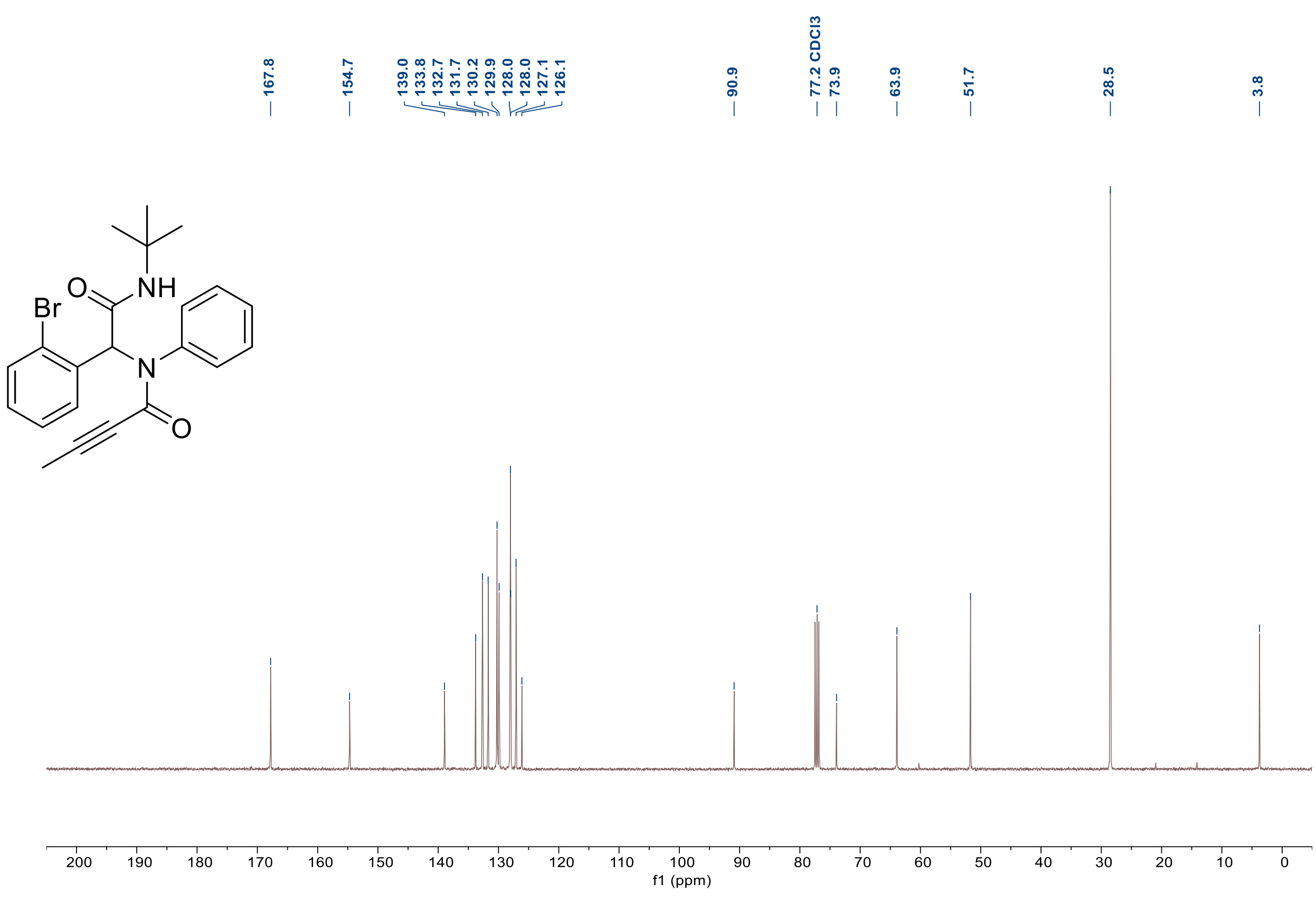

Copies of ^{1}H (500 MHz) and $^{13}C\{^{1}H\}$ (126 MHz) spectra of compound **27b**

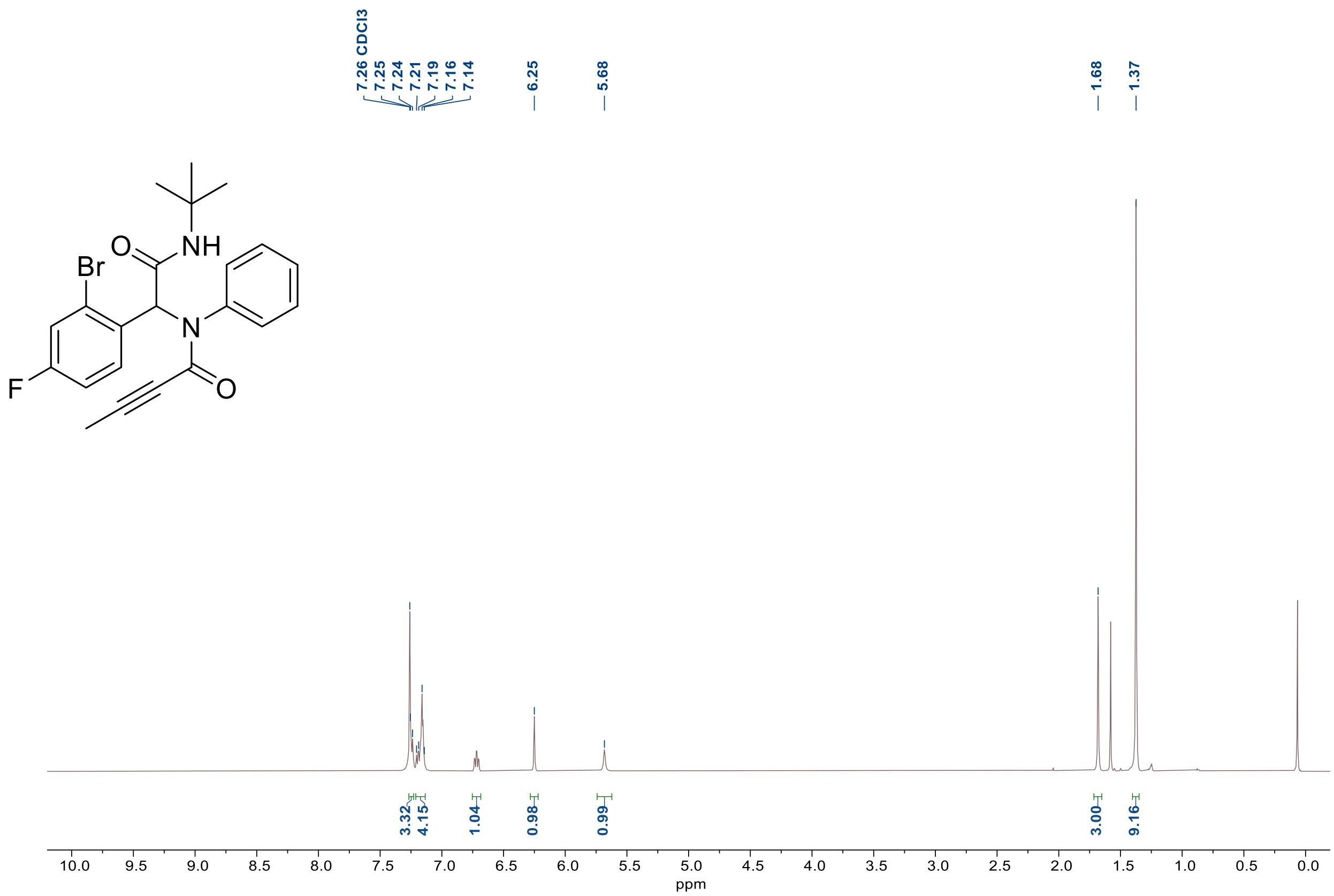

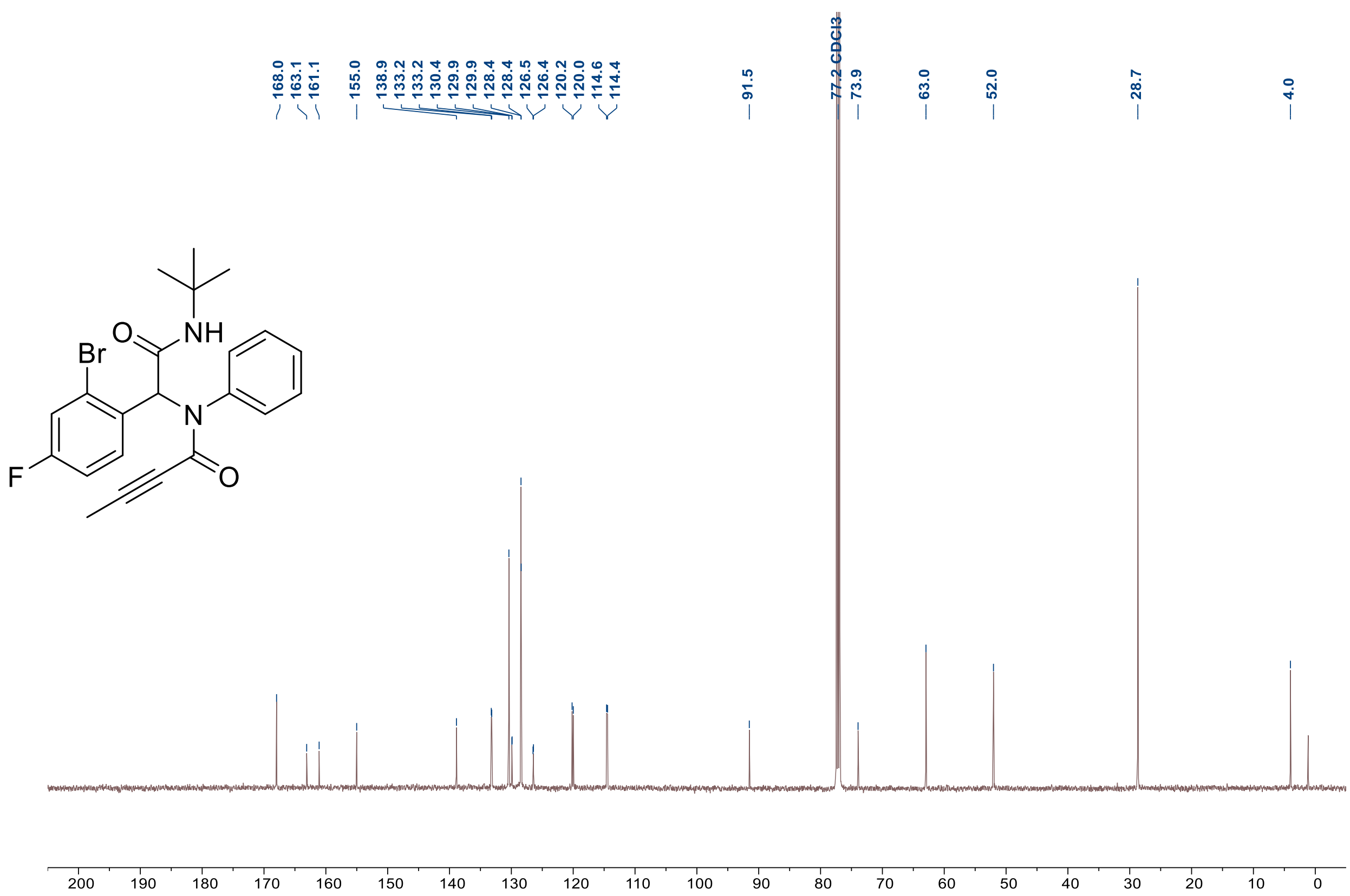

Copies of ^{1}H (400 MHz) and $^{13}C\{^{1}H\}$ (151 MHz) spectra of compound **27c**

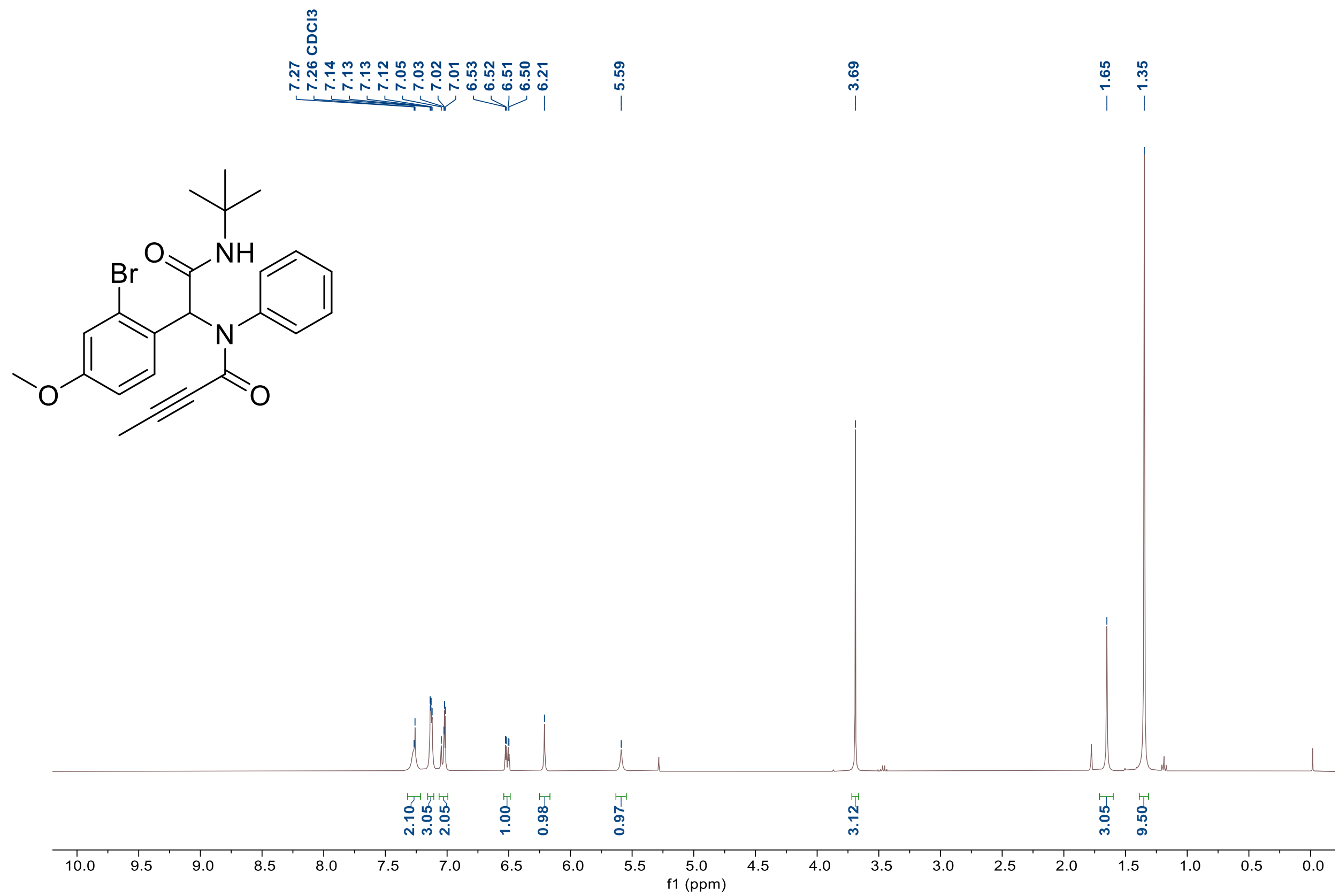

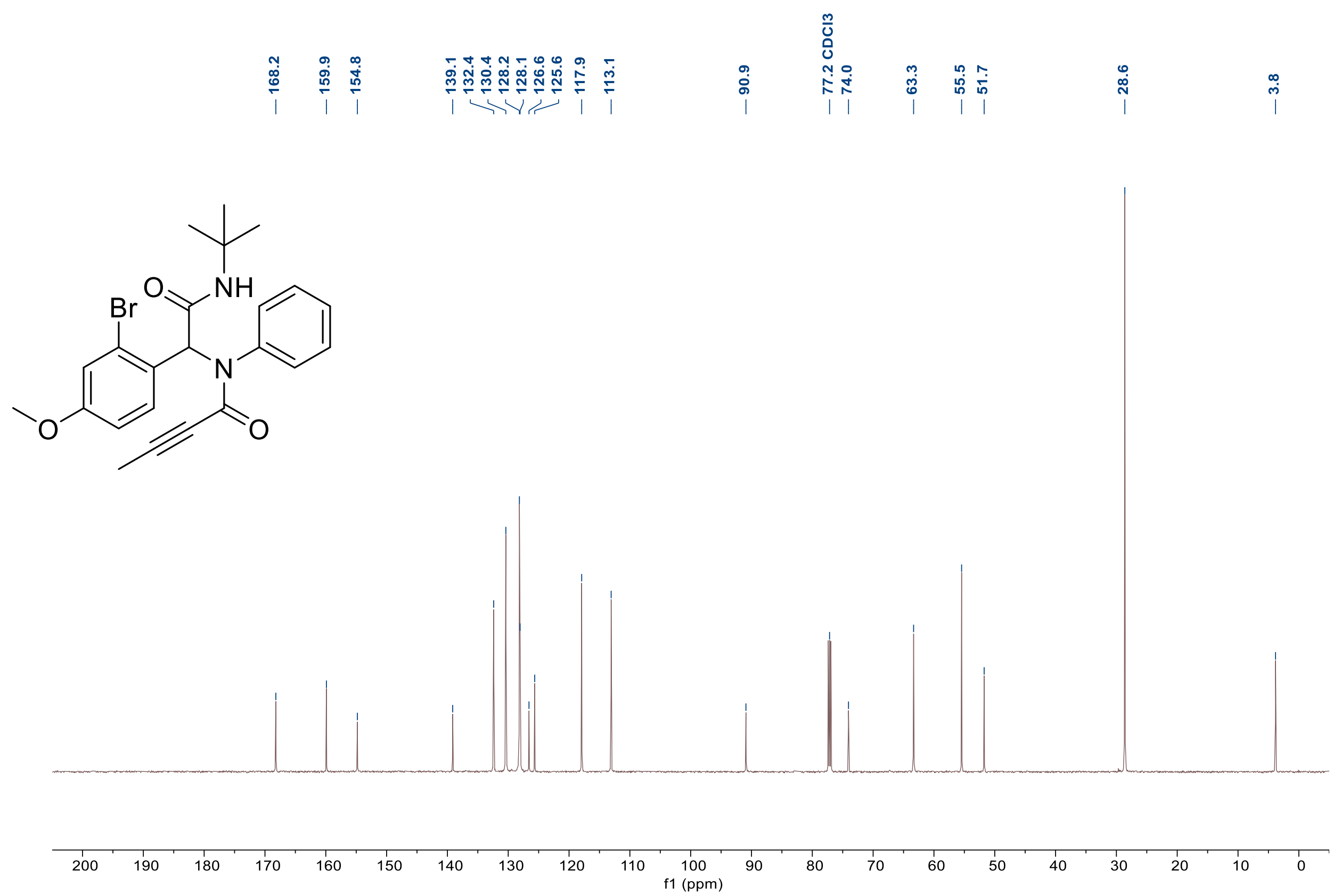

Copies of ^{1}H (500 MHz) and $^{13}C\{^{1}H\}$ (126 MHz) spectra of compound **27d**

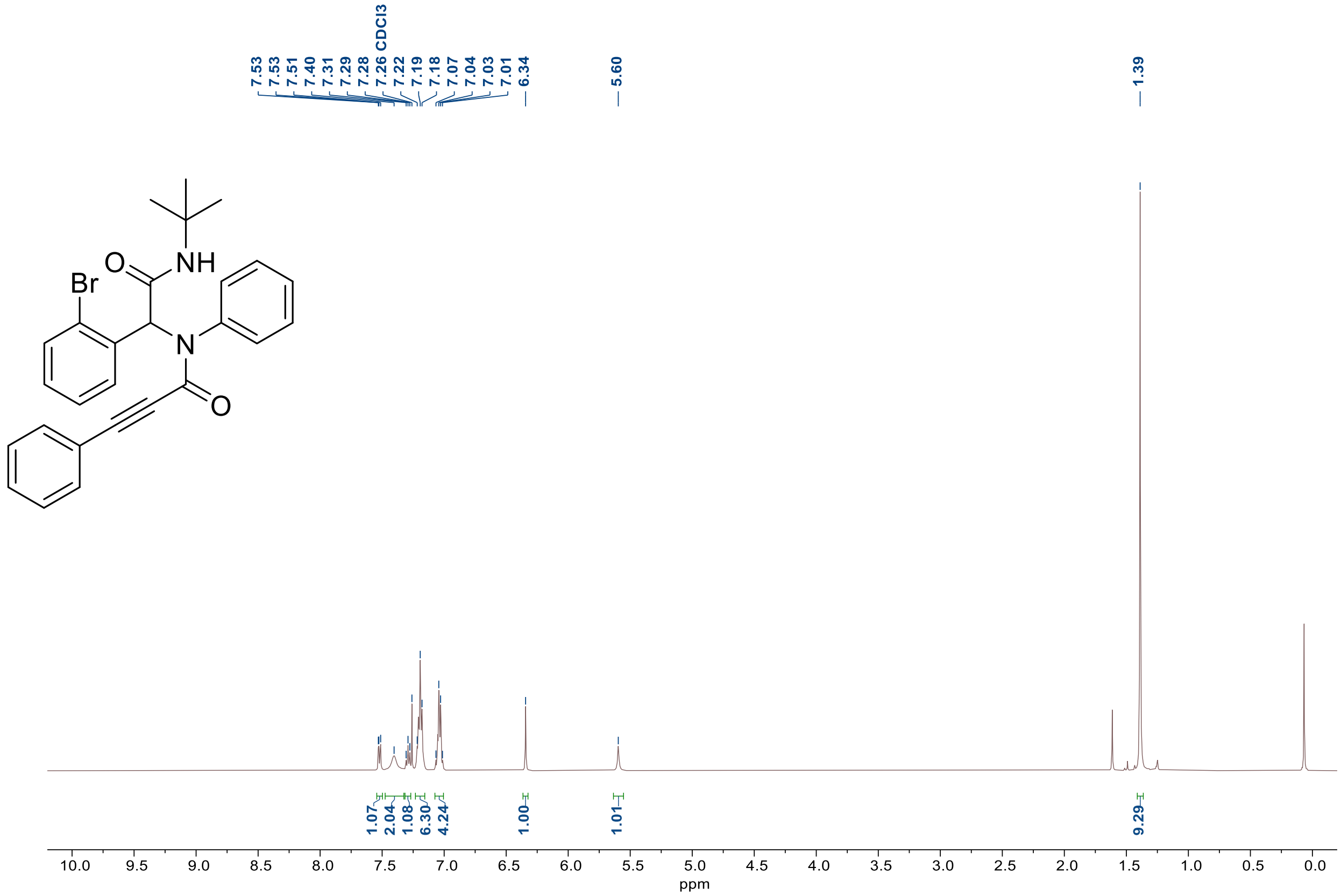

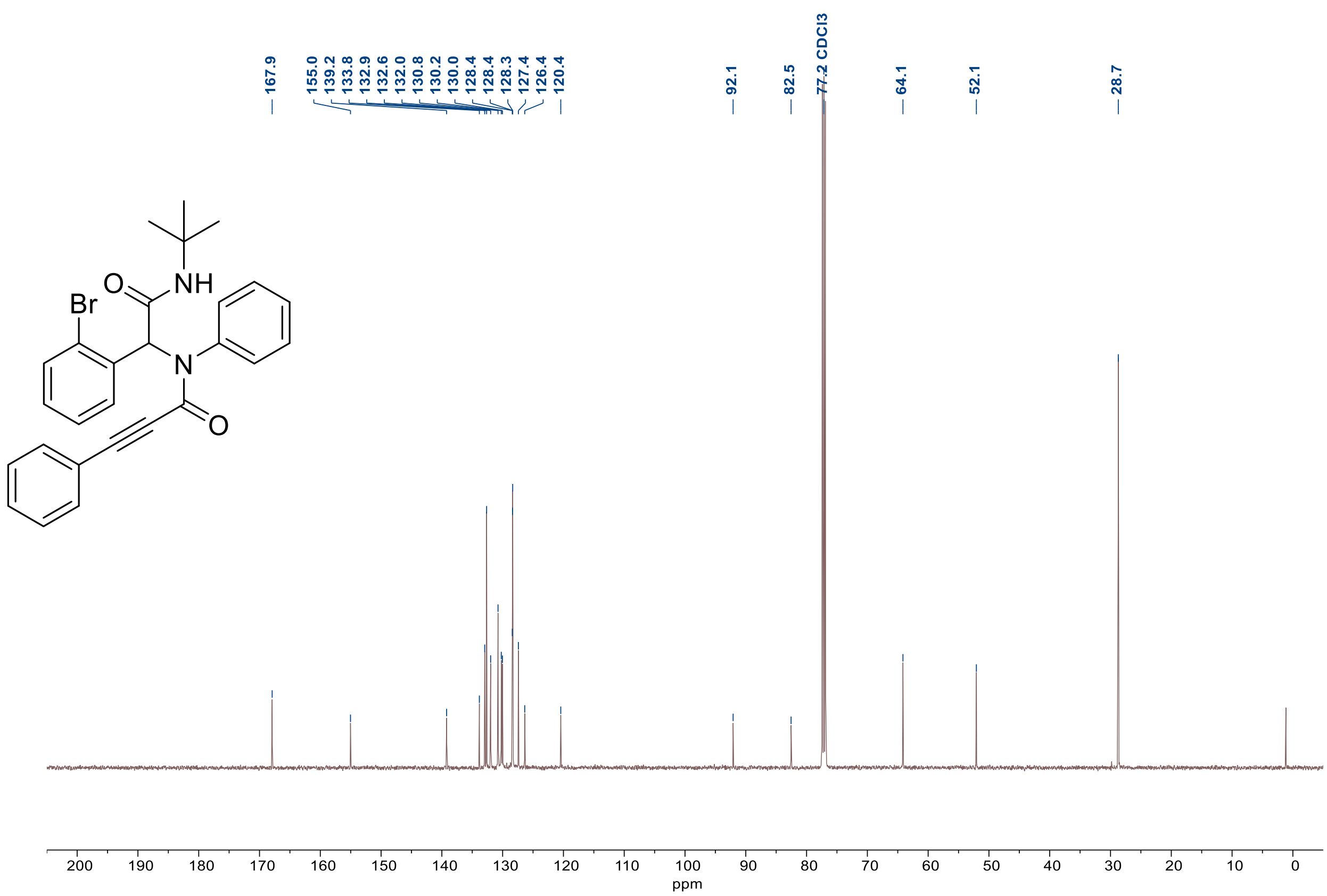

Copies of ^{1}H (500 MHz) and $^{13}C\{^{1}H\}$ (101 MHz) spectra of compound **27e**

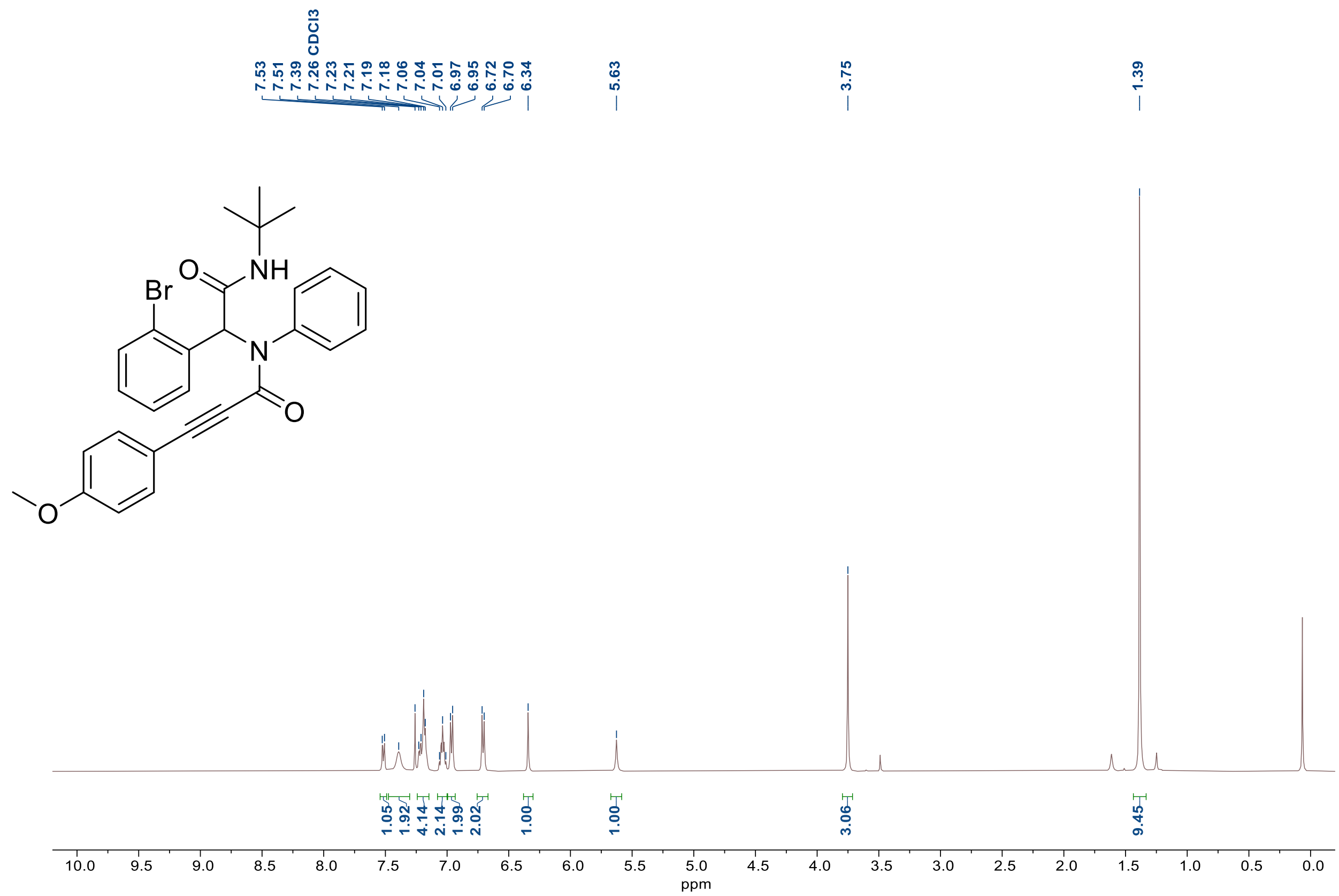

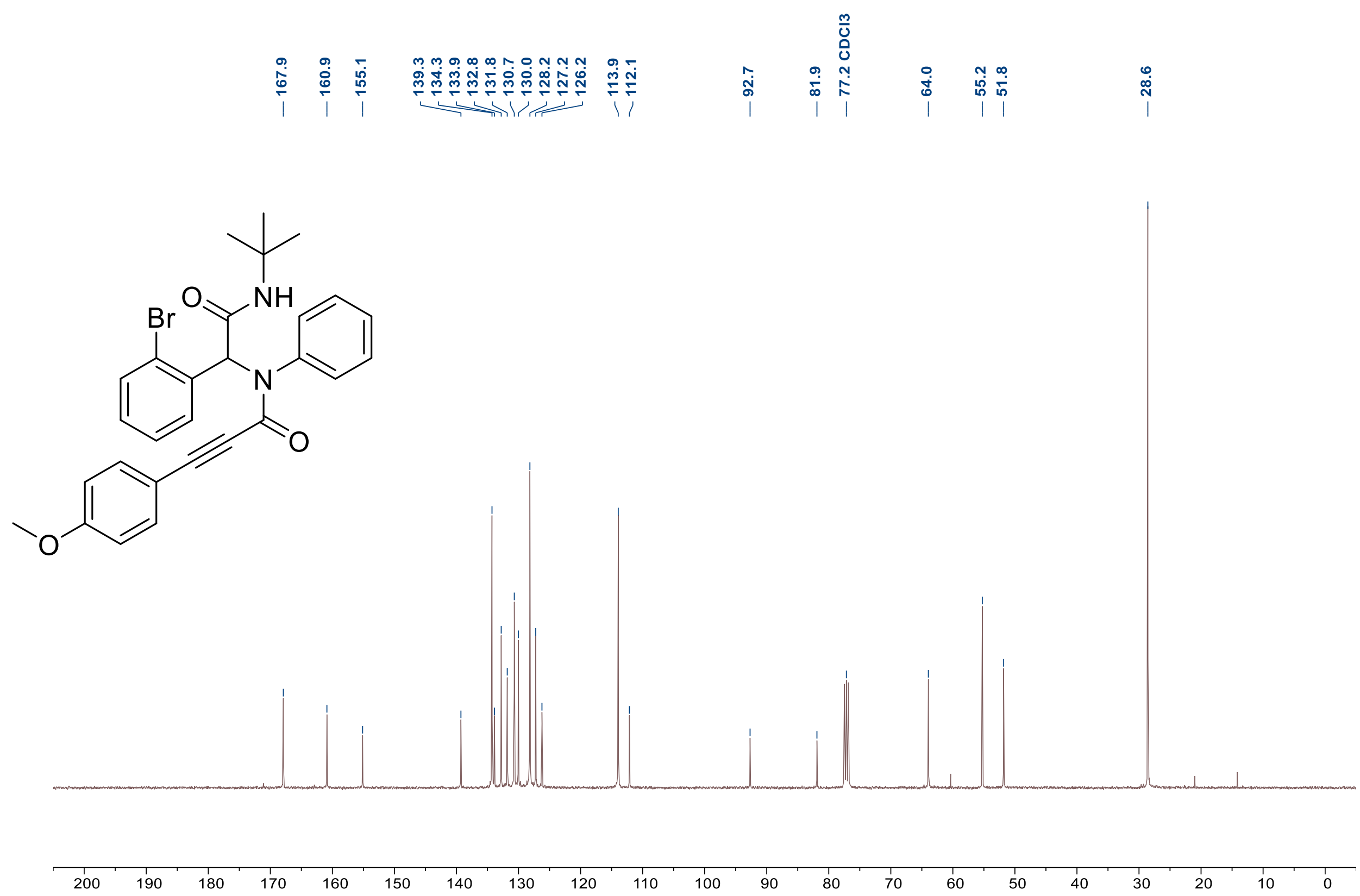

Copies of ^{1}H (500 MHz) and ^{13}C{^{1}H} (126 MHz) spectra of compound **27f**

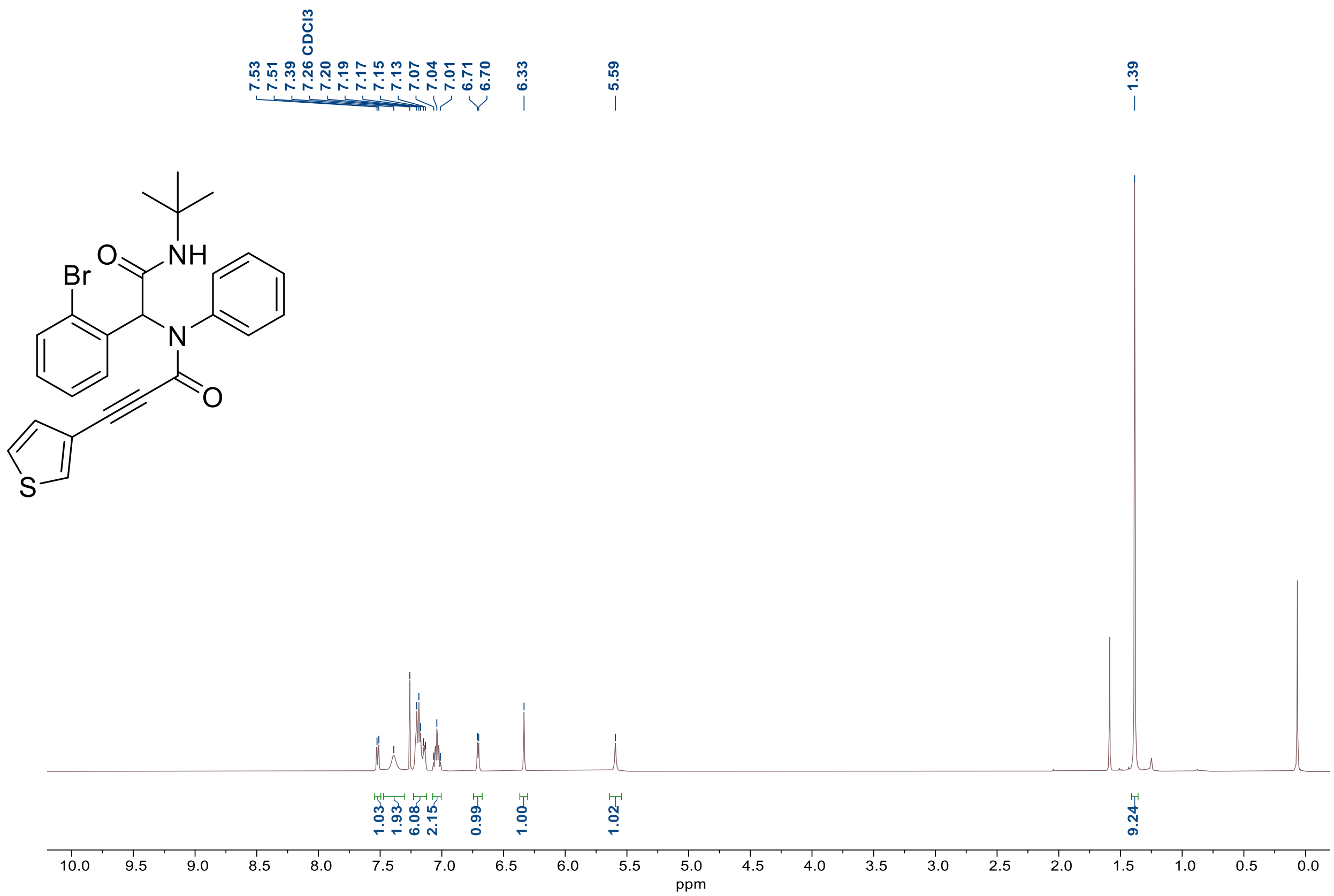

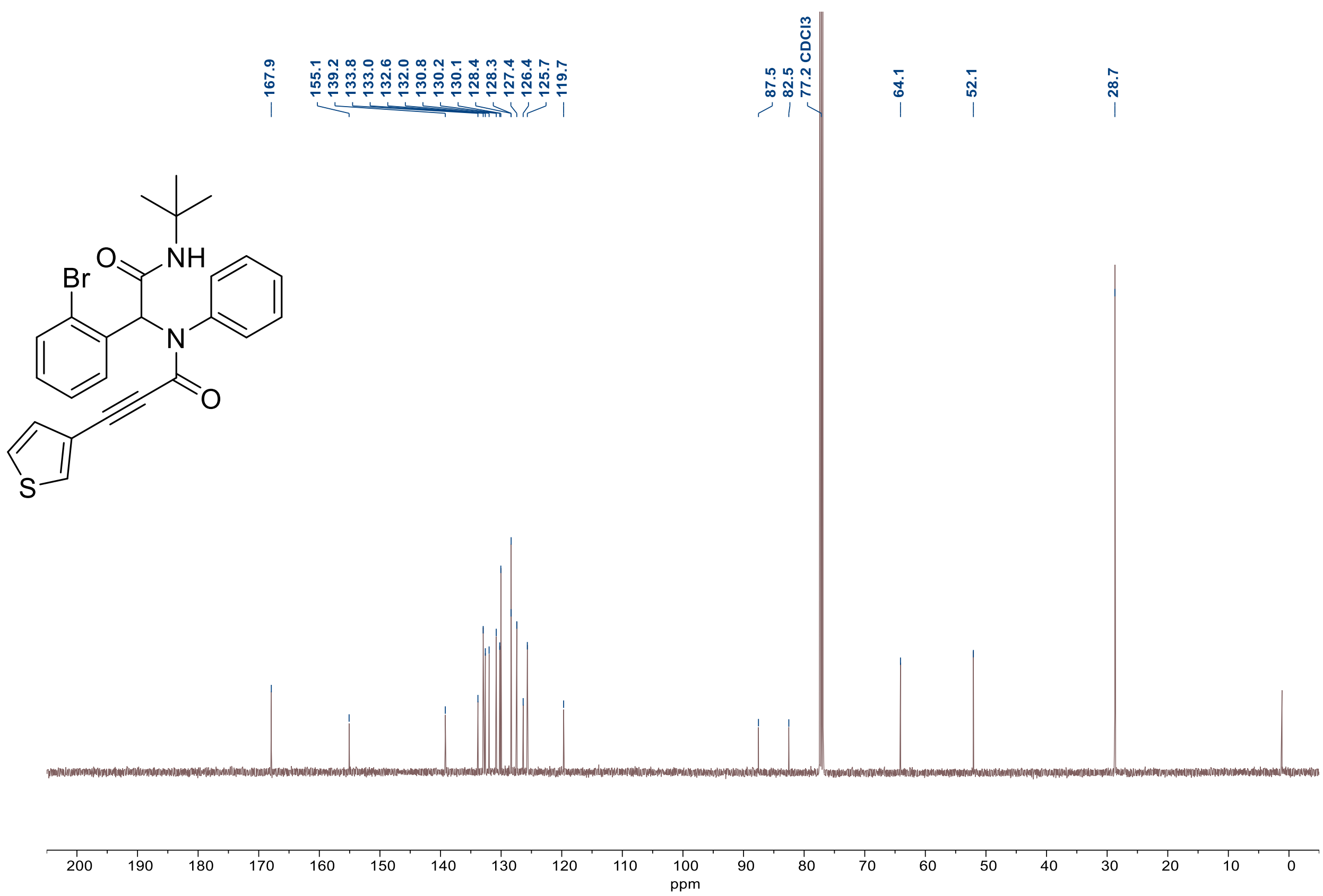

Copies of ^{1}H (500 MHz) and $^{13}C\{^{1}H\}$ (101 MHz) spectra of compound **27g**

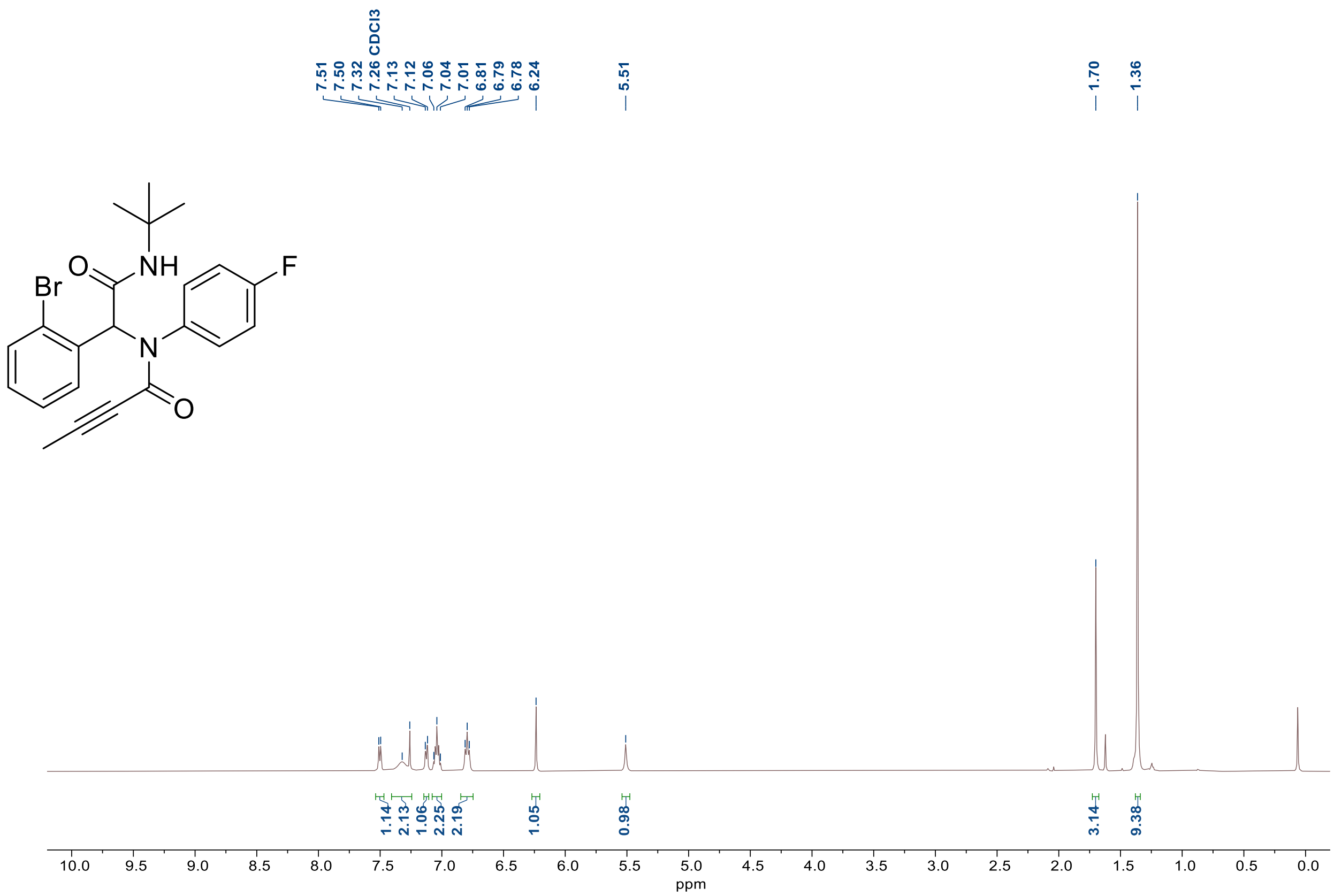

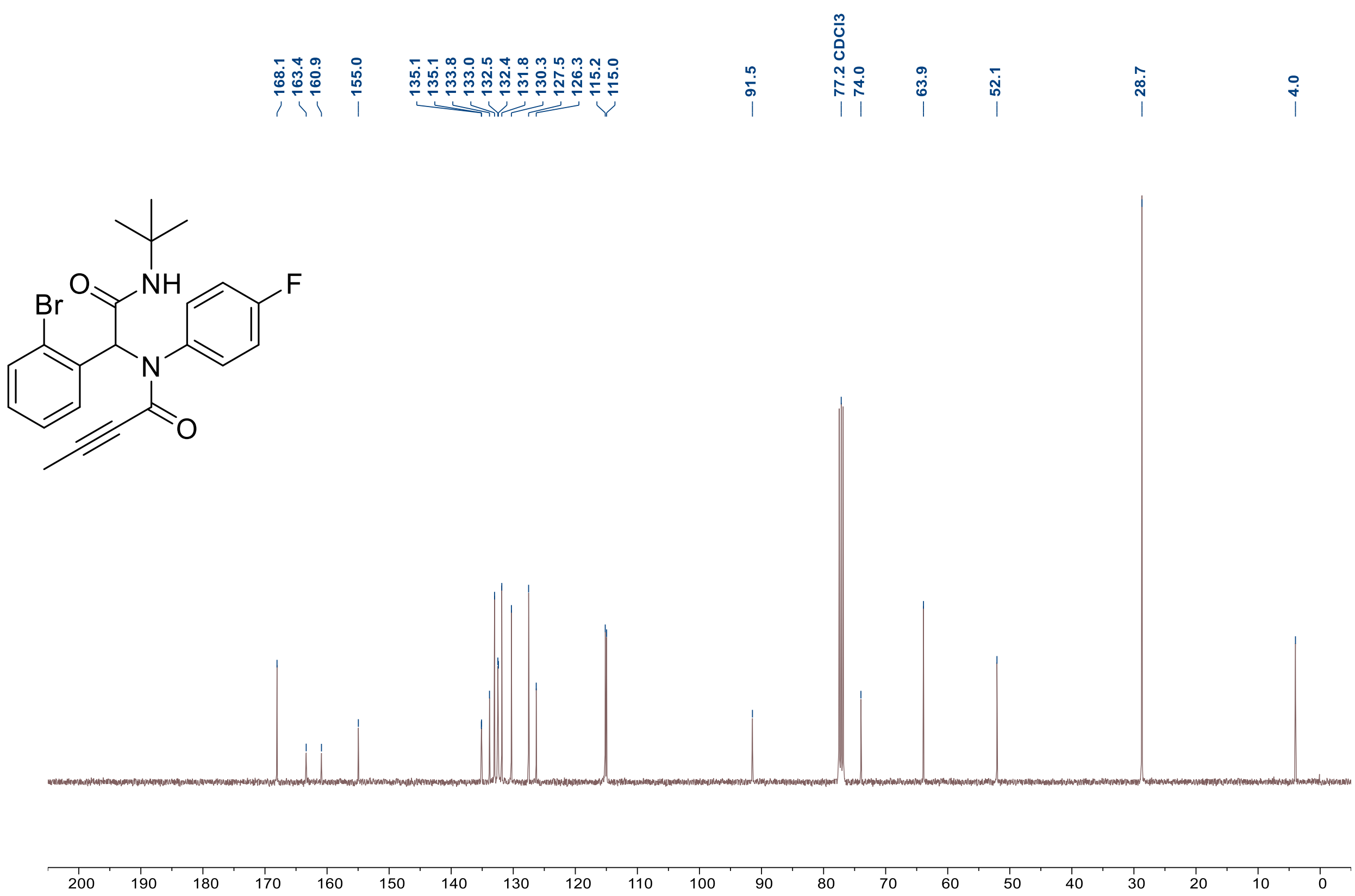

Copies of ^{1}H (400 MHz) and $^{13}C\{^{1}H\}$ (101 MHz) spectra of compound **27h**

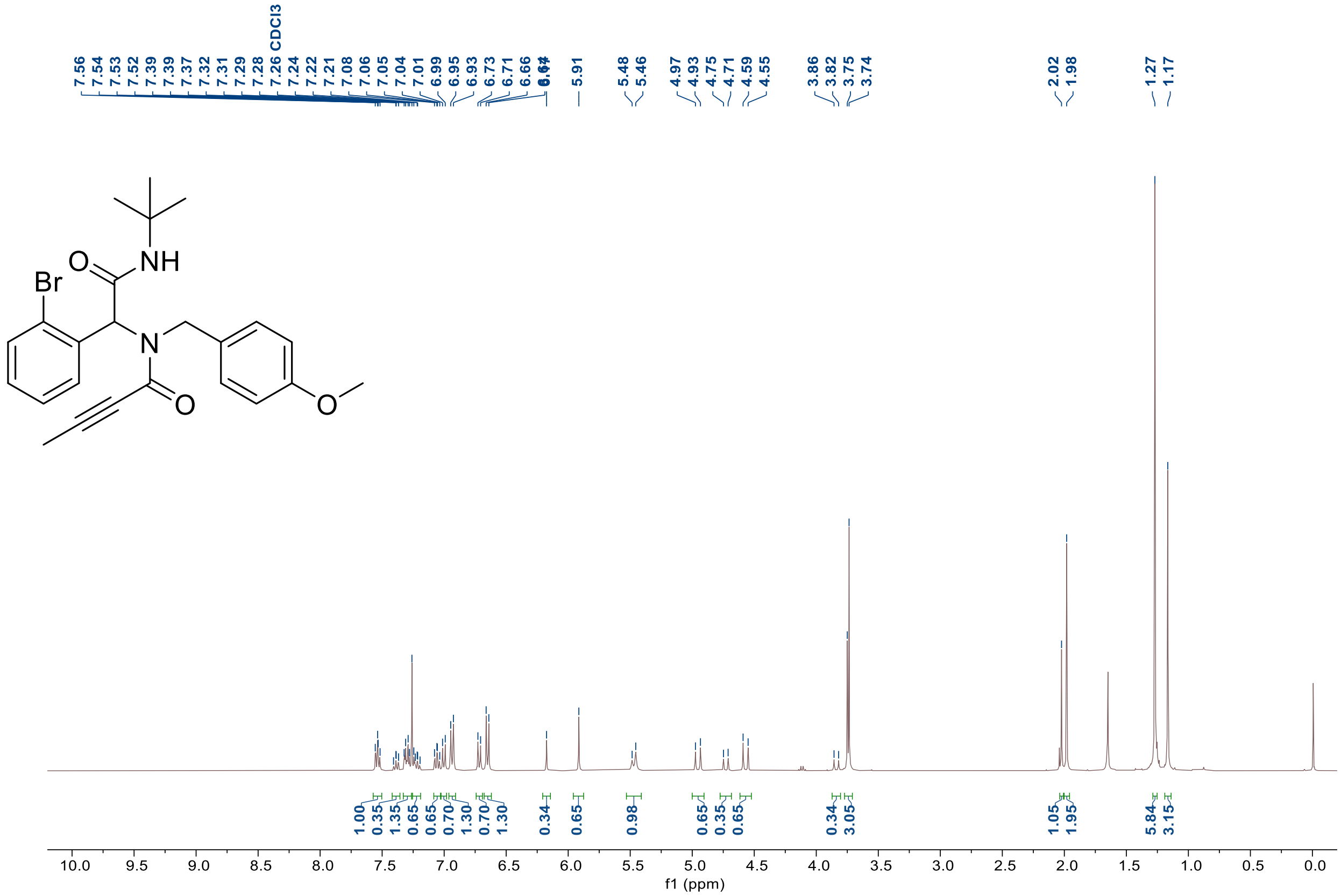

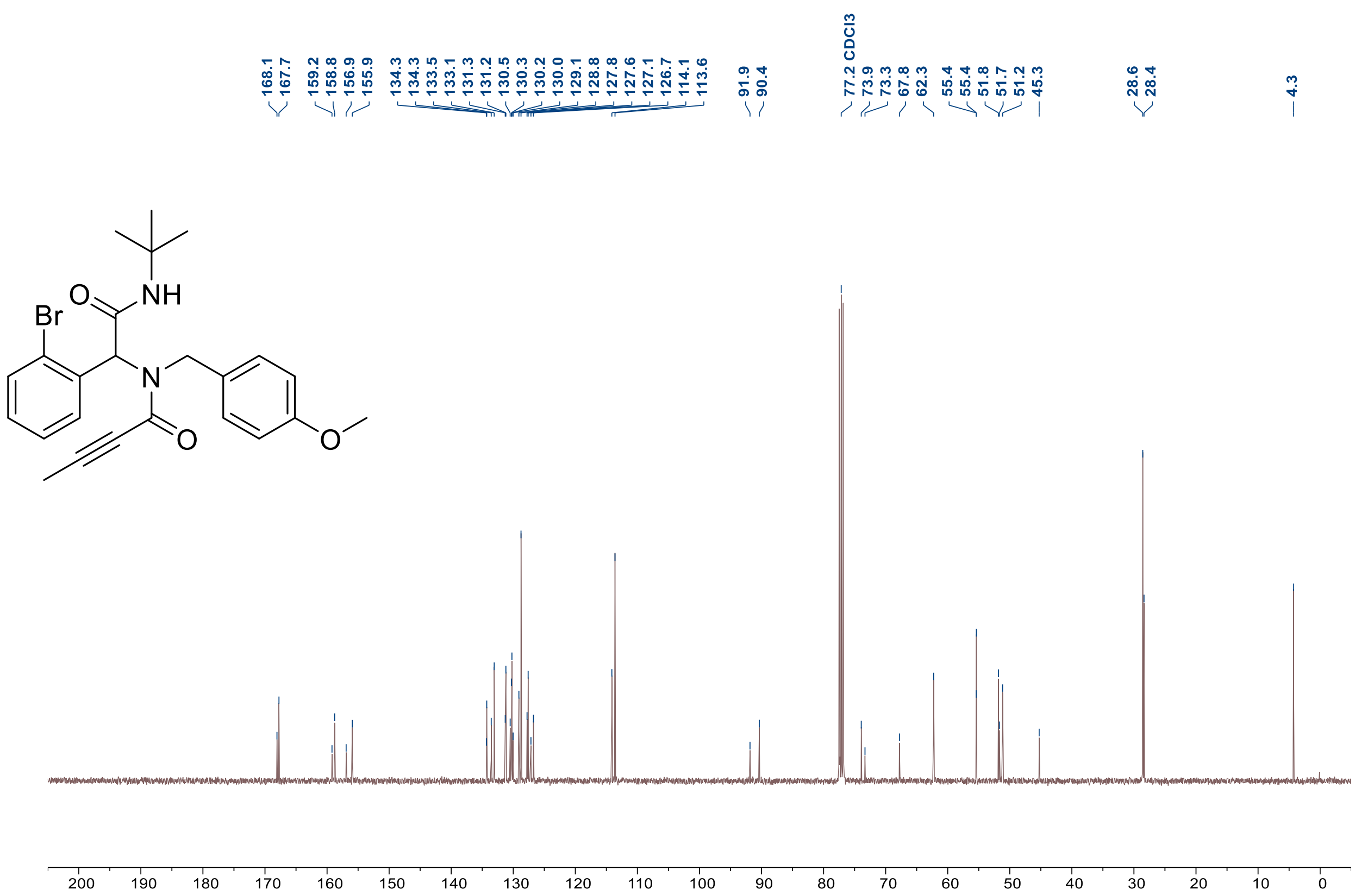

Copies of ^{1}H (500 MHz) and ^{13}C{^{1}H} (101 MHz) spectra of compound **27i**

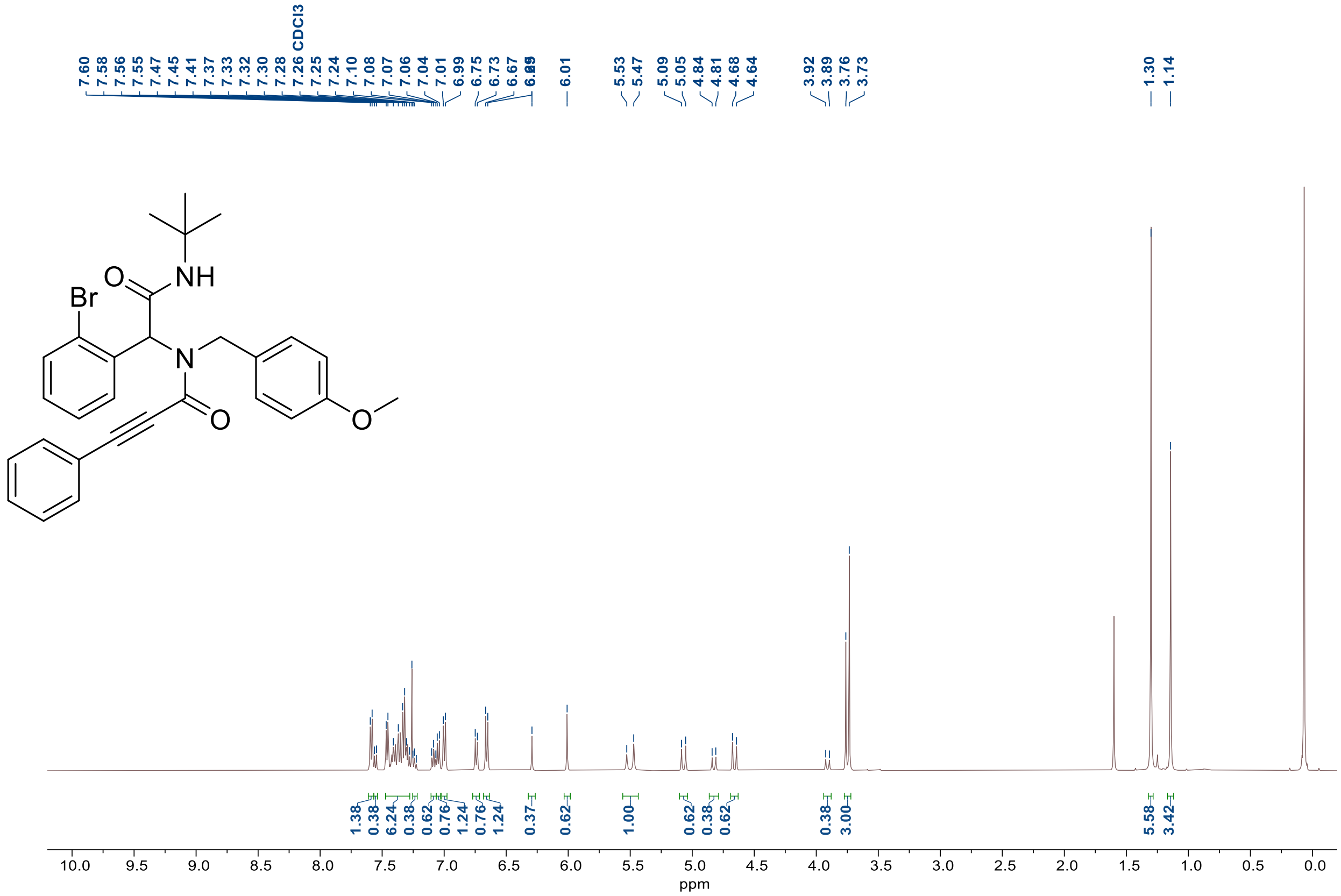

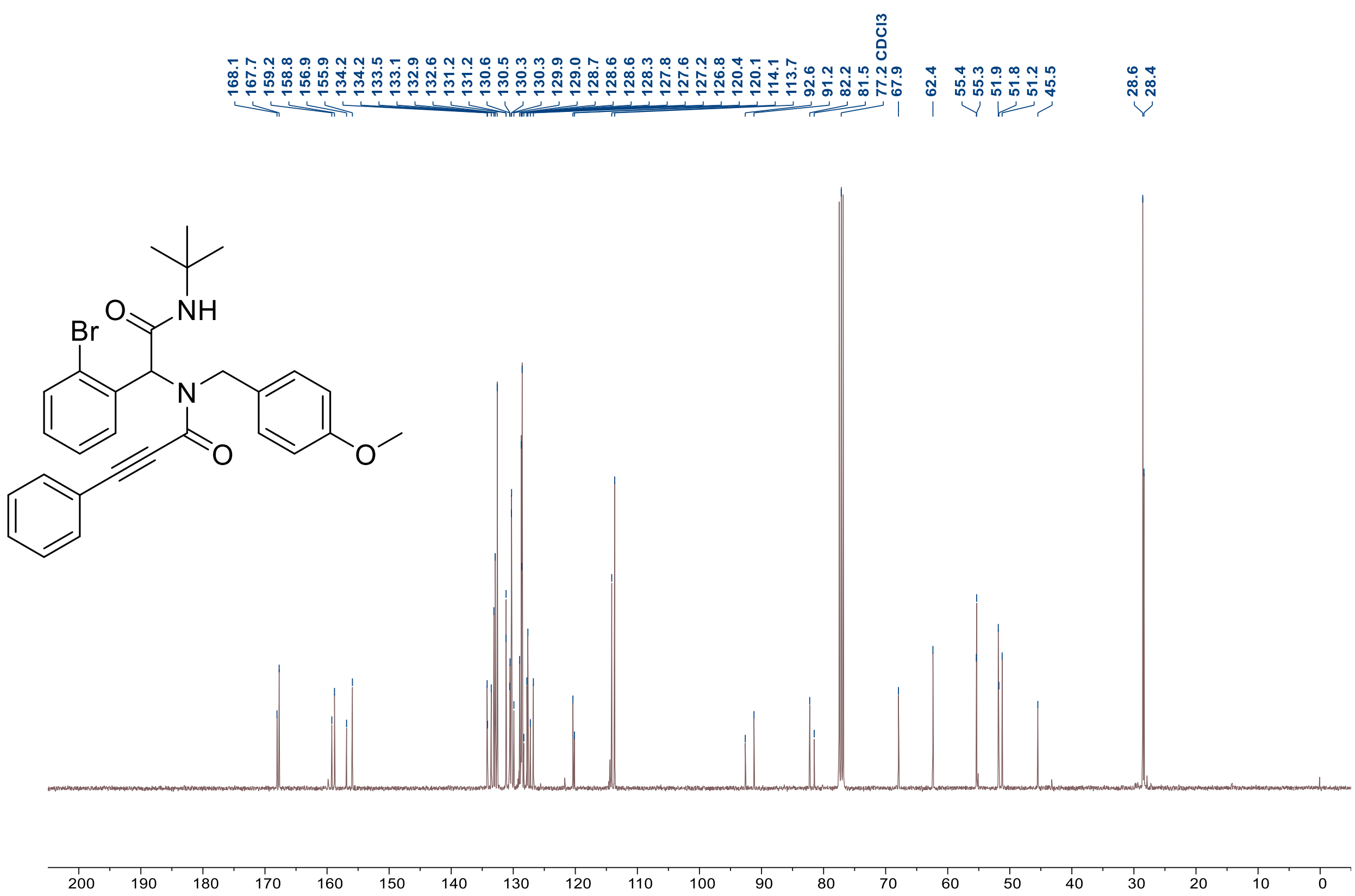

Copies of ^{1}H (400 MHz) and $^{13}C\{^{1}H\}$ (101 MHz) spectra of compound **27j**

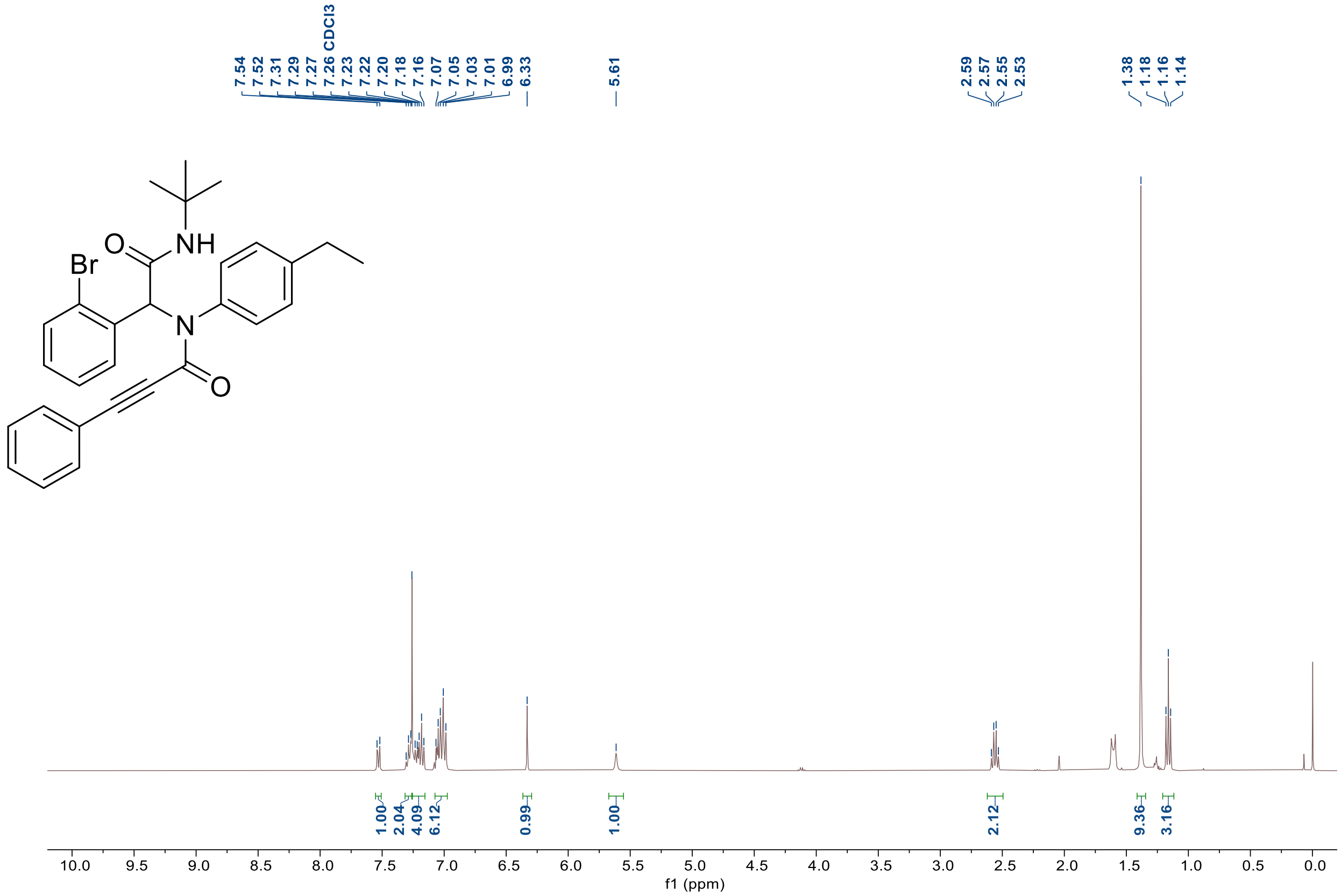

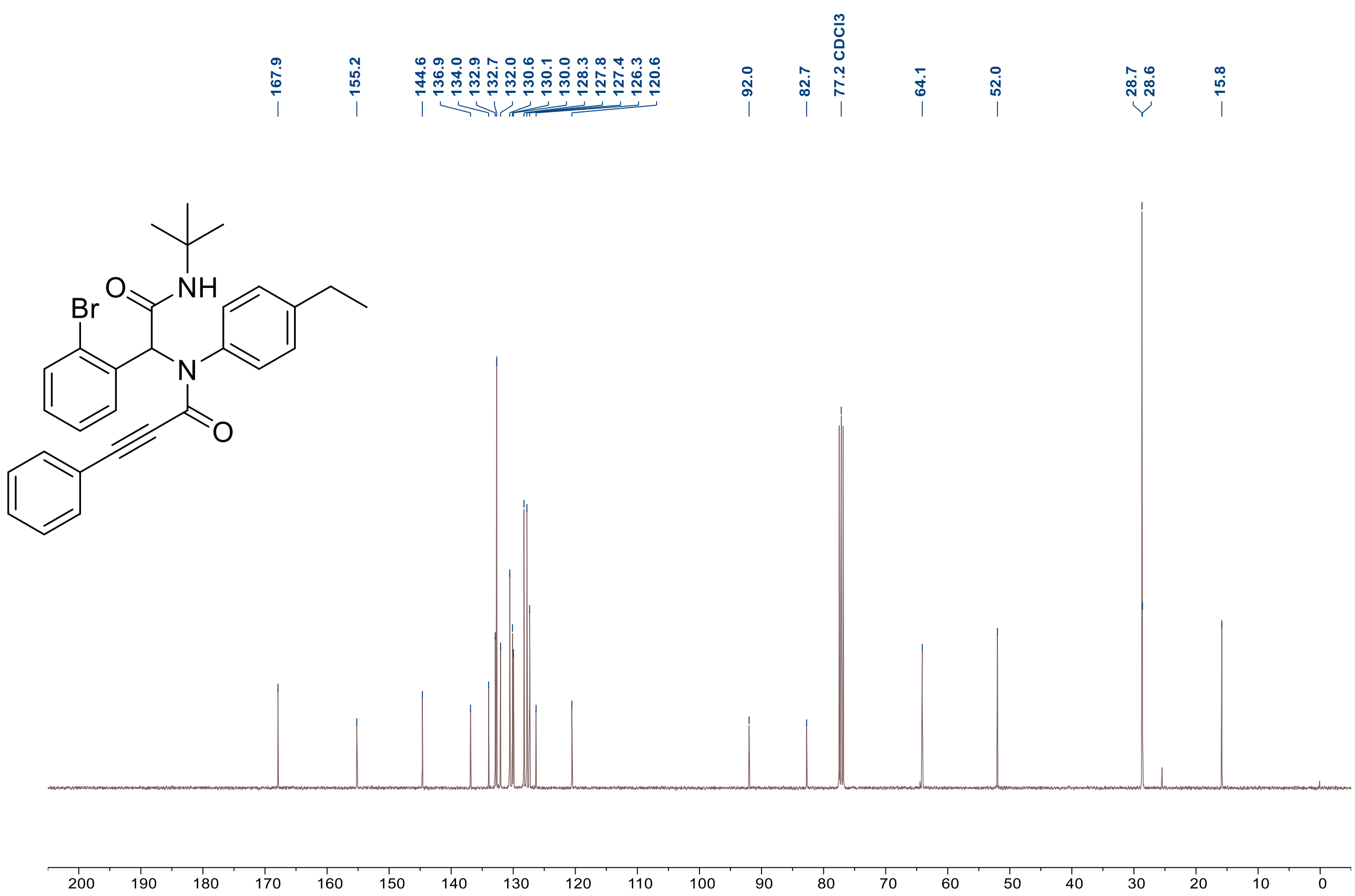

Copies of ^{1}H (400 MHz) and $^{13}C\{^{1}H\}$ (101 MHz) spectra of compound **27k**

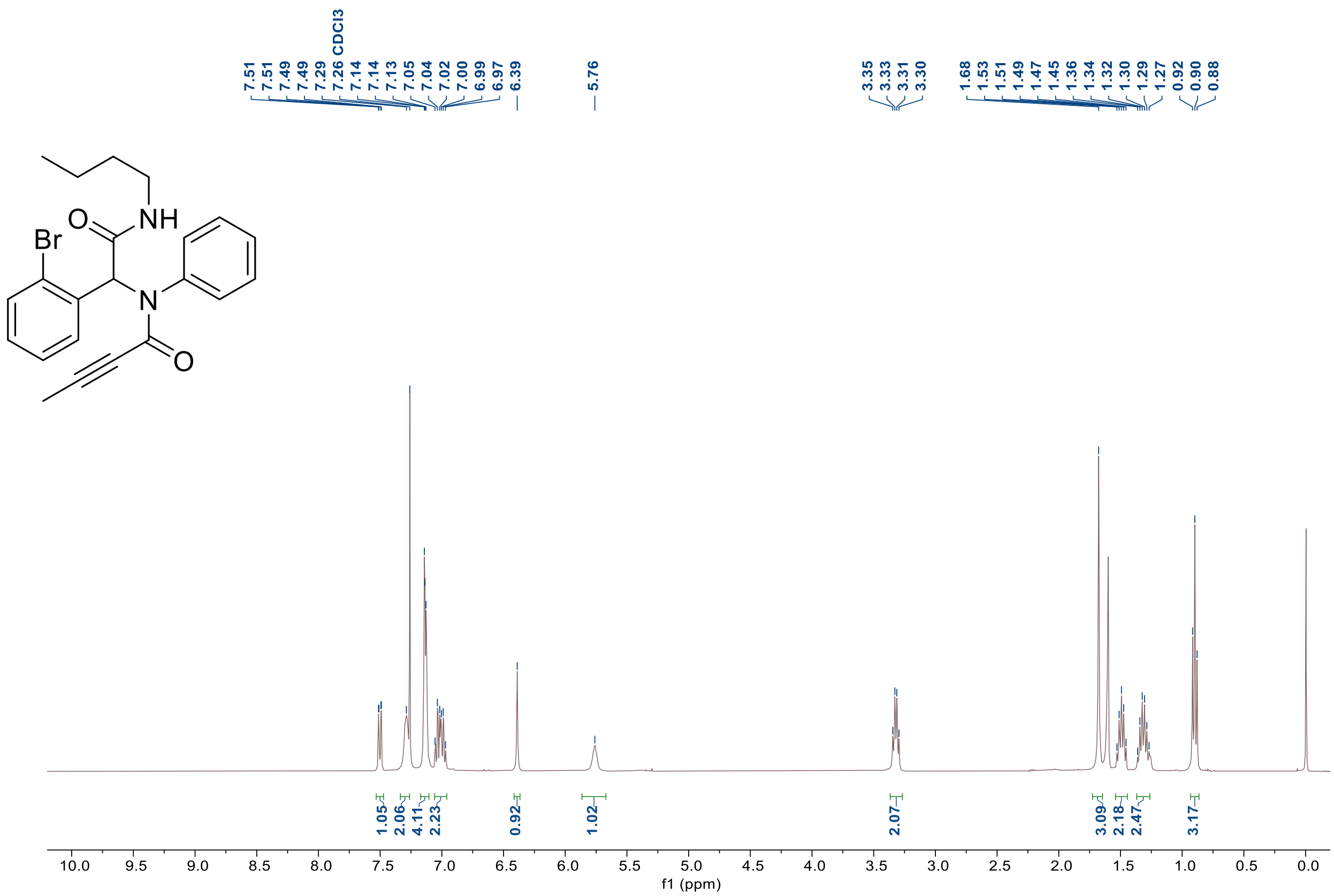

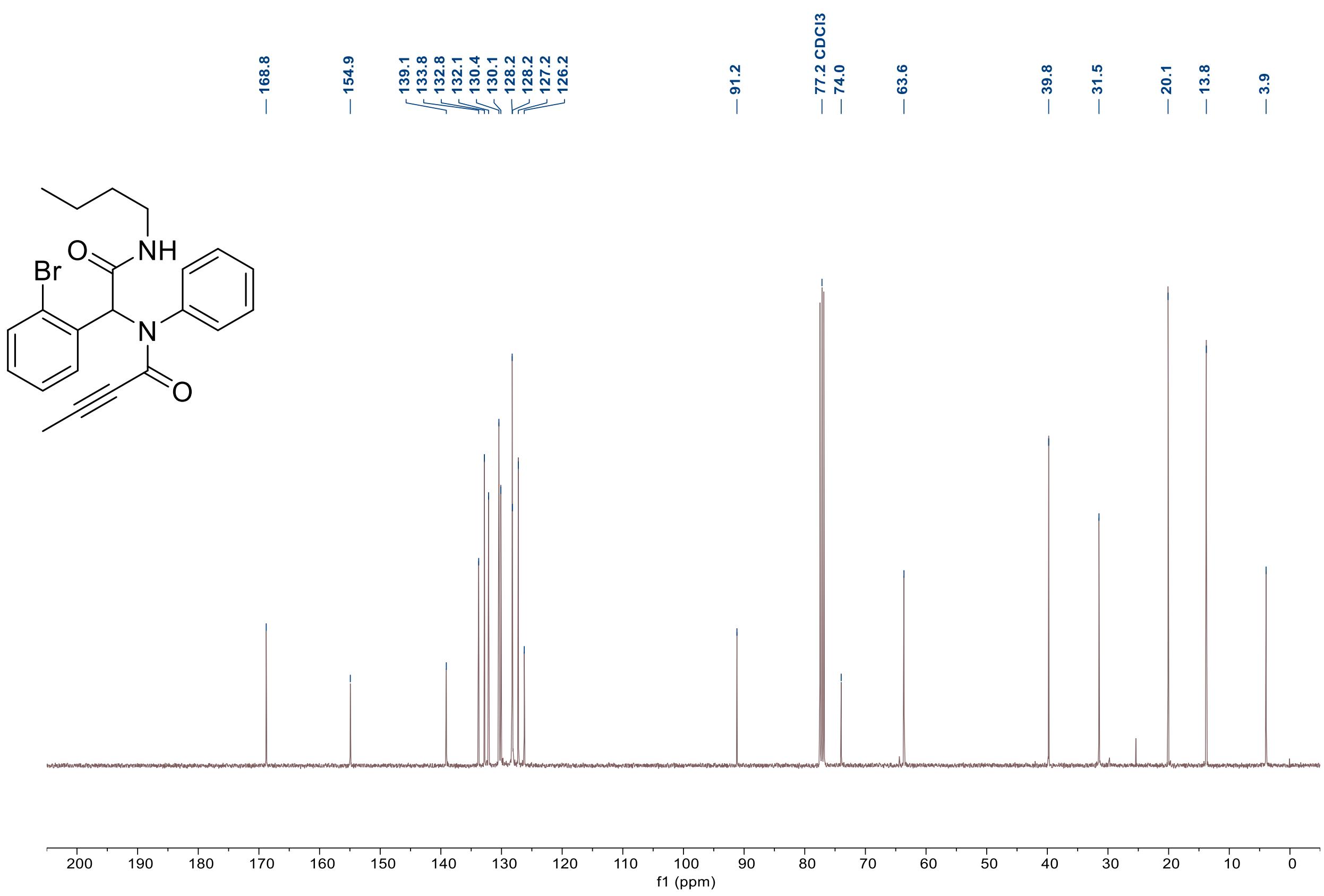

Copies of ^{1}H (500 MHz) and $^{13}C\{^{1}H\}$ (126 MHz) spectra of compound **27l**

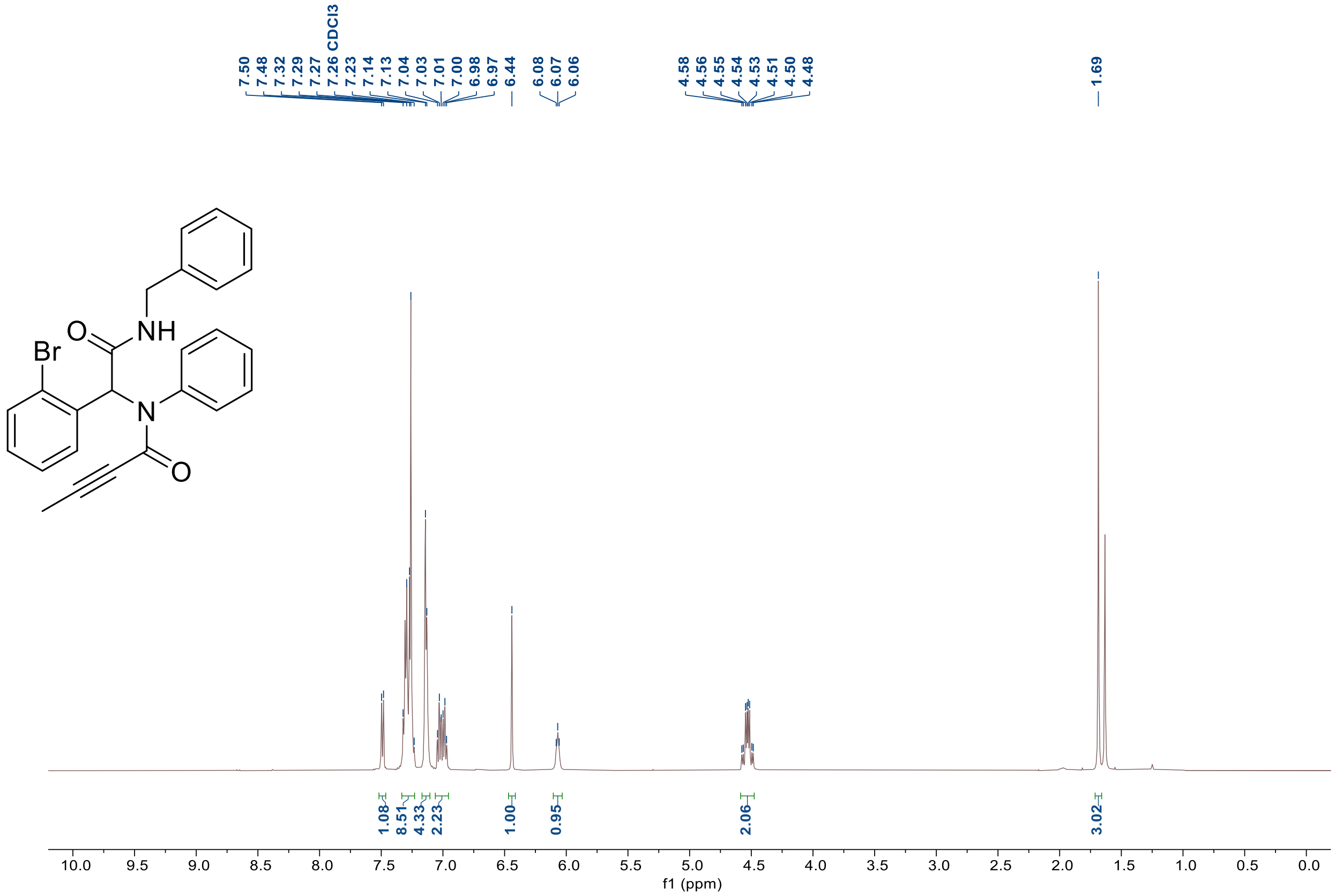

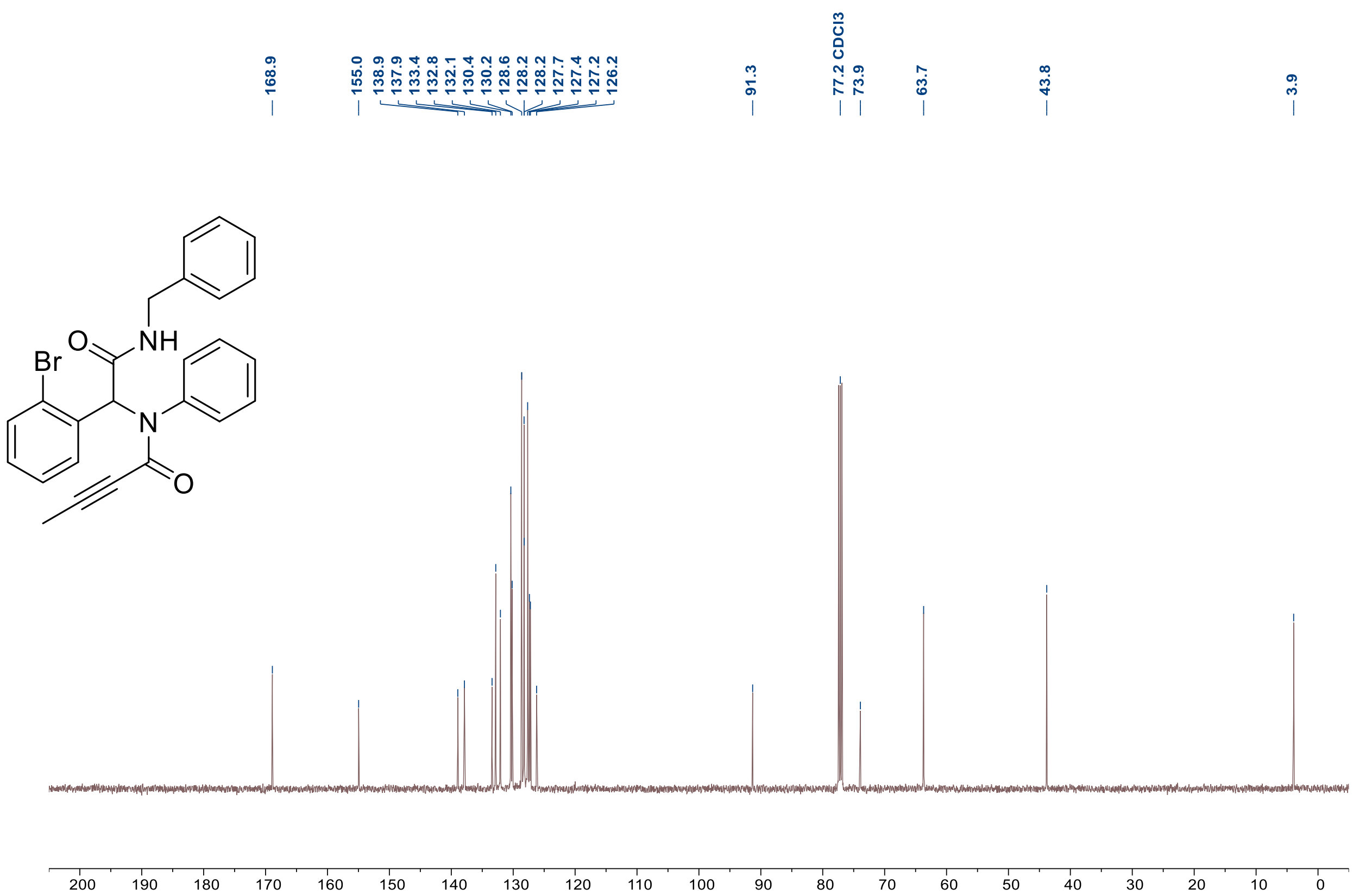

Copies of ^{1}H (500 MHz) and $^{13}C\{^{1}H\}$ (126 MHz) spectra of compound (*Z*)-**28a**

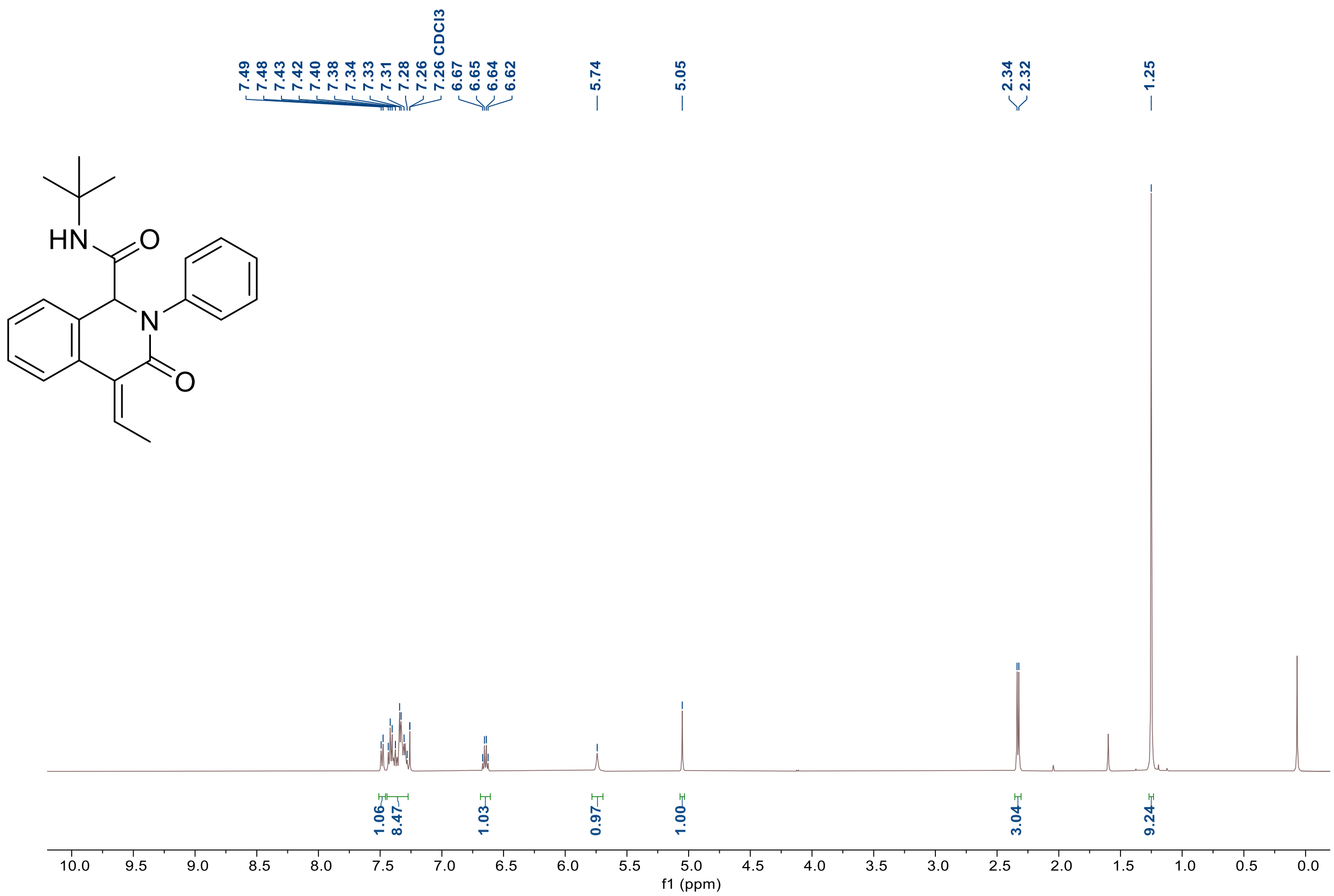

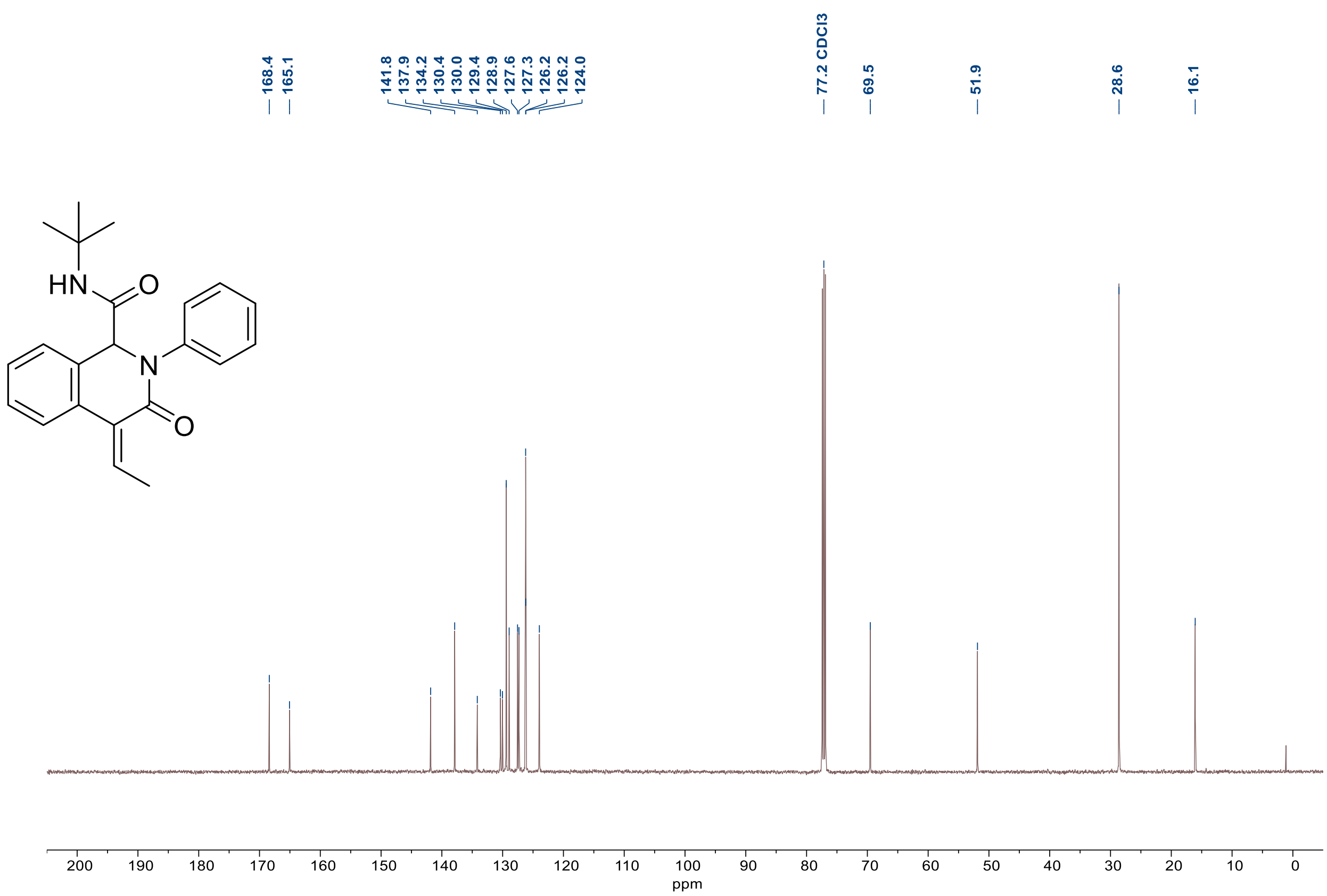

Copies of ^1H (500 MHz) and $^{13}C\{^1H\}$ (126 MHz) spectra of compound (*E*)-**28a**

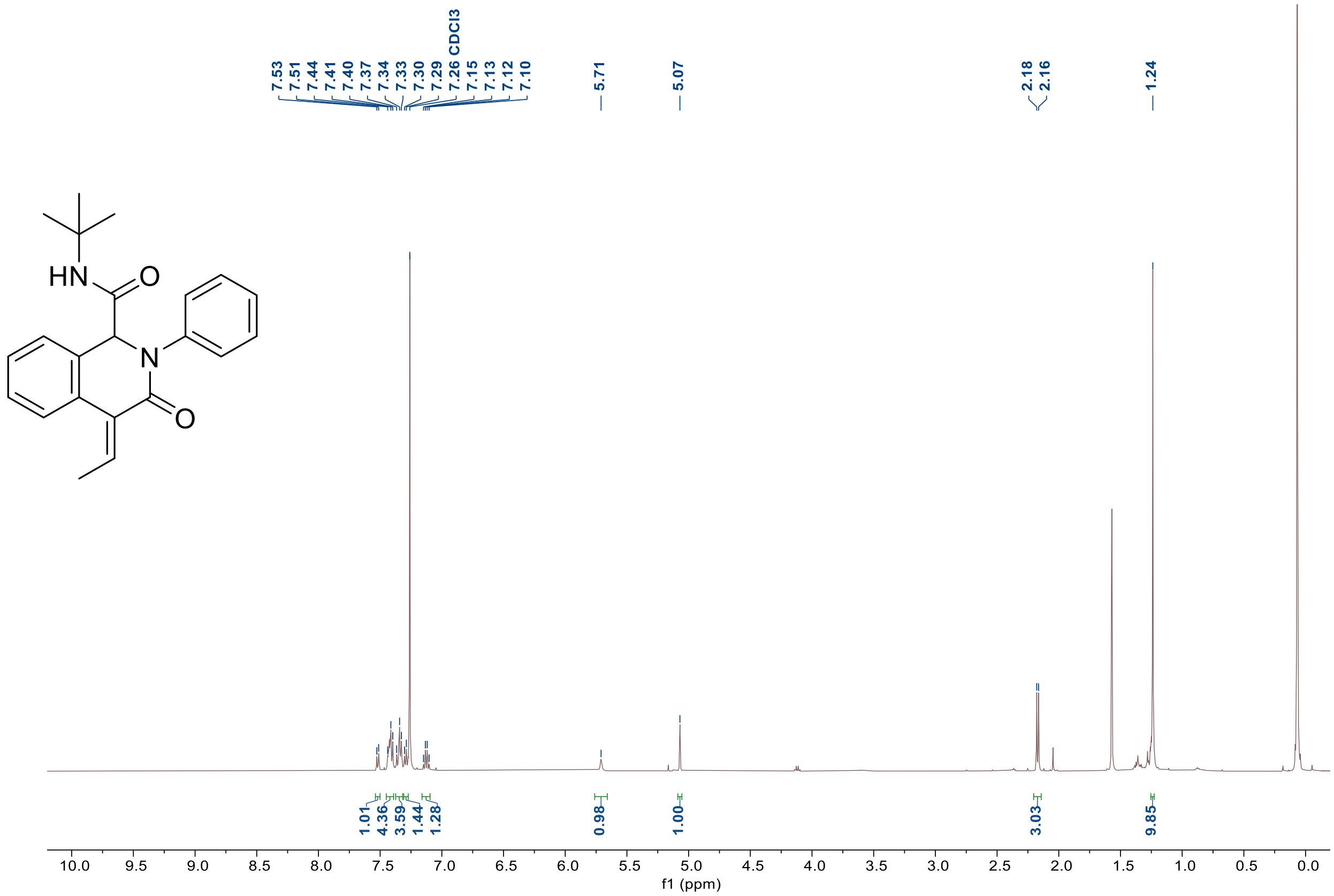

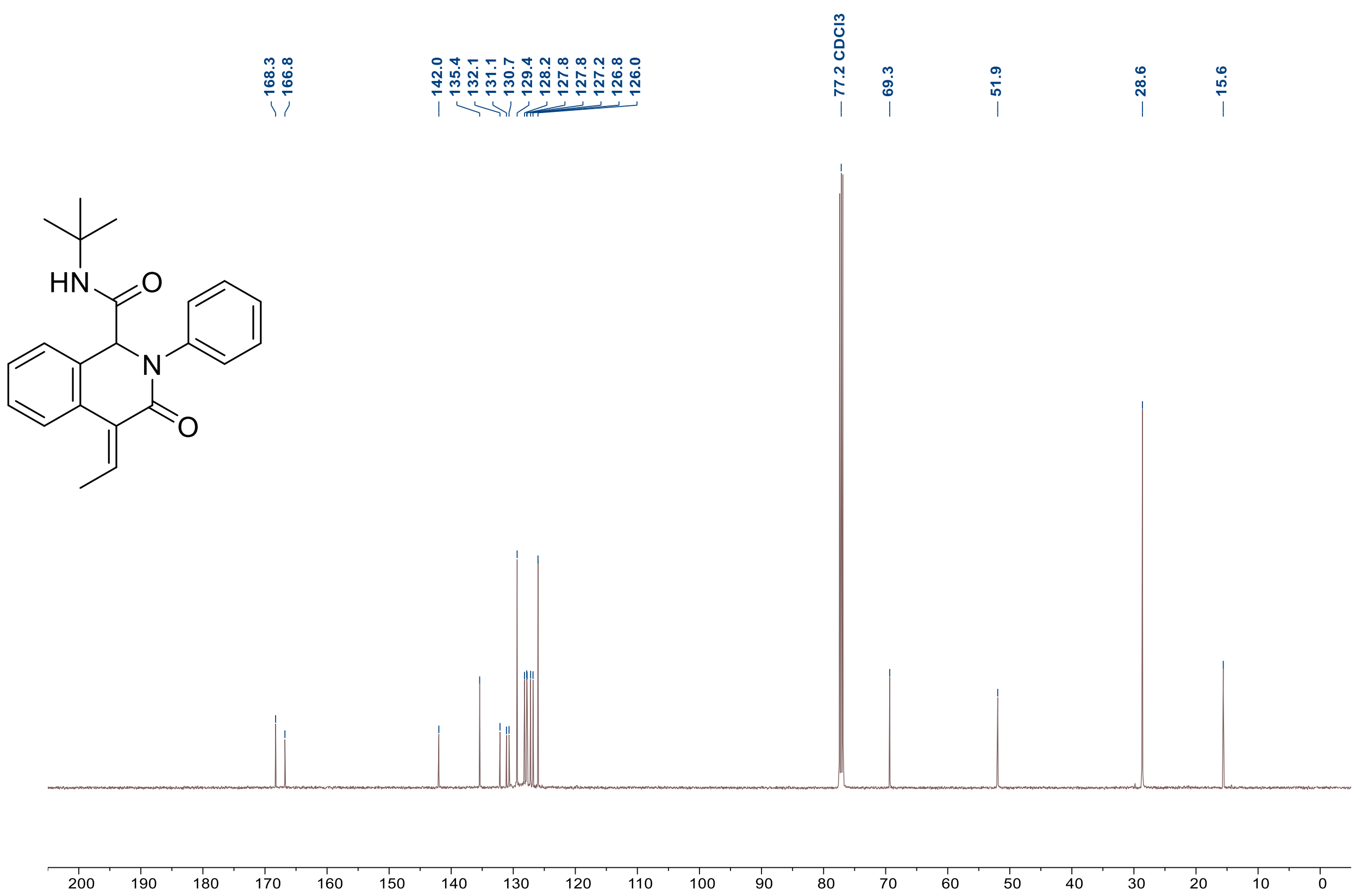

Copies of ^{1}H (500 MHz) and $^{13}C\{^{1}H\}$ (126 MHz) spectra of compound (*Z*)-**28b**

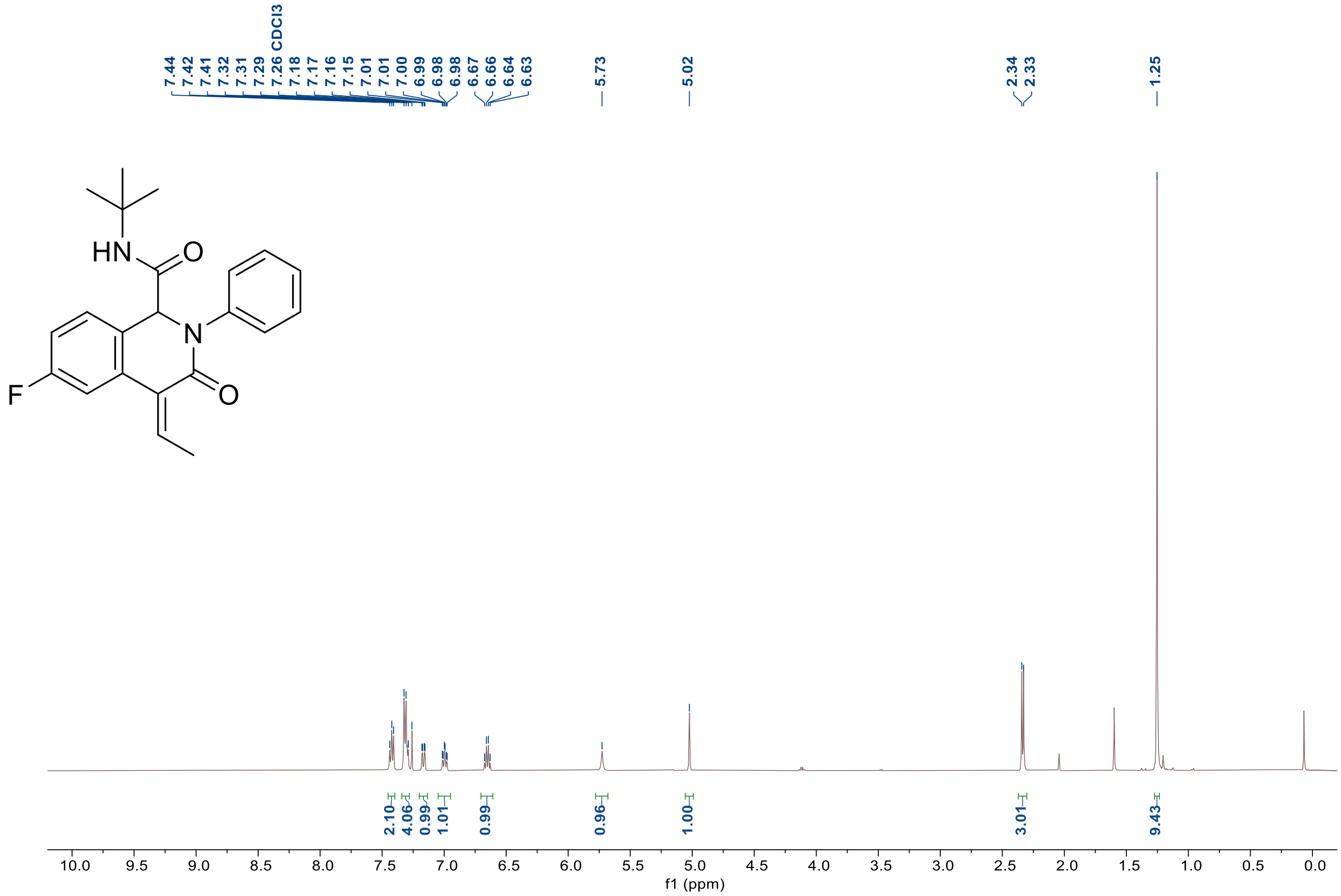

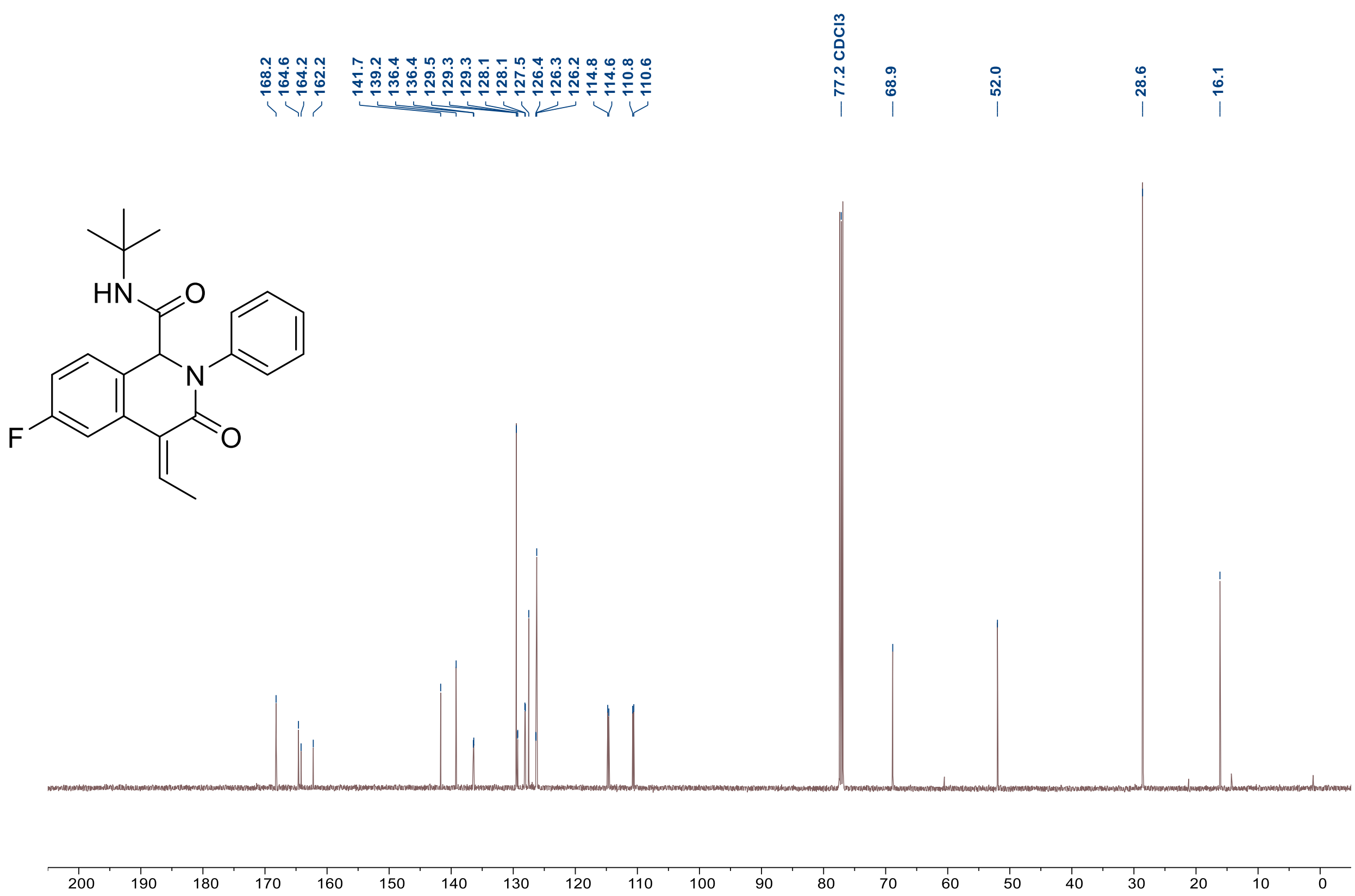

Copies of ^{1}H (500 MHz) and $^{13}C\{^{1}H\}$ (126 MHz) spectra of compound (*E*)-**28b**

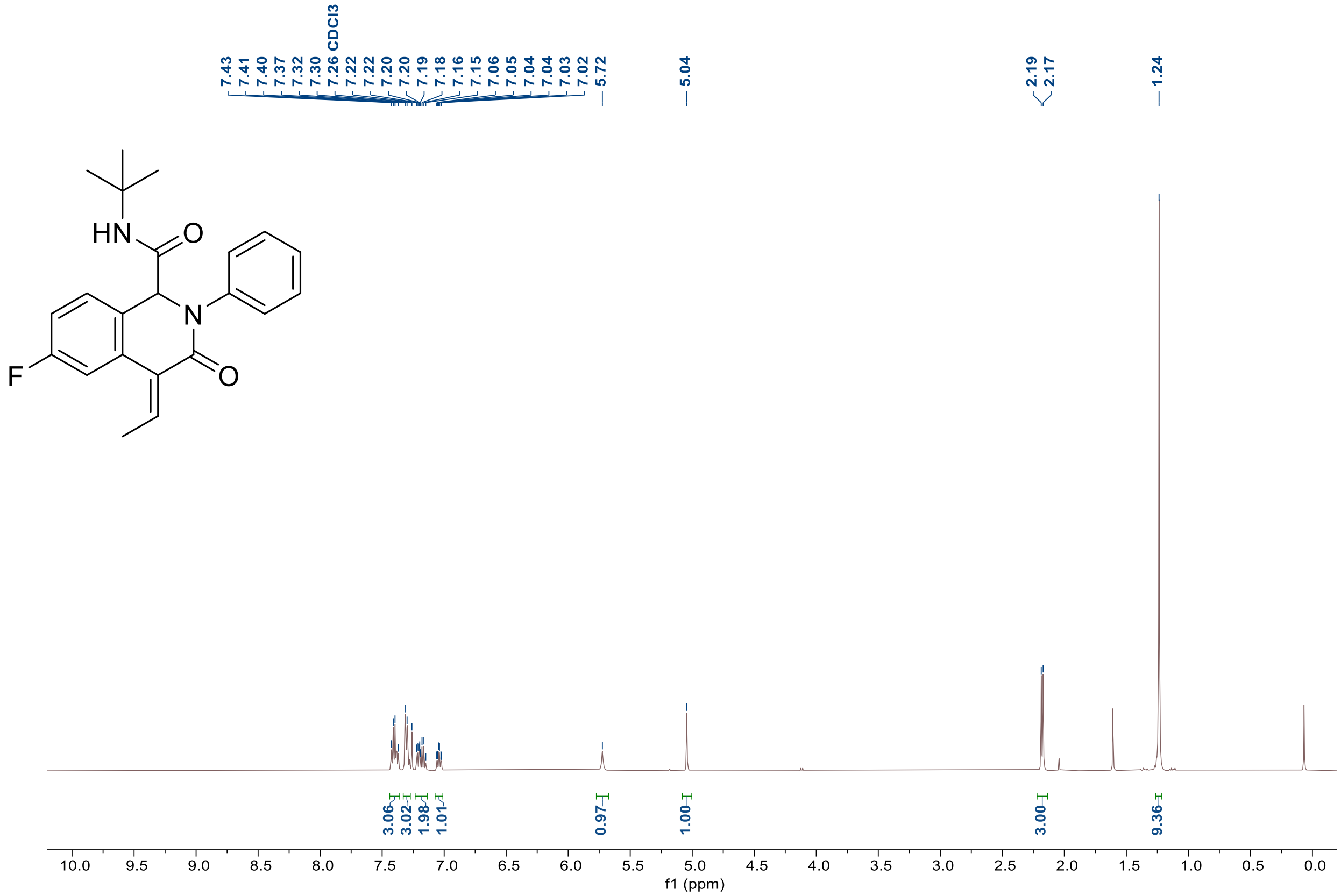

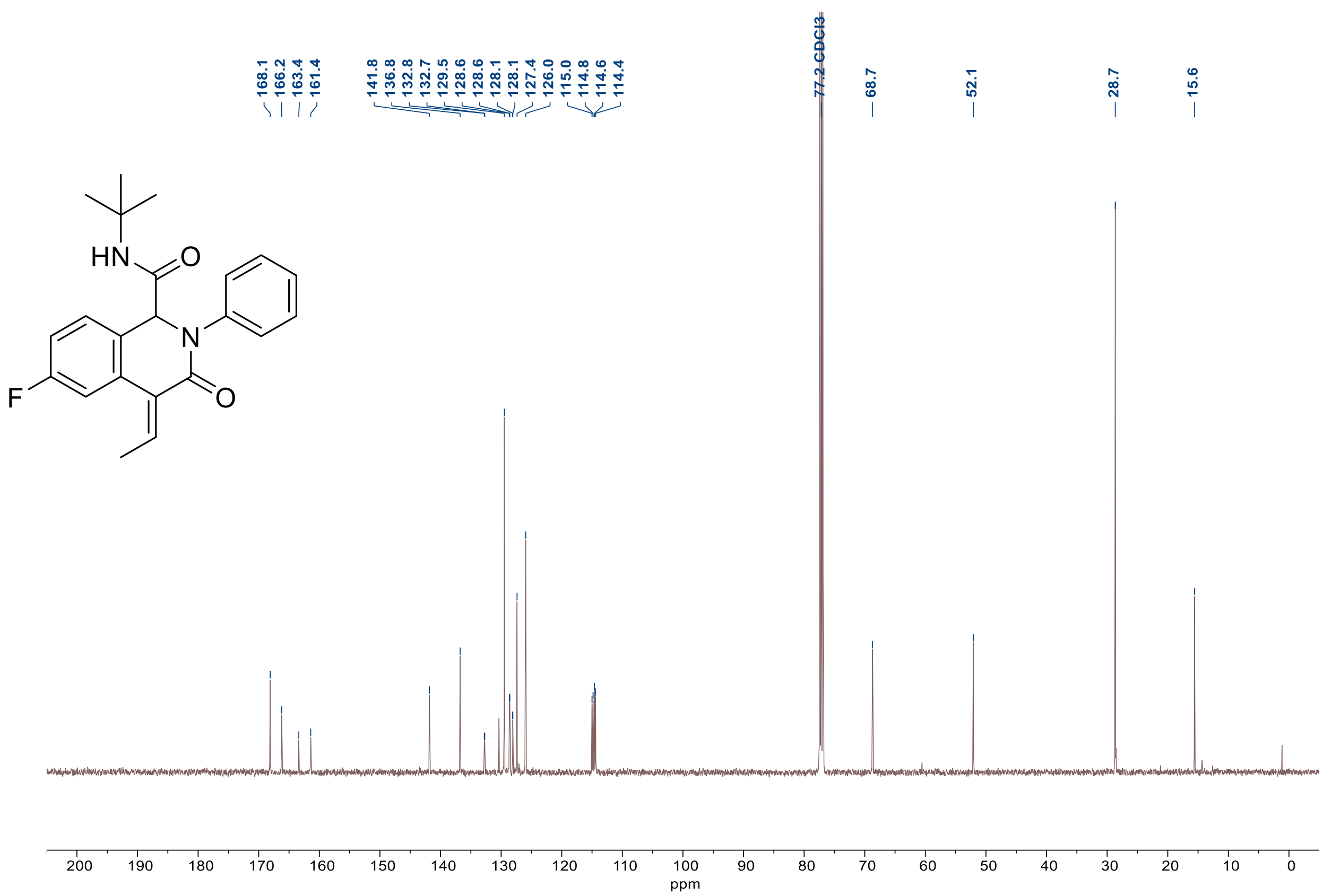

Copies of ^{1}H (500 MHz) and $^{13}C\{^{1}H\}$ (126 MHz) spectra of compound (*Z*)-**28c**

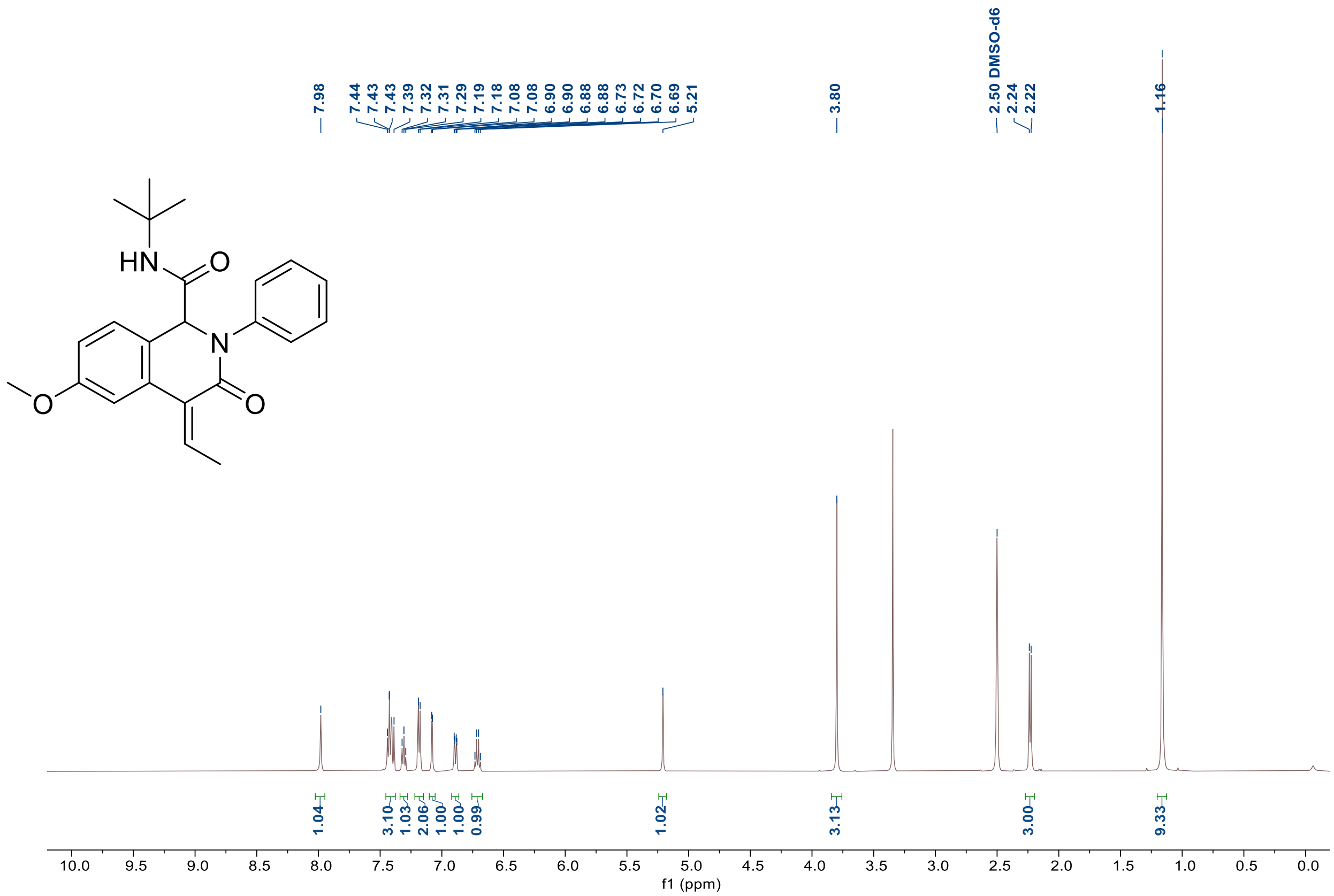

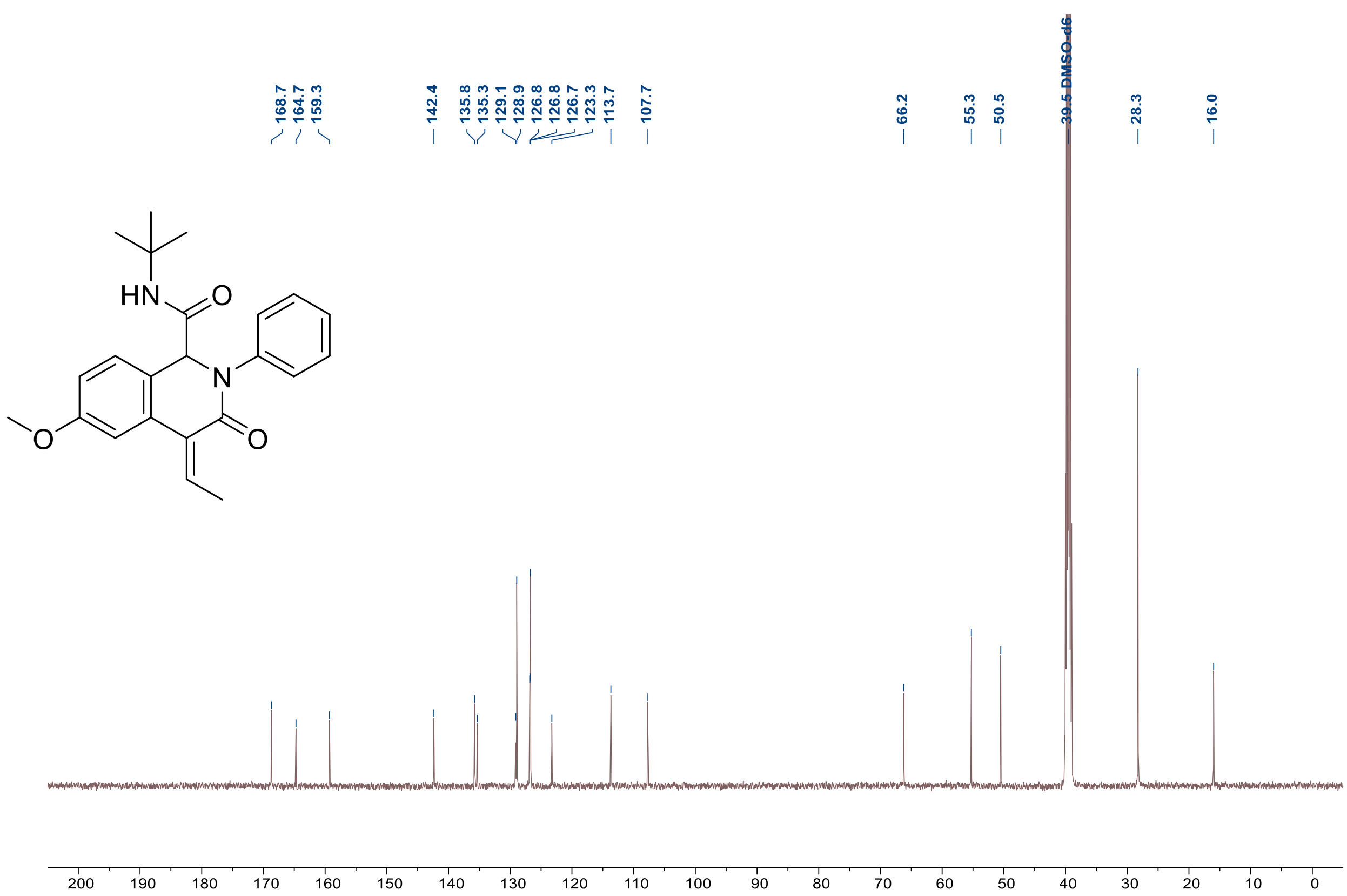

Copies of ^{1}H (500 MHz) and $^{13}C\{^{1}H\}$ (126 MHz) spectra of Z/E mixture of **28d**

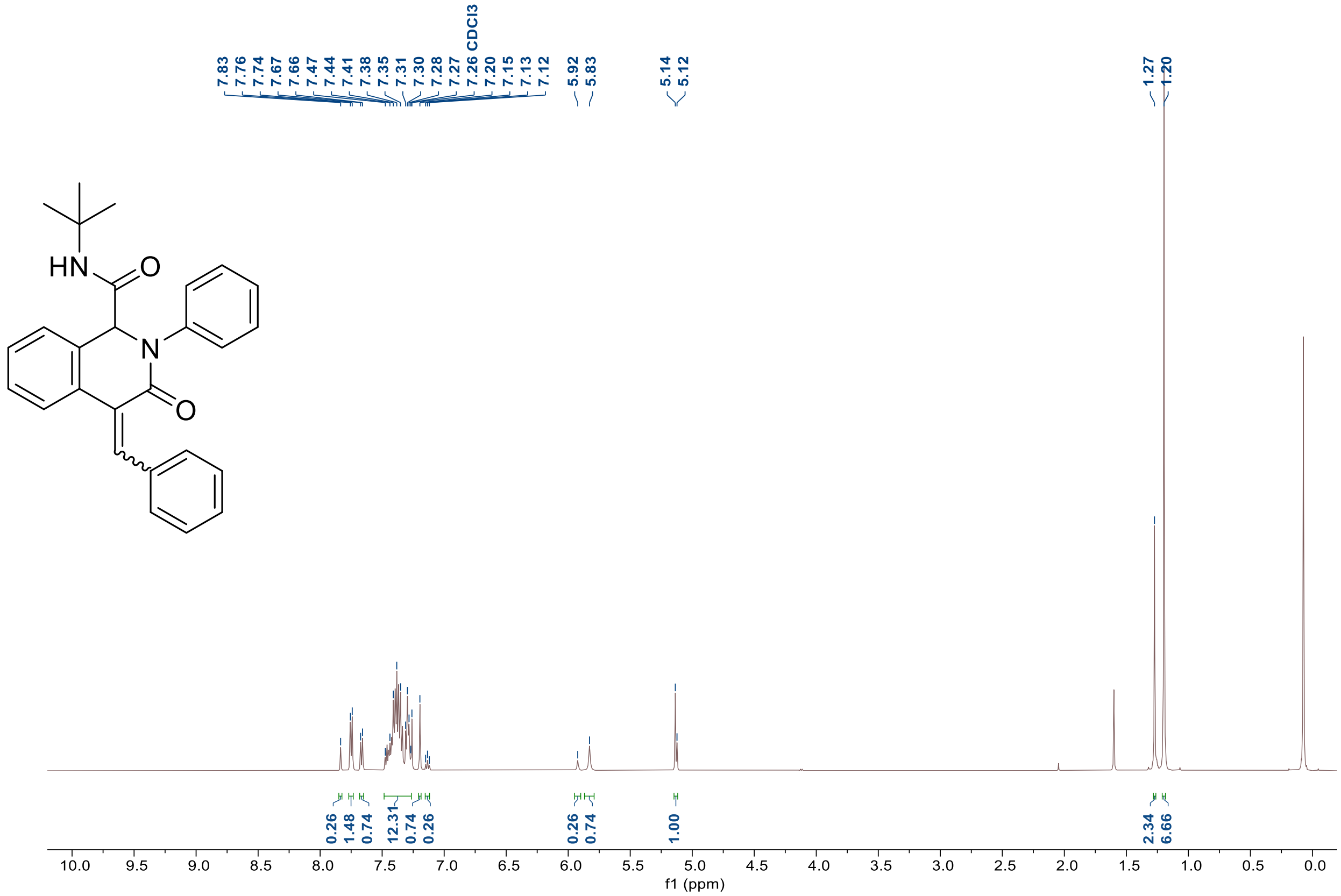

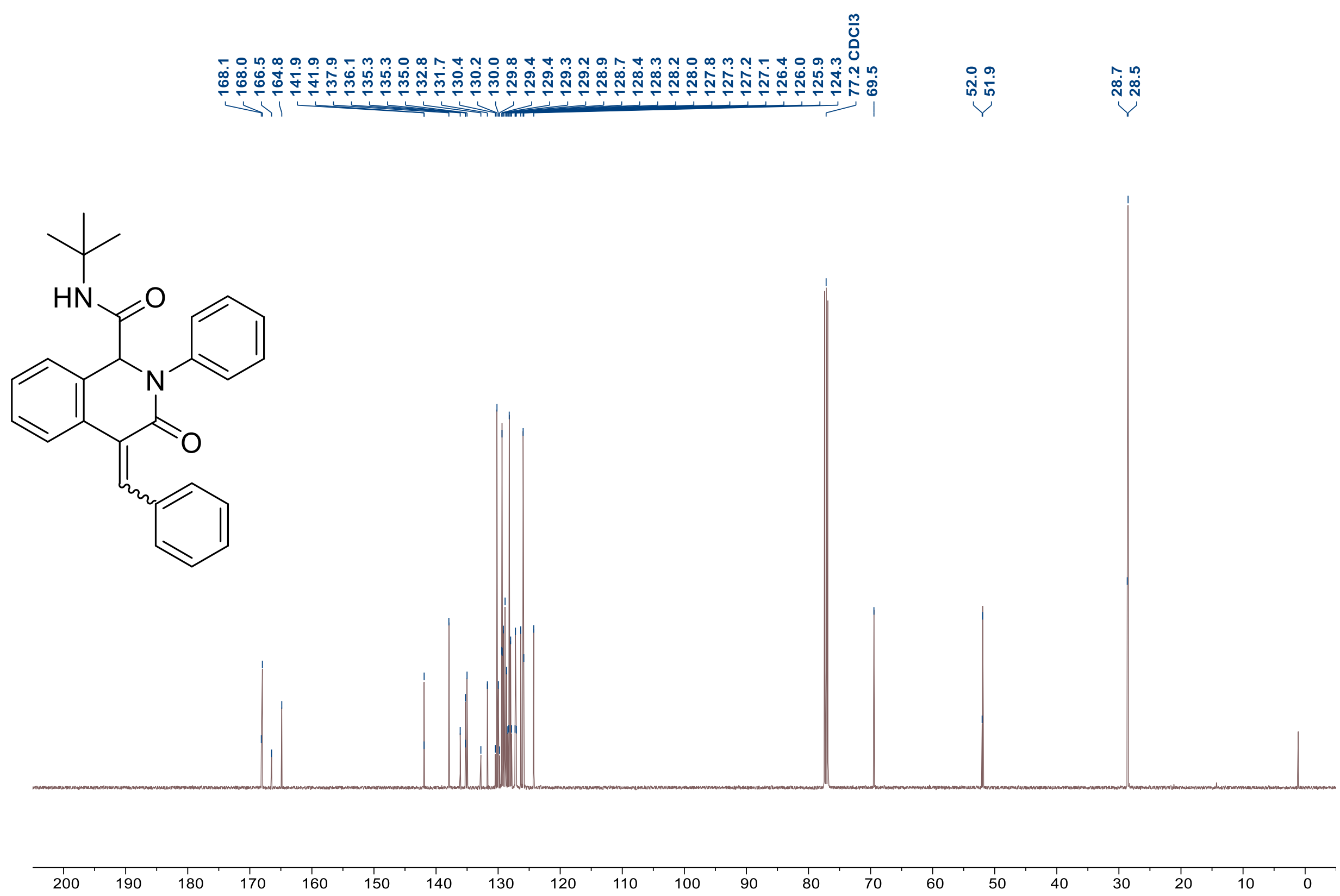

Copies of ^{1}H (500 MHz) and ^{13}C{^{1}H} (126 MHz) spectra of compound (*Z*)-**28e**

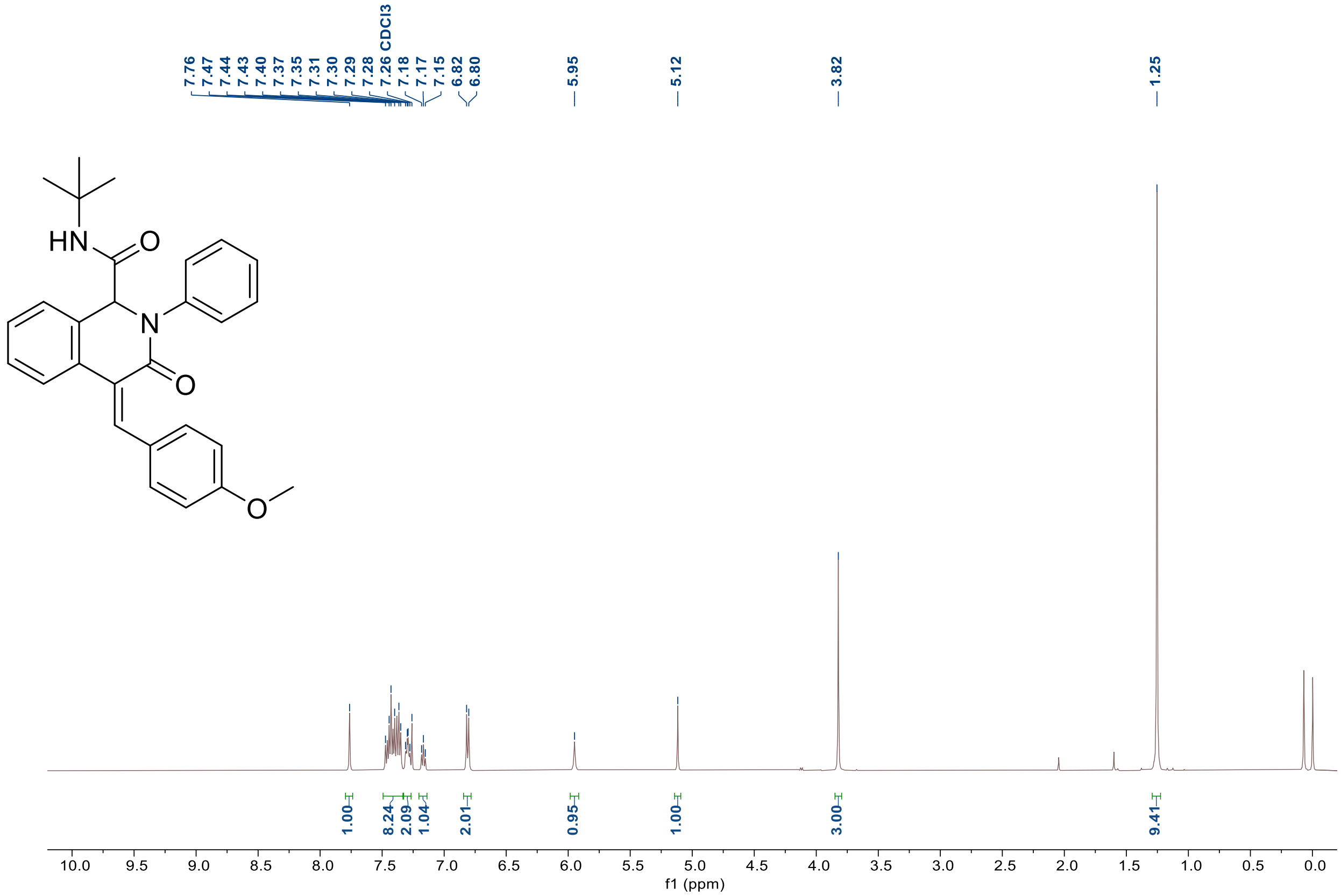

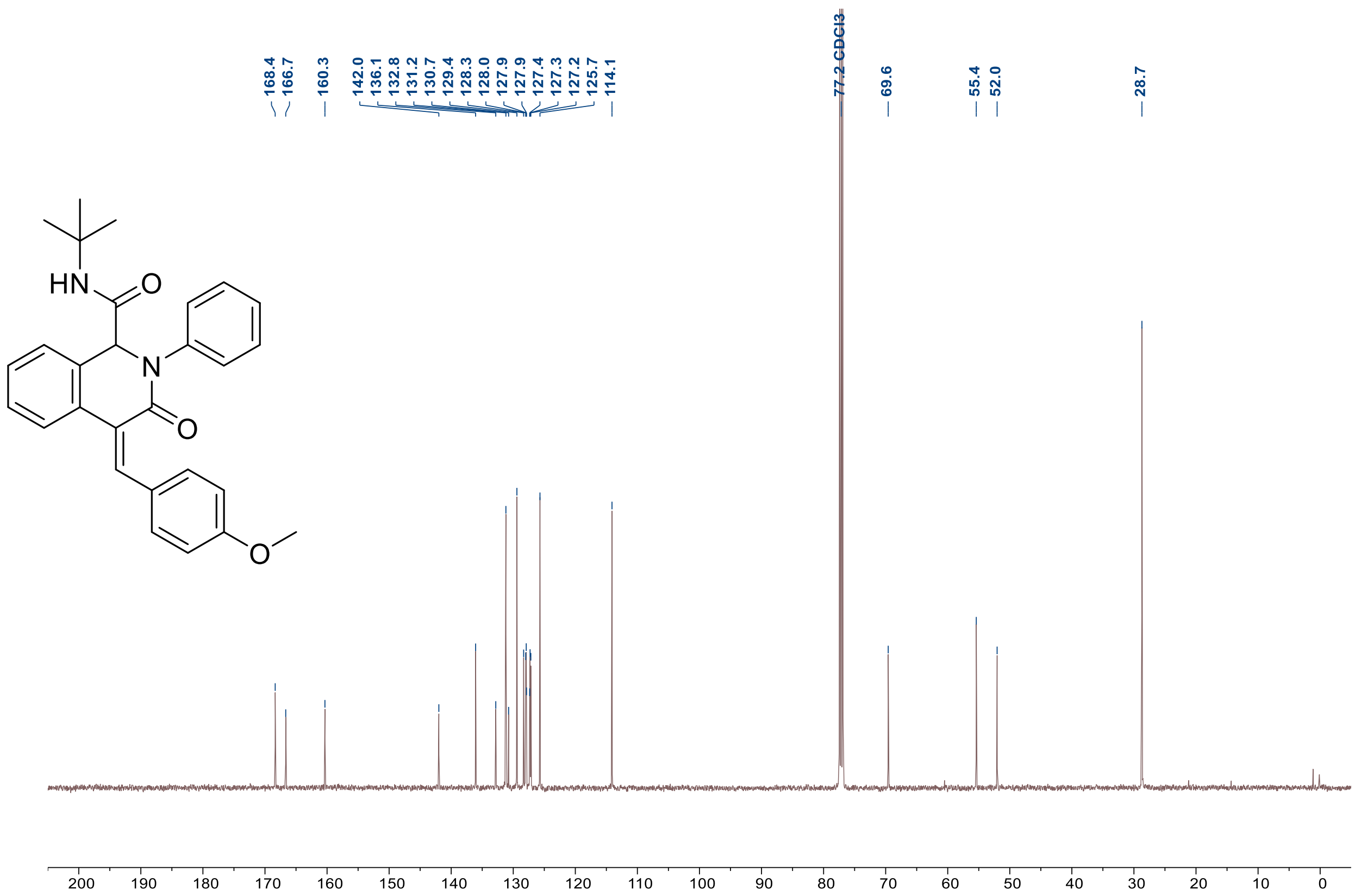

Copies of ^{1}H (500 MHz) and $^{13}C\{^{1}H\}$ (126 MHz) spectra of compound (*E*)-**28e**

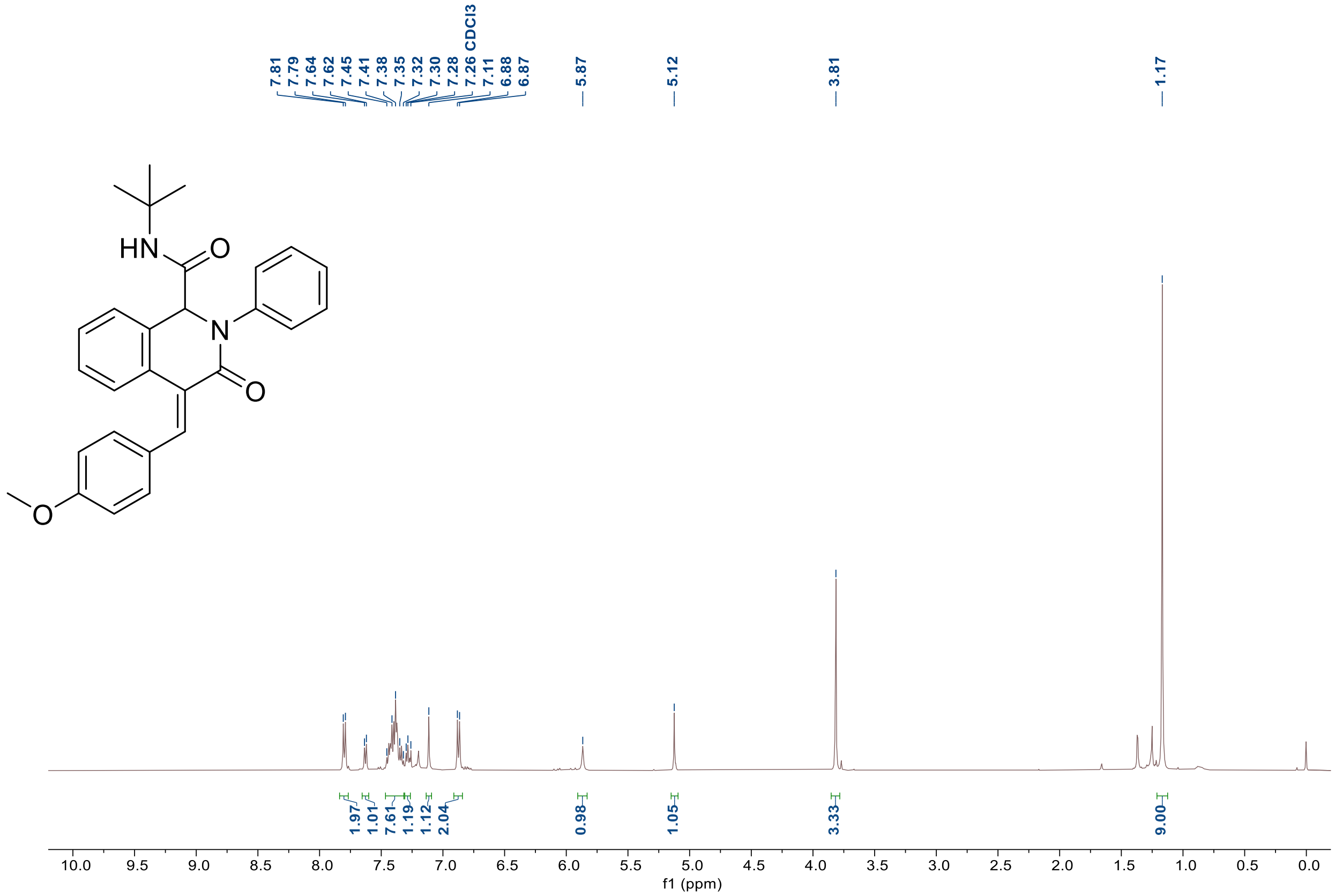

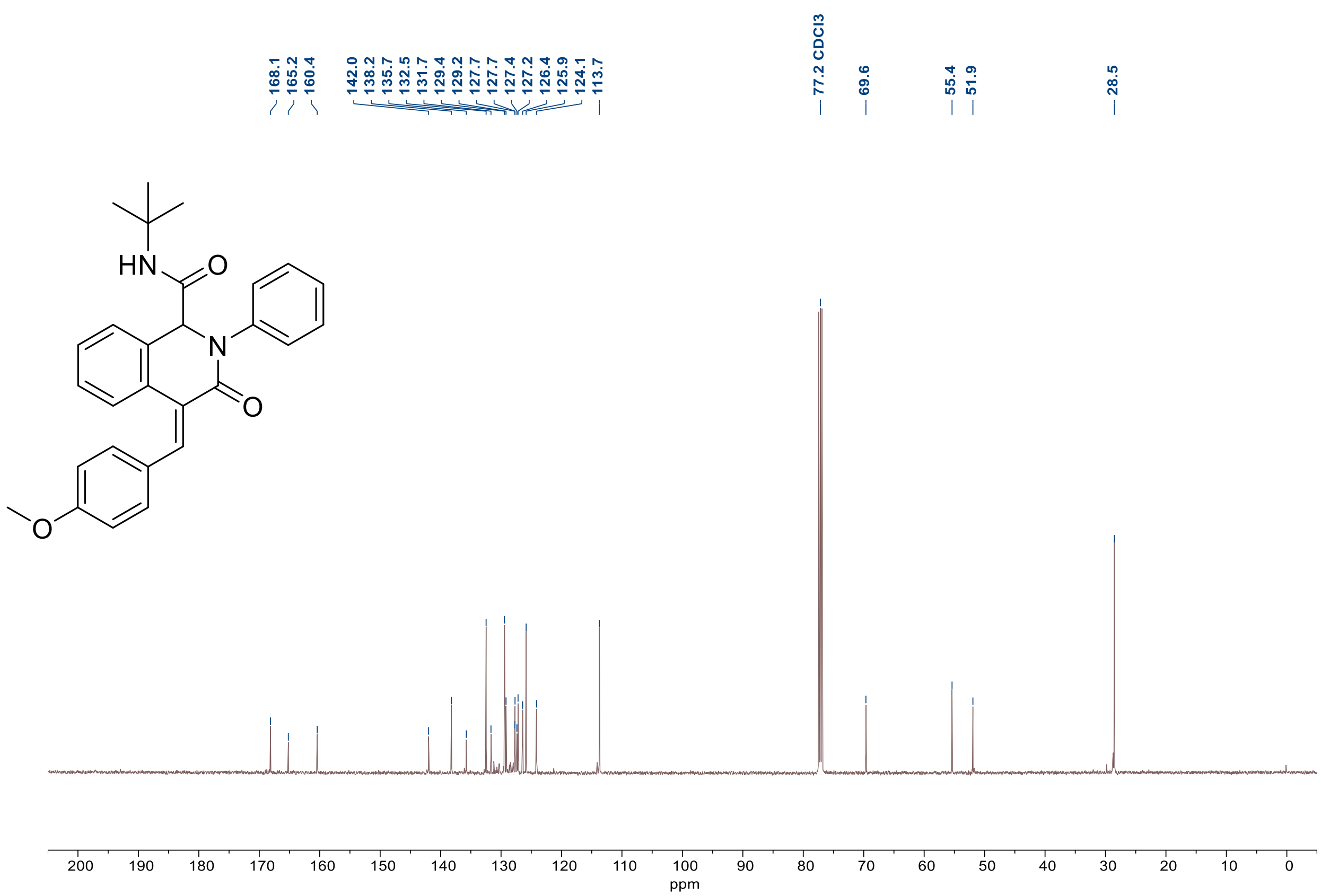

Copies of ^{1}H (500 MHz) and $^{13}C\{^{1}H\}$ (126 MHz) spectra of compound (*Z*)-**28f**

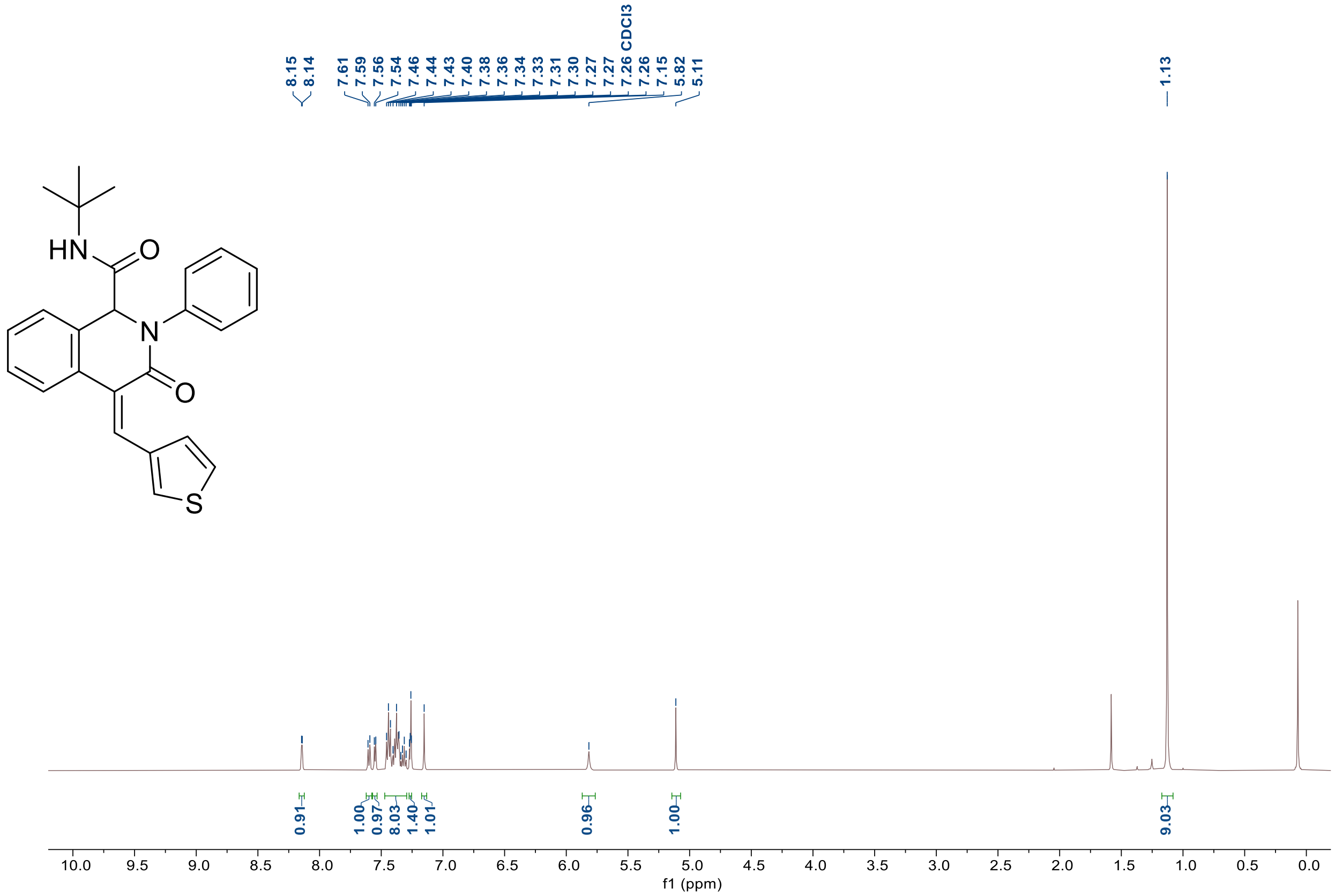

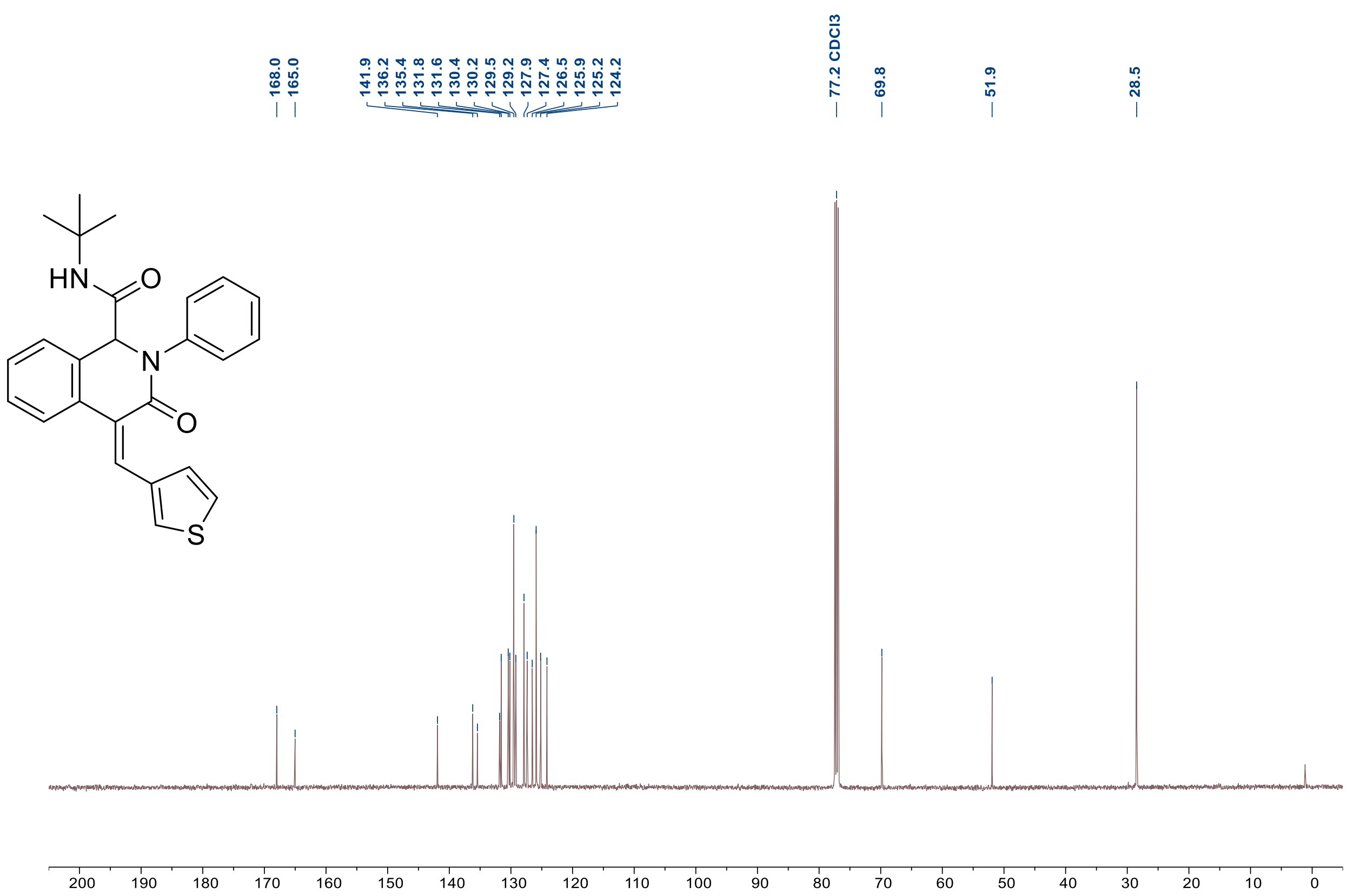

Copies of ^{1}H (500 MHz) and ^{13}C{^{1}H} (126 MHz) spectra of compound (*E*)-**28f**

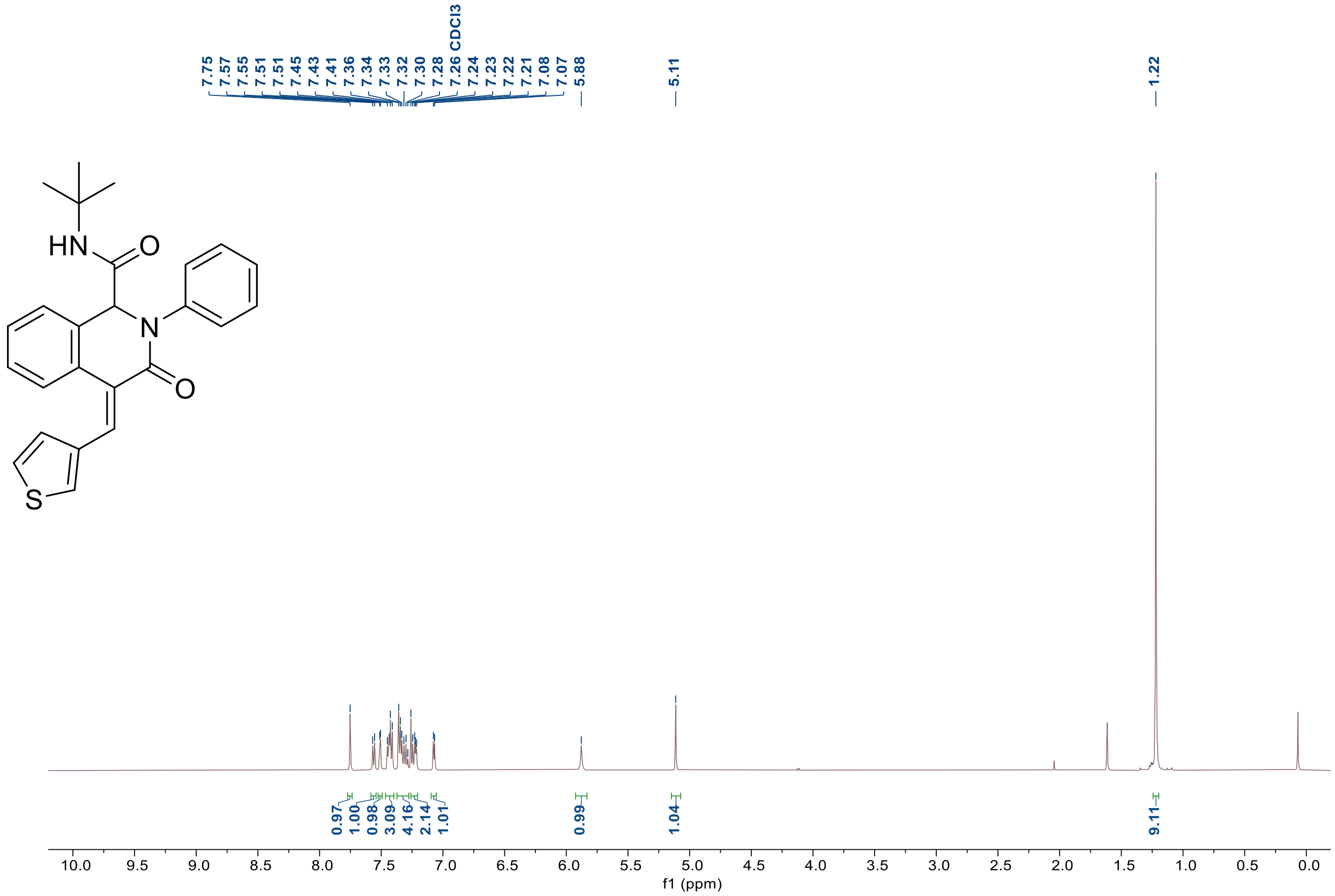

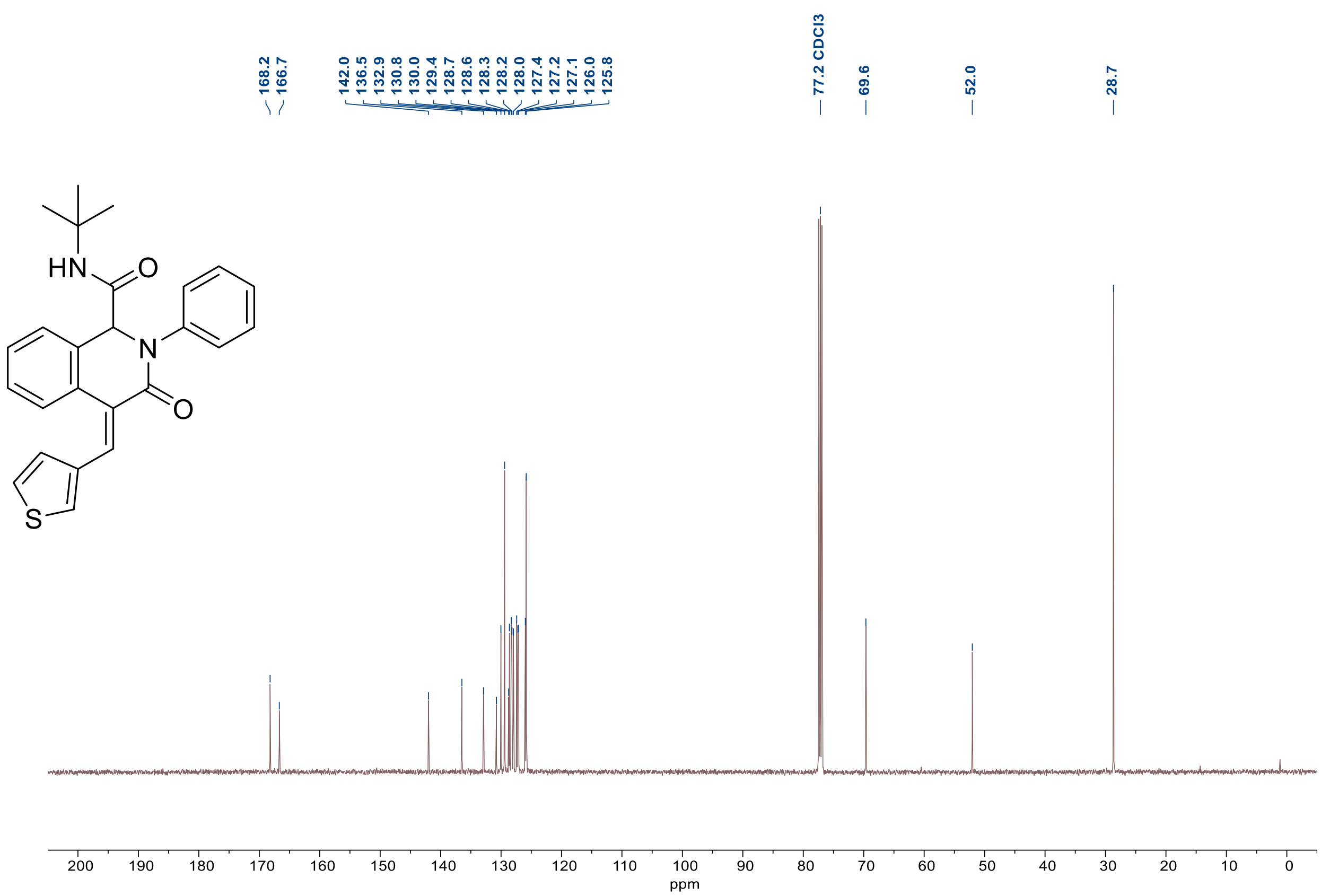

Copies of ^{1}H (500 MHz) and $^{13}C\{^{1}H\}$ (126 MHz) spectra of compound (*Z*)-**28g**

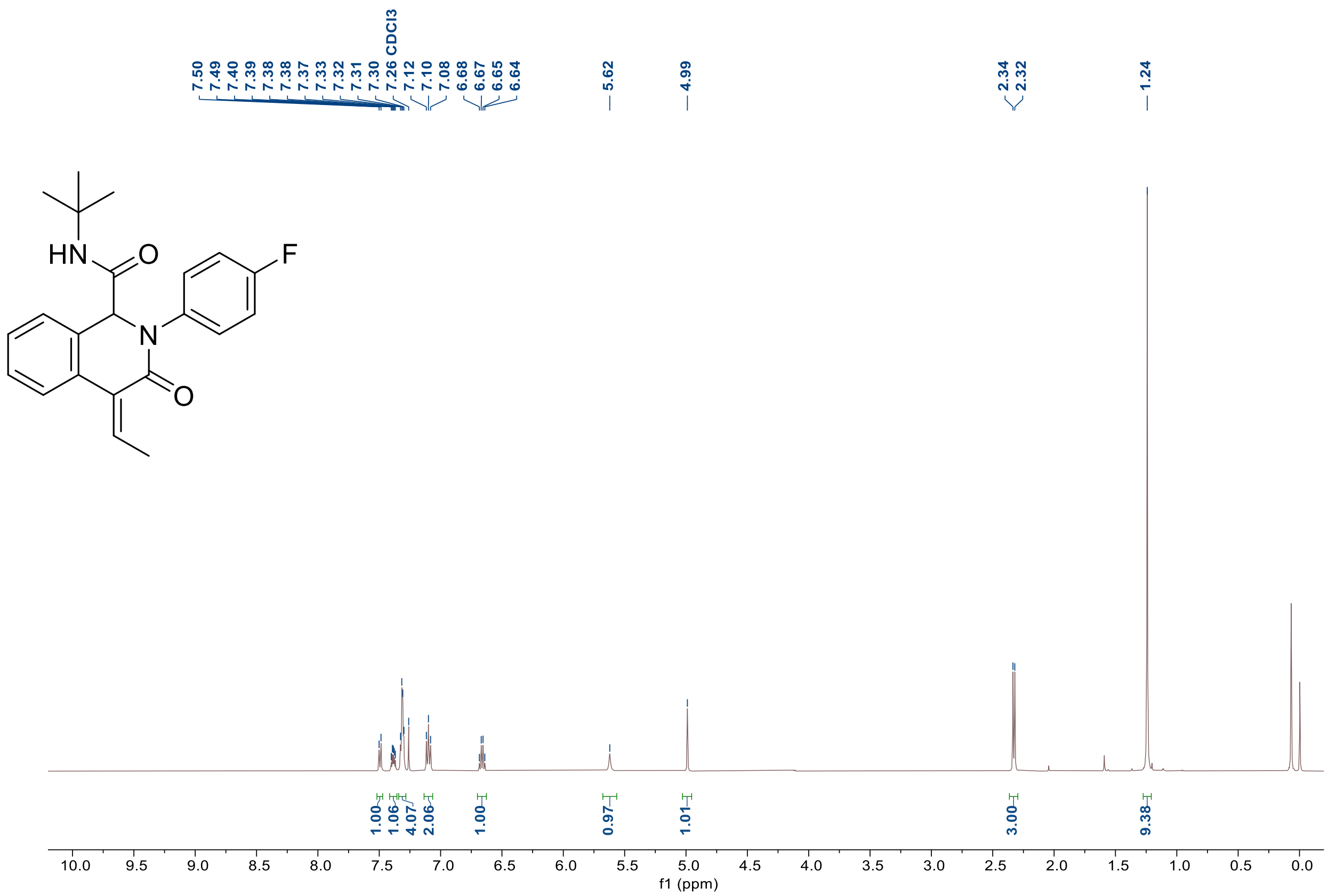

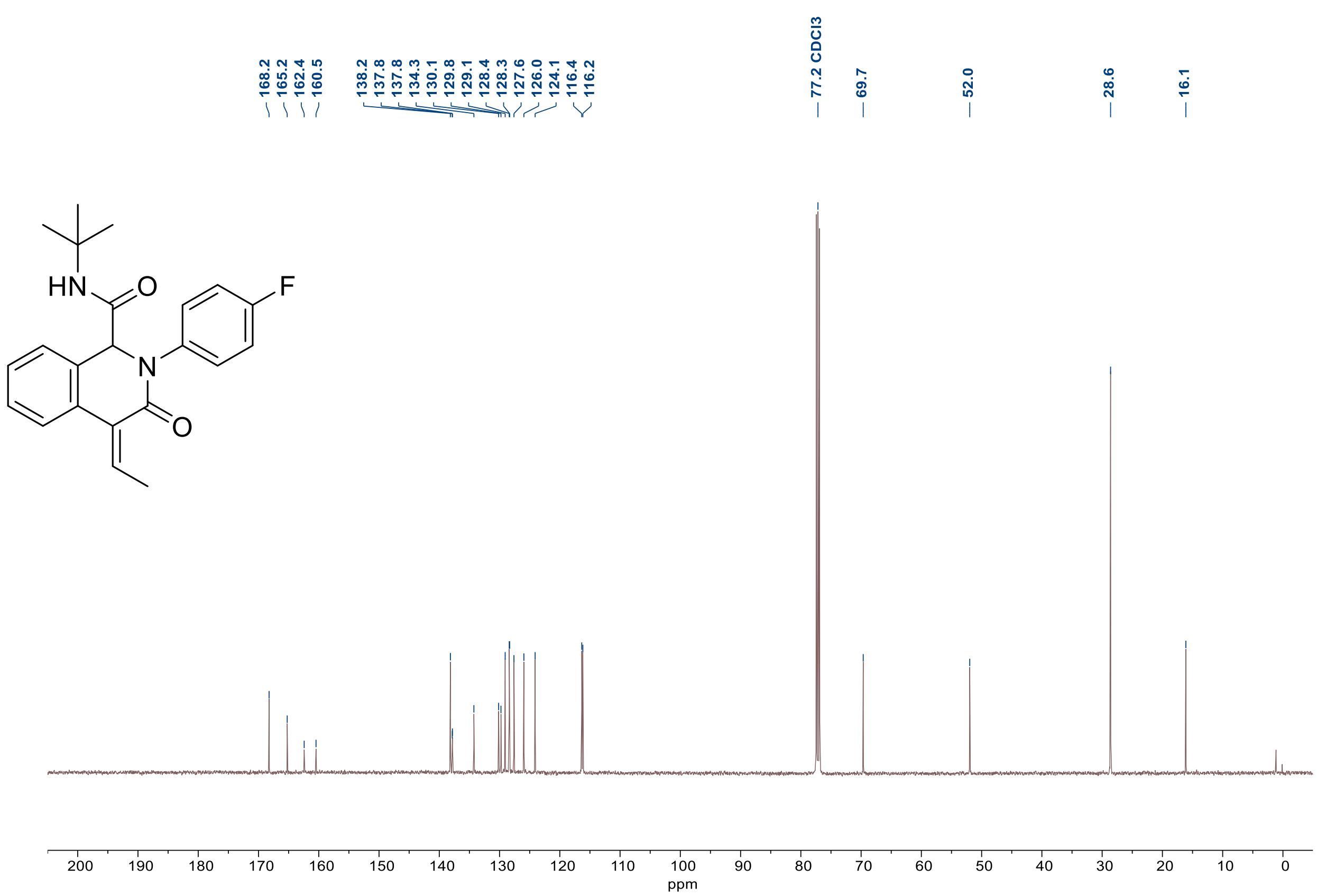

Copies of ^{1}H (500 MHz) and $^{13}C\{^{1}H\}$ (126 MHz) spectra of compound (*E*)-**28g**

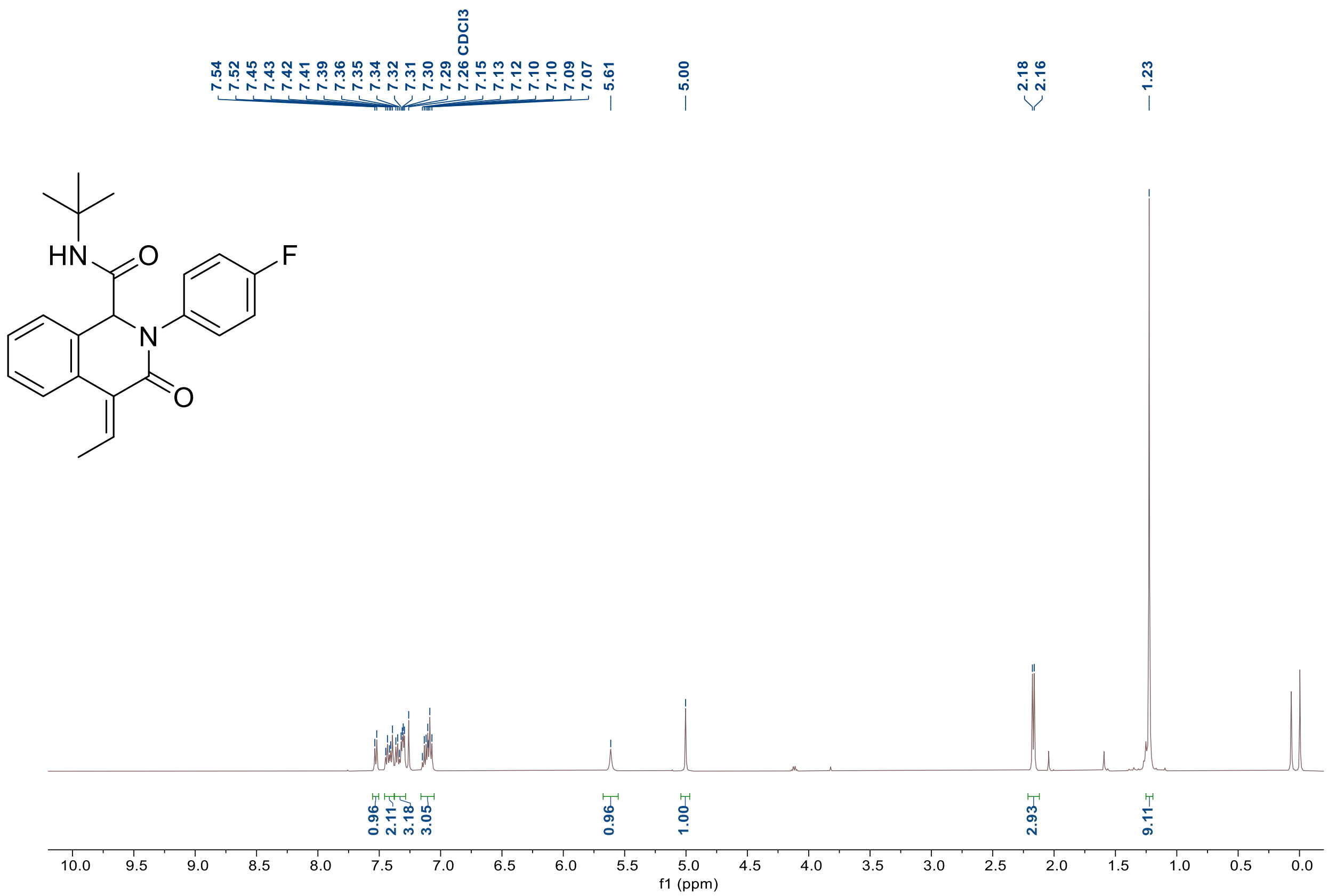

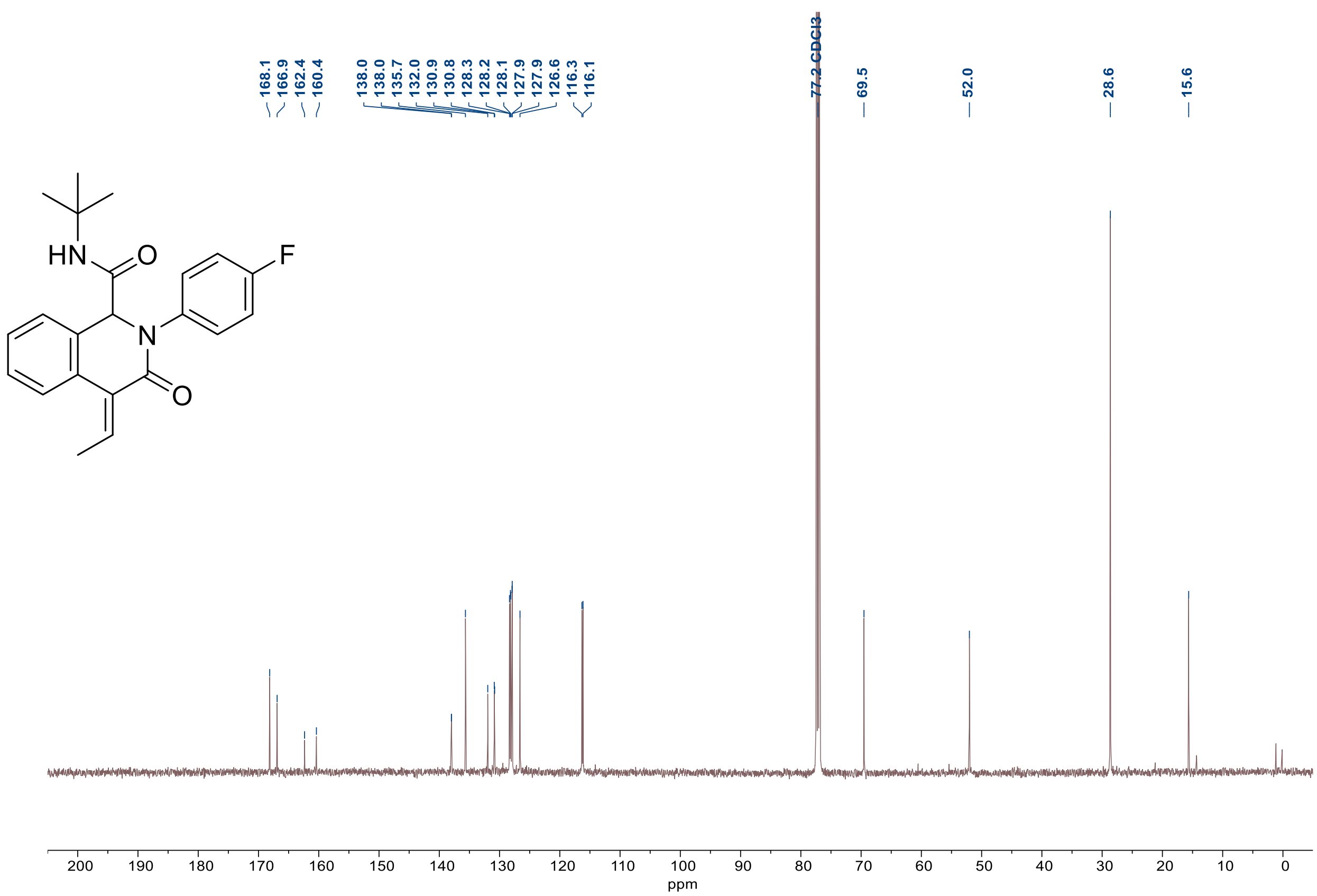

Copies of ^{1}H (400 MHz) and $^{13}C\{^{1}H\}$ (101 MHz) spectra of compound (*Z*)-**28h**

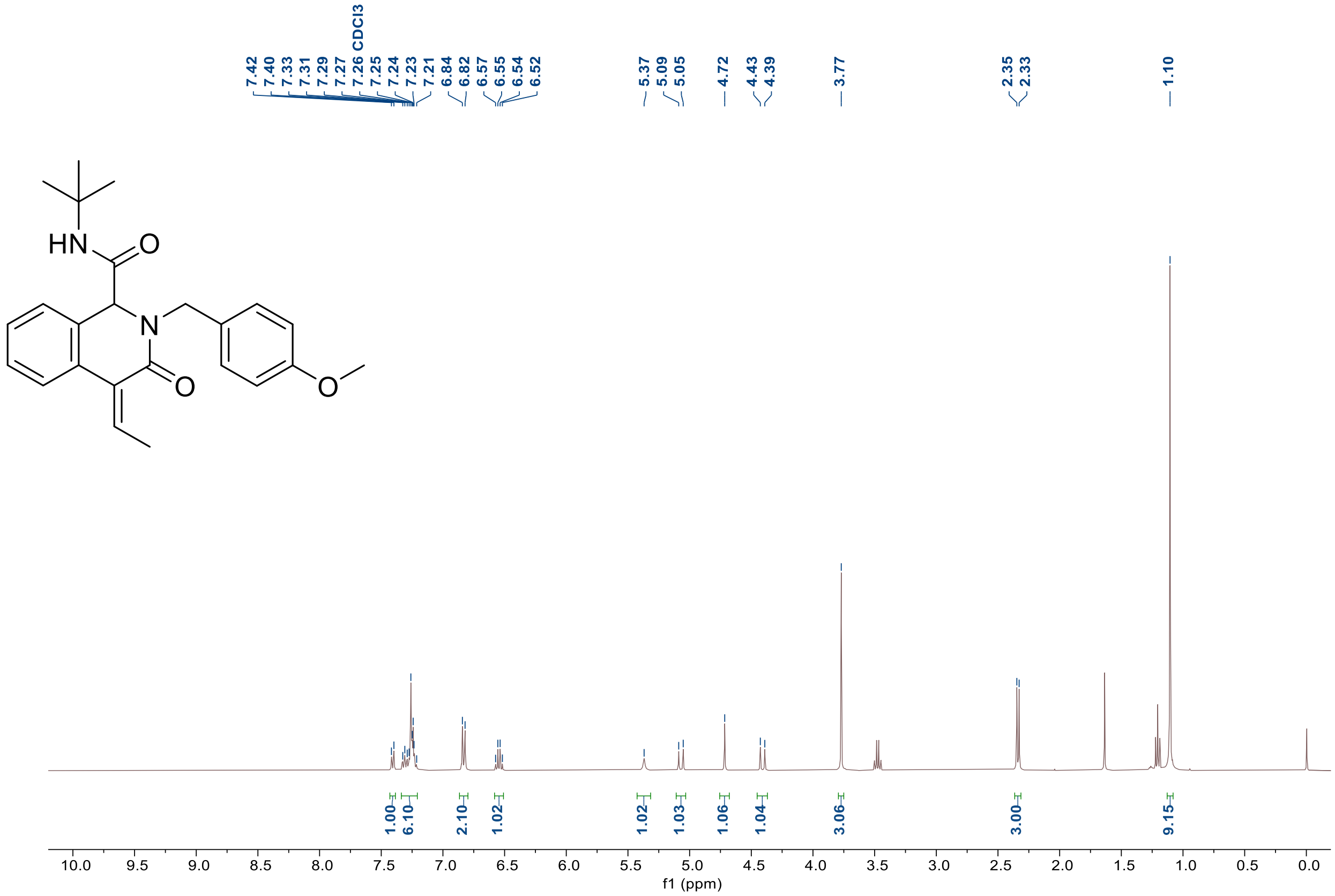

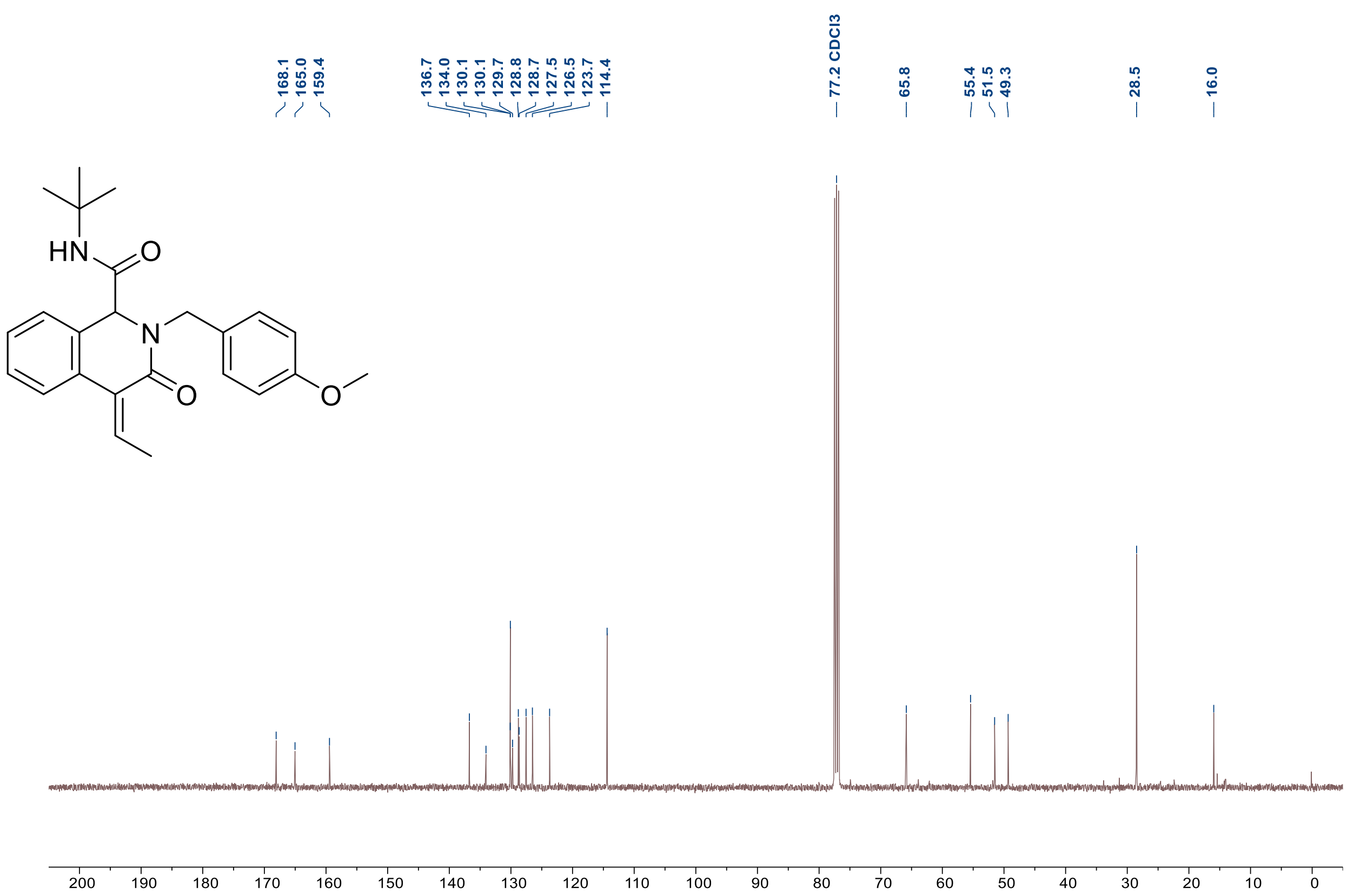

Copies of ^{1}H (500 MHz) and $^{13}C\{^{1}H\}$ (126 MHz) spectra of compound (*Z*)-**28i**

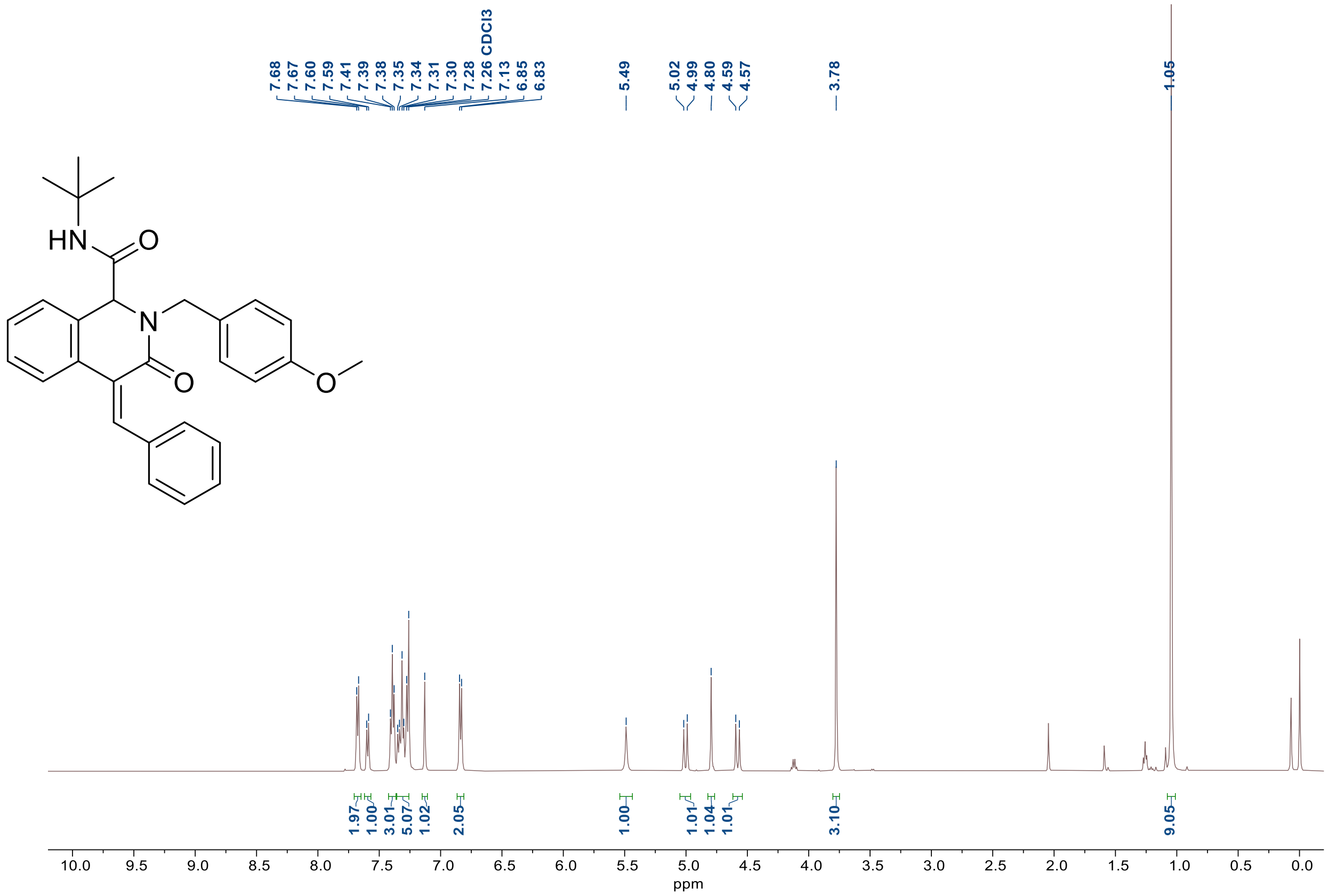

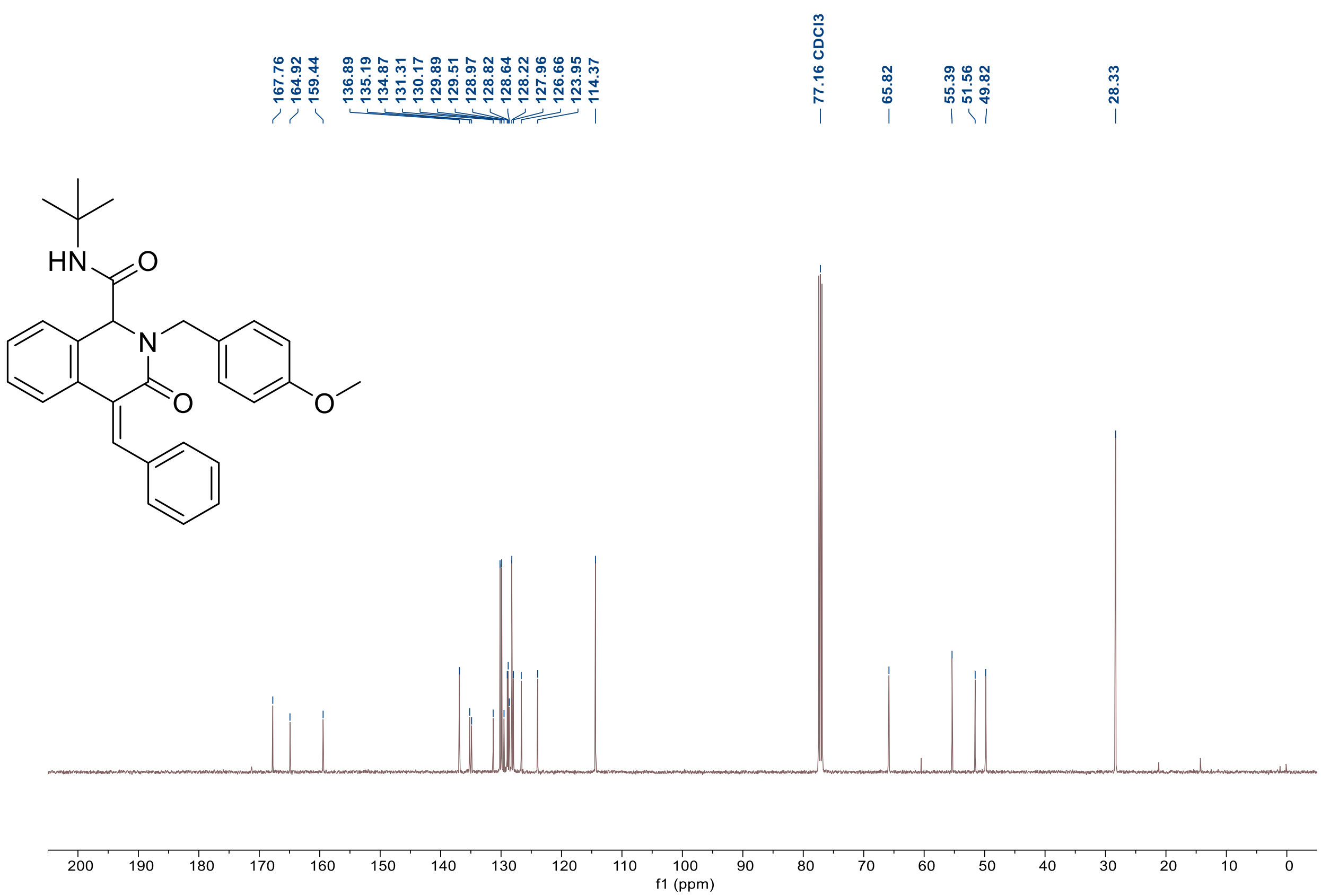

Copies of ^{1}H (400 MHz) and $^{13}C\{^{1}H\}$ (151 MHz) spectra of compound (*Z*)-**28j**

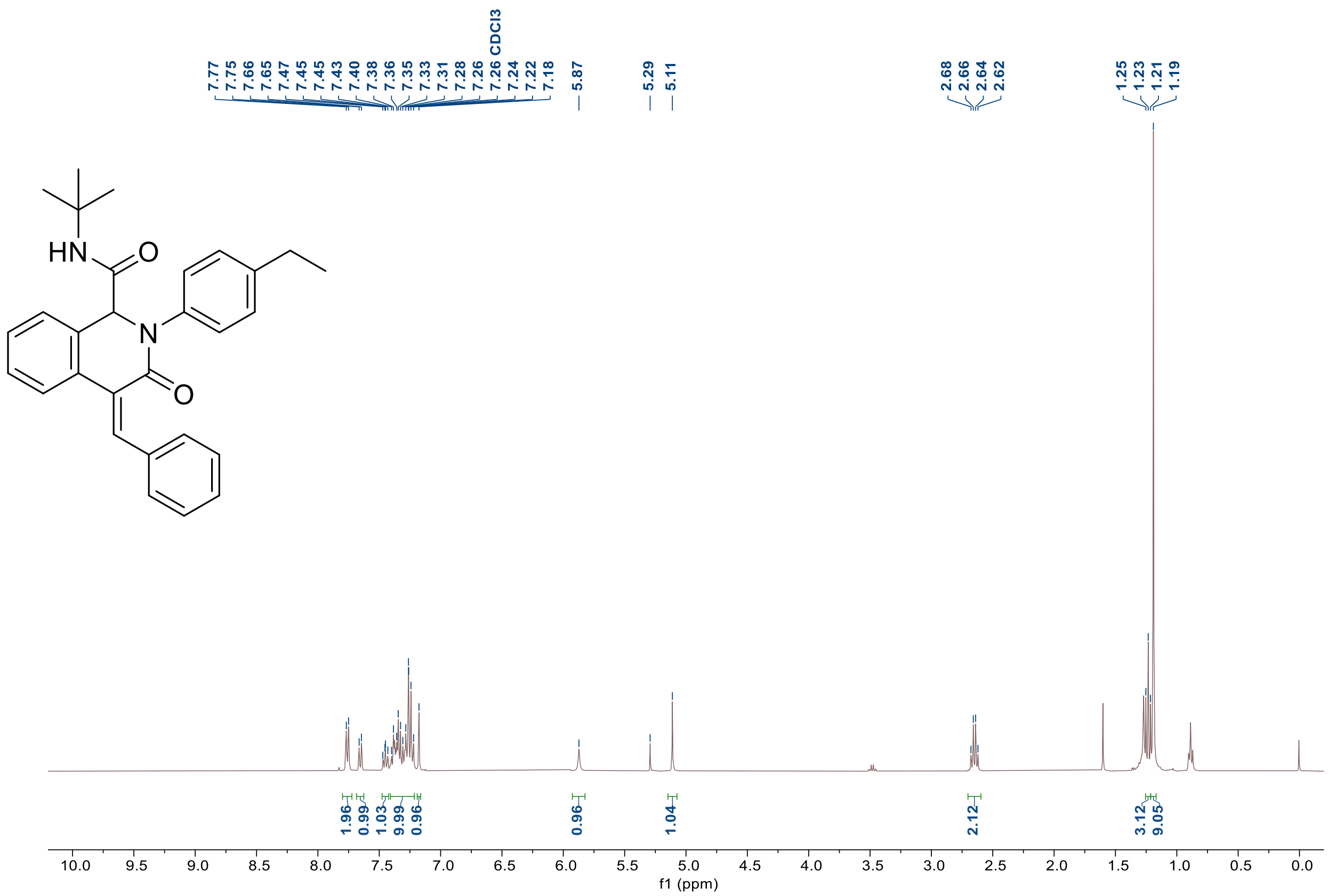

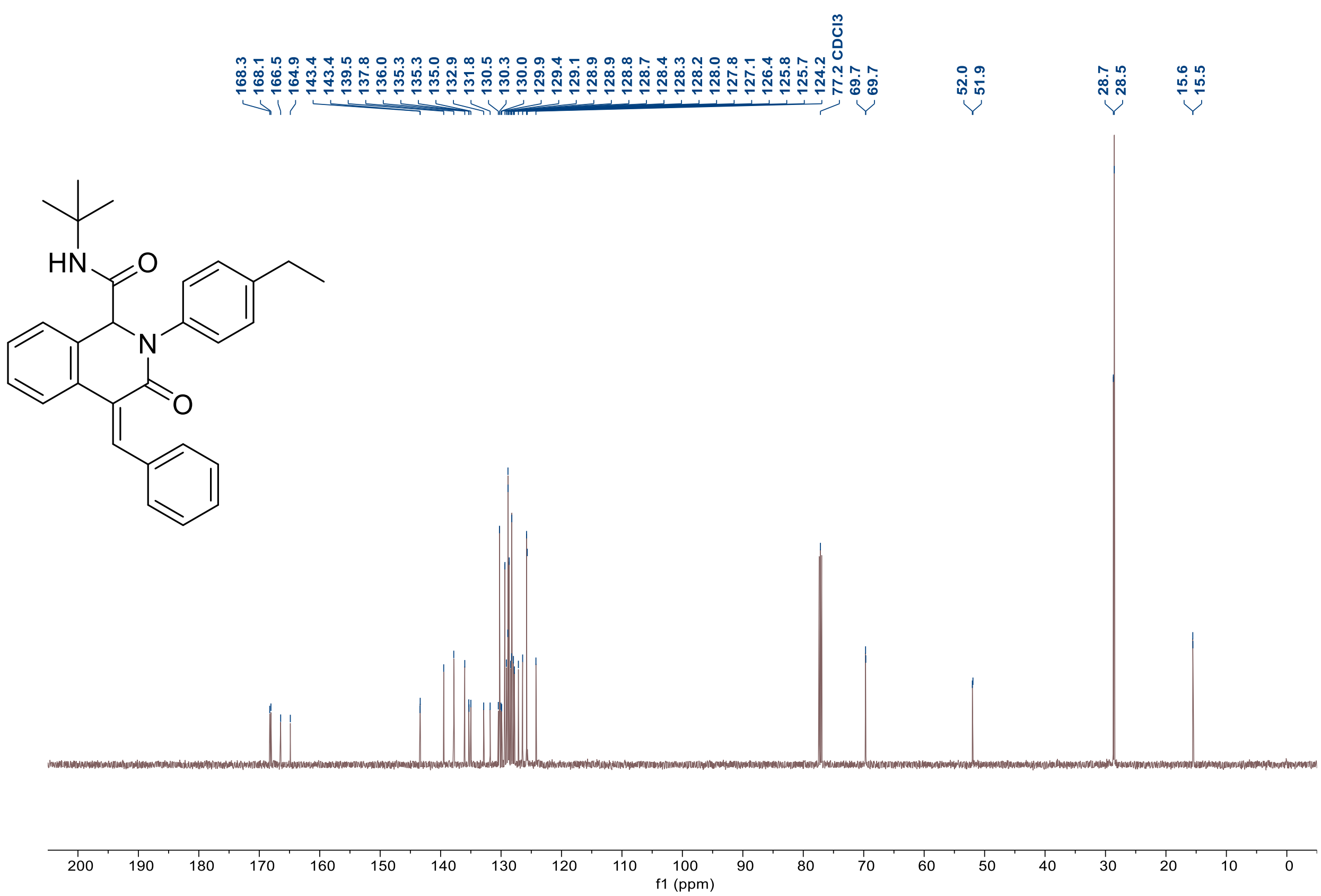

Copies of ^{1}H (400 MHz) and $^{13}C\{^{1}H\}$ (101 MHz) spectra of compound (*Z*)-**28k**

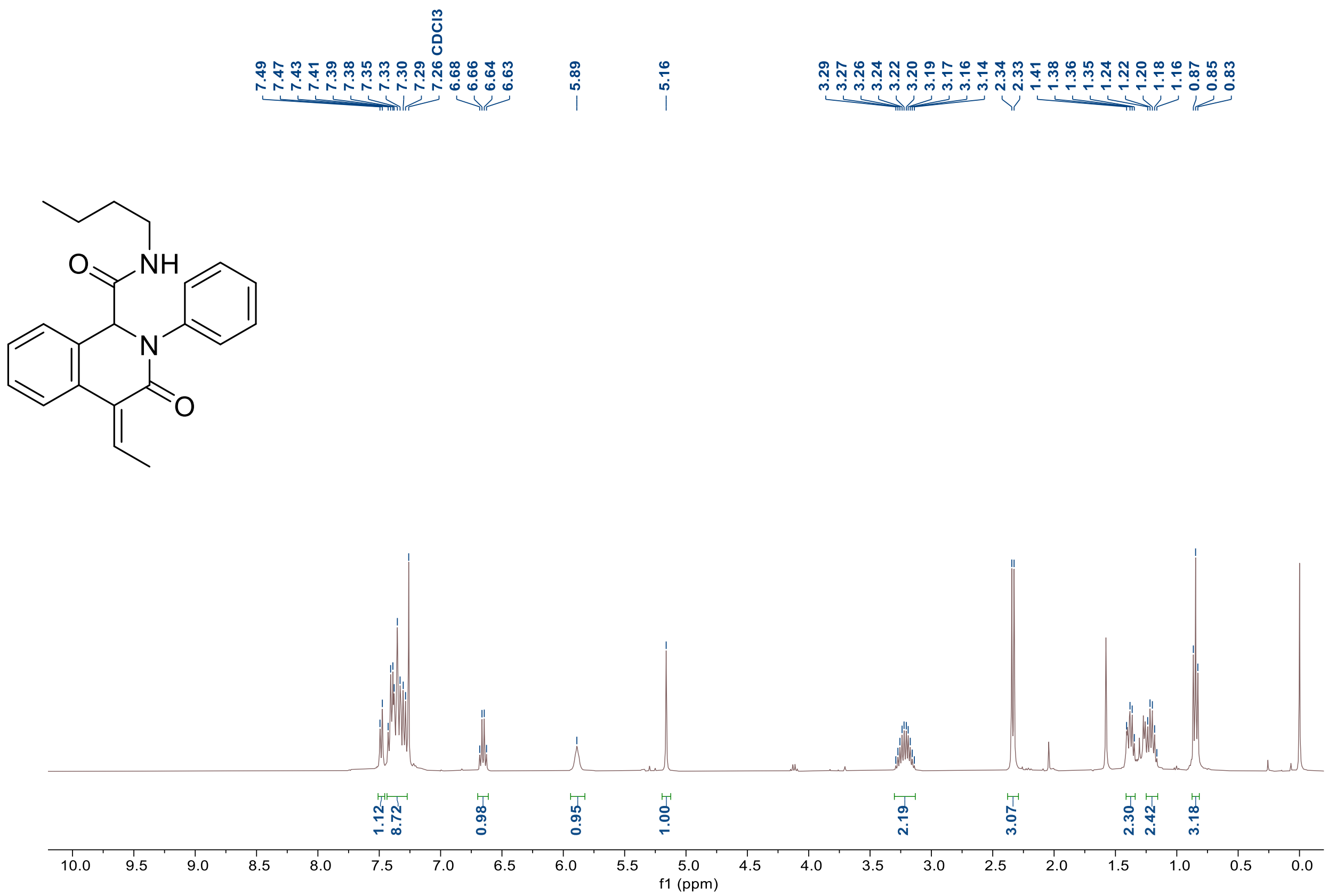

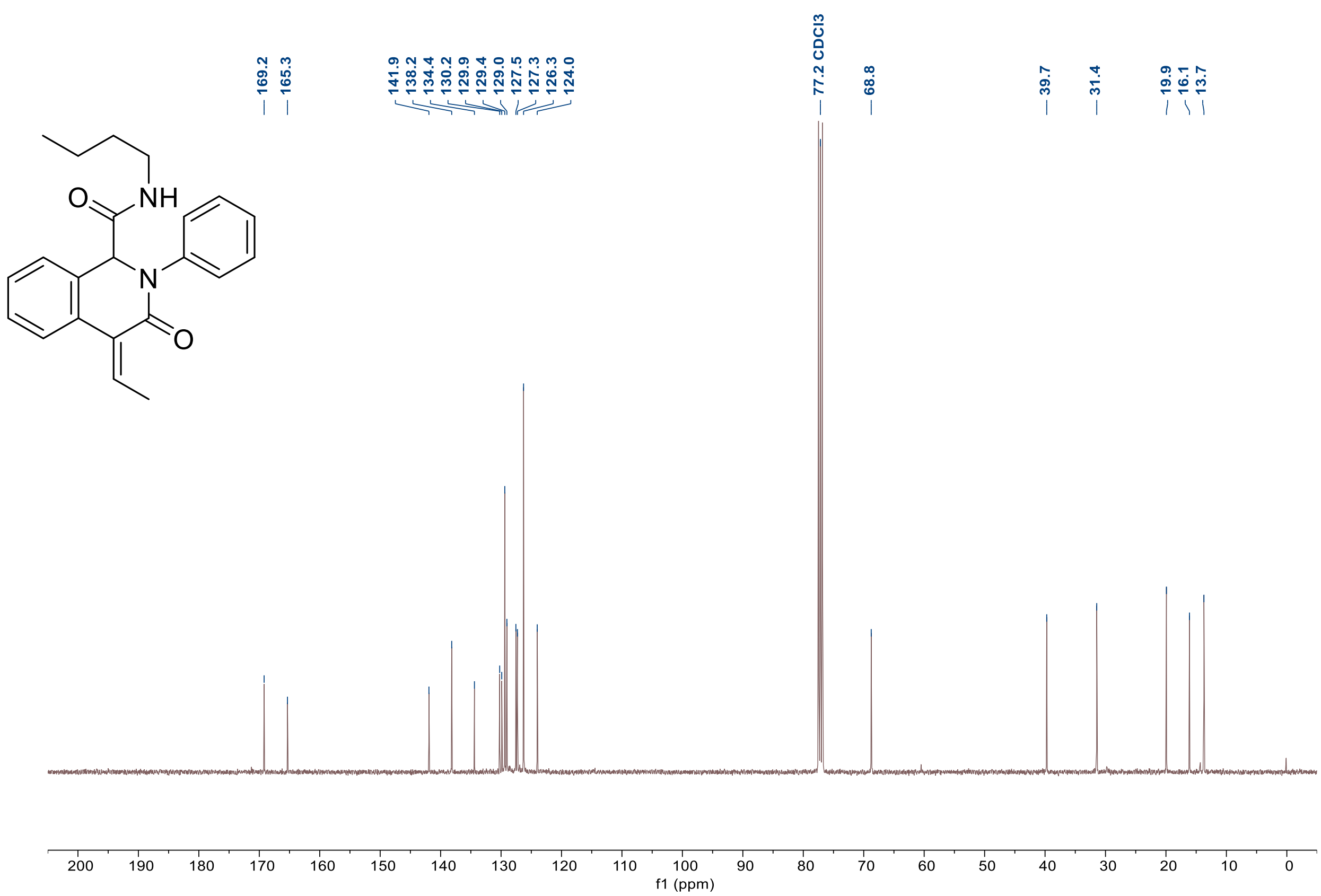

Copies of ^{1}H (500 MHz) and $^{13}C\{^{1}H\}$ (126 MHz) spectra of Z/E mixture of **28l**, but only the signals from the major *Z* isomer are marked and integrated

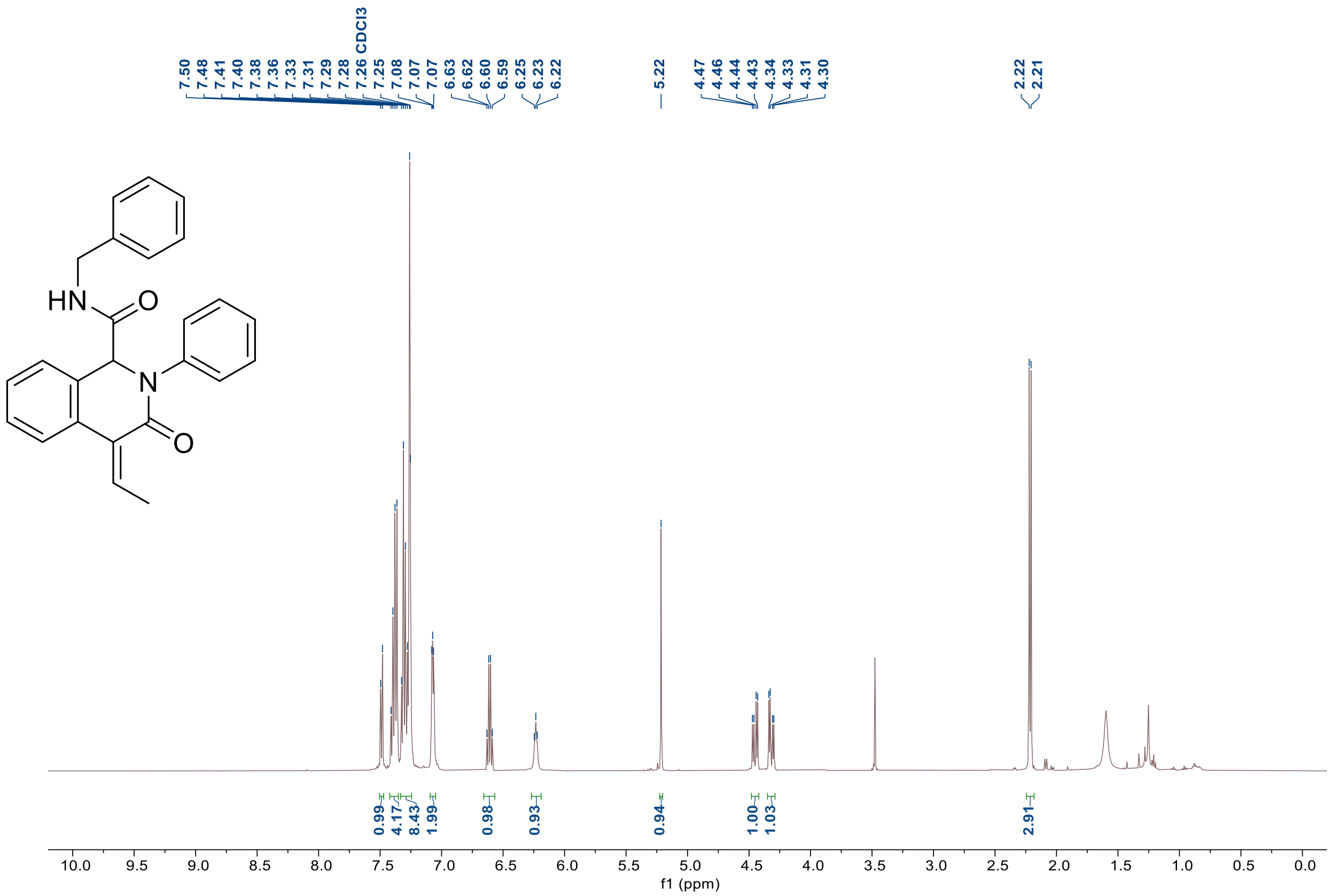

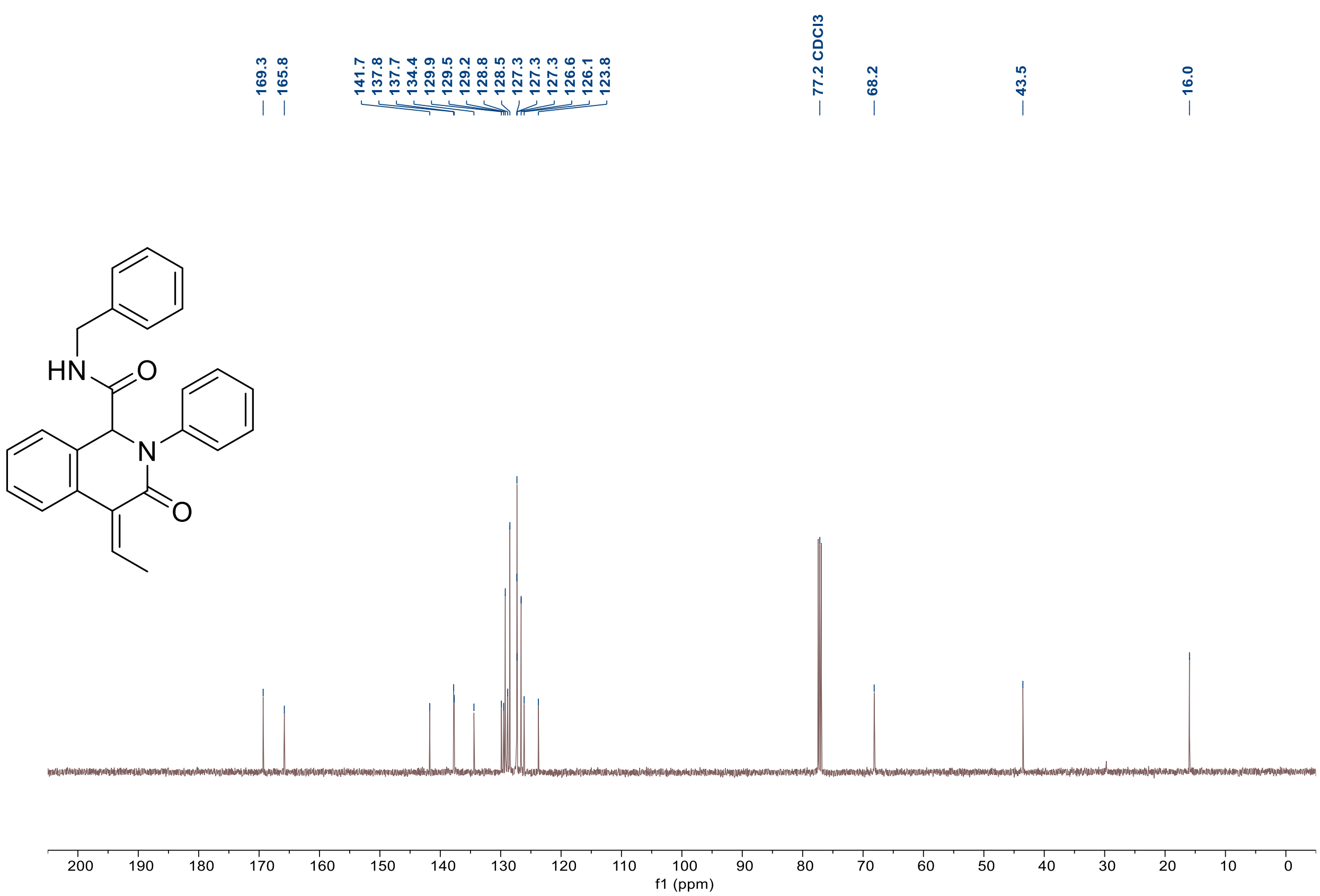

Copies of ^{1}H (500 MHz) and $^{13}C\{^{1}H\}$ (126 MHz) spectra of compound *cis*-**29a**

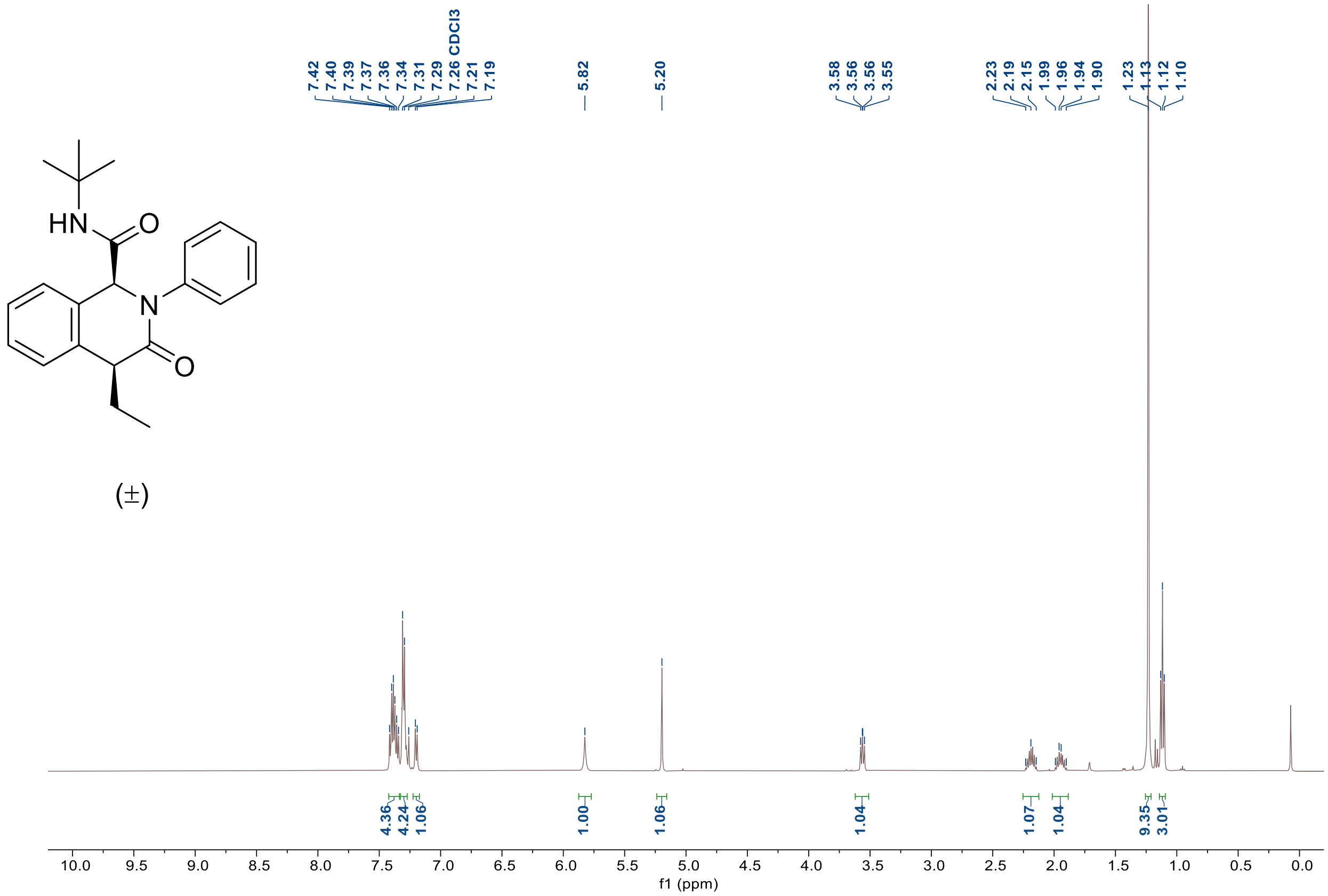

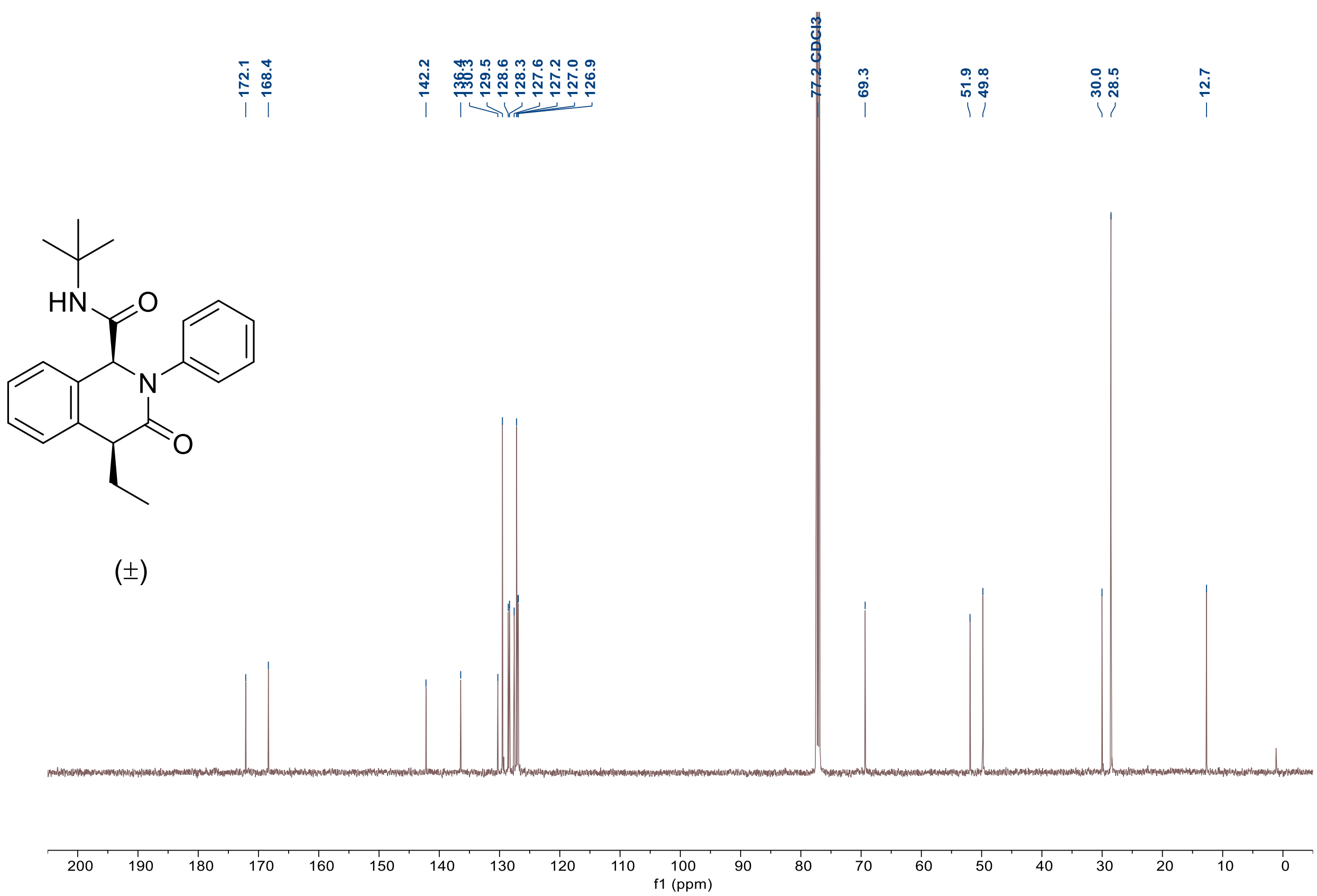

Copies of ^{1}H (500 MHz) and ^{13}C{^{1}H} (126 MHz) spectra of compound *cis*-**29b**

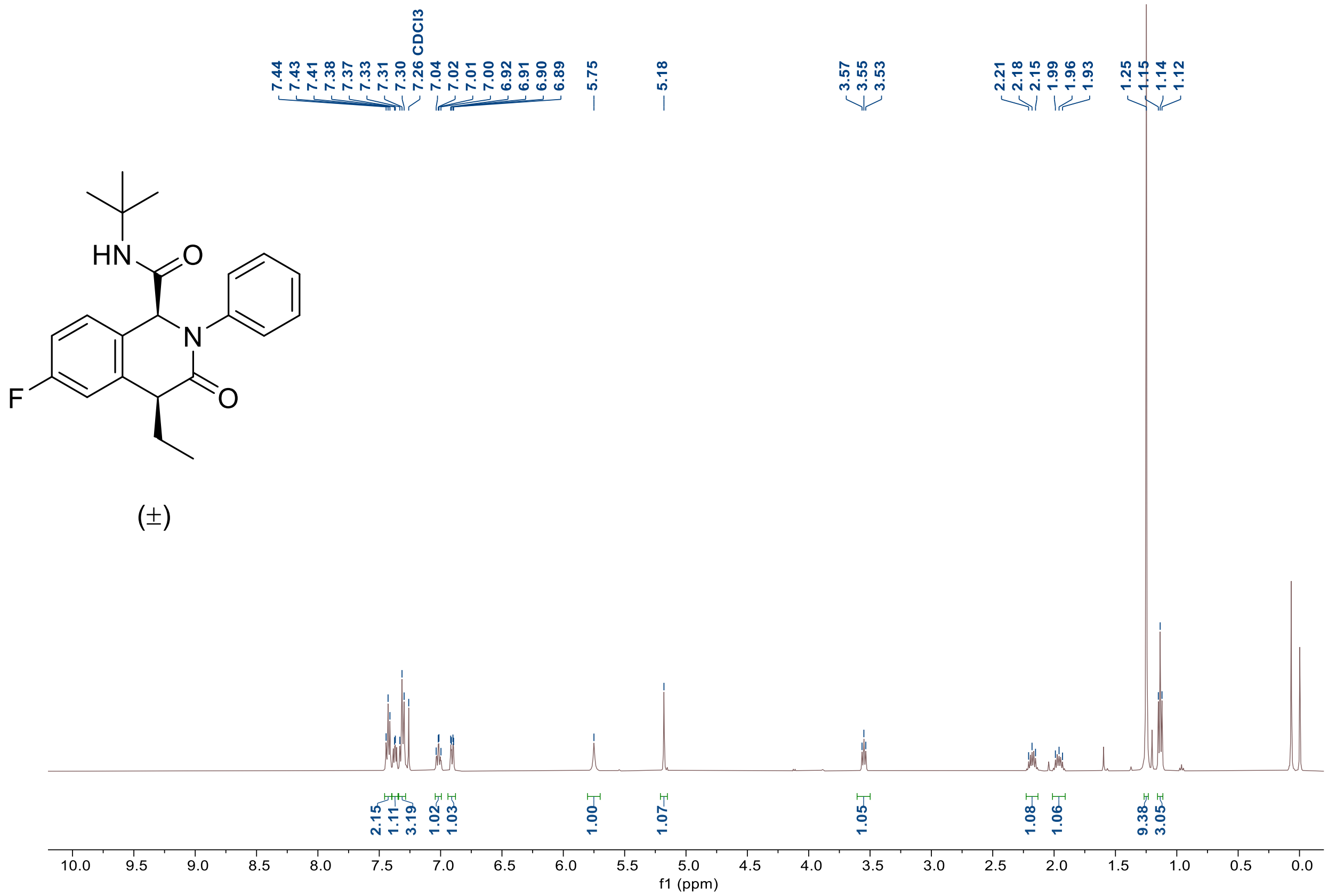

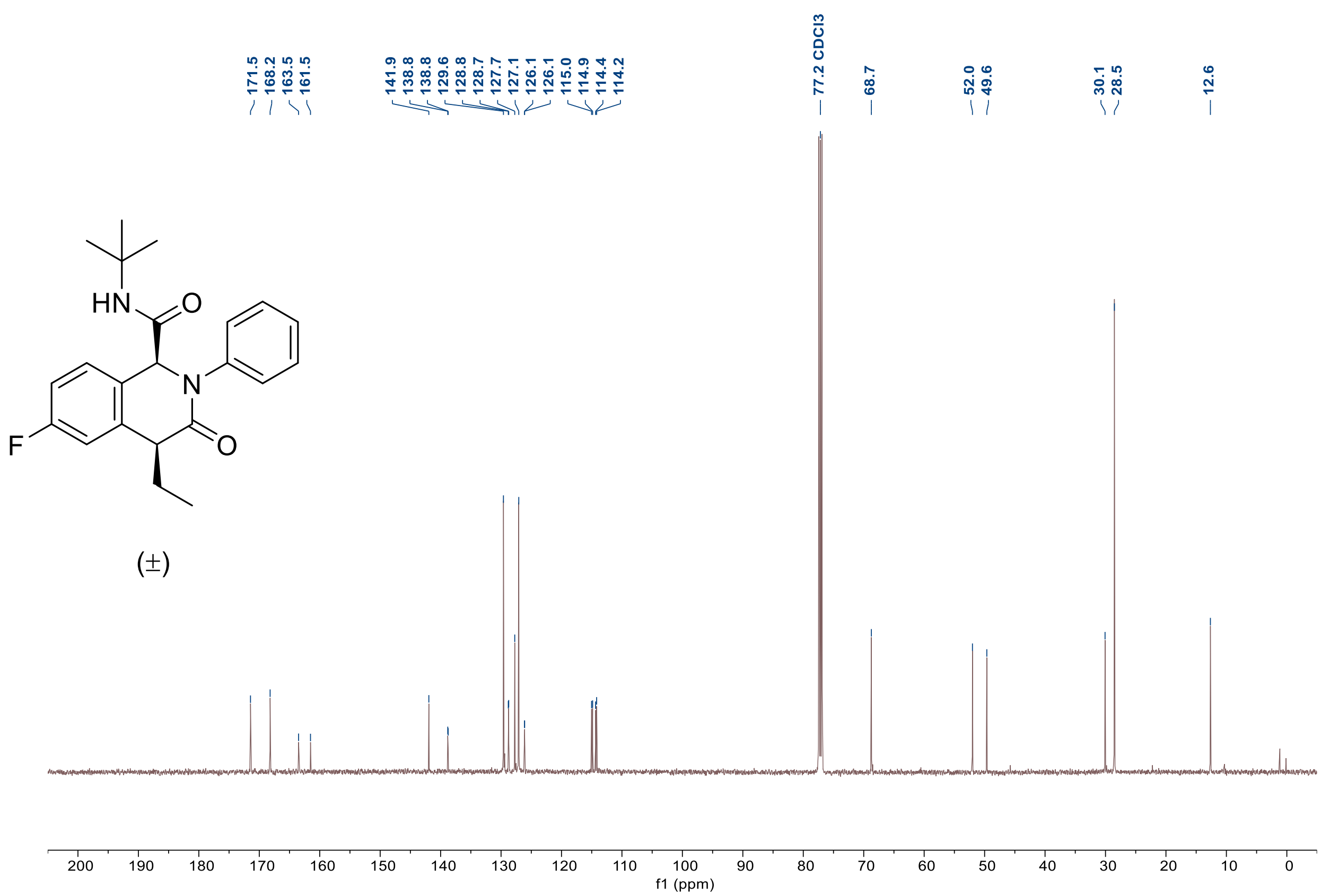

Copies of ^{1}H (400 MHz) and ^{13}C{^{1}H} (101 MHz) spectra of compound *cis*-**29c**

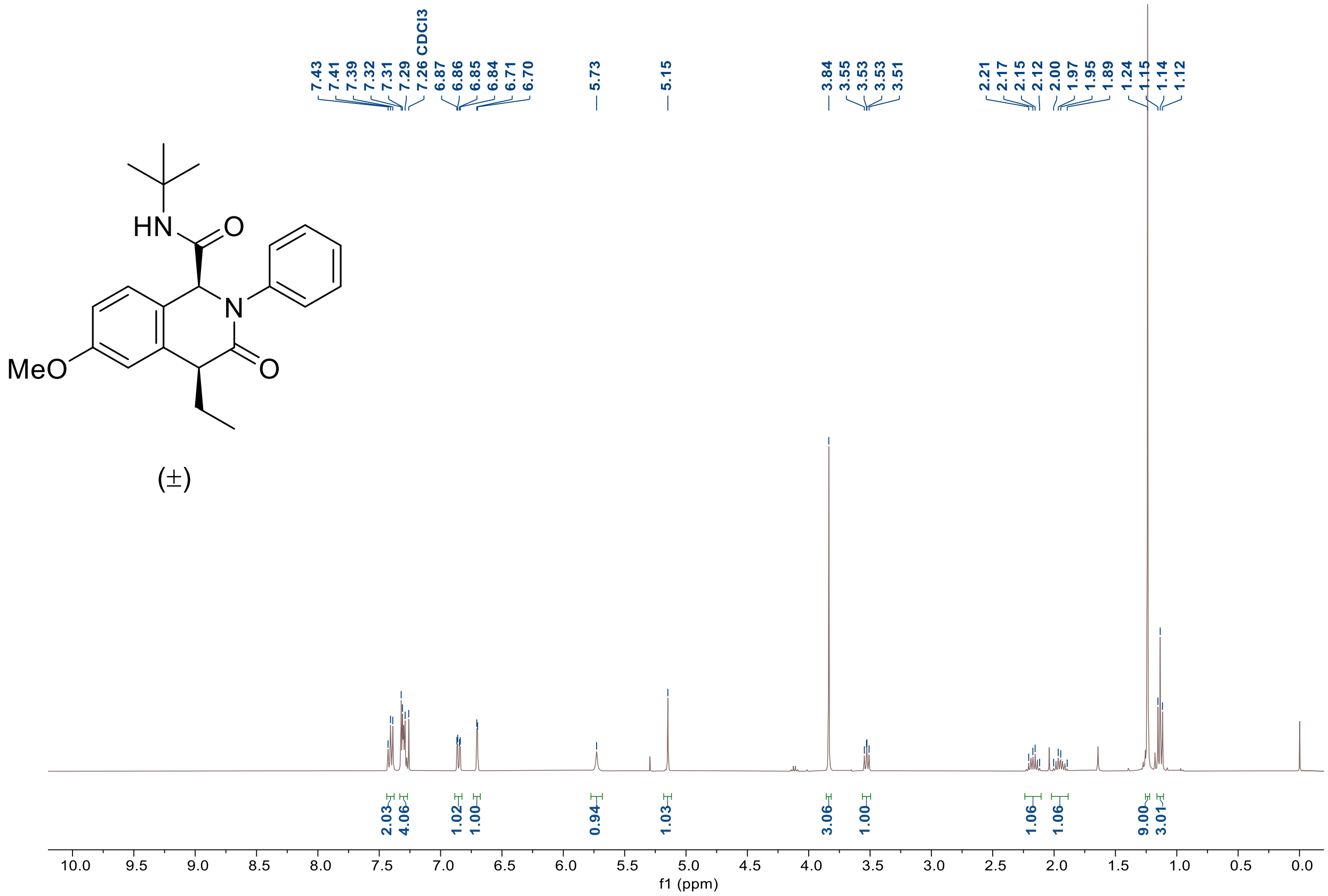

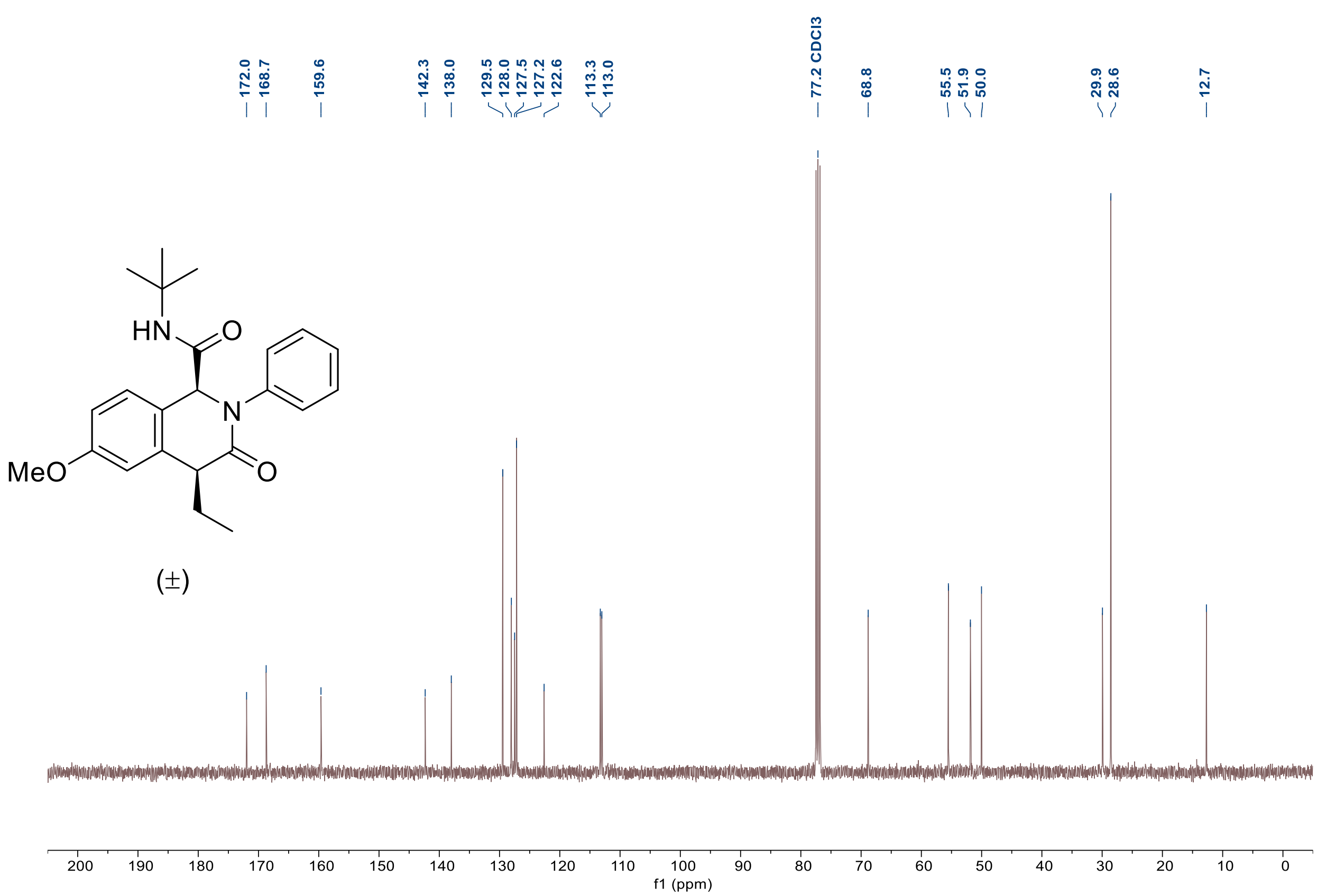

Copies of ^{1}H (500 MHz) and ^{13}C{^{1}H} (126 MHz) spectra of compound *cis*-**29d**

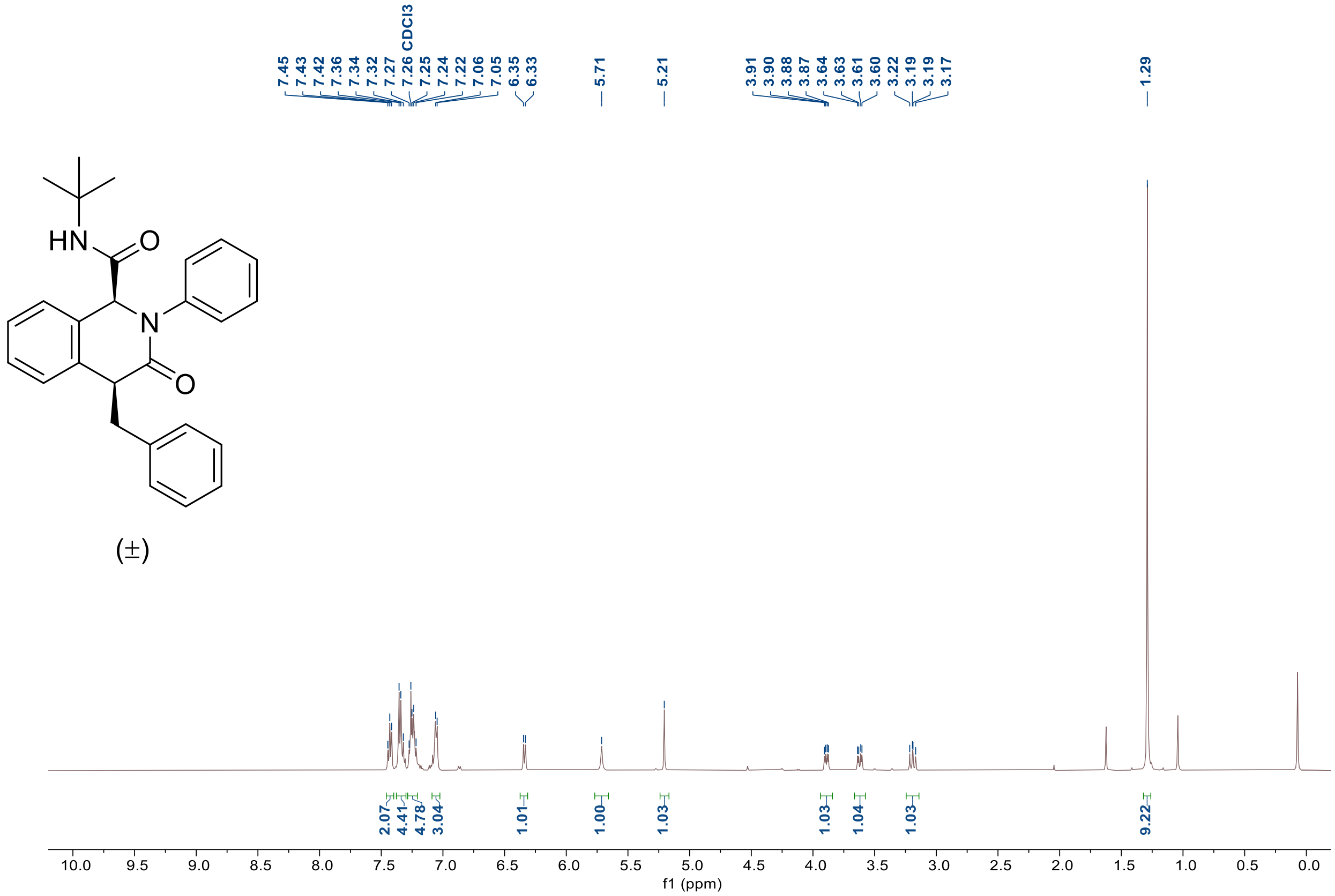

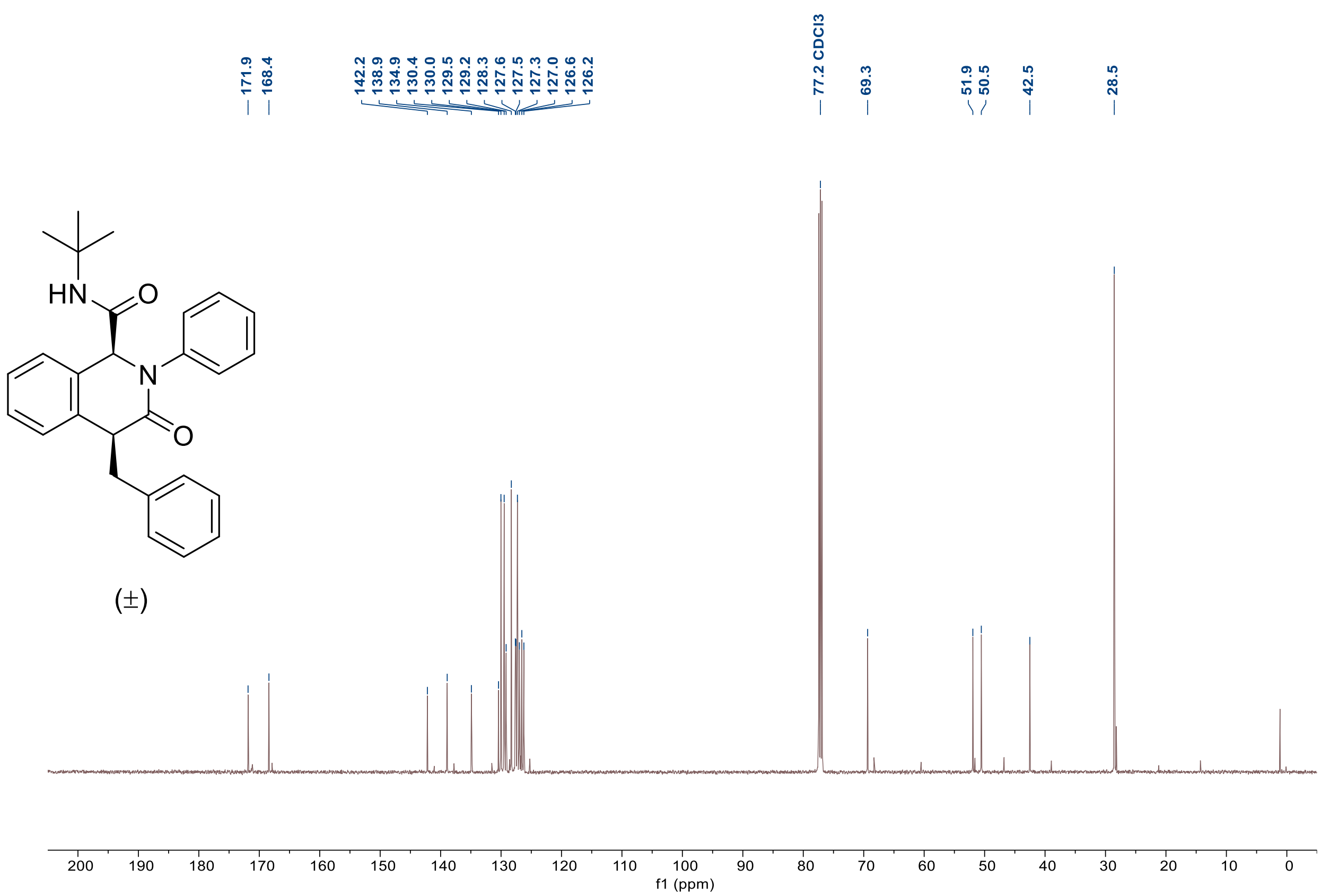

Copies of ^{1}H (500 MHz) and $^{13}C\{^{1}H\}$ (126 MHz) spectra of compound *cis*-**29e**

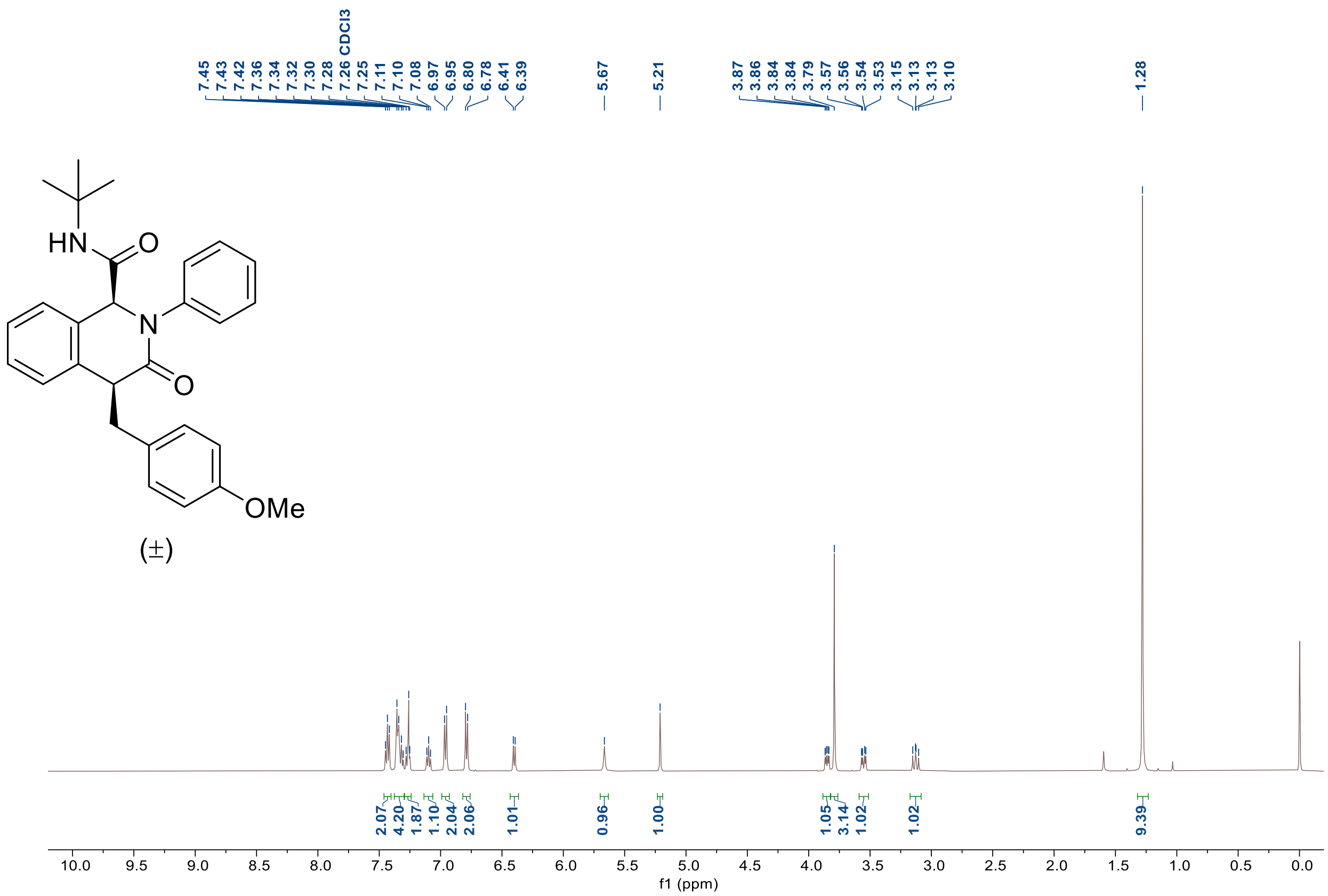

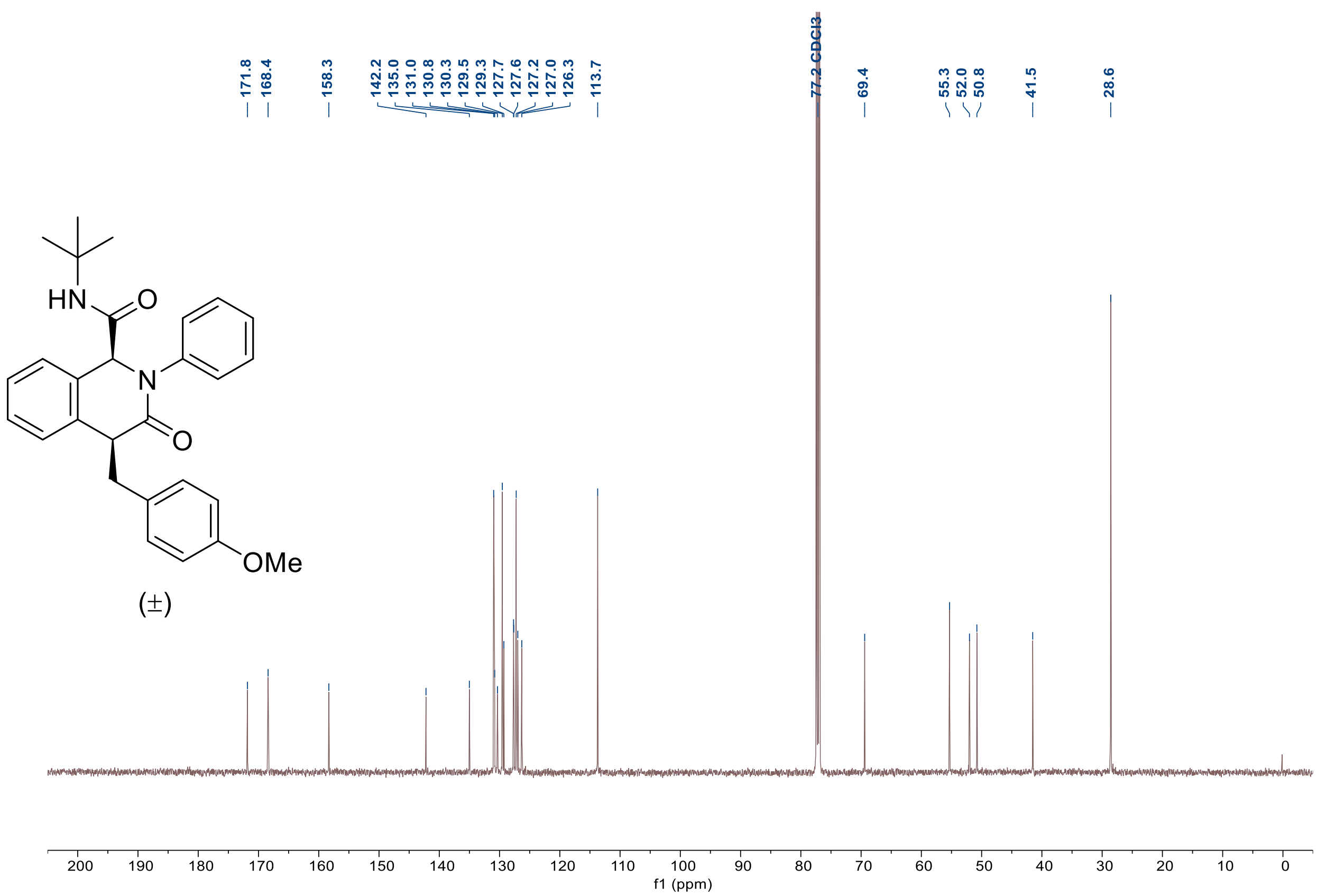

Copies of ^{1}H (400 MHz) and $^{13}C\{^{1}H\}$ (101 MHz) spectra of compound *cis*-**29f**

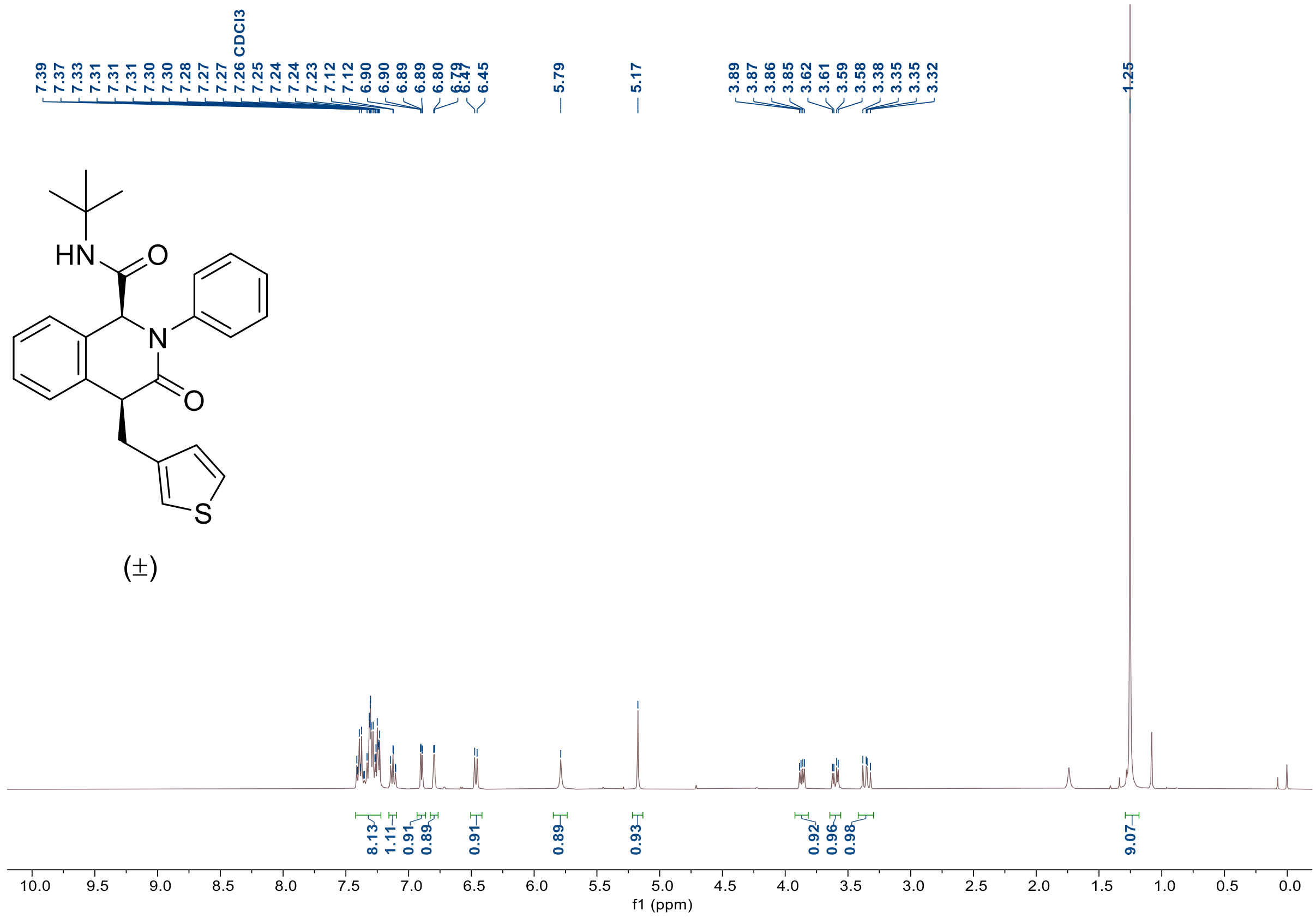

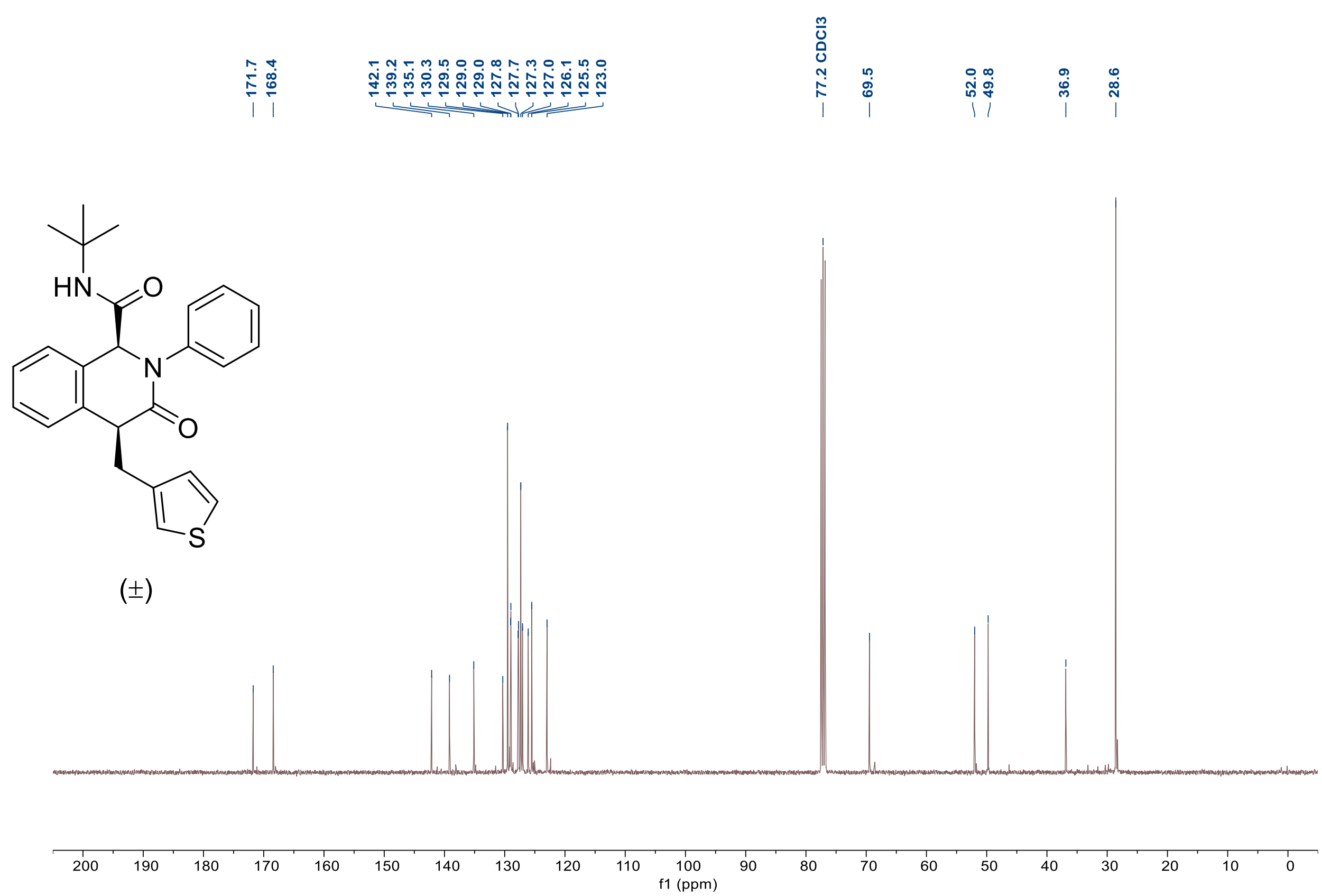

Copies of ^{1}H (500 MHz) and $^{13}C\{^{1}H\}$ (126 MHz) spectra of compound *cis*-**29g**

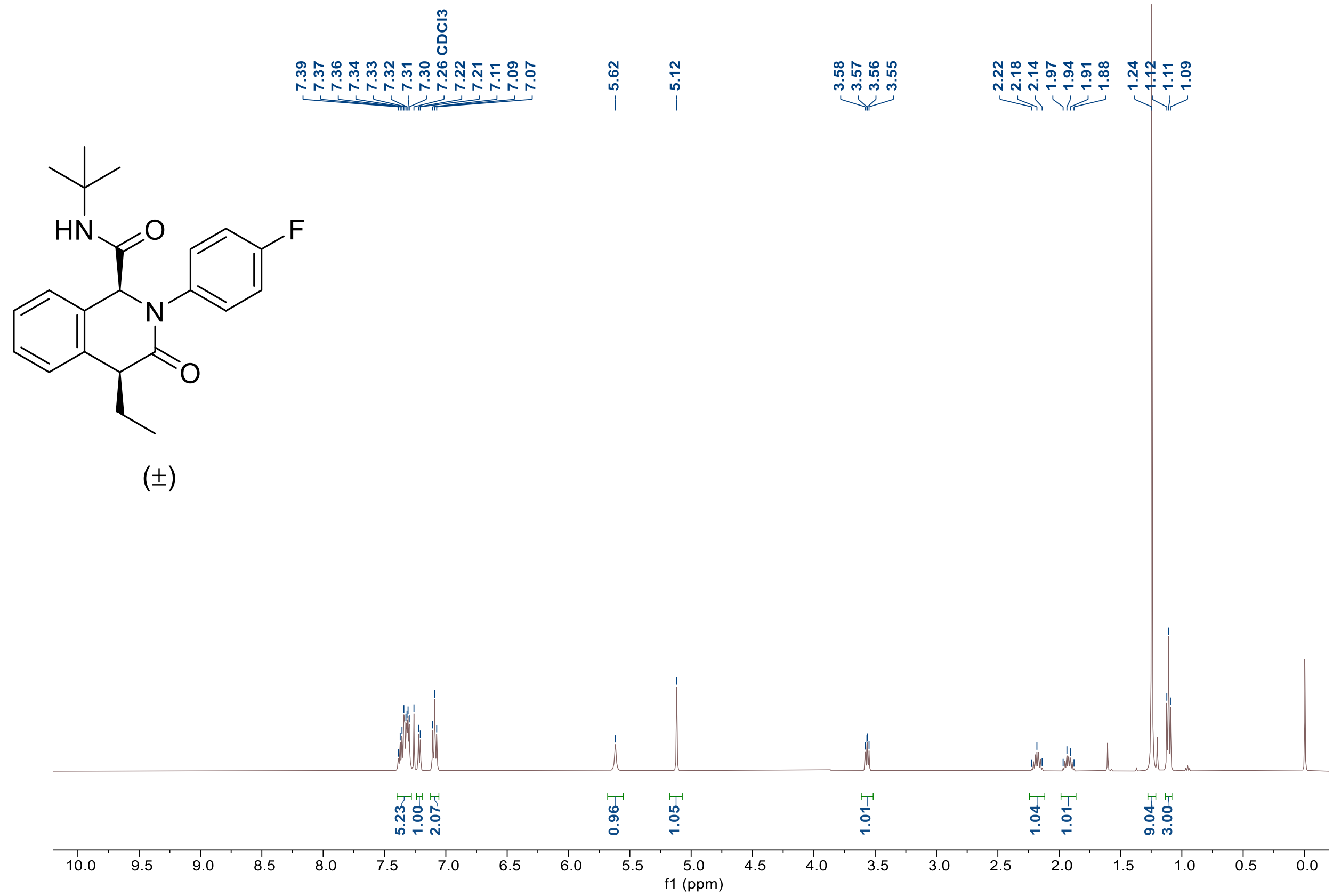

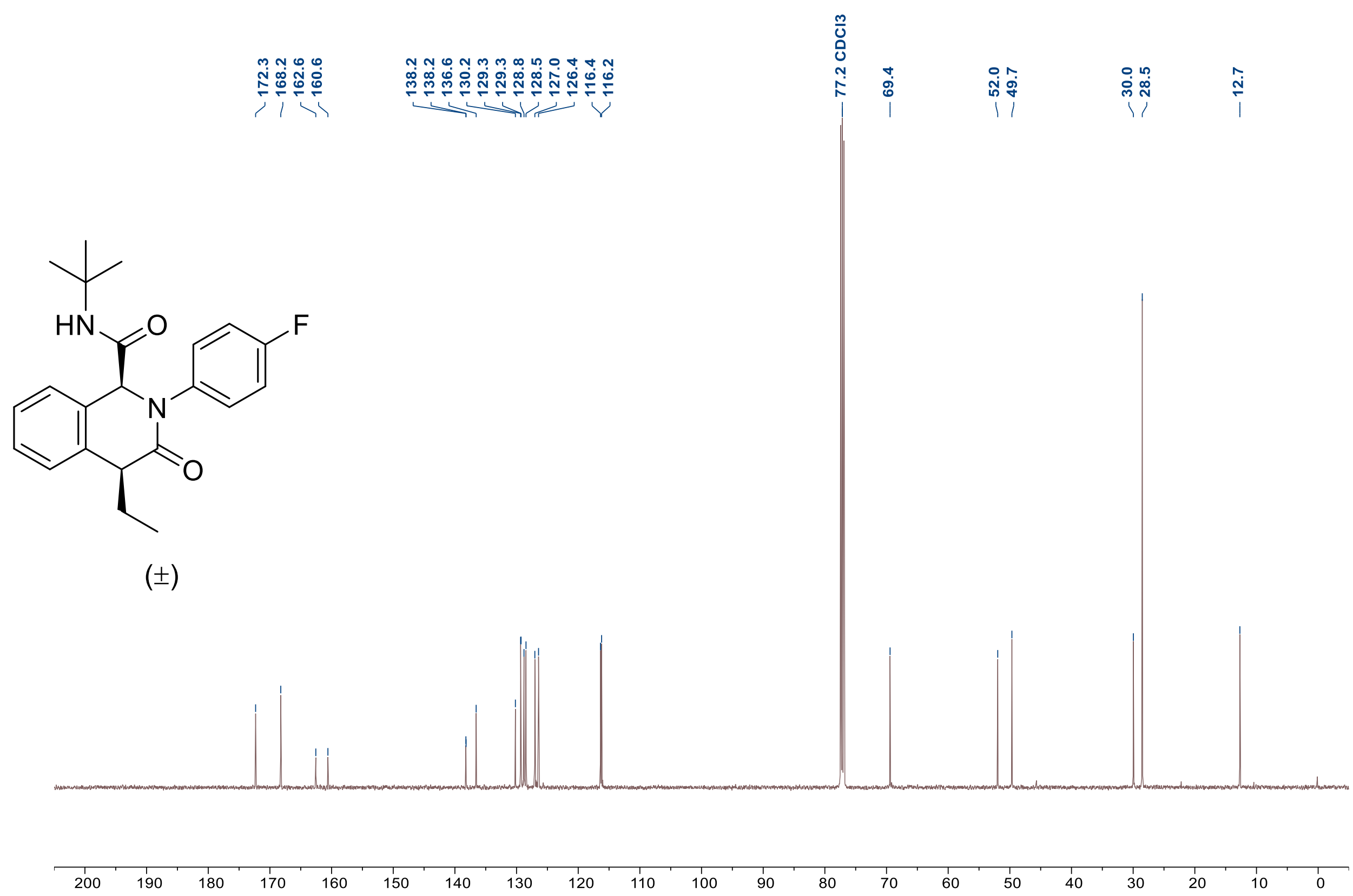

Copies of ^{1}H (400 MHz) and $^{13}C\{^{1}H\}$ (151 MHz) spectra of compound *cis*-**29h**

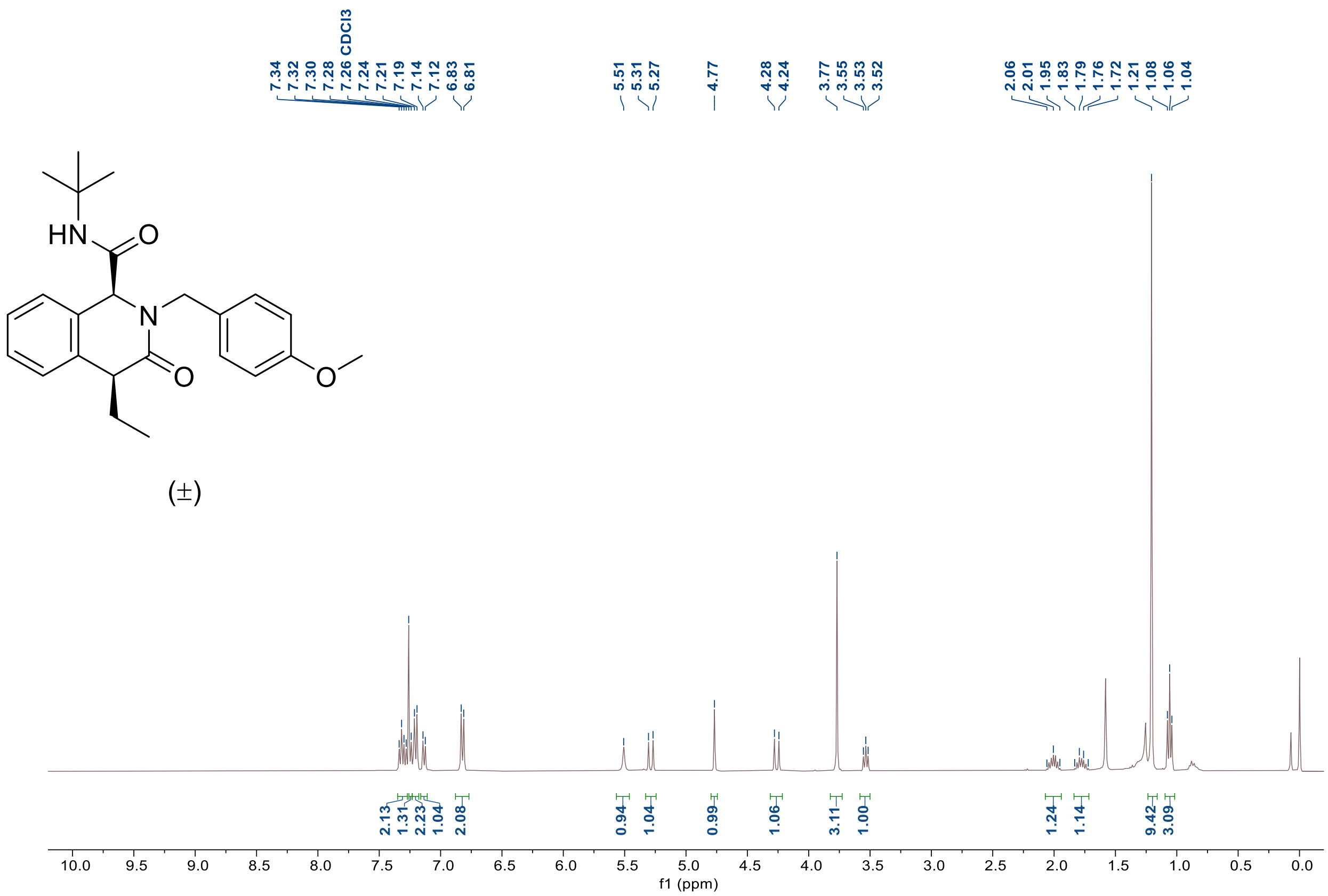

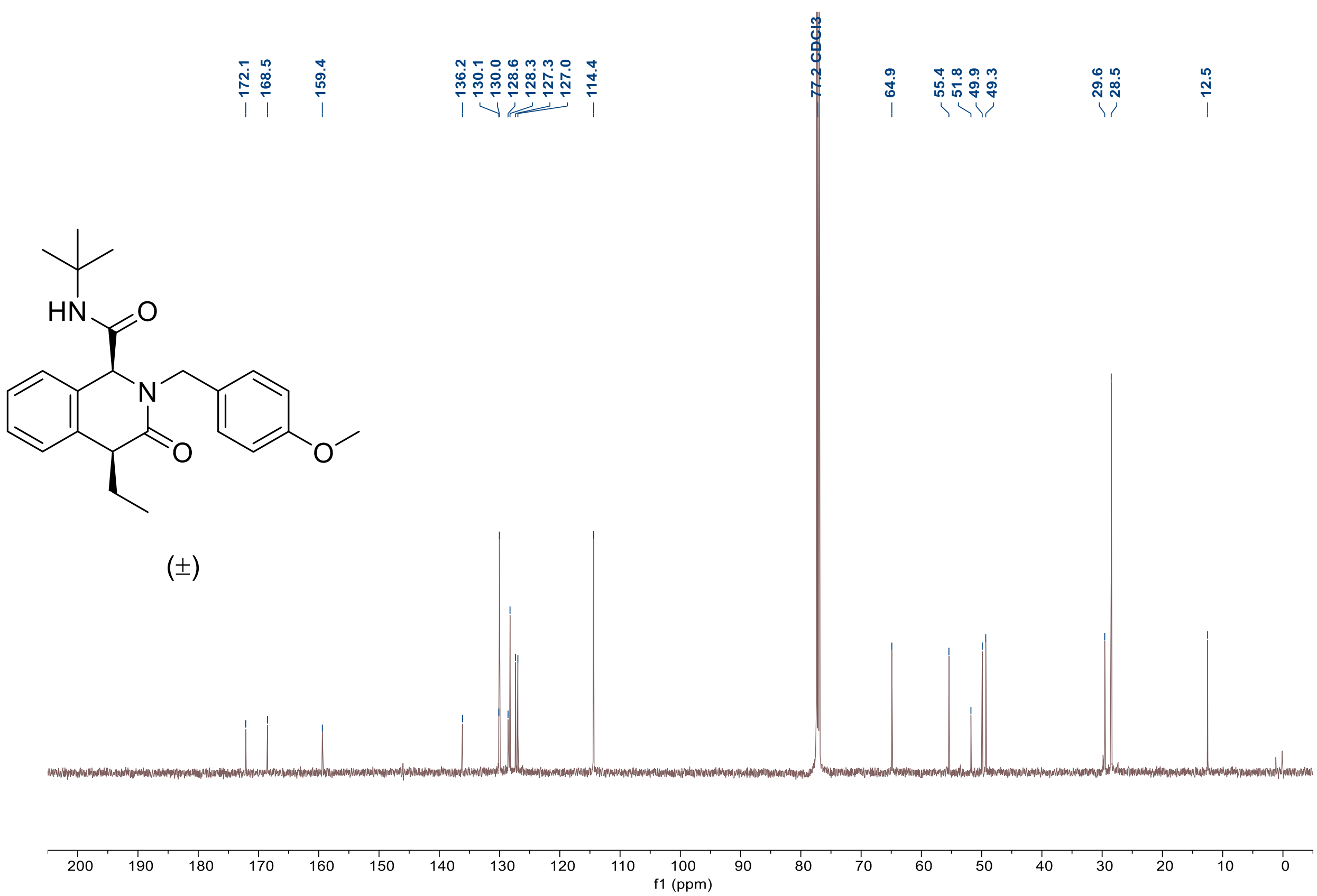

Copies of ^{1}H (400 MHz) and $^{13}C\{^{1}H\}$ (101 MHz) spectra of compound *cis*-**29i**

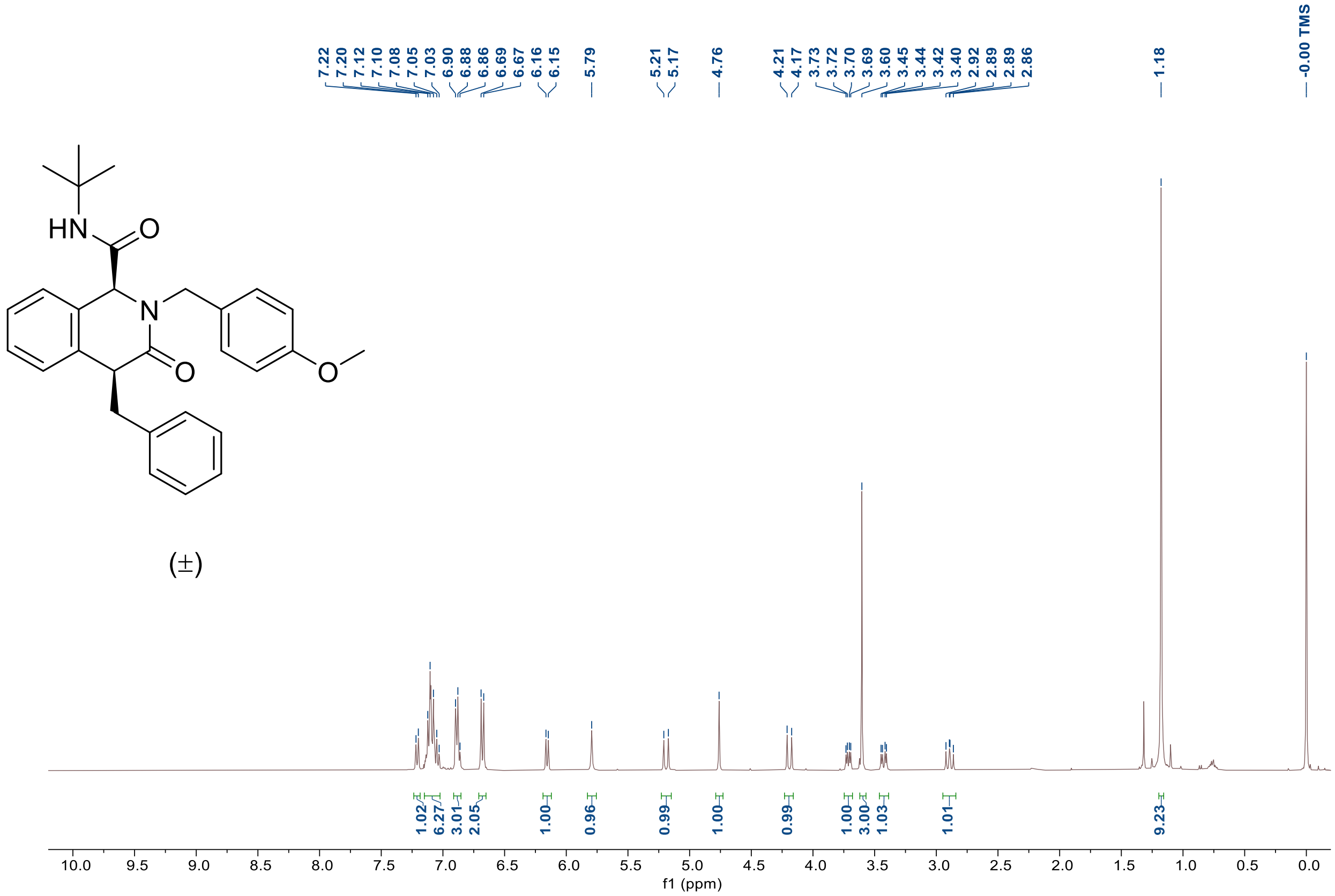

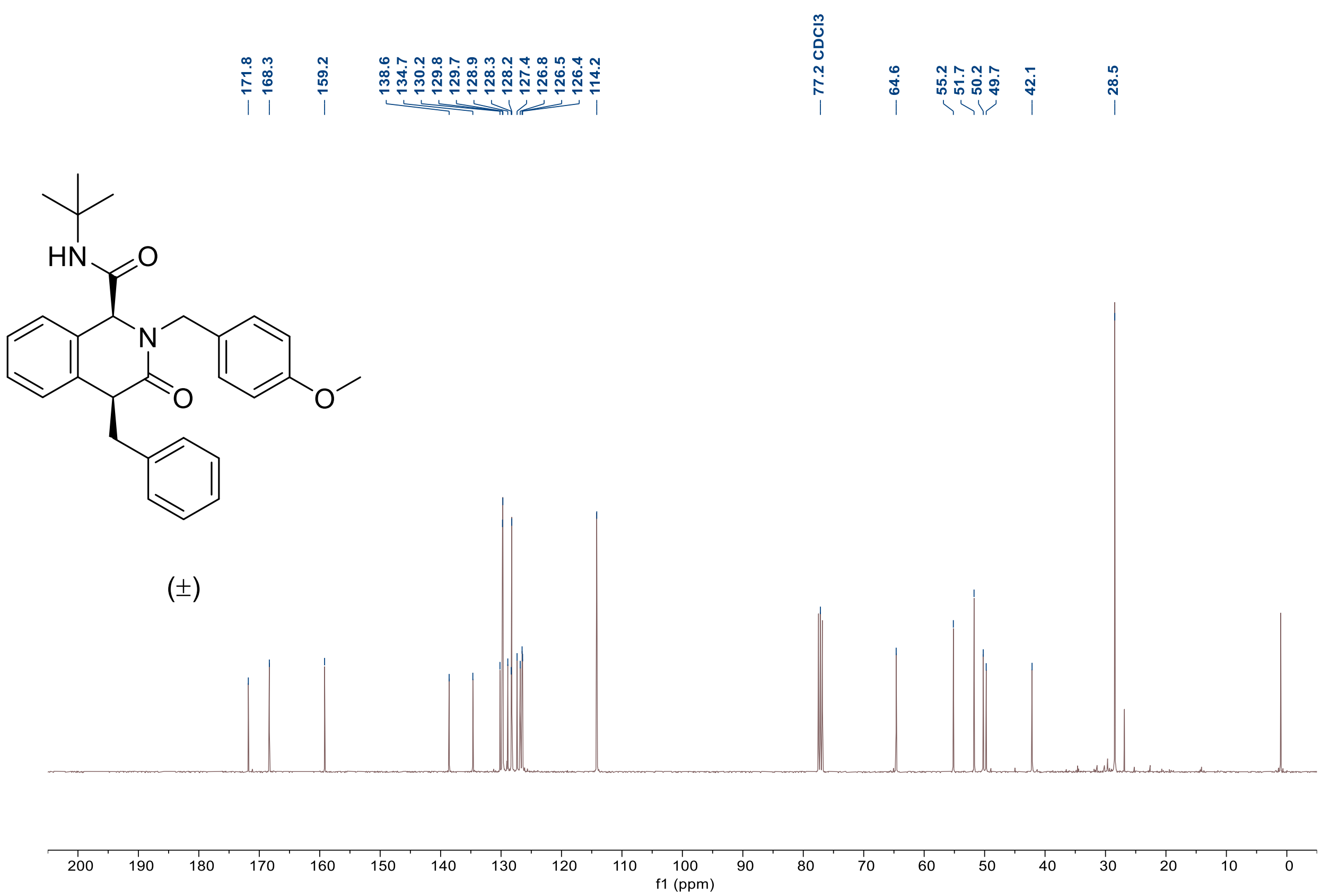

Copies of ^{1}H (400 MHz) and $^{13}C\{^{1}H\}$ (101 MHz) spectra of compound *cis*-**29j**

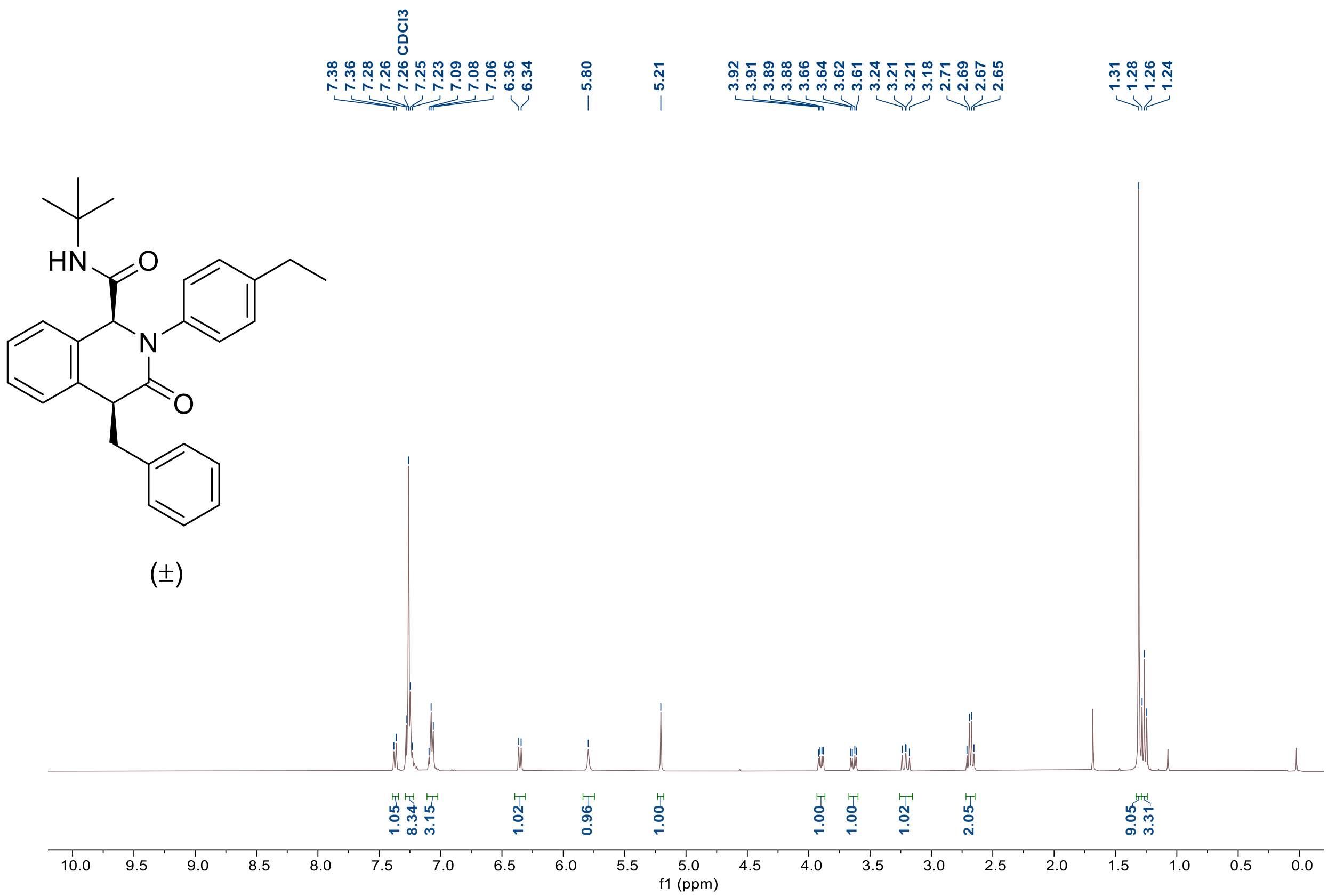

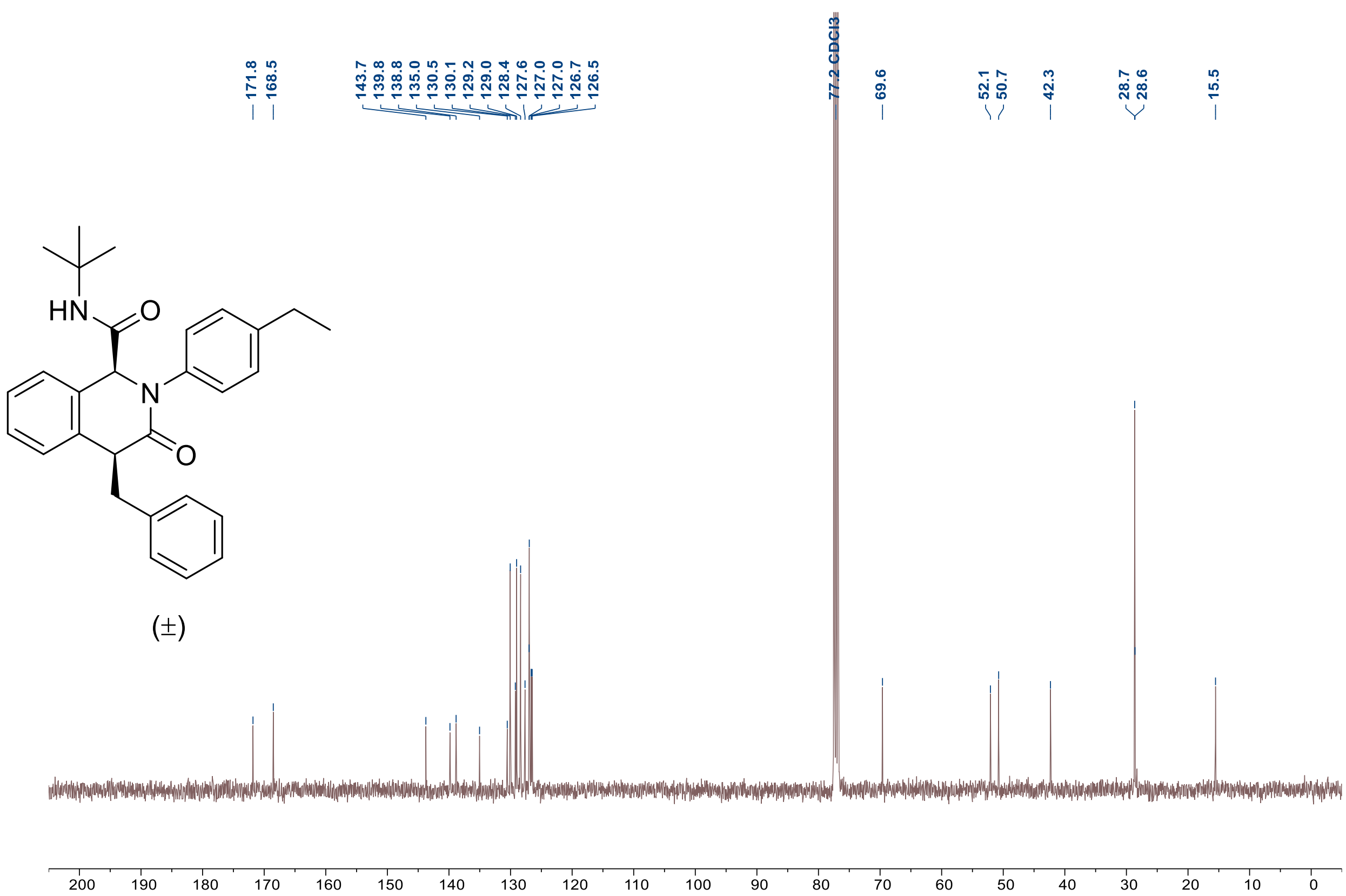

Copies of ^{1}H (400 MHz) and $^{13}C\{^{1}H\}$ (101 MHz) spectra of compound *cis*-**29k**

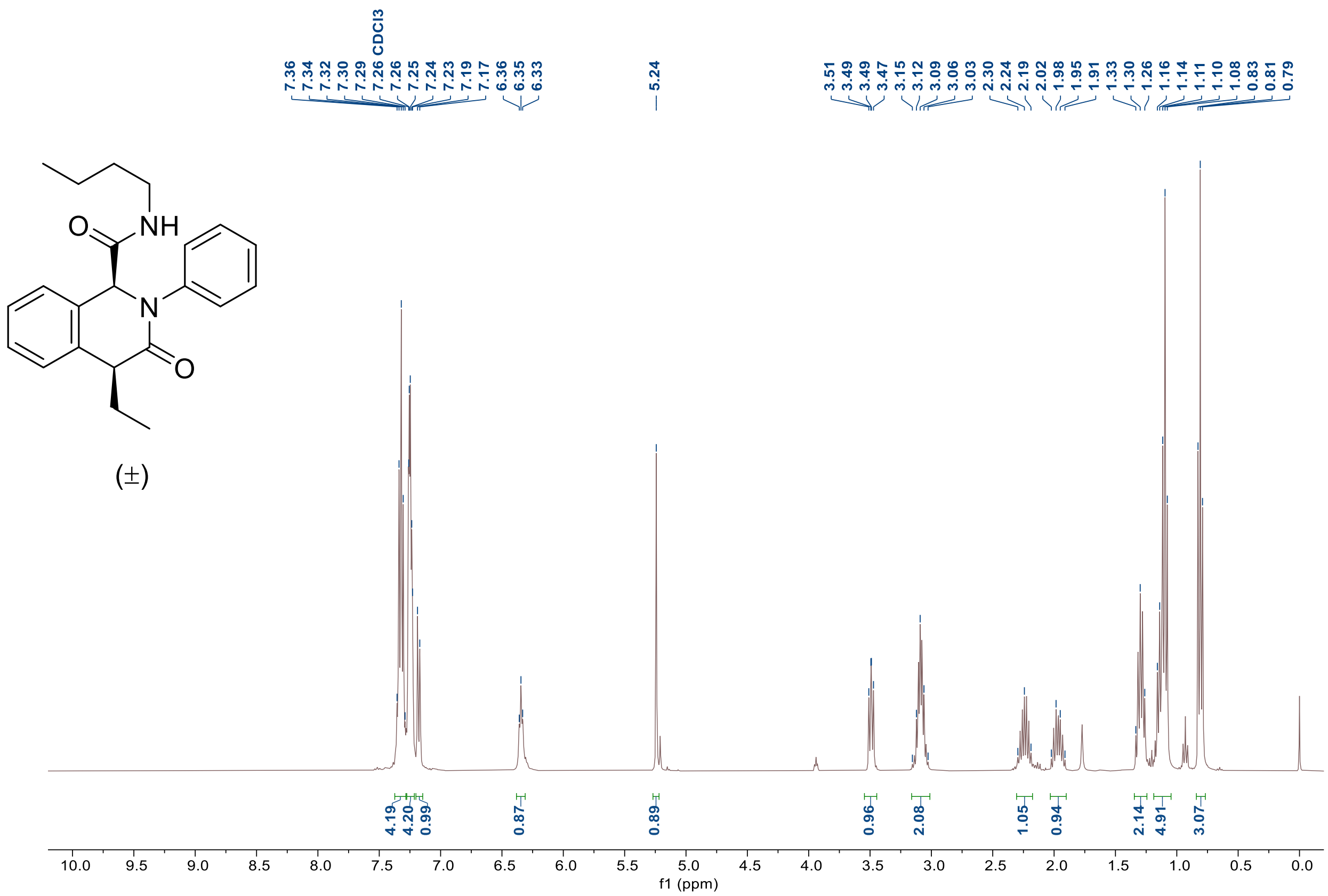

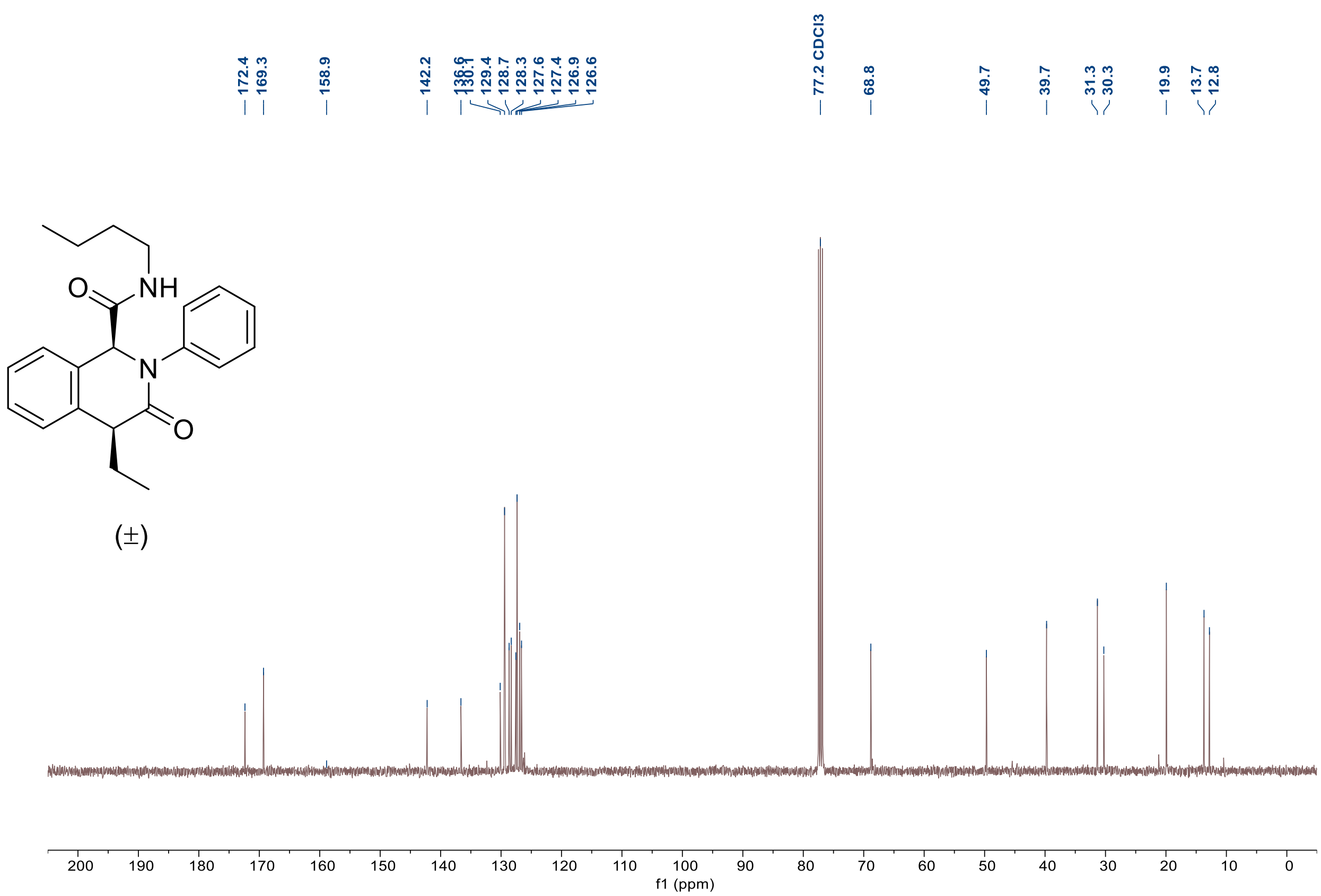

Copies of ^{1}H (500 MHz) and $^{13}C\{^{1}H\}$ (126 MHz) spectra of compound *cis*-**29l**

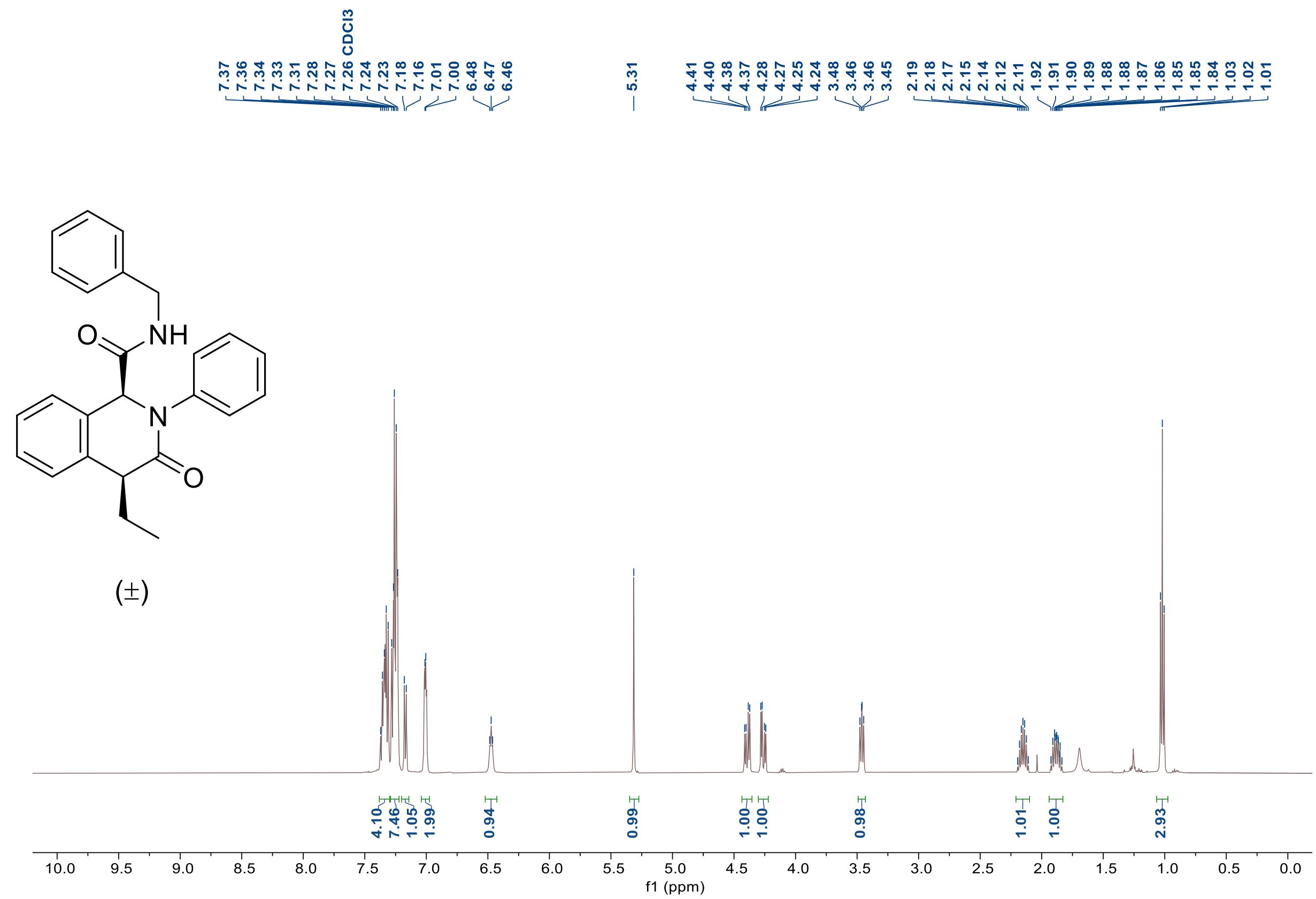

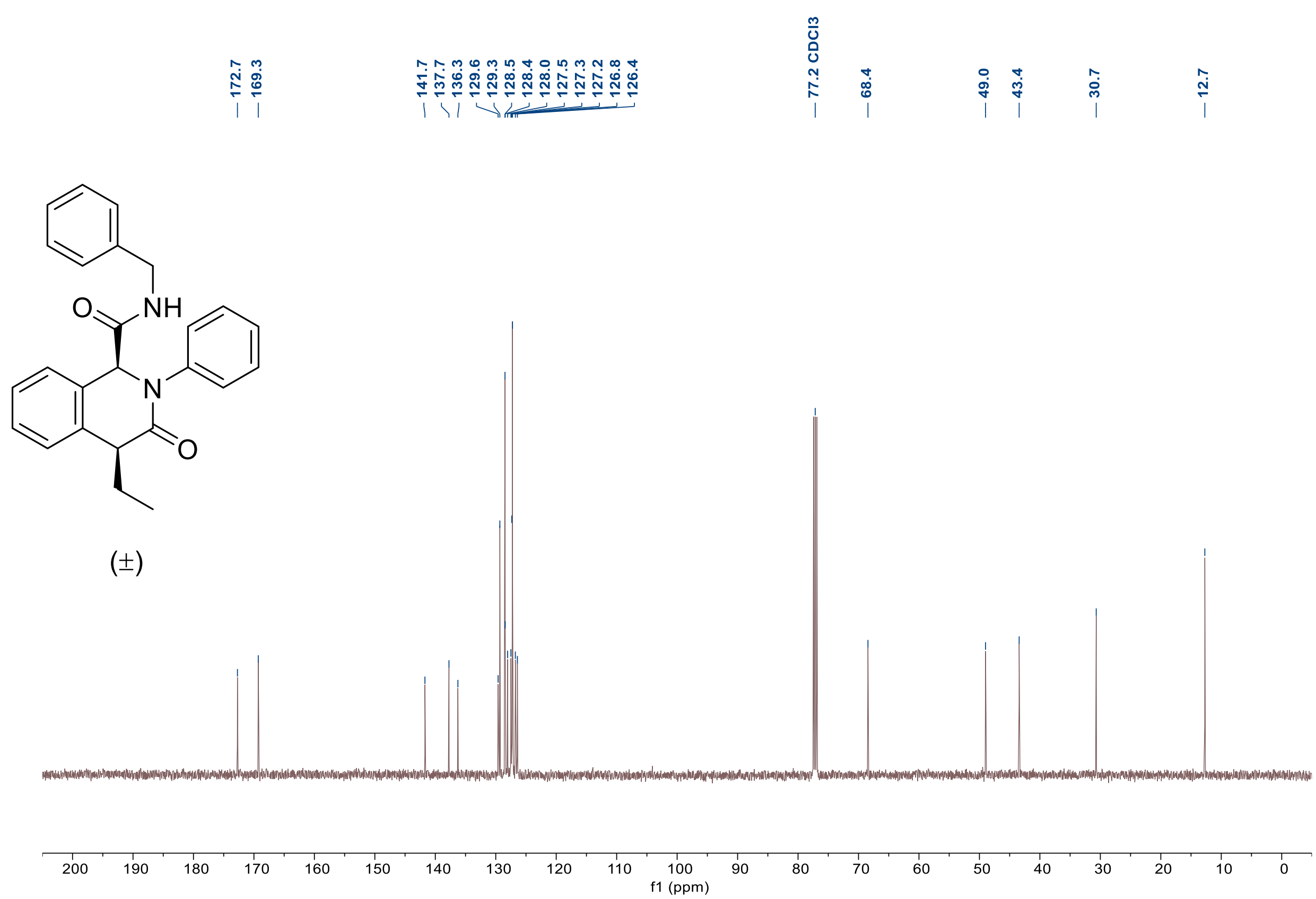

Copies of ^{1}H (500 MHz) and $^{13}C\{^{1}H\}$ (126 MHz) spectra of compound *trans*-**29l**

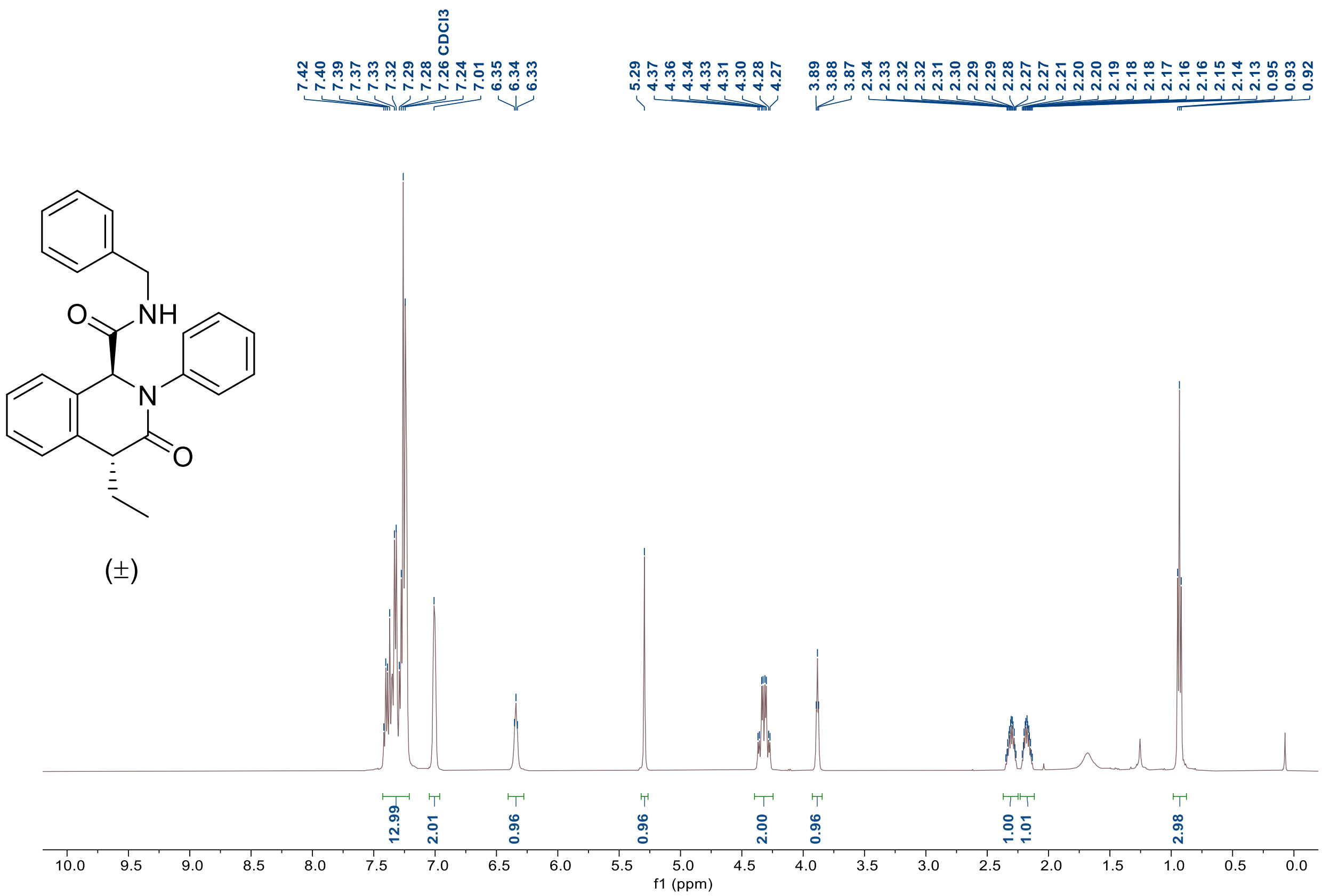

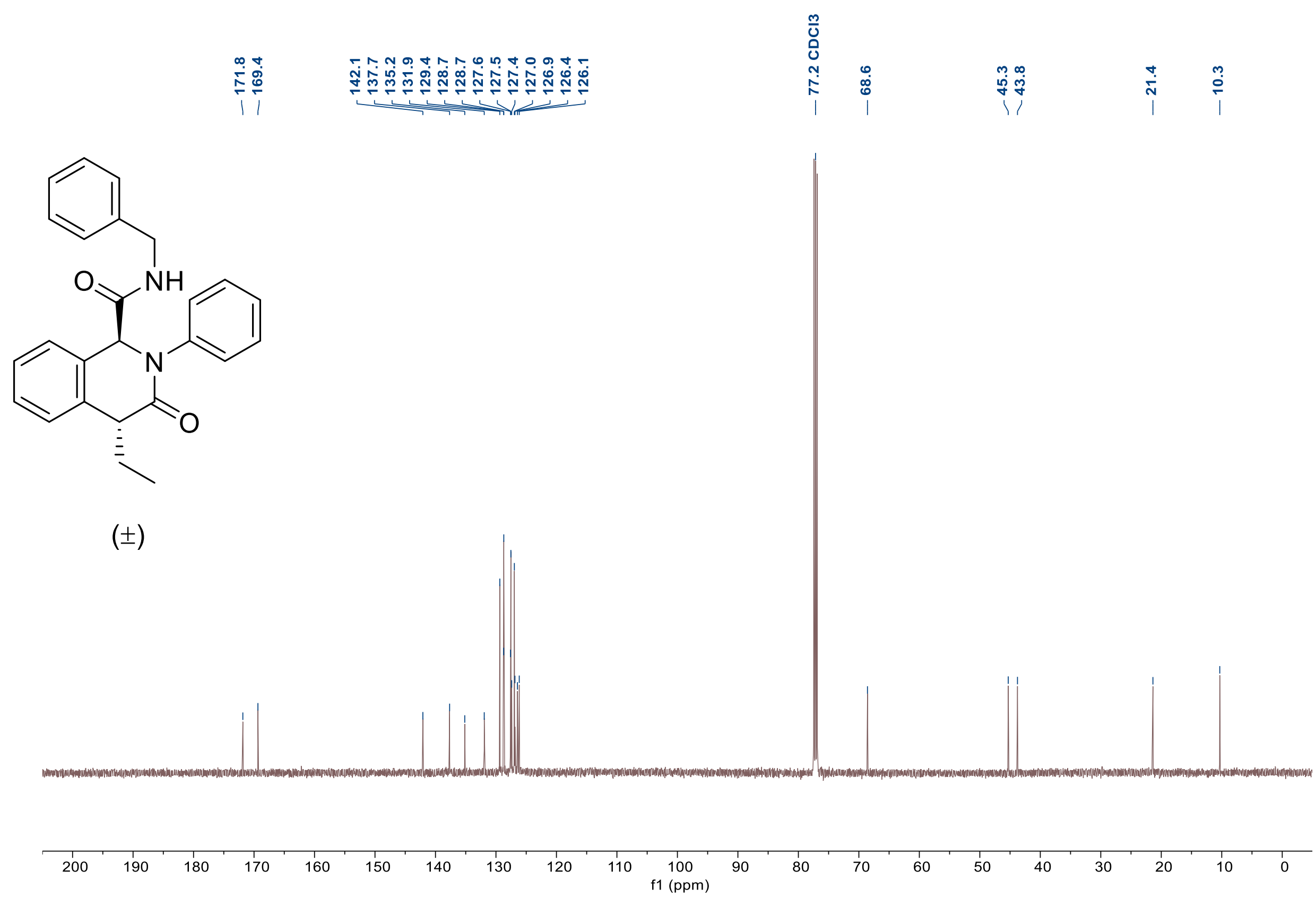

Copies of ^{1}H (500 MHz) and $^{13}C\{^{1}H\}$ (126 MHz) spectra of compound **30**

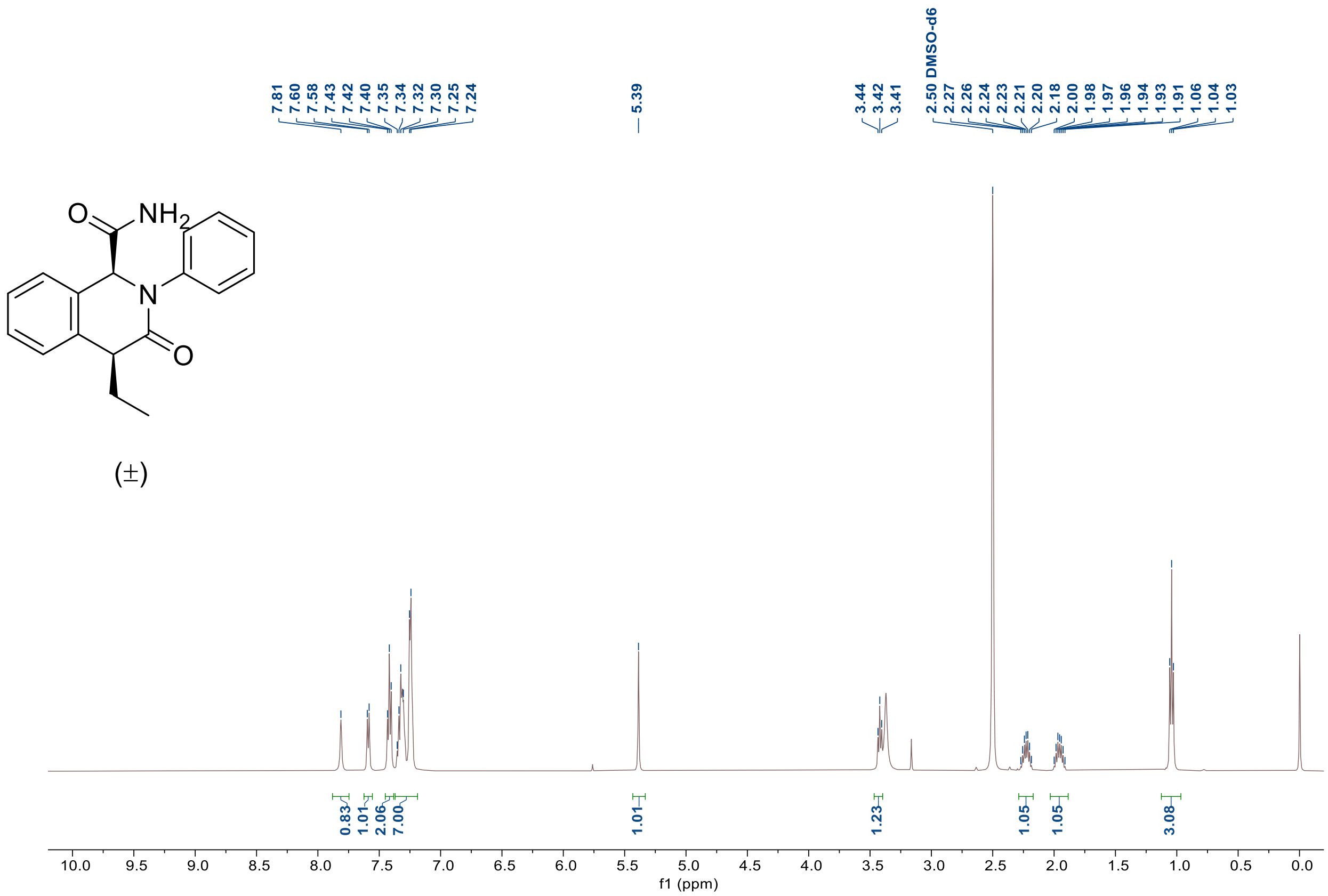

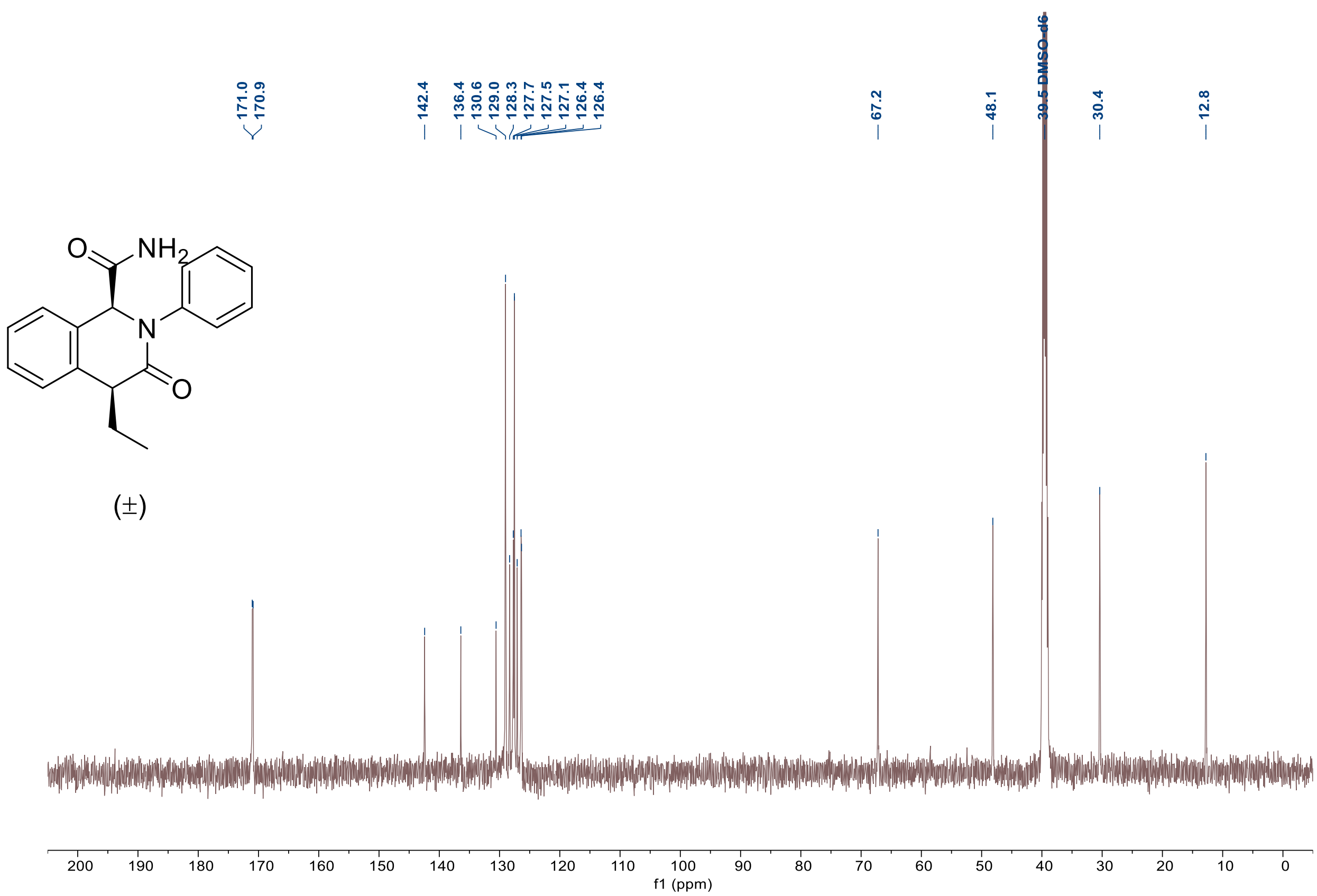

Copies of ^{1}H (500 MHz) and $^{13}C\{^{1}H\}$ (126 MHz) spectra of compound **31**

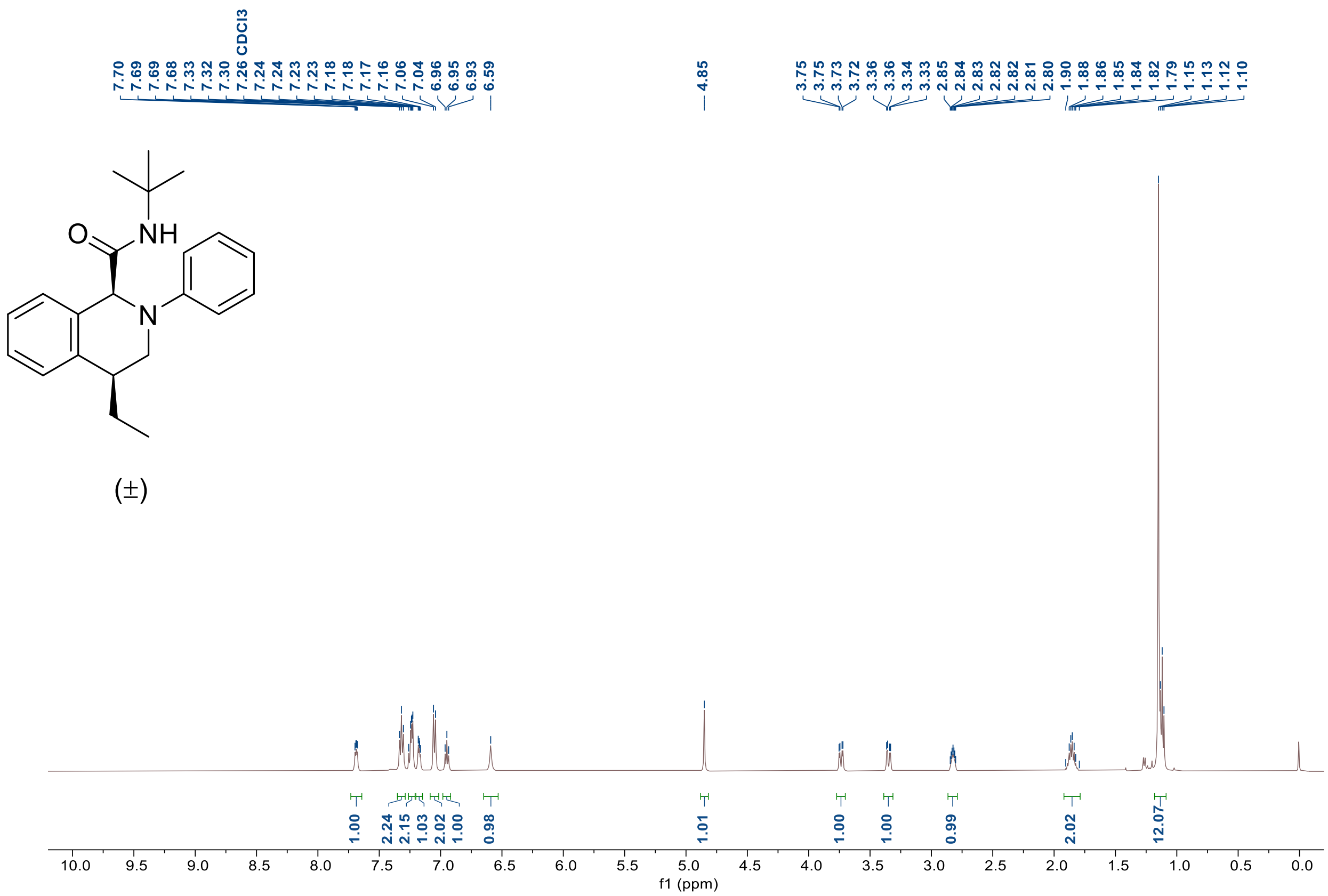

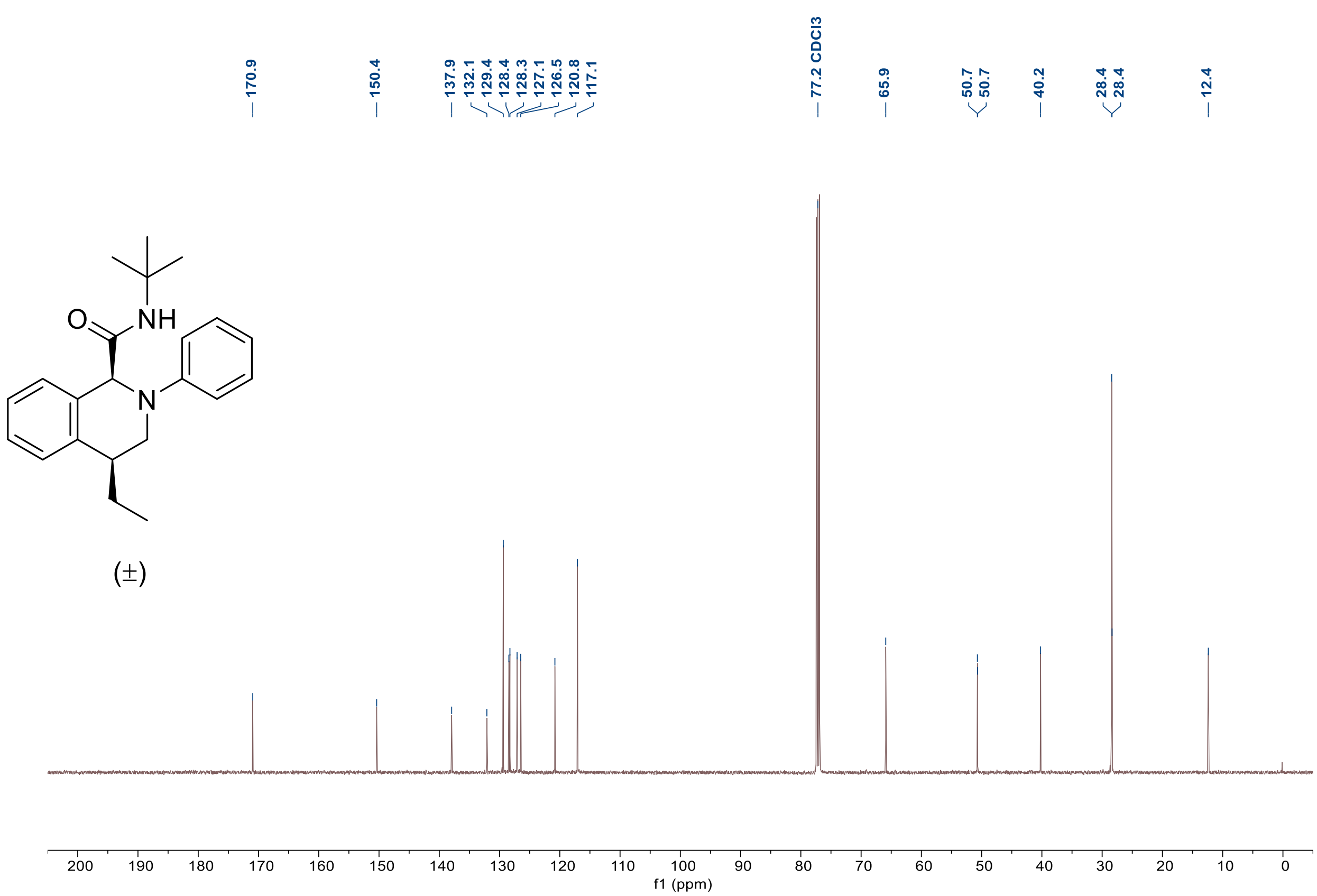

Copies of ^{1}H (500 MHz) and ^{13}C{^{1}H} (126 MHz) spectra of compound **33**

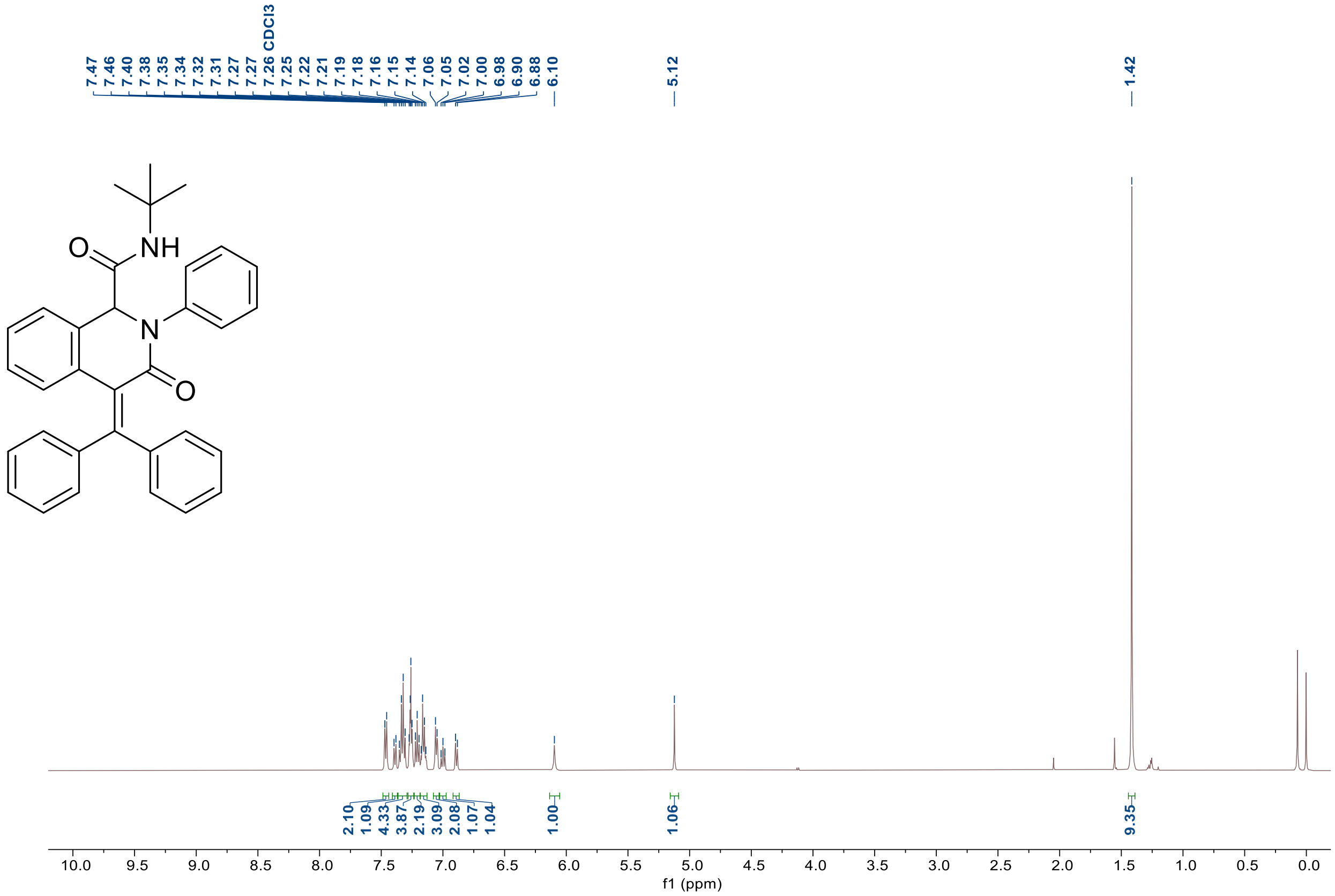

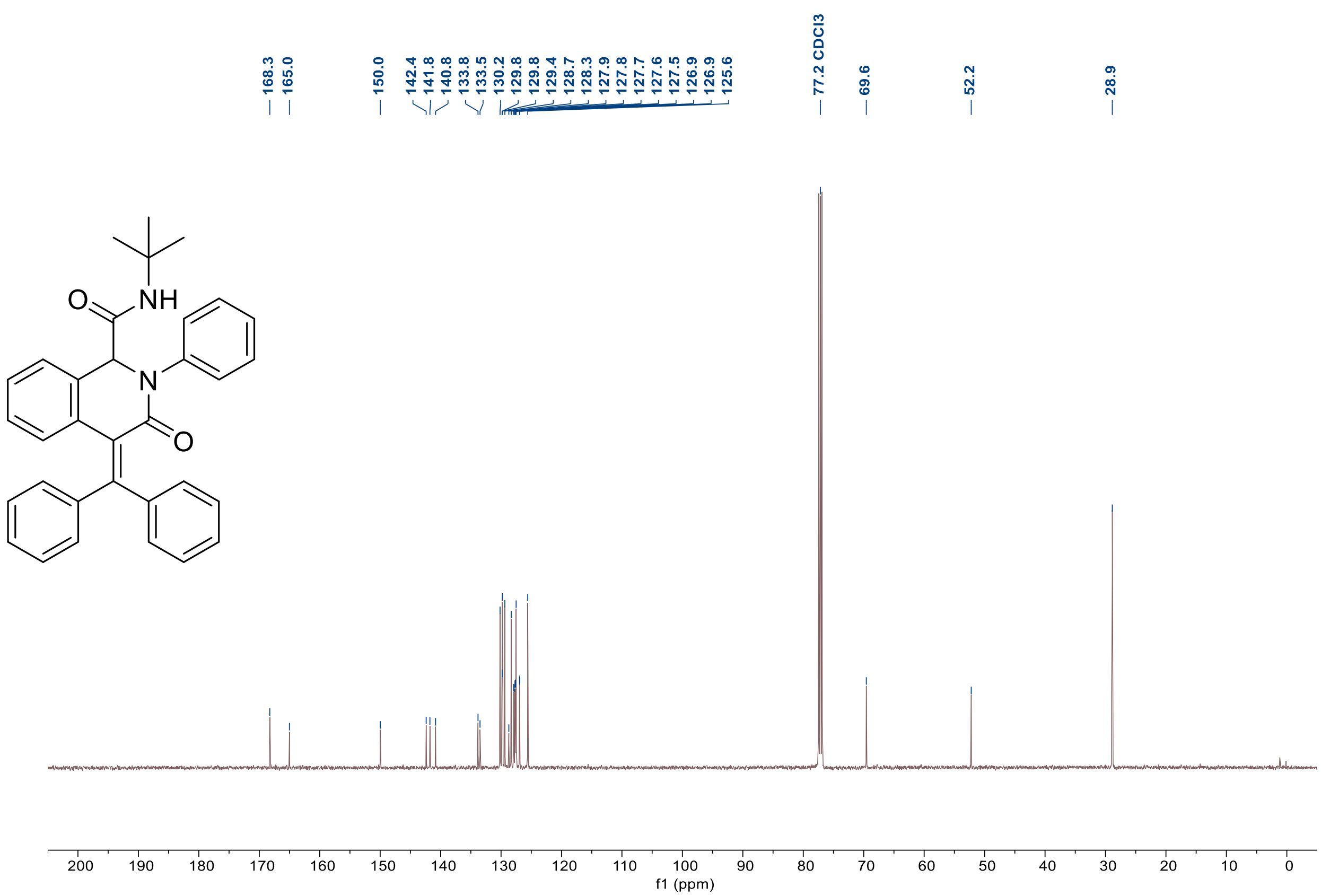

Copies of ^1H (500 MHz) and $^{13}C\{^1H\}$ (126 MHz) spectra of compound *cis*-**34**

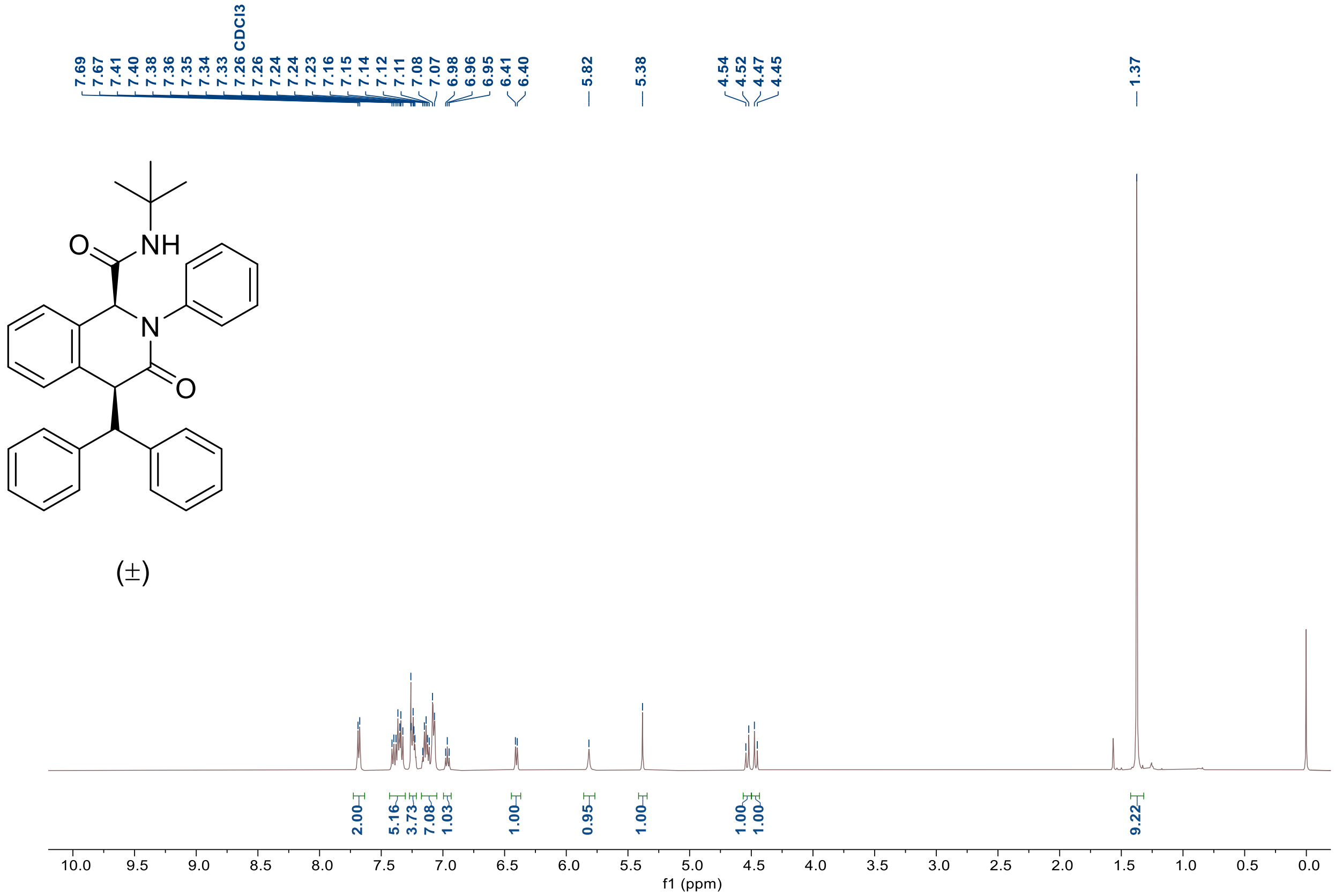

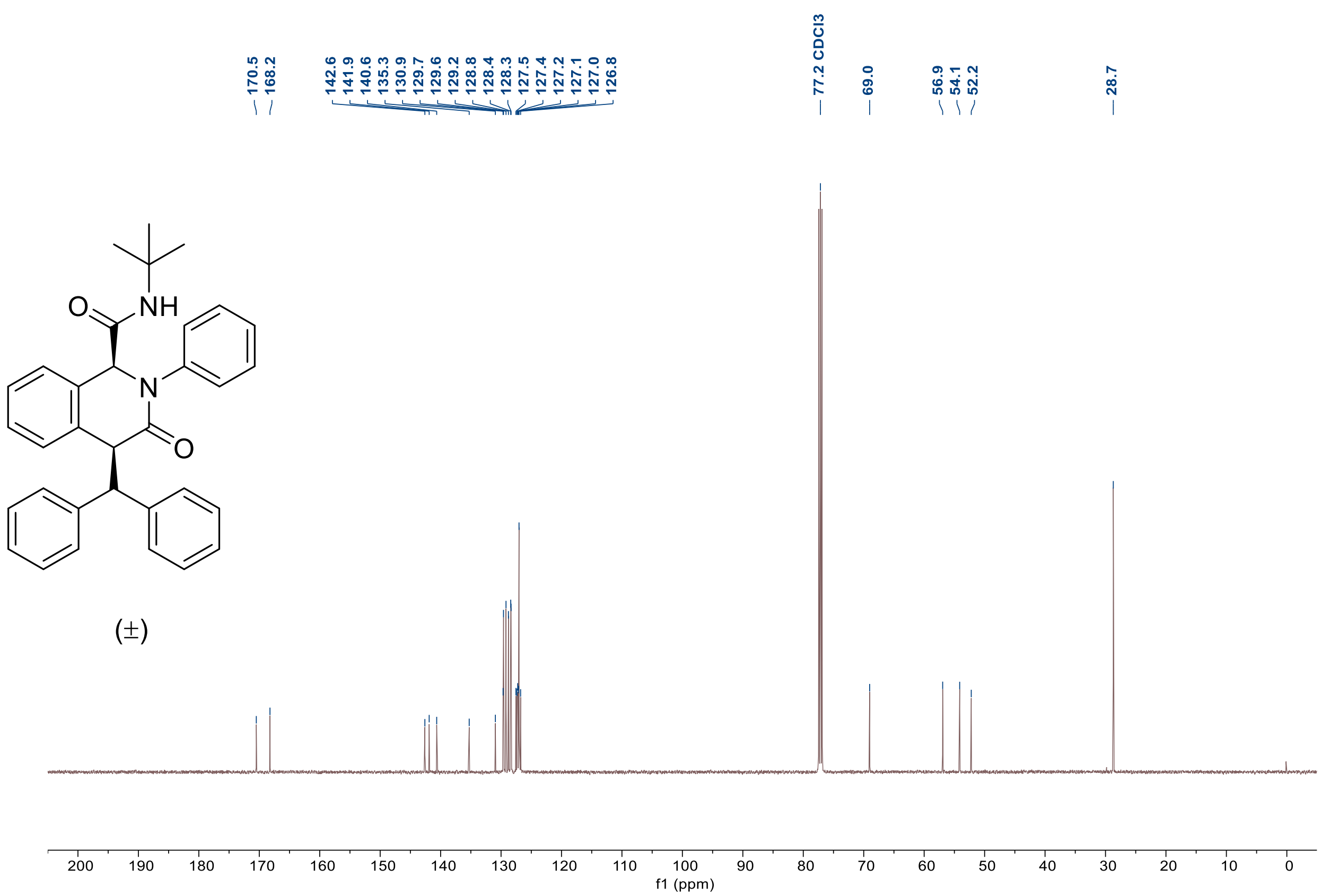

Copies of ^{1}H (500 MHz) and $^{13}C\{^{1}H\}$ (126 MHz) spectra of compound *trans*-**34**

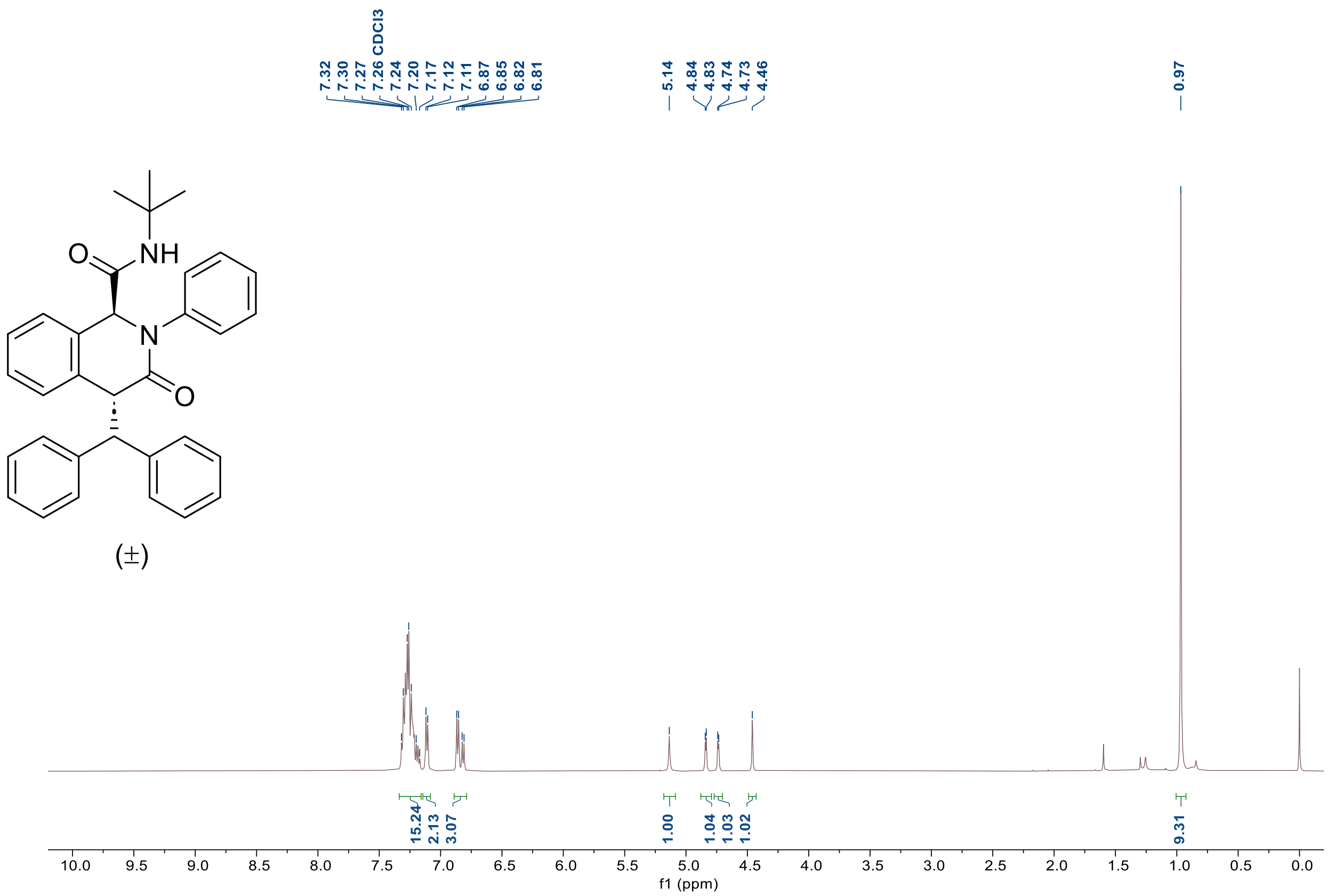

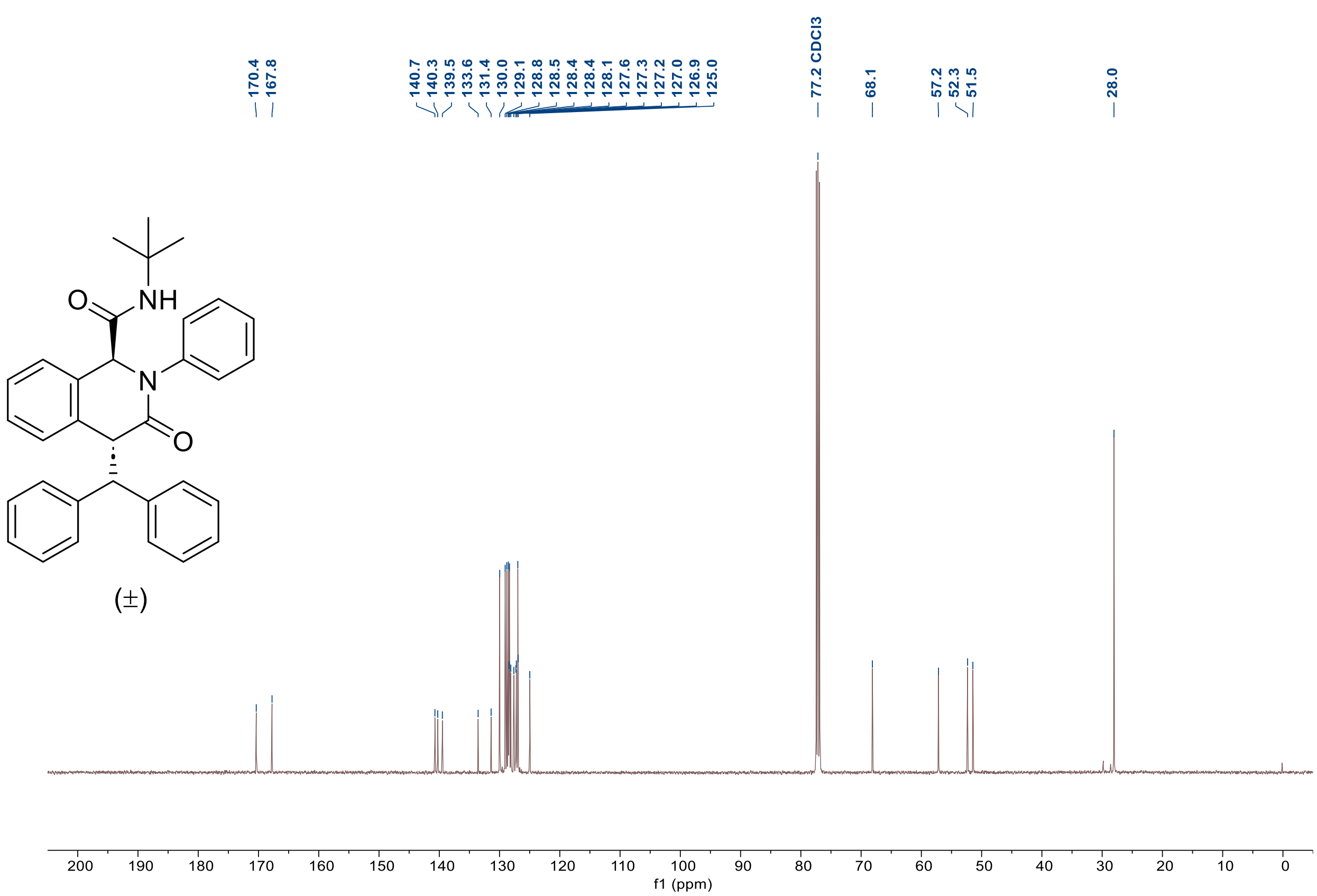

Copies of ^{1}H (500 MHz) and $^{13}C\{^{1}H\}$ (126 MHz) spectra of compound **35**

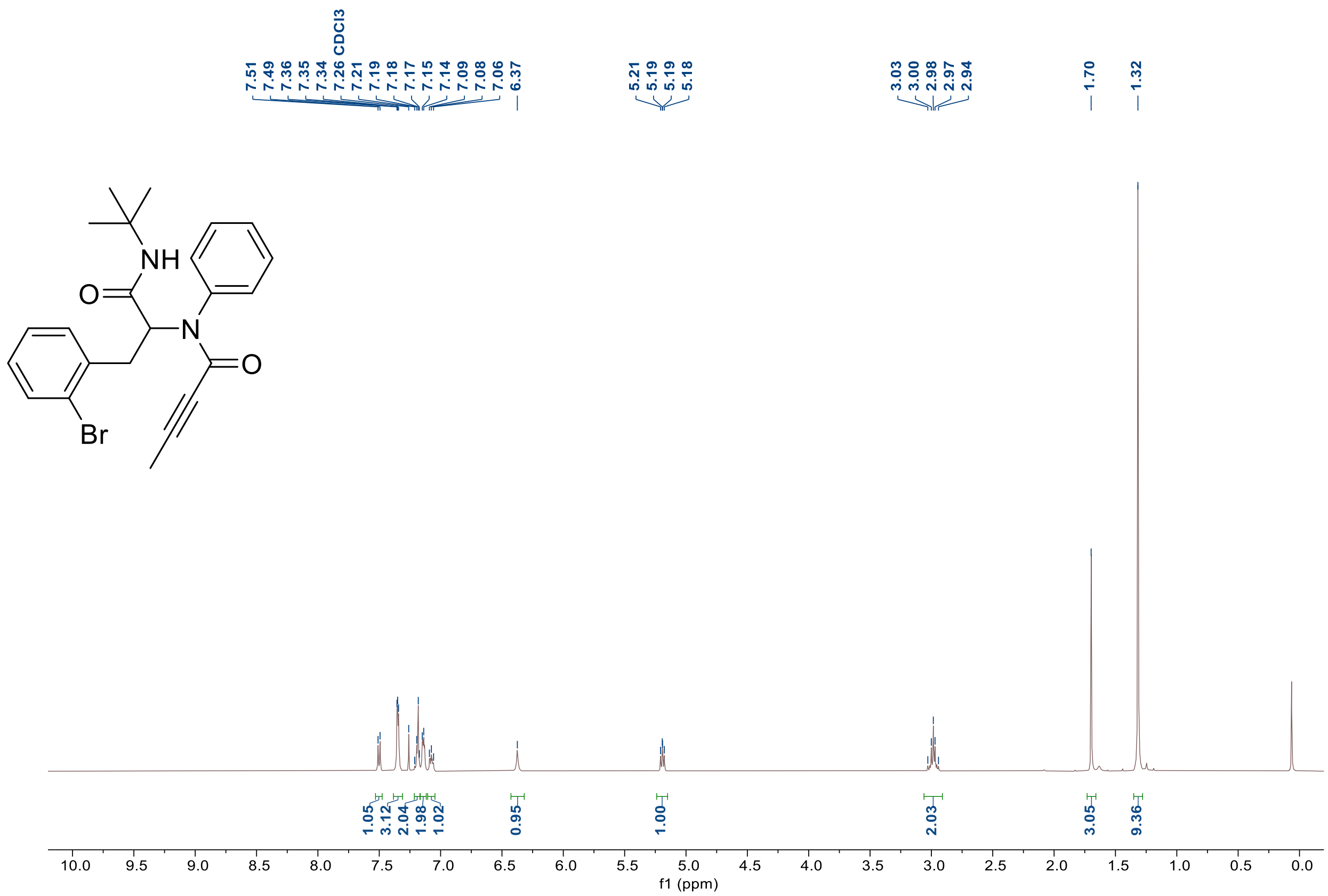

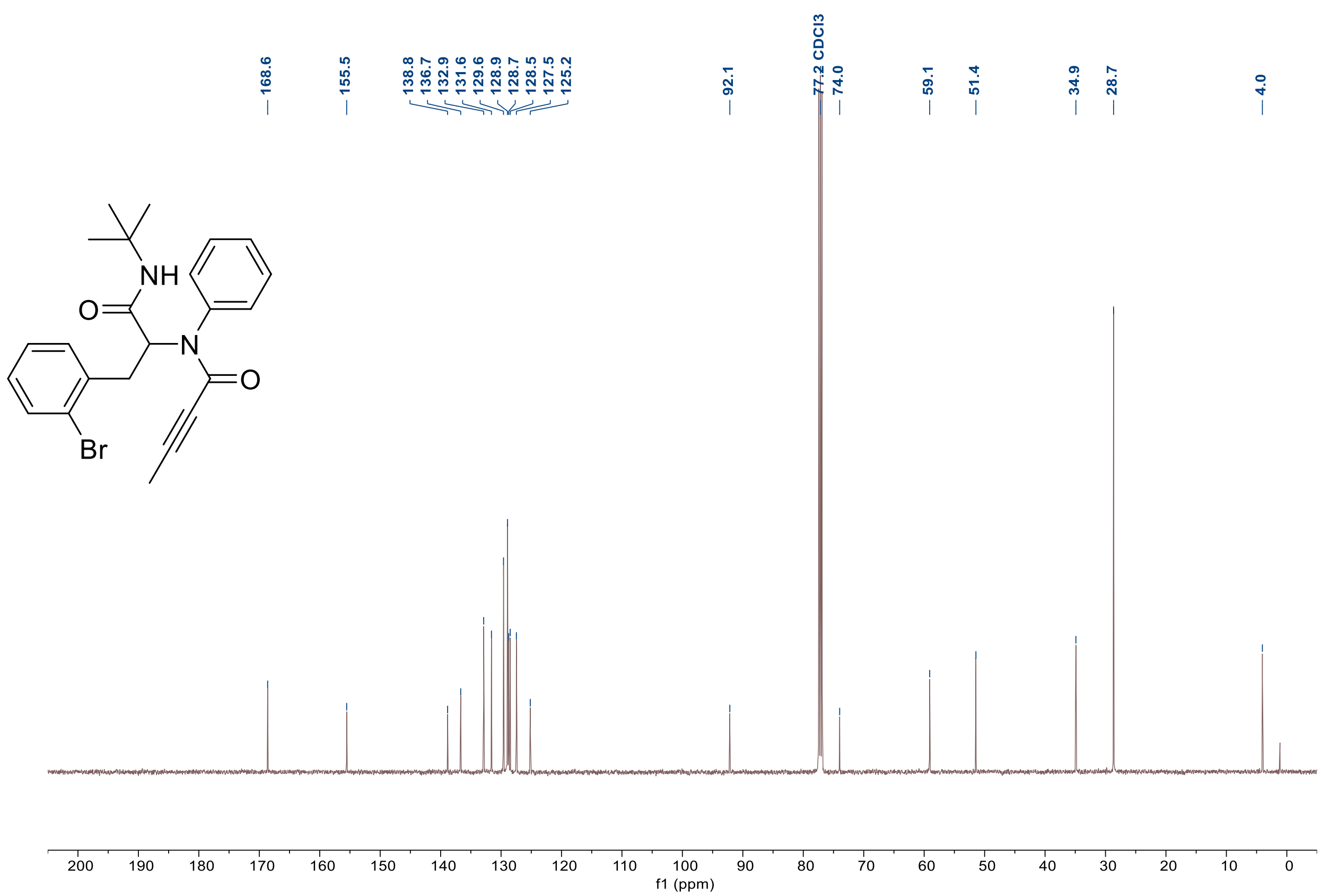

Copies of ^{1}H (500 MHz) and $^{13}C\{^{1}H\}$ (126 MHz) spectra of compound **36**

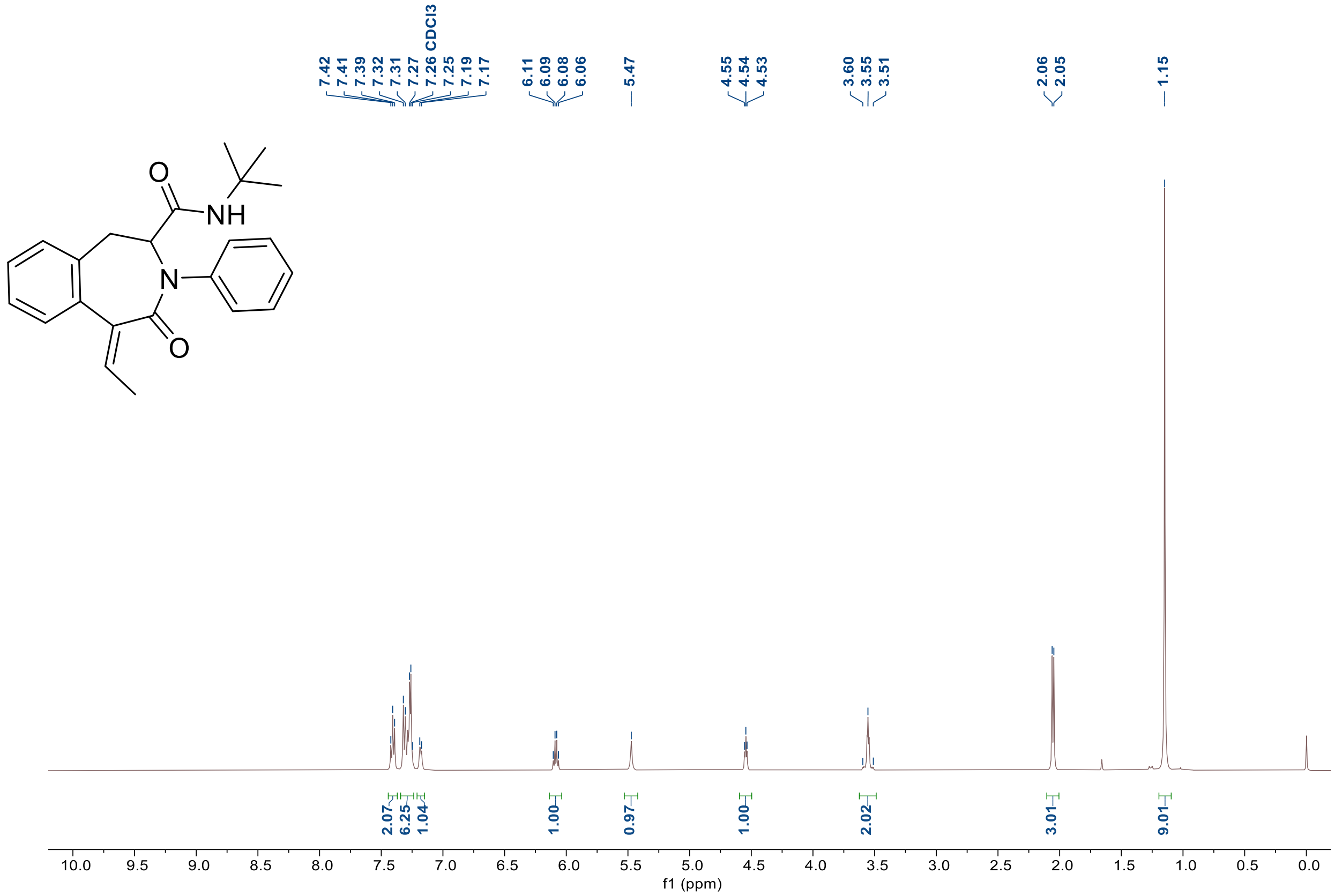

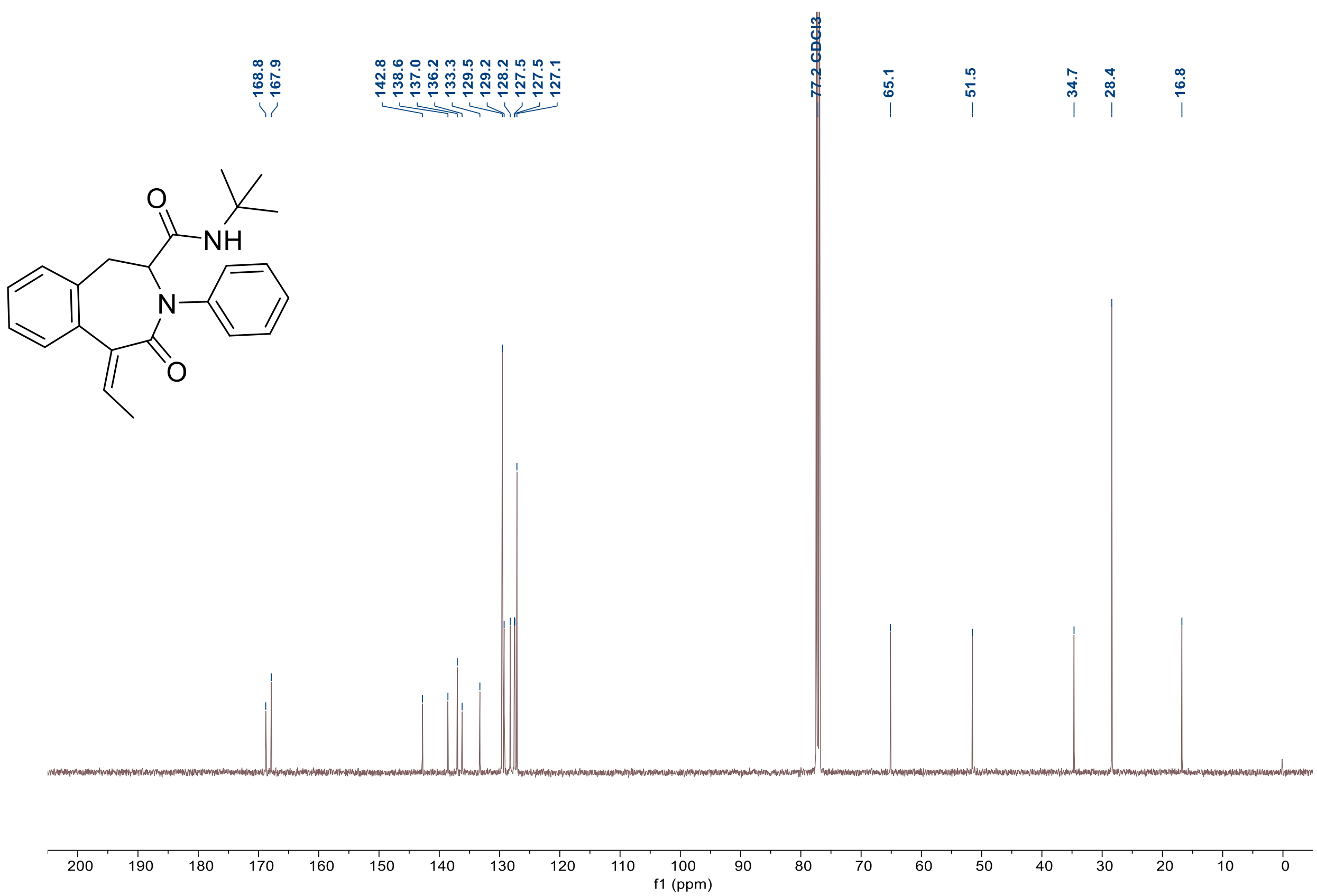

Copies of ^{1}H (500 MHz) and $^{13}C\{^{1}H\}$ (126 MHz) spectra of compound *cis*-**37**

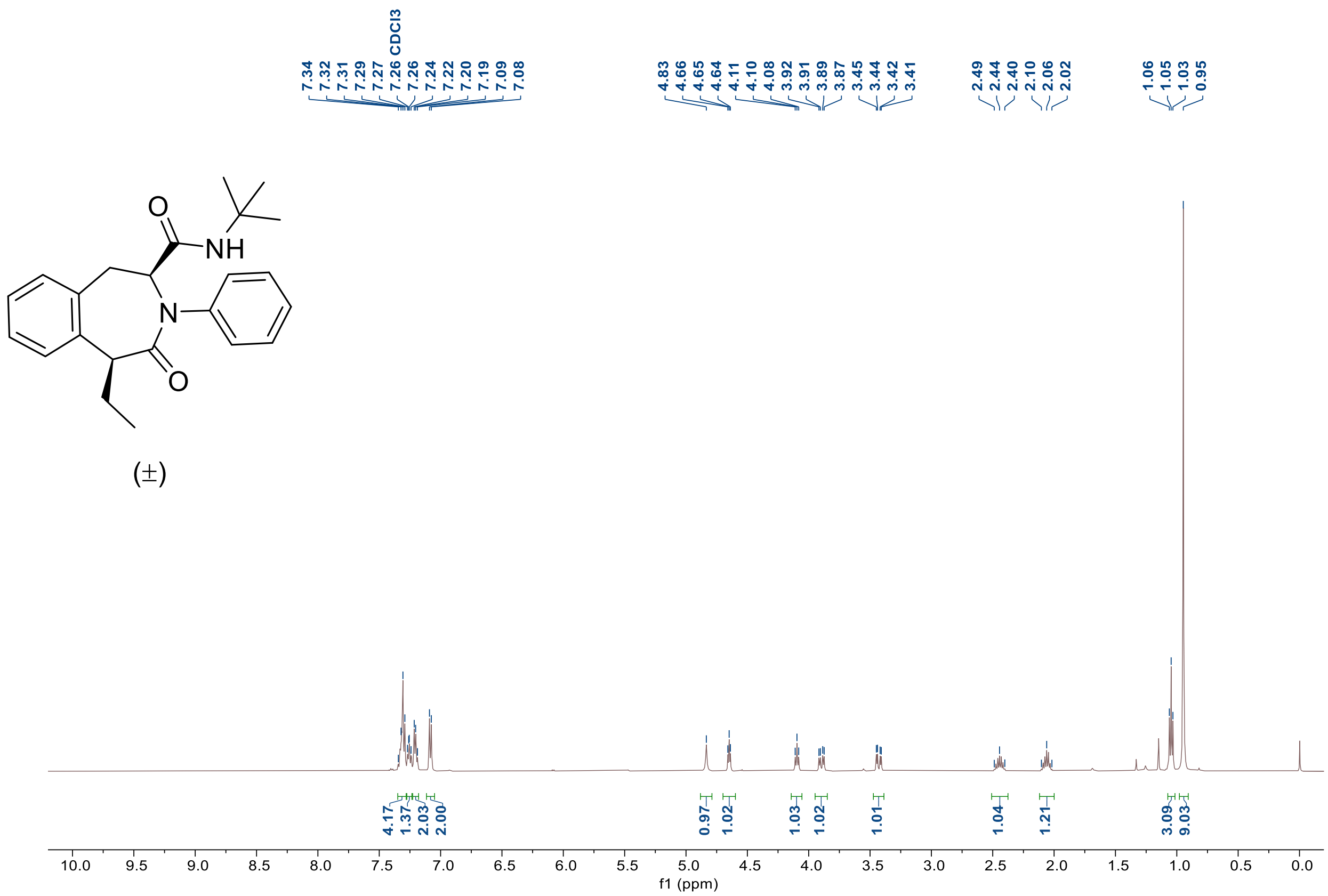

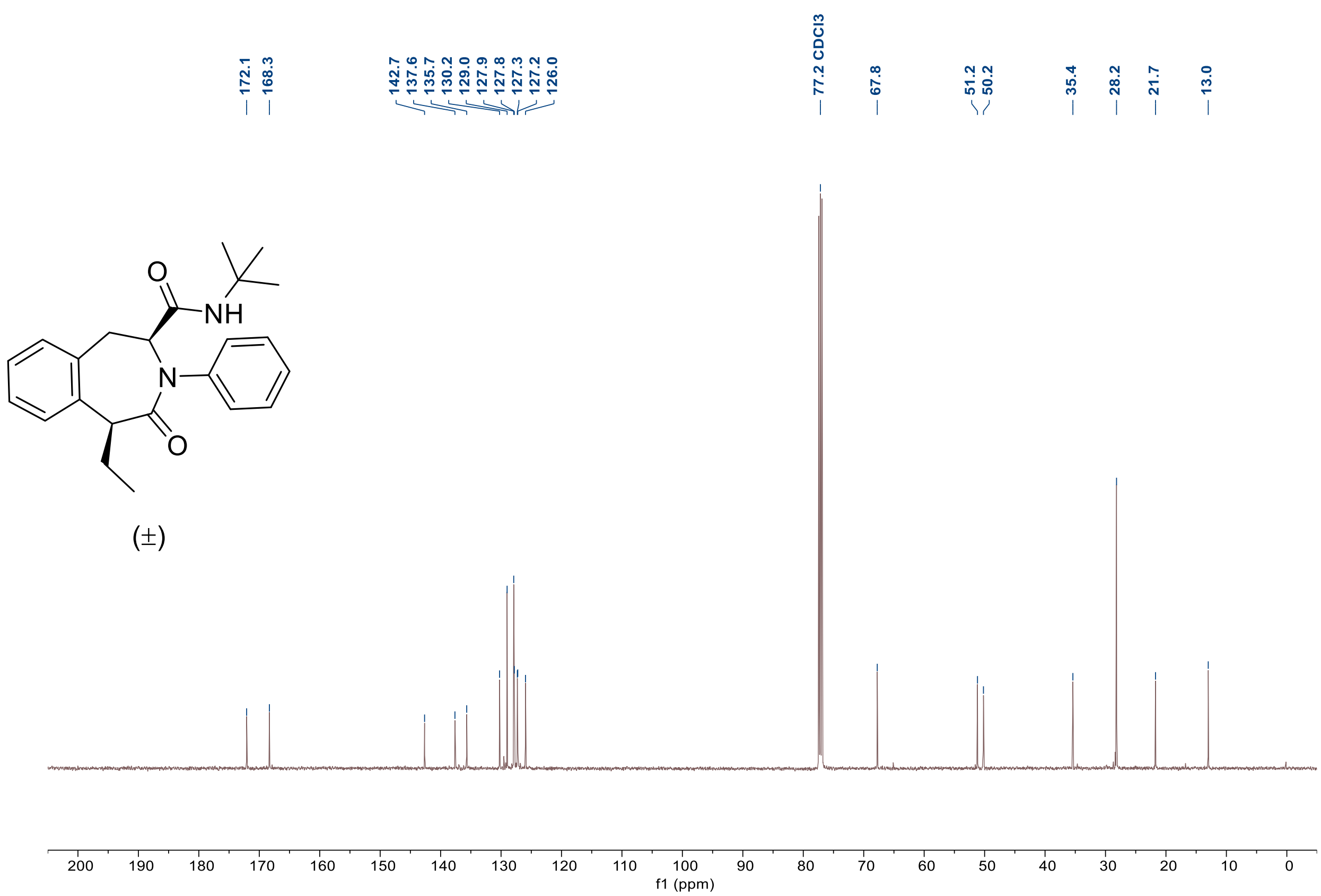

Copies of ^{1}H (500 MHz) and $^{13}C\{^{1}H\}$ (126 MHz) spectra of compound *trans*-**37**

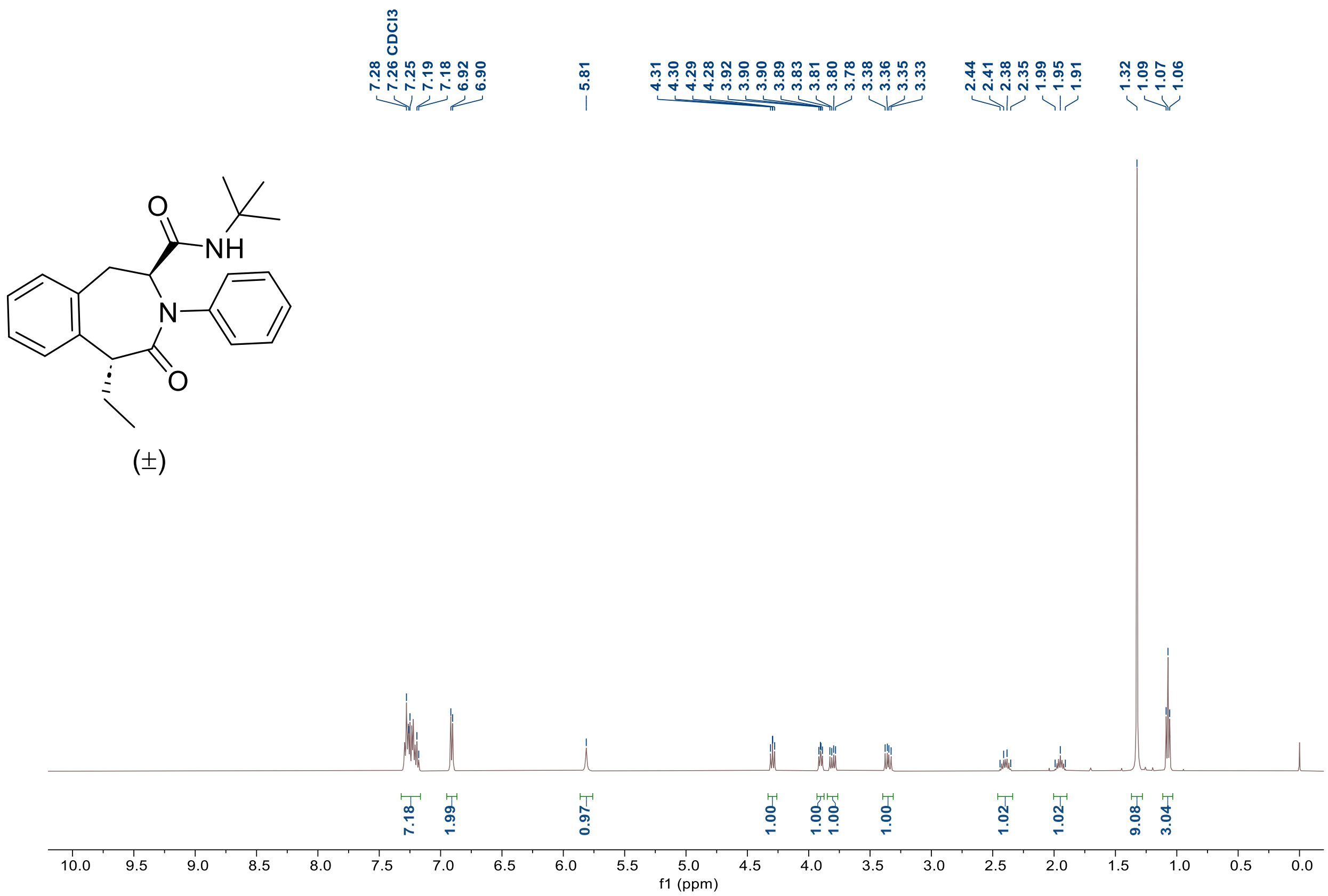

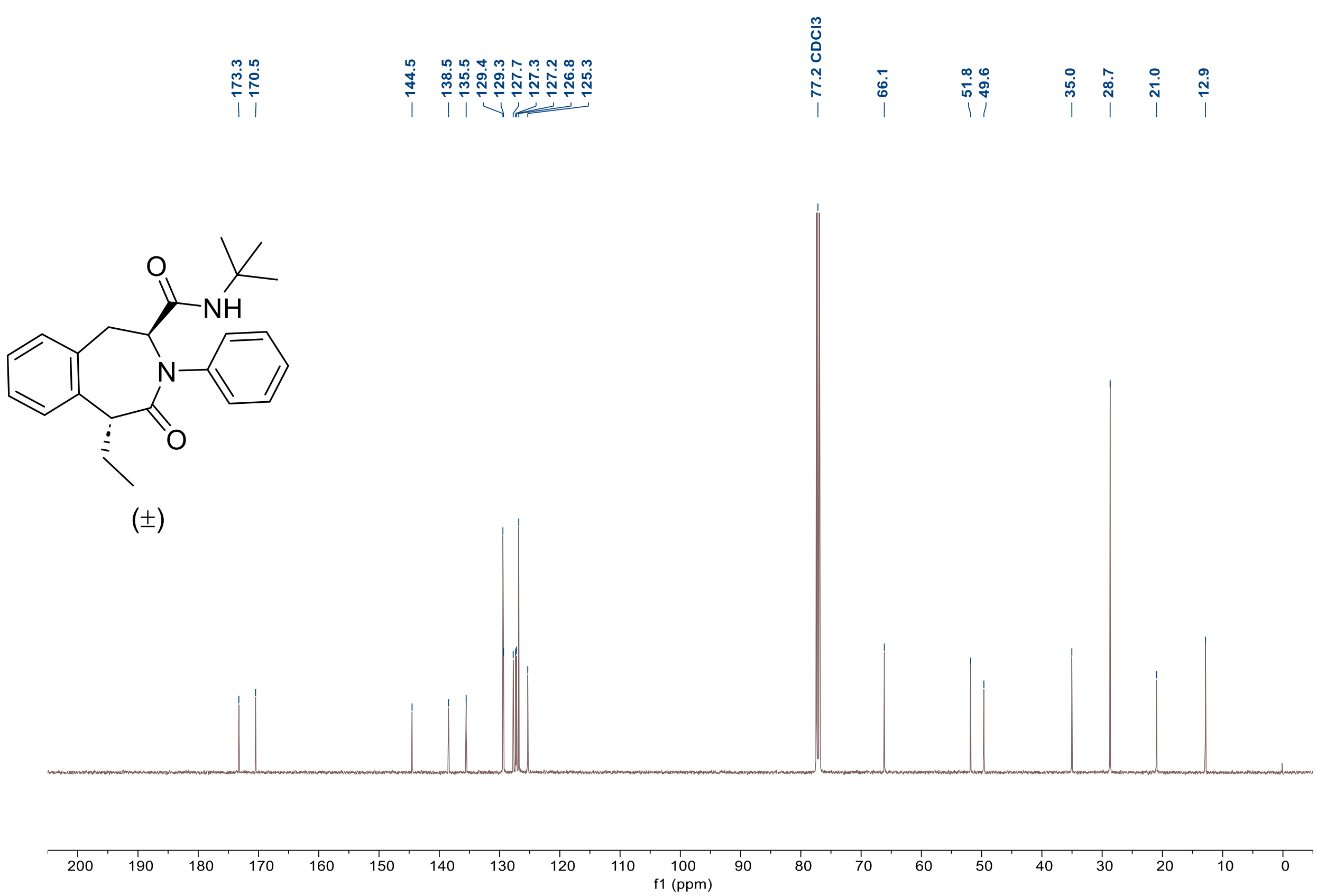

Supplement: Supplementary file 1 [file RA-016-D6RA06856A-s001.pdf]
